# Identification and comprehensive analyses of the *CBL* and *CIPK* gene families in wheat (*Triticum aestivum* L.)

**DOI:** 10.1186/s12870-015-0657-4

**Published:** 2015-11-04

**Authors:** Tao Sun, Yan Wang, Meng Wang, Tingting Li, Yi Zhou, Xiatian Wang, Shuya Wei, Guangyuan He, Guangxiao Yang

**Affiliations:** The Genetic Engineering International Cooperation Base of Chinese Ministry of Science and Technology, The Key Laboratory of Molecular Biophysics of Chinese Ministry of Education, College of Life Science and Technology, Huazhong University of Science & Technology, Wuhan, 430074 China

**Keywords:** *TaCIPK24*, *CBL*–*CIPK*, Expression profiles, Stress response, Preferential interactions, Wheat

## Abstract

**Background:**

Calcineurin B-like (CBL) proteins belong to a unique group of calcium sensors in plant that decode the Ca^2+^ signature by interacting with CBL-interacting protein kinases (CIPKs). Although CBL-CIPK complexes have been shown to play important roles in the responses to various stresses in plants, little is known about their functions in wheat.

**Results:**

A total of seven *TaCBL* and 20 *TaCIPK* genes were amplified from bread wheat, *Triticum aestivum* cv. Chinese Spring. Reverse-transcriptase-polymerase chain reaction (RT-PCR) and *in silico* expression analyses showed that *TaCBL* and *TaCIPK* genes were expressed at different levels in different tissues, or maintained at nearly constant expression levels during the whole life cycle of the wheat plant. Some *TaCBL* and *TaCIPK* genes showed up- or down-regulated expressions during seed germination. Preferential interactions between TaCBLs and TaCIPKs were observed in yeast two-hybrid and bimolecular fluorescence complementation experiments. Analyses of a deletion series of TaCIPK proteins with amino acid variations at the C-terminus provided new insights into the specificity of the interactions between TaCIPKs and TaCBLs, and indicated that the TaCBL–TaCIPK signaling pathway is very complex in wheat because of its hexaploid genome. The expressions of many *TaCBLs* and *TaCIPKs* were responsive to abiotic stresses (salt, cold, and simulated drought) and abscisic acid treatment. Transgenic *Arabidopsis* plants overexpressing *TaCIPK24* exhibited improved salt tolerance through increased Na^+^ efflux and an enhanced reactive oxygen species scavenging capacity.

**Conclusions:**

These results contribute to our understanding of the functions of CBL-CIPK complexes and provide the basis for selecting appropriate genes for in-depth functional studies of *CBL*–*CIPK* in wheat.

**Electronic supplementary material:**

The online version of this article (doi:10.1186/s12870-015-0657-4) contains supplementary material, which is available to authorized users.

## Background

The calcium ion (Ca^2+^) functions as a ubiquitous second messenger in plants, and participates in signal transduction in response to various environmental and developmental stimuli. The transient Ca^2+^ signal in the cytoplasm of plant cells is captured by a variety of Ca^2+^ sensors including calmodulins, calmodulin-like proteins (CML), calcium-dependent protein kinases (CDPKs), and calcineurin B-like (CBL) proteins [1–3]. These Ca^2+^ sensors transfer the environmental and developmental signals to downstream components, causing a series of physiological and biochemical changes.

CBLs, which harbor a core region consisting of four conserved EF hands, capture the Ca^2+^ signal, and interact with CBL-interacting protein kinases (CIPKs) to phosphorylate their target proteins [4]. The CIPKs with the NAF motif for interaction with CBL belong to the SnRK3 protein family, whose members have a specific Ser/Thr protein kinase domain [5]. Recently, CIPK proteins were shown to phosphorylate their interacting CBL proteins at N-terminal conservative Ser residue (AtCBL1-Ser^201^, AtCBL2-Ser^216^, AtCBL4-Ser^205^ and AtCBL10-Ser^237^) [6–8] and this proper phosphorylation of CBL was proved to be absolutely required for the *in vivo* activation of the AKT1 K^+^ channel by CBL1-CIPK23 and CBL9-CIPK23 complexes in oocytes [6]. The *CBL* and *CIPK* gene families have been described in *Arabidopsis thaliana*, *Oryza sativa, Zea mays*, and *Brassica napus* [9–12].

In previous studies, CBL–CIPK complexes were found to play very important roles in responses to external stimuli. The salt overly sensitive (SOS) pathway was the first CBL-CIPK network to be identified, and this CBL-CIPK complex comprised CBL4 (SOS3) and CIPK24 (SOS2). The CBL4 protein was shown to interact with CIPK24 and recruit it to the cytoplasmic membrane, where it activated the H^+^/Na^+^ (SOS1) antiporter to enhance salt tolerance [13]. This result indicated that the CBL-CIPK complex functions in regulating sodium ion homeostasis. Meanwhile, V-ATPase proteins were shown to directly interact with SOS2 to rescue the salt-defective phenotype of *sos2* mutant plants [14]. In recent years, many CBLs and CIPKs have been shown to promote stress tolerance by balancing the intracellular ion concentration in plants. The sodium concentration was markedly lower in the *Arabidopsis cbl10* mutant than in control plants, in both normal and salt-stress conditions [15]. The *CBL10*-overexpressing lines exhibited a K^+^-sensitive phenotype, like that of the *akt1* mutant under low K^+^ conditions. The interaction between CBL10 and AKT1 was verified in bimolecular fluorescence complementation (BiFC), yeast two-hybrid (Y2H), and co-immunoprecipitation experiments, and was shown to impair AKT1-mediated inward potassium (K^+^) currents [16]. AtCIPK23 could directly interact with CBL1 to promote K^+^ uptake under low K^+^ conditions by phosphorylating and activating the K^+^ channel (AKT1) in *Arabidopsis* and rice [17, 18]. However, a recent study suggested that CBL1/CBL9 might interact with AKT1 independently of CIPK23 [19]. Moreover, in response to low-K^+^ conditions, CIPK9 was recruited to the tonoplast by CBL3 to regulate K^+^ homeostasis in *Arabidopsis* [20].

Over-expression and mutant analyses of *CBL*s and *CIPK*s have greatly enriched our understanding of their functions. The *cbl2/cbl3* double mutant showed defects in growth and ion homeostasis, possibly as a result of disrupted vacuolar H^+^-ATPase activity [21]. In another study, CBL2 and CBL3 were shown to affect seed development and morphology [22]. The ectopic overexpression of *ZmCIPK16* in the *Arabidopsi sos2* mutant enhanced the expression of *SOS1* and improved salt tolerance under saline conditions [12]. *AtCIPK3* was induced by abscisic acid (ABA) and stress conditions and established a molecular connection between stress- and ABA-induced Ca^2+^ signals [23]. CBL10 and CIPK6 were shown to positively regulate both immunity- and disease-associated programmed cell death in tomato and tobacco [24]. The *oscipk31* insertion mutant was hypersensitive to ABA and stresses (salt, mannitol, and glucose), and showed altered expression levels of several stress-responsive genes when it was exposed to these abiotic stresses during the seed germination and seedling stages [25]. In a recent study, a few AtCBL members were also found to target to another group of enzymes 5'-methylthioadenosine nucleosidases by yeast two-hybrid system, indicting an additional level of complexity on the CBL-mediated signaling networks [26]. However, with respect to the larger families of CBLs and CIPKs, few CBL-CIPK complexes have been functionally characterized, especially in bread wheat (*Triticum aestivum* L.), which is one of the most important food crops. Bread wheat has an allohexaploid genome with a size of approximately 17 GB, making it one of the largest and most complex plant genomes. In recent years, the genome sequencing of *T. aestivum*, *Triticum urartu,* and *Aegilops tauschii* has promoted research on bread wheat, especially studies on agronomically important gene families related to disease resistance, abiotic stress endurance, and grain quality [27–29].

In our previous work, we demonstrated that transgenic tobacco overexpressing wheat *TaCIPK14* and *TaCIPK29* showed increased tolerance to salinity and drought stress, respectively [30, 31]. In this study, seven *TaCBL* and 20 *TaCIPK* genes in wheat were identified and cloned by a genome-wide analysis combined with expressed sequence tag (EST) assembly. Here, we present the results of analyses of expression profiles, the specific interactions between TaCBLs and TaCIPKs, and the stress tolerance of transgenic *Arabidopsis* plants expressing *TaCIPK24*.

## Results and discussion

### Identification of *TaCBL* and *TaCIPK* gene families in wheat

**Fig. 1:**
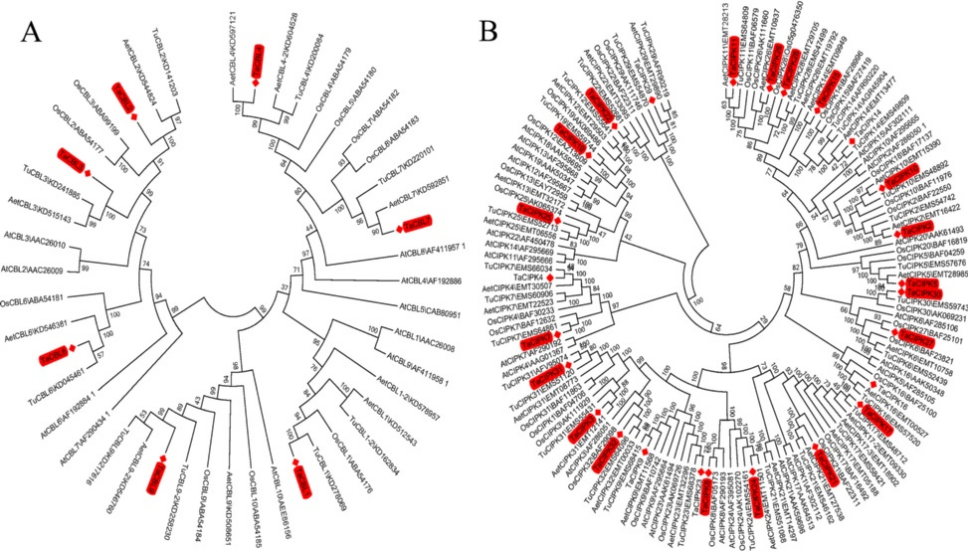
Phylogenetic tree of CBL **a** and CIPK **b** proteins of *T. aestivum* (Ta), *A. tauschii* (Aet), *T. urartu* (Tu), *A. thaliana* (At) and *O. sativa* (Os). Multiple sequence alignment of CBL and CIPK proteins was performed using ClustalW. The wheat proteins were indicated with “rhombus” symbol. The genes belonging to A, B and D sub-genomes were marked with letters suffix and genes cloned in this study were colored with red

The bread wheat genome is thought to have formed *via* fusion of three ancestral genomes, which are believed to be related to *T. urartu*, *Aegilops speltoides,* and *A. tauschii*. These fusion events are thought to have occurred several hundred thousand years ago [32]. We searched the wheat genome and found 24 *CBL* (7, 9, and 8 loci in sub-genomes A, B, and D, respectively) and 79 *CIPK* (21, 34, and 24 loci in sub-genomes A, B, and D, respectively) loci on wheat chromosomes (Table 1). The relative positions of these loci on chromosomes were identified on genetic maps (Additional file 1). The protein and DNA sequences of *T. urartu* and *A. tauschii* (the B sub-genome was not analyzed in this work) were retrieved from NCBI and all the *CBL* and *CIPK* nucleotide sequences from these three species (*T. aestivum*, *T. urartu*, and *A. tauschii*) were clustered against each other (Fig. 1). The closest matching genes were regarded as equivalent partners, as shown in Table 1. Ten and nine genes of *T. urartu* and *A. tauschii*, respectively, had no equivalents in *T. aestivum,* implying the existence of potential un-identified *TaCBL*/*TaCIPK* genes. Additionally, *TaCIPK15-A*, *TaCIPK26-A*, *TaCIPK8-D,* and *TaCIPK22-D2* (wheat specific genes) had no equivalents in *T. urartu* and *A. tauschii* (Table 1). Considering that the physical maps of current wheat genome do not cover all the chromosome arms, it is difficult to know whether these potential un-identified *TaCBL*/*TaCIPK* genes are resulted from gene retention, duplication and whether these wheat specific genes are the results of the loss of *CBL* and *CIPK* genes after polyploidization. In the future, a more detailed wheat physical map may resolve this question.

**Table 1:** Identification of *TaCBL* and *TaCIPK* gene families in wheat genome

Gene ^a^	Gene names related to Chr.	Amino Acid	Exon	Chr.	Contigs	Gene Position Start end		*Triticum urartu*	*Aegilops tauschii*	GenBank Acc No.
*TaCBL1* ^a^	*TaCBL1–A1* ^*^	215	8	1AL	3974931	2941	6756	KD278069	–	JX2443002
	*TaCBL1–A2* ^*P*^	–	–		–	–	–	KD162834		
	*TaCBL1–B*	–	–	1BL	3900149	166	3357	–	–	
	*TaCBL1–D1*	–	–	1DL	1737972	560	1484	–	KD512543	
	*TaCBL1–D2*	–	–	1DL	464815	15		–	KD578957	
*TaCBL2* ^a^	*TaCBL2–A*	215	8	5AS	1464783	5151	93449	KD141203	–	JX243003
	*TaCBL2–B* ^*^	245	7	5BS	2281375	80	4667	–	–	
	*TaCBL2–D*	225	–	5DS	2741958	5680	9158	–	KD544824	
*TaCBL3* ^a^	TaCIPK3–A^P^	–	–	–	–	–	–	KD241885	–	JX243004
	*TaCBL3–B* ^*^	–	8	4BS	4897686	4127	8100	–	–	
	*TaCBL3–D*	226	–	4DS	2274776	2834	7044	–	KD515143	
*TaCBL4* ^a^	*TaCBL4–A*	–	–	1AL	3951410	100	1380	KD20084	–	JX243005
	*TaCBL4–B*	–	7	1BL	3917583	1507	2702	–	–	
	*TaCBL4–D1* ^*^	218	–	1DL	2274485	1479	2868	–	KD597121	
	*TaCBL4–D2*	–	7	1DL	2273437	4080	5129	–	KD604528	
*TaCBL6* ^a^	*TaCBL6–A* ^*^	218	5	5AL	2189286	246	2332	KD045461	–	JX243006
	*TaCBL6–B*	226	8	5BL	10906286	5260	8935	–	–	
	*TaCBL6–D*	–	–	5DL	4595540	1995	5159	–	KD546381	
*TaCBL7* ^a^	*TaCBL7–A*	–	–	1AL	3887581	5948	7521	KD220101	–	JX243001
	*TaCBL7–B* ^*^	–	8	1BL	3897439	22549	24230	–	–	
	*TaCBL7–D*	213	8	1DL	2195979	3589	5243	–	KD592851	
*TaCBL9* ^a^	*TaCBL9–A1*	296	8	3AL	4339532	1	784	KD258230	–	JX243010
	*TaCBL9–D1* ^P^	–	–	3D	–	–	–	–	KD508651	
	*TaCBL9–A2*	–	5	3AS	3303239	2125	4252	KD217616	–	
	*TaCBL9–D2* ^P^	296	–	–	–	–	–	–	KD546760	
	*TaCBL9–B1* ^*^	296	9	3B	10535092	2016	4459	–	–	
	*TaCBL9–B2*	–	–	3B	10578811	5421	7996	–	–	
	*TaCBL9–B3*	–	–	3B	10423444	3743	5534	–	–	
*TaCIPK2* ^a^	*TaCIPK2–A*	–	1	2AS	1136699	1058	2612	KD178374	–	KJ561791
	*TaCIPK2–B*	452	1	2BS	5209173	4420	6031	–	–	
	*TaCIPK2–D* ^*^	452	1	2DS	5352995	3439	6170	–	KD537702	
*TaCIPK3* ^a^	*TaCIPK3–A*	456	14	2AS	5278308	778	4232	KD224079	–	KJ561800
	*TaCIPK3–B*	447	14	2BS	5215259	1889	4792	–	–	
	*TaCIPK3–D* ^*^	447	14	2DS	5388639	6194	9544	–	KD551511	
*TaCIPK4*	*TaCIPK4–B*	448	2 2	5BS	2276618	1805	3100	–	–	–
	*TaCIPK4–D*	432		5DS	2735985	15903	17161	–	KD505215	
*TaCIPK5* ^a^	*TaCIPK5–A*	433	1	3AS	3302816	1652	3448	KD141920	–	KJ561802
	*TaCIPK5–B*	466	1	3B	10642252	4923	6712	–	–	
	*TaCIPK5–D* ^*^	464	–	3DS	1035659	1	1176	–	KD508285	
*TaCIPK6*	*TaCIPK6–A* ^*P*^	–	–	–	–			KD207883	–	–
	*TaCIPK6–D* ^*P*^	–	–	–	–			–	KD557345	
*TaCIPK7* ^a^	*TaCIPK7–A1* ^*P*^	–	–	–	–			KD036546		KJ561803
	*TaCIPK7–A2* ^*P*^	–	–	–	–			KD051070		
	*TaCIPK7–A3* ^*P*^	–	–	–	–			KD103566		
	*TaCIPK7–B*	–	1	5BL	10732661	4349	5764	–	–	
	*TaCIPK7–D* ^*P*^	431	–	–	–			–	KD522041.1	
*TaCIPK8* ^a^	*TaCIPK8–B*	–	3	3B	10524427	1670	3978	–	–	KJ561804
	*TaCIPK8–D*	464	–	3DL	6956205	367	2132	–	–	
*TaCIPK9*	*TaCIPK9–A*	–	14	5AL	1668033	412	3585	KD006963	–	AK332473
	*TaCIPK9–B*	446	–	4BL	6995603	4780	8912	–	–	
	*TaCIPK9–D*	–	15	4DL	14472121			–	KD554016	
*TaCIPK10* ^a^	*TaCIPK10–A*	445	1	4AS	5974107	2247	3728	KD248893	–	KJ561787
	*TaCIPK10–B*	384	1	4BL	7036526	5067	6516	–	–	
	*TaCIPK10–D* ^*^	439	1	4DL	14429928			–	KD540780	
*TaCIPK11* ^a^	*TaCIPK11–A*	438	1	3AL	2838468	1233	3144	KD051997	–	KJ561788
	*TaCIPK11–B1* ^*^	507	1	3B	10507835	1	1663	–	–	
	*TaCIPK11–B2*	507	–	3B	10507836	1	1544	–	–	
	*TaCIPK11–D*	–	1	3DL	6897880	8953	10856	–	KD509836	
	*TaCIPK11–D2* ^*P*^	507	–						KD556601	
*TaCIPK12*	*TaCIPK12–A*	–	2	1AL	3875215	1	875	KD167973	–	–
	*TaCIPK12–B*	–	1	1BL	3828145	1	326	–	–	
	*TaCIPK12–D*	–	1	1DL	2112699	1	418	–	KD507283	
*TaCIPK13*	*TaCIPK13–B*	–	1	3B	10753103			–	–	–
	*TaCIPK13–D* ^*P*^	–	–	–	–	–	–	–	KD502264	
*TaCIPK14*	*TaCIPK14–A*	–	1	4AL	7147326			KD239100	–	JX879754
	*TaCIPK14–B*	449	1	4BS	4951408	11612	13239	–	–	
	*TaCIPK14–D*	444	1	4DS	2283949	3085	4712	–	KD546797	
*TaIPK15* ^a^	*TaIPK15–A*	449	1	5AL	1668033	1866	3335	–	–	KJ561789
	*TaCIPK15–B*	–	1 –	5BL	10796477	2838	4331	–	–	
	*TaCIPK15–D* ^*^	438		5DL	4490016	1021	2355	–	KD560960	
*TaCIPK16*	*TaCIPK16–A* ^*P*^	–	–	–	–	–	–	KD143696	–	AK331419
	*TaCIPK16–B*	–	2	5BL	10860020	4595	6233	–	–	
	*TaCIPK16–D* ^*P*^	447	–	–	–	–	–	–	KD721304	
*TaCIPK17* ^a^	*TaCIPK17–A1*	–	6	1AS	3313233	19	2337	KD250230	–	KJ561790
	*TaCIPK17–A2*	–	11	1AS	3259469	1075	3521	KD132920	–	
	*TaCIPK17–B1* ^*^	–	12	1BS	1265003	1916	5673	–	–	
	*TaCIPK17–B2*	466	12	1BS	3424219	1678	5957	–	–	
	*TaCIPK17–D1*	466	–	1DS	1916039	456	3542	–	KD530641	
	*TaCIPK17–D2*	–	–	1DS	1890755	1	3079	–	KD563756	
	*TaCIPK17–D3*	–	11	1DS	271737	999	3311	–	KD589116	
*TaCIPK19* ^a^	*TaCIPK19–A* ^*P*^	–	–	–	–	–	–	KD117636	–	JX234011
	*TaCIPK19–B1*	–	2	3B	10573976	10157	13650	–	–	
	*TaCIPK19–B2*	483	1	3B	10753103	13446	14093	–	–	
*TaCIPK20*	*TaCIPK20–A*	525	4	6AS	4406549			KD224235	–	–
	*TaCIPK20–B*	–	12	6BS	2936057			–	–	
	*TaCIPK20–D*	–	11	6DS	2096196			–	KD544165	
*TaCIPK21* ^a^	*TaCIPK21–A* ^*^	–	12	2AS	5304926	1845	6080	KD277172	–	KJ561792
	*TaCIPK21–B*	451	–	2BS	5197551	4370	8637	–	–	
	*TaCIPK21–D*	–	–	2DS	5319123	2007	4463	–	KD511088	
*TaCIPK22* ^a^	*TaCIPK22–A*	–	–	2AL	6383902	1	934	KD203757	–	KJ561793
	*TaCIPK22–B1*	–	–	2BL	7988831	2848	4399	–	–	
	*TaCIPK22–B2*	–	–	2BL	8033849	3684	4533	–	–	
	*TaCIPK22–B3*	–	–	2BL	7949729	3040	4484	–	–	
	*TaCIPK22–D1*	–	1	2DL	9860188	4959	6607	–	KD500669	
	*TaCIPK22–D2* ^*^	433	–	2DL	9908802	3988	4888	–	–	
*TaCIPK23*	*TaCIPK23–A*	–	14	2AL	6369709	442	4149	KD007171	–	JX243012
	*TaCIPK23–B*	452	14	2BL	7939869	5925	8812	–	–	
	*TaCIPK23–D*	462	14	2DL	9894674	10517	14199	–	KD502068	
*TaCIPK24* ^a^	*TaCIPK24–A*	462	–	7AL	4552082	7689	11571	KD185370	–	KJ561794
	*TaCIPK24–B1* ^*^	–	–	7BL	6743884	3133	8220	–	–	
	*TaCIPK24–B2*	–	–	7BL	6725100	299	1430	–	–	
	*TaCIPK24–D*	–	14	7DL	3369425	6709	12113	–	KD554191	
*TaCIPK25* ^a^	*TaCIPK25–A*	446	–	7AL	4557464	1	906	KD203605	–	KJ561795
	*TaCIPK25–D* ^*^	–	–	7DL	3315123	3089	4703	–	KD578984	
*TaCIPK26* ^a^	*TaCIPK26–A*	–	1	6AS	4399246	659	2242	–	–	KJ561796
	*TaCIPK26–B*	481	–	6BS	1542240	652	2262	–	–	
	*TaCIPK26–D* ^*^	–	1	6DS	32939	832	2285	–	KD556601	
*TaCIPK27* ^a^	*TaCIPK27–B1* ^*^	486	–	5BL	10894042	2308	3872	–	–	KJ561797
	*TaCIPK27–B2*	–	1	5BL	10894043	2308	3872	–	–	
*TaCIPK28* ^a^	*TaCIPK28–A* ^*^	447	1	1AL	2752572	4061	5848	KD259754	–	KJ561798
	*TaCIPK28–B*	472	1	1BL	3799843	6758	8542	–	–	
	*TaCIPK28–D*	472	1	1DL	2281163	487	2269	–	KD506845	
*TaCIPK29* ^a^	*TaCIPK29–A* ^*P*^	472	–		–	–	–	KD176996	–	JX243013
	*TaCIPK29–B* ^*^	–	1	2BS	5246729	19006	20606	–	–	
	*TaCIPK29–D* ^*P*^	436	–	–	–	–	–	–	KD506473	
*TaCIPK30*	*TaCIPK30–A*	–	1	3AL	4303945	2592	3265	KD117636	–	AK330597
*TaCIPK31* ^a^	*TaCIPK31–A*	478	14	4AS	5932853	800	4412	KD170661	–	KJ561799
	*TaCIPK31–B* ^*^	449	15	4BL	7038614	5874	10593	–	–	
	*TaCIPK31–D* ^P^	449	–	–	–	–	–	–	KD566562	
*TaCIPK32* ^a^	TaCIPK32–A^P^	–	–	–	–	–	–	KD060684	–	KJ561801
	*TaCIPK32–B* ^*^	–	14	4BS	4875486	20714	24433	–	–	
	*TaCIPK32–D*	439	14	4DS	2325367	3990	77792	–	KD929083	

“–” represents no data available. “P” represents the potential genes were not identified in this work. “a” represents those genes that were amplified in this study. “*” represents those amplified genes that were identified as transcripts from indicated chromosomes

Among the identified *TaCBL*/*TaCIPK* genes, the cDNAs of seven *TaCBLs* and 20 *TaCIPKs* were successfully PCR amplified from hexaploid bread wheat (*T. aestivum*) using gene-specific primers (Additional file 2) from a cDNA mixture prepared from wheat cv. Chinese Spring as the template. The genomic DNA and cDNA of all identified *TaCBL* and *TaCIPK* are provided in Additional file 3. To explore the evolutionary relationship of the CBL/CIPKs, two phylogenetic trees were generated based on all transcribed CBL/CIPKs from *T. aestivum*, *T. urartu*, *A. tauschii*, *A. thaliana*, and *O. sativa*. As expected, the genes from *T. aestivum*, *T. urartu,* and *A. tauschii* had the closest evolutionary relationships, and these genes had closer phylogenetic relationships with rice (a monocot) than with *Arabidopsis* (a dicot). Thus, the names assigned to the identified genes in wheat followed their homology to rice *CBL*s and *CIPK*s (Fig. 1). To analyze the structural characteristics of *TaCBL* and *TaCIPK* genes, the gene structures including exons and introns were mapped based on the wheat genome sequence (Additional file 4). Some genes' structures were partly characterized with exons/intros, because of lack adequate genome sequences. Only the structures covering the ORF were regarded as complete structures and used for further analysis. All the *TaCBL*s were intron-rich, with five to eight introns. Among the *TaCIPKs*, 26 *TaCIPK*s had no introns, and the other 15 *TaCIPK*s had 5–14 introns. For most genes, there were no obvious differences in gene structure among genomes A, B, and D (e.g., *TaCIPK3*, *TaCIPK10* and *TaCIPK23*). Some genes (like *TaCIPK31, TaCBL9*) had a different intron length between the structures in different genome locations.

The CBL proteins containing EF-hand motifs are known as Ca^2+^-binding proteins, and show similarities to CML and CaM proteins. Each of the seven TaCBL proteins harbored four EF-hand motifs. Although these motifs in CBLs did not shared high similarity compared with CaM and CML proteins in other species (Arabidopsis, rice, *C.elegans*, yeast, zebrafish, mouse, and human), they were conserved among the CBLs in *Arabidopsis*, rice, and wheat (Additional file 5). As for the TaCIPK proteins, all 20 TaCIPKs (the genes were amplified by PCR) had domain structures similar to that of AtSOS2; that is, these 20 TaCIPKs harbored a protein kinase catalytic domain (PKC) and the NAF/FISL motif. Considering that CIPK proteins belong to the SnRK superfamily, we selected some representative SnRK superfamily proteins for comparisons of their structural features. As shown in Additional file 6, the PKC domains in SnRK family proteins were conserved and had some highly consistent sites, especially in the activation loop, while the NAF/ FISL motif was unique to CIPK proteins.

### Identification and validation of TaCBL–TaCIPK interactions

We analyzed the physical interactions between TaCBL and TaCIPK proteins in wheat using the Y2H method. As shown in Table 2 (Additional file 7), the interactions between TaCBLs and TaCIPKs showed different strengths and specificities. TaCBL1 strongly interacted with five CIPKs (TaCIPK3, 5, 14, 15, and 25). TaCBL2 strongly interacted with 11 CIPKs (TaCIPK3, 5, 11, 14, 15, 17, 21, 25, 26, 27, and 32). TaCBL3 showed strong interactions with 10 TaCIPKs (TaCIPK3, 8, 11, 14, 15, 17, 21, 26, 27, and 31) out of 19 TaCIPKs. TaCBL4, the ortholog of AtSOS3 (CBL4), strongly interacted with six TaCIPKs (TaCIPK3, 5, 14, 15, 26, and 31). TaCBL6 strongly interacted with five TaCIPKs (TaCIPK5, 11, 15, 21, and 27), and TaCBL7 interacted with seven TaCIPKs (TaCIPK5, 14, 15, 21, 22, 26, and 31). TaCBL9 strongly interacted with only two TaCIPK proteins (TaCIPK11 and TaCIPK31). To view the interactions from a different perspective, we found that TaCIPK7, 10, 19, 22, 24, 28, and 29 did not interact with any of the seven TaCBL proteins assayed, implying that these CIPK proteins might perceive signals from other unidentified CBL proteins in wheat. We selected nine of the interactions (CBL1–CIPK15, CBL2–CIPK31, CBL3–CIPK15, CBL3–CIPK27, CBL4–CIPK15, CBL4–CIPK31, CIPK6–CIPK15, CBL7–CIPK27, and CBL9–CIPK31) detected between CBL and CIPK proteins using the Y2H assay for further analyses *in planta* using the BiFC method (Additional file 8). For all of the assayed TaCBL–TaCIPK interaction complexes, yellow fluorescence signals were observed when TaCBLs and TaCIPKs were co-expressed in epidermal cells of tobacco leaves. Notably, it seemed that the observed preferential interactions were inconsistent with the close phylogenetic relationships of the TaCIPKs, namely, the closely phylogenetic related CIPK pairs do not display same interaction profile. For example, TaCIPK14 and TaCIPK15, which had 81 % similarity, showed similar interactions with five TaCBLs (TaCBL1, 2, 3, 4 and 7) but different interactions with TaCBL6 and 9. Compared with TaCIPK14/15, other TaCIPKs showed lower amino acid sequence similarity and more diverse interaction specificity. Thus, sequence similarity or phylogenetic relationships are not sufficient to predict the result of CBL-CIPK interactions in the Y2H assay.

**Table 2:** Interactions of TaCIPKs with TaCBLs in yeast two-hybrid assay

	TaCBL1-BD	TaCBL2-BD	TaCBL3-BD	TaCBL4-BD	TaCBL6-BD	TaCBL7-BD	TaCBL9-BD
TaCIPK3-AD	+	+	+	+	-	-	-
TaCIPK5-AD	+	+	-	+	+	+	-
TaCIPK7-AD	-	-	-	-	-	-	-
TaCIPK8-AD	-	-	+	-	-	-	-
TaCIPK10-AD	-	-	-	-	-	-	-
TaCIPK11-AD	-	+	+	-	+	-	+
TaCIPK14-AD	+	+	+	+	+	+	-
TaCIPK15-AD	+ *	+	+	+*	+*	+	-
TaCIPK17-AD	-	+	+	-	-	-	-
TaCIPK19-AD	-	-	-	-	-	-	-
TaCIPK21-AD	-	+	+	-	+	+	-
TaCIPK22-AD	-	-	-	-	-	+	-
TaCIPK24-AD	-	-	-	-	-	-	-
TaCIPK25-AD	+	+	-	-	-	-	-
TaCIPK26-AD	-	+	+	+	-	+	-
TaCIPK27-AD	-	+	+*	-	+	-*	-
TaCIPK28-AD	-	-	-	-	-	-	-
TaCIPK29-AD	-	-	-	-	-	-	-
TaCIPK31-AD	-	+*	+	+	-	+	+*
TaCIPK32-AD	-	+	+	-	-	-	-

The interaction analyses of wheat TaCBL and TaCIPK proteins were performed by Y2H method. TaCIPKs and TaCBLs were respectively cloned to PGAD and PGBK Vector, and then co-transformed into Y187 strains. The transformants containing the target plasmid combinations were grown on selection medium (TDO: SD/–Trp/–Leu/–His/+10 mM 3AT) and indicated as growth (+, interaction) and no growth (−, no interaction). “*” represents the interactions were verified by BiFC assays

**Fig. 2:**
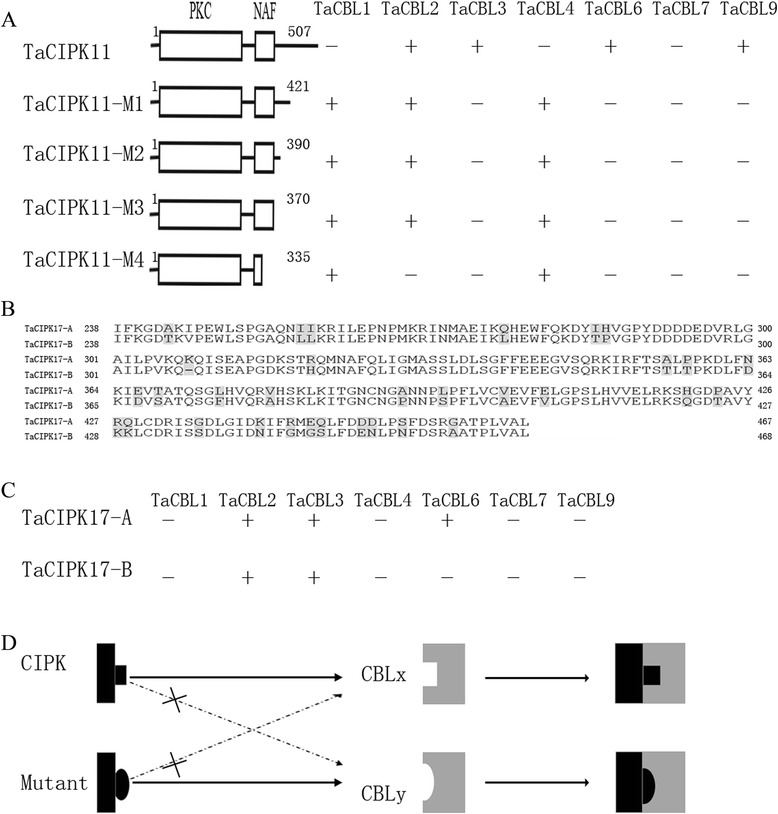
Deletions or mutations in *C*-terminal ends of TaCIPKs dramatically changes its ability to interact with TaCBLs. **a** series deletions in *C*-terminal ends of TaCIPK11 leads to changing interacting patterns with TaCBLs. The boxes indicate the PKC and NAF domains, and numbers beside the lines represent the beginning and the ending positions of TaCIPK11 protein fragments. **b**, *C*-terminal ends alignment between TaCIPK17-A and TaCIPK17-B. Residues with grey background indicates mutant amino acid sites. **c**, interactions of TaCIPK17-A and TaCIPK17-B with TaCBLs. Yeast was monitored on the selection medium and indicated as growth (+) and no growth (−). **d**, Schematic representation of the “concave-convex” model

Previous studies have shown that the NAF/FISL motif located in the *C*-terminal regulatory domain and the kinase activity of the PKC domain in the N-terminal region of CIPKs are necessary and sufficient for mediating interactions with CBLs [5, 6, 33]. The roles of the redundant parts (those other than the NAF/FISL motif) in the *C*-terminal region of CIPKs have not yet been fully studied. Here, the TaCIPK11 protein, which interacted with CBL2, 3, 6, and 9, contained a large *C*-terminal region (amino acids 311–507) that was not present in the other CIPKs. This led us to speculate about the role of this large fragment in regulating the specific interactions of this protein. Analyses of a series of *C*-terminal deletion mutants of TaCIPK11 (CIPK11–M1, M2, M3, and M4; M1–M3 containing the complete NAF/FISL motif, and M4 with a truncated NAF/FISL motif) showed that there were diverse interaction patterns between TaCIPK11 mutants and TaCBLs (Fig. 2a). CBL3, 6, and 9 could not interact with any of the TaCIPK11 deletion mutants, while TaCBL1/TaCBL4 physically interacted with some or all of the mutants. TaCBL2 lost its specific interaction with the minimal mutant (CIPK11–M4). TaCBL7 did not interact with TaCIPK11 or any of its mutants. It seems that TaCBL3, 6, and 9 require the last 86 amino acid for interaction with TaCIPK11 and also inhibits the binding of TaCBL1 and TaCBL4. These changes in the patterns of interaction may result from changes in protein structure after the deletions. In terms of this deletion assay, we supposed that the interaction between CBL and CIPK followed a “concave–convex” model as shown in Fig. 2d, similar to the model for the structures of AtSOS3-AtSOS2 and AtCBL2-AtCIPK14 complexes proposed by Sánchez et al. [34]. In this model, the spatial structures of CBLs/CIPKs have an important role for their interactions, and a consummate physical interaction depends on whether the NAF/FISL motif properly reach the CBL cavity. It is believed that the diversified spatial structures resulted from divergent *C*-terminal region of CIPKs explain the molecular basis of the selectivity of certain CBLs towards particular CIPKs. This led to another intriguing question; that is, do the nonsynonymous sequence variations of alleles from different wheat sub-genomes result in aberrant spatial structures that affect the patterns of interaction? To answer this question, we amplified *TaCIPK17*-*A*, which encodes a protein with many amino acid variations in the *C*-terminal regulatory region compared with the homologous TaCIPK17-B protein (Fig. 2b). Interestingly, TaCIPK17-A not only interacted with TaCBL2 and TaCBL3, but also with TaCBL6 in Y2H experiments (Fig. 2c). Despite two mutant sites (G^23^ to A^23^, G^48^ to D^48^) were observed at PKC domain, it is possible that several amino acid variations in the *C*-terminal regulatory region would confer different patterns of interaction. Such changes in interactions may render CIPKs capable of perceiving signals from other CBLs. These results indicate that the TaCBL-TaCIPK signaling pathway in wheat is very complex because of its allohexaploid genome.

### Expression patterns of *TaCBL* and *TaCIPK* genes at different developmental stages in wheat

**Fig. 3:**
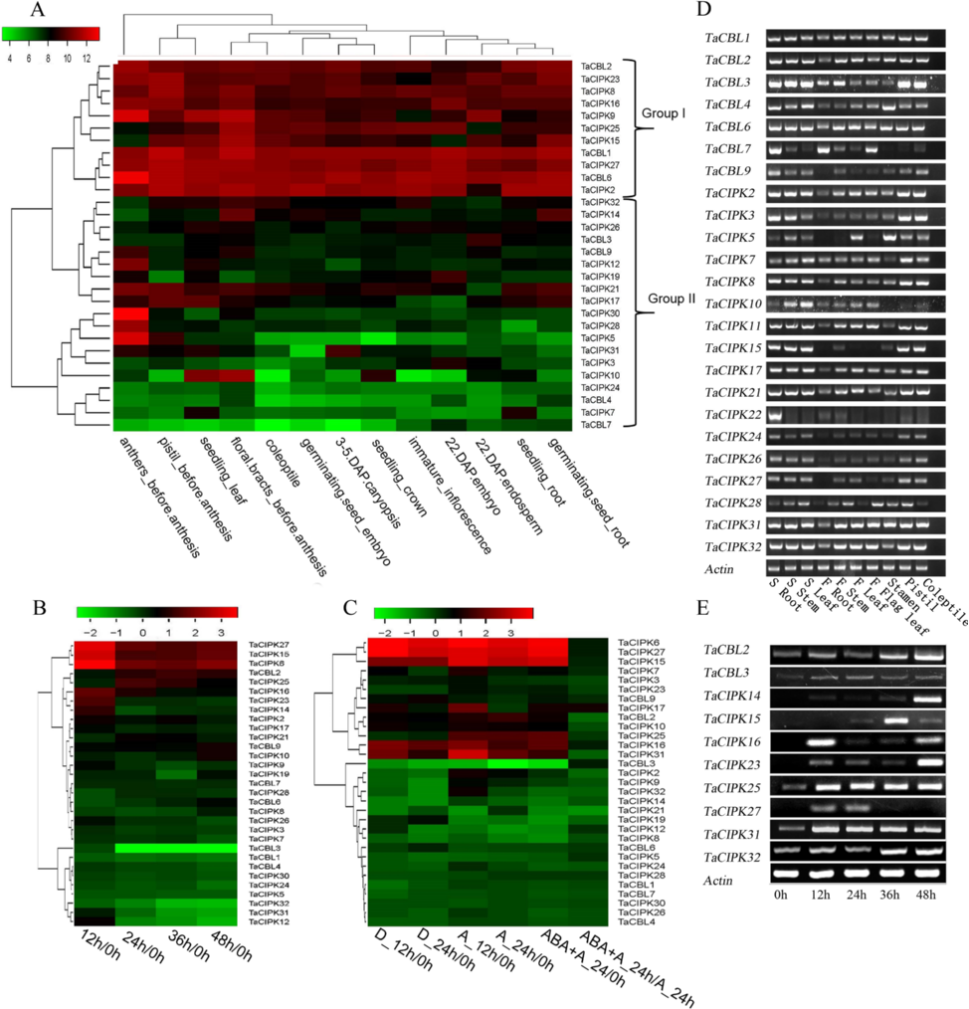
Expression profile analyses of *TaCBL* and *TaCIPK* in wheat tissues with microarray data **a**, **b** and **c**) and RT-PCR (**d** and **e**). **a** gene expression analyses in wheat whole life cycle using public microarray data. **b**, gene expression analyses during seeds germination process after immersed with water. **c**, comparative transcriptomic analyses between dormant and after-ripened seeds in both imbibed and ABA application states. The value (**b** and **c**) represents relative transcript levels compared to control (before immersion; 0 h). The up panels in **a b** and **c** represent log2 transformed values. **d**, gene expression analyses during wheat development using RT-PCR method. “S” and “F” represent seedlings and flowering stages, respectively. **e**, the induced genes in seeds germination (**b**) were confirmed by RT-PCR

To investigate the spatial expression patterns of *TaCBL*s and *TaCIPK*s in wheat, we detected their transcriptional levels using RT-PCR in 10 representative tissues, i.e., coleoptile, root (seedling and flowering stage), stem (seedling and flowering stage), leaf (seedling and flowering stage), flag leaf (flowering stage), pistil, and stamen (Fig. 3d). The results showed that the majority of *TaCBL*s and *TaCIPK*s were expressed at different levels in all tissues tested. Most *TaCBL* genes were constitutively expressed in all organs and at all developmental stages, although the transcript levels of certain genes were very low in some tissues. Some *CIPK* genes were expressed abundantly in certain tissues. For example, *TaCIPK22* was specifically expressed in the root at the seedling stage, indicating that it may play an important role in this organ. Moreover, the gene transcript levels differed between vegetative (seedling) and flowering stages. There were higher transcript levels of *TaCBL3* in seedlings than in plants at the flowering stage (roots, stems, and leaves). The flag leaf, as the last leaf of wheat, delivers the largest proportion of photosynthate to fill wheat grains [35]. Among the genes expressed in the flag leaf, *TaCBL7* showed lower transcript levels in leaves of seedlings and plants at the flowering stage, and higher transcript levels in the flag leaf, implying that it might play roles in regulating photosynthesis and/or metabolism in the flag leaf. To study the transcription levels of *TaCBL*s and *TaCIPK*s during the whole life cycle of wheat, we analyzed microarray data that were obtained from tissues at various developmental stages; these data were obtained from publically accessible databases. The genes formed two groups according to their expression patterns (Fig. 3a). Group I consisted of the genes with high transcript abundance in nearly all tissues (*TaCBL1*, *2*, and *6* and *TaCIPK2*, *8*, *9*, *15*, *16*, *23*, *25*, and *27*). Group II genes consisted of 4 *TaCBLs* and 15 *TaCIPKs* that showed different expression patterns in different tissues and stages. Some of them showed low transcript levels in all tissues. For most of the *TaCBL*s/*TaCIPK*s, their expression profiles in the microarray data matched to the RT-PCR results, but a few genes showed inconsistencies. For example, high transcript levels of *TaCBL4* were detected in the stamen in RT-PCR analyses, but low expression levels in anthers (before anthesis) were indicated in the microarray data. This inconsistency may have resulted from differences in the growth stages of plants between experiments, or from the different methods used.

To further clarify the detailed roles of *TaCIPK* and *TaCBL* in seed germination, we analyzed public microarray data representing the transcription patterns during seed germination. As shown in Fig. 3b, *TaCIPK6*, *12*, *14*, *15*, *16*, *23*, *25,* and *27,* and *TaCBL2* were up-regulated (log > 1, *P* < 0.05) after 12 h of imbibition, and then down-regulated (log < 1, *P* < 0.05). *TaCIPK12*, *TaCIPK31*, *TaCIPK32*, and *TaCBL3* were down-regulated during imbibition (log > 1, *P* < 0.05). Semi-quantitative RT-PCR analyses were conducted to confirm the *TaCBL* and *TaCIPK* transcription patterns at five stages of germination (0, 12, 24, 36, and 48 h). All of the selected up-regulated genes were induced during seed germination (the EST of *TaCIPK12* was unavailable, and the probe sequence of CIPK12 was not amplified), but the timing of induction differed (Fig. 3e). *TaCIPK16*, *TaCIPK25,* and *TaCIPK27* showed peak transcript levels after 12 h of imbibition, while *TaCIPK14* and *TaCIPK23* showed peak transcript levels at 48 h and lower transcript levels at other time points. These transcriptional patterns indicated that the genes played roles at different stages of germination. High transcript levels of *TaCIPK15* were detected only at 36 h, when the radicle of seeds emerged and the coleoptile became visible. There were high transcript levels of *TaCIPK16* at the initial stage of germination (12 h). For down-regulated genes (*TaCBL3*, *TaCIPK31* and *TaCIPK32*) (Fig. 3b), our data showed that these genes were actually up-regulated or showed no significantly changes in transcription. Moreover, despite some up-regulated genes were all validated in RT-PCR and microarray data, they displayed peak transcript levels at different time point. For example, *TaCIPK23* reached peak levels at 48 h in RT-PCR analysis, while it was up-regulated at 12 h followed with decreasing transcript levels in microarray analysis. The inconsistencies in the expression levels of genes may have been because the data were obtained using different methods, from different sub-species, or from seeds at slightly different stages of germination. We analyzed microarray data from another experiment, which focused on seed dormancy and after-ripening in bread wheat [36], and found that four genes (*TaCIPK7*, *10*, *17,* and *21*) corresponded to germination and that *TaCIPK31* was up-regulated gene during germination (Fig. 3c). Interestingly, *TaCIPK32* was down-regulated in dormant seeds, but up-regulated in after-ripened seeds. These data provided the preliminary information about the roles of TaCBL–TaCIPK in seed germination.

### Expression profiles of *TaCBL* and *TaCIPK* in response to abscisic acid and abiotic stresses

There is considerable evidence that *CBL* and *CIPK* genes play important roles in the responses to phytohormones and abiotic stresses [10, 11, 37]. Among the stress hormones, ABA is a well-known signaling molecule in biotic or abiotic stress responses [38]. Cold, drought, and salinity are crucial factors affecting wheat growth and crop yields, and these stresses often cause oxidative stress.

**Fig. 4:**
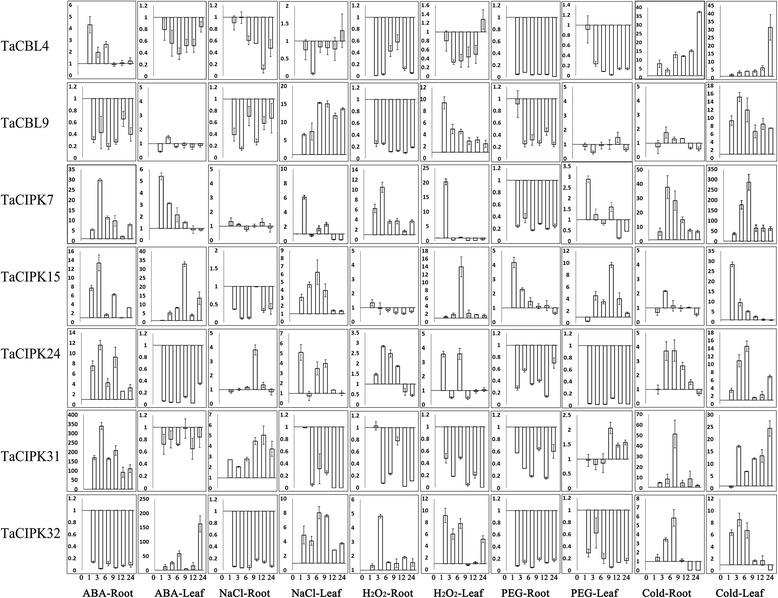
Expression analyses of *TaCBL* and *TaCIPK* in leaves and roots responding to various treatments, including ABA (10 μM), NaCl (200 mM), H_2_O_2_(10 mM), PEG (20 %) and cold (4 °C). Five time points (0 h, 1 h, 3 h, 6 h, 9 h, 12 h and 24 h) were used to detect the gene expressions. The vertical axis represents gene relative expression levels. Data is the mean of three biological replicates ± S.E

To investigate the expression profiles of *TaCBL* and *TaCIPK* genes under stress conditions, we conducted real-time quantitative RT-PCR analyses. We analyzed the transcript levels of two *TaCBL*s and five *TaCIPK*s in wheat seedling roots and leaves under salt (NaCl), H_2_O_2_, drought (PEG), and cold (4 °C) stress treatments, and in response to ABA application (Fig. 4). The transcript levels of all tested *TaCBL* and *TaCIPK* genes changed in response to many of the treatments. The genes that did not show significant changes in transcript levels (significant criterion: log > 2, up-regulated; log < 0.5, down-regulated) were *TaCBL9* in the leaf (ABA/PEG treatments) and in the root (cold treatment), *TaCBL7* in the root (NaCl treatment), *TaCIPK15* in the root (H_2_O_2_ treatment), and *TaCIPK31* in the leaf (ABA treatment). Among the analyzed genes, *TaCIPK31* exhibited remarkable ABA-induced up-regulation in roots (350-fold increase compared with that in the control at 3 h), but not in the leaves. Under salt stress, *TaCIPK24* was up-regulated in roots and leaves. Therefore, it was selected for further functional characterization. The expression patterns of the analyzed *TaCBL*s and *TaCIPK*s were not completely consistent between seedling roots and leaves. For example, *TaCBL4* was up-regulated in roots and down-regulated in leaves in response to ABA application. Several genes responded to the same stress; for example, *TaCBL9*, *TCIPK7*, *15*, *24*, and *32* were induced by H_2_O_2_. Among the five treatments, cold treatment resulted in the largest number of up-regulated genes, and none of the assayed genes were down-regulated by cold in roots or leaves. In brief, the expression patterns of the assayed genes differed among different treatments and tissues, indicating that various *TaCBLs* or *TaCIPK*s may participate in the signaling response to the same stress, and that a single *TaCBL* or *TaCIPK* might function in multiple stress responses.

In previous studies, monocot and dicot species were shown to have divergent gene expression patterns in response to abiotic stresses or ABA application [11, 39]. Thus, we conducted a comparative expression analysis of *CBLs*/*CIPKs* among wheat and two other important monocot crops (rice and maize). Microarray datasets of rice *CBL*/*CIPK* expression patterns in response to abiotic stresses were collected by Zhang et al. [11], and a dataset of maize *CIPK* expression patterns was obtained by semi-quantitative-PCR by Chen et al. [10]. The comparative analyses showed that several orthologous genes had similar transcription patterns. For example, the transcript level of *CIPK32* increased in leaves and decreased in roots under salinity treatment in wheat and maize. However, some orthologous genes exhibited divergent expression patterns. For example, *TaCBL4* showed increased transcript levels in roots and leaves under cold stress, while the transcript levels of *OsCBL4* decreased under cold stress. Thus, there are both conserved and divergent expression models for orthologous genes among wheat, maize, and rice.

Next, we focused on stress-responsive *cis*-elements to decode the *CBL*/*CIPK* expression models. These elements included the ABA responsive element (ABRE), the dehydration-responsive element (DRE)/C-repeat, and the low-temperature-responsive element (LTRE) [40–43]. Sequence analyses of 1-kbp sequences upstream from the start codon of the stress-inducible genes (*TaCBL4*, *TaCBL9*, *TaCIPK7*, *TaCIPK15*, and *TaCIPK24*) revealed that these genes contained putative ABRE, DRE, and LTRE elements (Additional file 9). These elements at least partly explained the stress-inducible expression patterns of these genes. However, even though *TaCIPK31* and *TaCIPK32* were induced by ABA, PEG, and cold, they contained only the ABRE element in their promoter regions (Additional file 9). This result is not so surprising, since we only searched for three *cis*-elements in this study, and there may be other unidentified elements that are important for stress-responsive gene expression. Generally, the putative stress-responsive *cis*-elements in promoter regions are crucial for stress-responsive expression, and activation of the *cis*-elements depends on the tissue, the development stage, and/or the genetic background. Therefore, there are many explanations for the diversified expression patterns of orthologous genes among different species.

### Over-expression of *TaCIPK24* improves salt tolerance in *Arabidopsis*

**Fig. 5:**
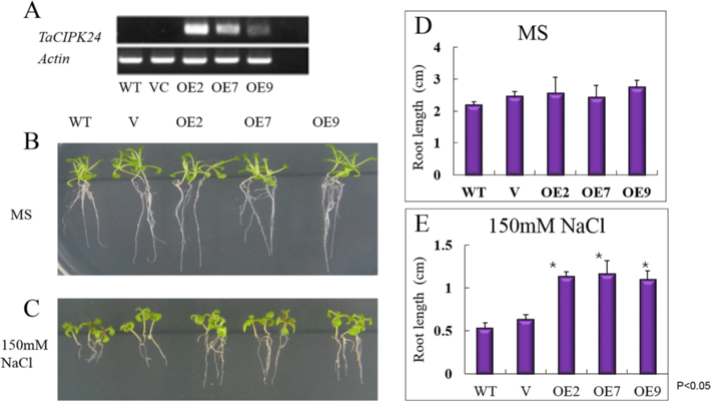
Overexpression of *TaCIPK24* in Arabidopsis. **a** *TaCIPK24* expression levels in selected transgenic lines, vector control and wild type. **b**, **c** the plants grown on control (MS medium) and stress condition (MS + 150 mM NaCl). **d**, **e** statistical analysis of the changing root length between MS control and salt stress condition (*p* < 0.05)

Previous studies showed that AtCIPK24 (SOS2) and BnaCIPK24 (homolog of TaCIPK24) participated in the salt stress response in transgenic *Arabidopsis* [11, 44, 45]. In our expression analysis, *TaCIPK24* was induced in roots and leaves under salt stress (Fig. 4). Therefore, we generated transgenic *Arabidopsis* plants over-expressing *TaCIPK24* (or harboring pBI121 as the empty-vector control). In total, 28 transgenic lines were obtained. Three independent T3 homozygous lines (designated as OE-2, OE-7, and OE-9) were selected for further analyses. The transcript levels of *TaCIPK24* were detected by RT-PCR (Fig. 5a). In plants grown on Murashige and Skoog (MS) medium, root growth did not differ significantly among the transformants, the empty-vector control, and wild type (Fig. 5b and d). However, the *35S*:*TaCIPK24* transgenic plants showed greater root growth than those of controls after treatment with 150 mM NaCl (Fig. 5c and e; *p* < 0.05). There were no significant differences in the phenotypes of aerial parts between transgenic lines and controls at the seedling stage under normal and salt-stress conditions. Therefore, our data indicated that TaCIPK24 promoted root elongation in transgenic plants under salt stress.

**Fig. 6:**
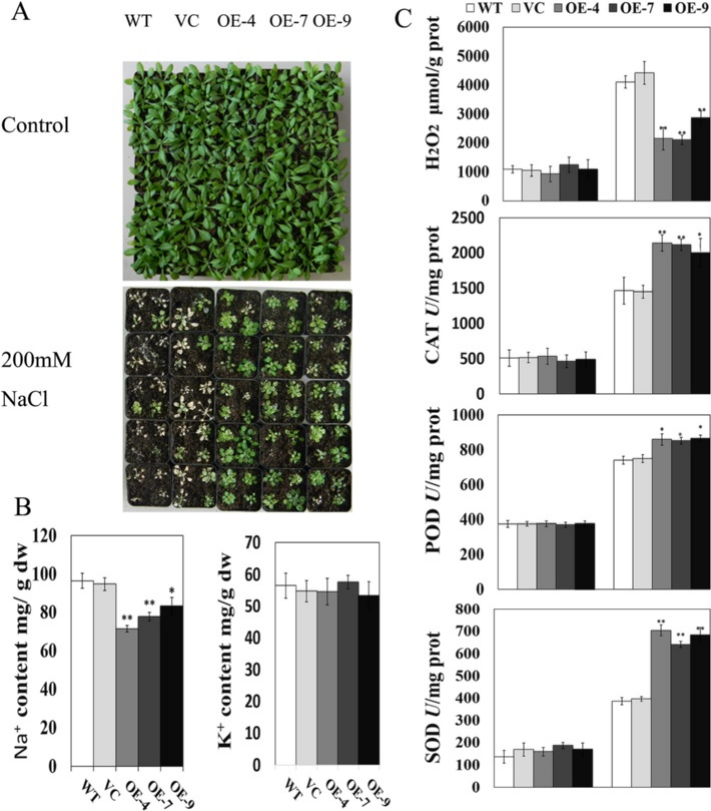
Phenotype of *TaCIPK24* over-expression lines in Arabidopsis. **a** phenotypic comparison of transgenic (OE-4, OE-7, and OE-9) and control (WT) plants under 200 mM salt stress and water control. **b** Na^+^/K^+^ concentration in seedlings under stress conditions. **c** contents of H_2_O_2_, oxidants malondialdehyde (MDA), enzymatic activities of peroxidase (POD), catalase (CAT) and superoxide dismutase (SOD) in control and transgenic plants

To further confirm the salt tolerance of *TaCIPK24*, seedlings of 10-day-old, soil-grown, wild-type and transgenic lines were irrigated with 200 mM NaCl for 25 d. As shown in Fig. 6a, the transgenic lines grew better than did the control plants. Furthermore, Na^+^ accumulated to lower levels in leaves of transgenic lines than in leaves of the empty-vector control and wild type. The K^+^ contents were not significantly different between transgenic lines and the empty-vector control/wild type (Fig. 6b), indicating that the improved salt tolerance may involve Na^+^ efflux. In addition, excess Na^+^ would generate reactive oxygen species (ROS), such as peroxide, superoxide, and hydrogen peroxide (H_2_O_2_), causing oxidative stress in plants. The accumulation of H_2_O_2_ was reduced in transgenic lines compared with that in the control plants (Fig. 6c). This result indicated that decreased Na^+^ contents led to lower ROS production (at least H_2_O_2_) and thus, the plants were able to grow well under salt stress conditions. Antioxidant enzymes are crucial for ROS scavenging and play roles in the salt stress response in plants. Therefore, we measured the activities of three important antioxidant enzymes (catalase, CAT; peroxidase, POD; and superoxide dismutase, SOD) in transgenic plants and controls under salt stress and normal conditions. The activities of CAT, POD, and SOD were higher in transgenic lines than in wild-type and empty-vector controls (Fig. 6c). These results showed that the ectopic expression of *TaCIPK24* enhanced salt tolerance through facilitating Na^+^ efflux and ROS scavenging.

## Conclusions

CBL and CIPK proteins play important roles in the Ca^2+^ signaling pathway and affect plant development, as well as participating in biotic and abiotic stress responses. *CBL*s and *CIPK*s have been identified and functionally characterized in *Arabidopsis*, rice, maize, and canola [10–12, 46, 47], but less is known about the *CBL*s and *CIPK*s in wheat. In this study, we identified seven *TaCBL* and 29 *TaCIPK* genes from wheat, and cloned cDNAs of seven *TaCBL*s and 20 *TaCIPK*s. Analyses of *TaCBL*–*TaCIPK* identified 24 *TaCBL* and 79 *TaCIPK* loci in the wheat genome, indicating that there were large numbers of these gene loci in the hexaploid wheat. Blast analyses suggested that four *TaCBL* and 55 *TaCIPK* loci were derived from the *T. urartu* and *A. tauschii* genomes. We studied the preferential interactions between TaCBL and TaCIPK proteins and found 44 CBL–CIPK interaction complexes. The proteins TaCIPK7, 10, 19, 22, 24, 28, and 29 did not interact with any of the seven TaCBL proteins assayed in these experiments. Expression analyses revealed that the majority of *TaCBL*s and *TaCIPK*s were expressed at varying levels in all tested tissues, although the transcript levels of some of these genes were very low in some tissues. We conducted RT-PCR analyses to monitor the transcript levels of seven *TaCB*L and 17 *TaCIPK* genes under abiotic stress and hormone treatments. Most were distinctly regulated by at least one treatment. Among them, *TaCIPK24*, the ortholog of *Arabidopsis SOS2*, was up-regulated by salt stress in root and leaf tissues. We generated transgenic *Arabidopsis* lines over-expressing *TaCIPK24*, and all of them showed enhanced salt tolerance. Overall, this study lays a solid foundation for further exploration of the roles of TaCBL and TaCIPK proteins in abiotic stress responses, and enhances our understanding the functions of the wheat *TaCBL* and *TaCIPK* gene families. These results also provide information that will be useful for the genetic manipulation of wheat to improve stress tolerance.

## Methods

### Identification of *TaCBL* and *TaCIPK* genes

The reported CBL and CIPK protein sequences from rice and *Arabidopsis* were used to search the NCBI UniGene database. All the selected EST sequences were collected to form a local database and were assembled into longer cDNA sequences. To further validate the integrity of *TaCBL* and *TaCIPK* gene sequences, these sequences were used to search the *T. aestivum* cv. Chinese Spring draft genome using the BLASTn method at Gramene (http://www.gramene.org/) and IWGSC (http://www.wheatgenome.org/). Rigorous criteria (E < 10^−5^, identity > 90 %) were used to ensure the reliability of the nucleotide sequences.

The gene-specific primers used for gene cloning were designed using Primer Premier 6 software (http://www.premierbiosoft.com/). *TaCBLs* and *TaCIPKs* were amplified by RT-PCR from cDNA mixtures of prepared from wheat (cv. Chinese Spring) that included cDNAs from the coleoptile, root, stem, leaf, flag, leaf, pistil, anthers, and from seedlings treated with polyethylene glycol, NaCl, cold, and ABA.

The structures of *TaCBL* and *TaCIPK* genes were illustrated using the Gene Structure Display Server (GSDS, http://gsds.cbi.pku.edu.cn/). Briefly, the cDNA sequences of *T. aestivum, T. urartu,* and *A. tauschii* were used to search the related genomic DNA sequences of *T. aestivum* following the instruction of GSDS. Multiple sequence alignments were executed using Clustal W (http://www.clustal.org) with default parameters. An un-rooted neighbor-joining phylogenetic tree was constructed using MEGA 5.2.2 software (http://www.megasoftware.net). Genome-based sequences were used to confirm the affiliations of genes in all 21 chromosomes. Chromosomal locations were identified on genetic maps with Genome Zipper V5 at IWGSC and visualized by MapDraw. To analyze putative *cis*-elements in the promoters of *TaCBL*/*TaCIPK* genes, 1-kbp regions upstream of the CDS were extracted from wheat genomic sequences and subjected to a *cis*-element search using PLACE (http://www.dna.affrc.go.jp/PLACE/signalup.html).

### Gene expression analyses

Wheat (*T. aestivum* L. cv. Chinese Spring) seeds were surface sterilized and then germinated in distilled water in a greenhouse at 25 °C. The germinated seeds were collected at five different time points (0 h, 12 h, 24 h, 36 h, and 48 h) to analyze gene expression during seed germination. After 2 weeks, young seedlings were subjected to ABA (10 μM), H_2_O_2_ (10 mM), cold (4 °C), NaCl (200 mM), and PEG (20 % v/v) treatments for 24 h. Samples were collected at 0 h, 1 h, 3 h, 6 h, 9 h, 12 h, and 24 h after the stress treatments for analyses. Leaf samples for analyses of CAT, POD, and SOD activities were collected at 25 d after the stress treatments. All the samples were harvested, frozen in liquid nitrogen, and stored at −80 °C until use. Total RNA was isolated from frozen tissue using a Plant total RNA Extraction kit (ZOMANBIO, http://www.zomanbio.com). The reverse-transcription reactions were performed using a FastQuant RT kit (TIANGEN, http://www.tiangen.com/).

The primers used for expression analyses are listed in Additional file 2. The microarray datasets were processed using R 3.0.1 with the “Affy” package. Expression intensities were first normalized using the arithmetic RMA normalization method and then used for expression analyses. *TaCBL* and *TaCIPK* DNA sequences were used to probe the Affymetrix microarray datasets with blastn in PLEXdb (http://www.plexdb.org/index.php). The microarray expression data and gene probes used for these analyses are listed in Additional file 10.

### Yeast two-hybrid (Y2H) and bimolecular fluorescence complementation (BiFC) assays

The Y2H assays were conducted using the MatchMaker yeast two-hybrid system (www.clontech.com). The ORF regions of *TaCBL* and *TaCIPK* were respectively sub-cloned into pGBKT7 and pGADT7 vectors and co-transformed into the Y187 yeast strain. The transformants were grown on double-dropout medium (DDO: SD/-Trp/-Leu) and selected on triple-dropout medium (TDO: SD/-Trp/-Leu/-His) containing 10 mM 3-amino-1, 2, 4-triazole (3-AT). For BiFC assays, the ORF regions of *TaCBL* and *TaCIPK* were sub-cloned into 35S-SPYCE and 35S-SPYNE vectors, respectively. After confirmation by sequencing, these constructs were separately transformed into *Agrobacterium tumefaciens* GV3101, then into tobacco leaves by *Agrobacterium* infiltration*.* Freshly transformed *Agrobacterium* cell cultures were re-suspended in suspension medium (10 mM MES-KOH (pH 5.6), 10 mM MgCl_2_, and 0.1 mM acetosyringone), adjusted to an OD_600_ of 0.5-0.8, and left at room temperature for 3 h before infiltration into tobacco leaves. Infiltrated leaf discs were collected 3–5 d later for observation under a confocal microscope. The *TaCBLs* and *TaCIPKs* primers used for vector construction are listed in Additional file 2: Table S1.

### *Arabidopsis* transformation and treatments

The full-length coding sequence of *TaCIPK24* was inserted into the pBI121 overexpression vector. *Arabidopsis* plants were transformed using the floral-dip method with *A. tumefaciens* strain EHA105 [48]. The assayed plants (transgenic *Arabidopsis* lines and wild type) were grown on MS medium until they reached the four-leaf stage, then transferred onto MS medium containing NaCl (150 mM) for salt stress analysis. The seedling root lengths were measured 7 days later. For salt tolerance analysis, 10-day-old soil-grown plants were irrigated with 200 mM NaCl at 5 d intervals for 25 d.

### Ion accumulation, H_2_O_2_ content, and enzyme activity measurements

To determine ion contents, samples were analyzed by atomic absorption spectrometry. Briefly, leaves were dried at 80 °C for 3 d, mixed with 30 % (v/v) H_2_O_2_, heated for 15 min at 180 °C, and then digested with concentrated HNO_3_ overnight. The samples were then analyzed using an atomic absorption spectrometer (AA-6300, Shimadzu Corporation). The H_2_O_2_ content and activities of CAT, POD, and SOD were measured by spectrophotometric methods using commercial detection kits (A064, A007, A084, and A001, Nanjing Jiancheng, China).

## Availability of supporting data

All the supporting data are included as additional files.

## Additional files

Additional file 1:
**Chromosomal distribution of wheat** ***TaCBL*** **and** ***TaCIPK*** **genes identified in this study.** The chromosome number is indicated on the top of each chromosome. (PDF 788 kb) Additional file 2:
**Forward and reverse primers used in gene cloning, expression and vector constructions.** (DOC 72 kb) Additional file 3:
**The genome DNA, cDNA and protein sequences of all identified** ***CBL*** **and** ***CIPK*** **of** ***T. aestivum***, ***T. urartu*** **and** ***A. tauschii.*** (DOC 1296 kb) Additional file 4:
**Exon-intron analysis of** ***TaCBLs*** **and** ***TaCIPKs*** **in wheat.** (PDF 1435 kb) Additional file 5:
**Analysis of EF-hand motifs in calcium binding proteins of representative species.** (PDF 147 kb) Additional file 6:
**Analysis of SnRK proteins of representative species.** (PDF 420 kb) Additional file 7:
**The interaction analysis of wheat TaCBL and TaCIPK proteins were performed by Y2H method.** (PDF 191 kb) Additional file 8:
**The bimolecular fluorescence complementation experiments.** (PDF 310 kb) Additional file 9:
**Putative ABRE, DRE and LTRE core sequences in the 1-kb promoter regions of the stress-inducible genes.** (PDF 114 kb) Additional file 10:
**Probes and selected microarray data in expression analysis.** (XLS 66 kb)

## Abbreviations

ABA: Abscisic acid
ABRE: ABA responsive element
BiFC: bimolecular fluorescence complementation
CBL: Calcineurin B-like protein
CDPK: calcium-dependent protein kinase
CIPK: CBL interacting protein kinase
CML: calmodulins, calmodulin-like protein
DDO: double-dropout medium
DRE: dehydration-responsive element
EST: expressed sequence tag
H_2_O_2_: hydrogen peroxide
LTRE: low-temperature-responsive element
MS: Murashige and Skoog medium
POD: peroxidase
qRT-PCR: quantitative real-time polymerase chain reaction
ROS: reactive oxygen species
SOD: superoxide dismutase
SOS: salt overly sensitive
TDO: triple-dropout medium
VC: vector control
Y2H: yeast two-hybrid
