# Supplementary material for: Identification and comprehensive analyses of the CBL and CIPK gene families in wheat (Triticum aestivum L.)
Source: BMC Plant Biol. 2015 Nov 4;15:269. doi: 10.1186/s12870-015-0657-4 (PMC4634908; doi:10.1186/s12870-015-0657-4)
Supplement: Additional file 1: — Chromosomal distribution of wheat TaCBL and TaCIPK genes identified in this study. The chromosome number is indicated on the top of each chromosome. (PDF 788 kb) [file 12870_2015_657_MOESM1_ESM.pdf]

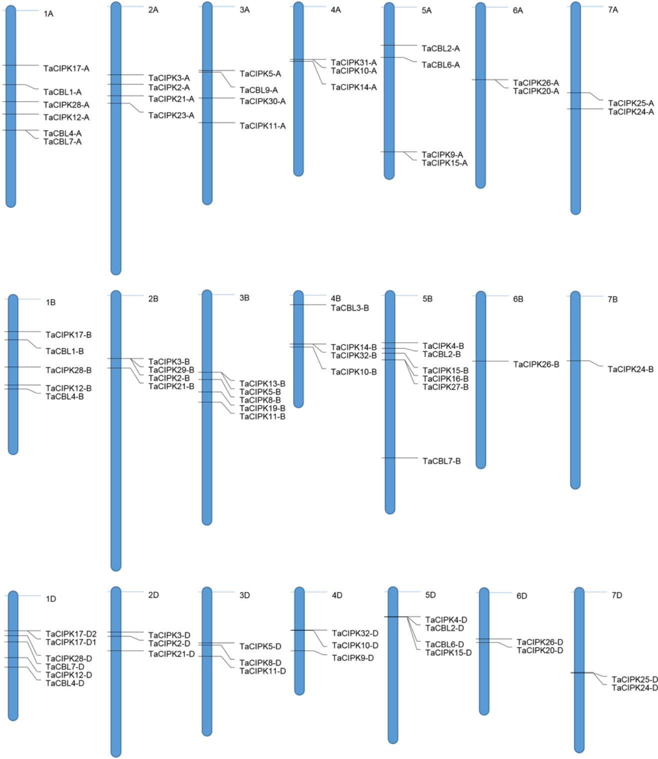

1A
TaCIPK17-A
TaCBL1-A
TaCIPK28-A
TaCIPK12-A
TaCBL4-A
TaCBL7-A
2A
TaCIPK3-A
TaCIPK2-A
TaCIPK21-A
TaCIPK23-A
3A
TaCIPK5-A
TaCBL9-A
TaCIPK30-A
TaCIPK11-A
4A
TaCIPK31-A
TaCIPK10-A
TaCIPK14-A
5A
TaCBL2-A
TaCBL6-A
TaCIPK9-A
TaCIPK15-A
6A
TaCIPK26-A
TaCIPK20-A
7A
TaCIPK25-A
TaCIPK24-A
1B
TaCIPK17-B
TaCBL1-B
TaCIPK28-B
TaCIPK12-B
TaCBL4-B
2B
TaCIPK3-B
TaCIPK29-B
TaCIPK2-B
TaCIPK21-B
3B
TaCIPK13-B
TaCIPK5-B
TaCIPK8-B
TaCIPK19-B
TaCIPK11-B
4B
TaCBL3-B
TaCIPK14-B
TaCIPK32-B
TaCIPK10-B
5B
TaCIPK4-B
TaCBL2-B
TaCIPK15-B
TaCIPK16-B
TaCIPK27-B
TaCBL7-B
6B
TaCIPK26-B
7B
TaCIPK24-B
1D
TaCIPK17-D2
TaCIPK17-D1
TaCIPK28-D
TaCBL7-D
TaCIPK12-D
TaCBL4-D
2D
TaCIPK3-D
TaCIPK2-D
TaCIPK21-D
3D
TaCIPK5-D
TaCIPK8-D
TaCIPK11-D
4D
TaCIPK32-D
TaCIPK10-D
TaCIPK9-D
5D
TaCIPK4-D
TaCBL2-D
TaCBL6-D
TaCIPK15-D
6D
TaCIPK26-D
TaCIPK20-D
7D
TaCIPK25-D
TaCIPK24-D

Additional file 1: Chromosomal distribution of *TaCBL* and *TaCIPK* genes identified in this study. The chromosome number is indicated on the top of each chromosome.
