# Supplementary material for: Identification and comprehensive analyses of the CBL and CIPK gene families in wheat (Triticum aestivum L.)
Source: BMC Plant Biol. 2015 Nov 4;15:269. doi: 10.1186/s12870-015-0657-4 (PMC4634908; doi:10.1186/s12870-015-0657-4)
Supplement: Additional file 2: — Forward and reverse primers used in gene cloning, expression and vector constructions. (DOC 72 kb) [file 12870_2015_657_MOESM2_ESM.doc]

Additional file 2. Forward and reverse primers used in gene cloning, expression analysis and vector constructions

| Gene | |  | Expression | Y2H | BiFC | Overexpression |
| --- | --- | --- | --- | --- | --- | --- |
| *TaCBL1* | F:tgggtggaggcggcggtgatg  R:aacaagactaactctcggtgtaatg | | F:cctcaaggacataacgaccacg  R:caacattagccagcatcaagca | F:cggaattcatggggtgcatccagtccacg  R:cgggatcctcatgtaacaatatcatcaacc | F:ctagtctagaatggggtgcatccagtccac  R:cgcggatcctgtaacgatatcatcaacct |  |
| *TaCBL2* | F:cgattgattgcttccttccgcccgct  R:tttgctccactttactatagattgc | | F:tacctcaaggacatcaccacaac  R:caaaggctcccaacaatactaaa | F:cggaattcatggtgcagtgtctcgacggc  R:cgggatcctcaggtatcgtcaacctgagaa | F:ctagtctagaatggtgcagtgtctcgacgg  R:cgcggatccggtatcgtcaacctgagaat |  |
| *TaCBL3* | F:agagagacgggcgttgacggac  R:atcaacaaaggcatttctgctacg | | F:tagttctcagacatccctcattac  R:aacaaaggcatttctgctacg | F:ggaattccatatgatgttgcagtgcctggaggg  R:aaaactgcagttatgtatcgtcgacctgagaat | F:ctagtctagaatgttgcagtgcctggaggg  R:cggggtacctgtatcgtcgacctgagaat |  |
| *TaCBL4* | F:gggagaggttgagagttgagacgcaaagac  R:ttttttgcacaggggattat | | F:gaatggcgacgacaggataga  R:gattggctcccttgcgttctc | F:cggaattcatgggctgcgtgttgtcatcgcc  R:cgggatccttatttgctgattccactgta | F:ctagtctagaatgggctgcgtgttgtcatc  R:cgcggatcctttgctgattccactgtaat |  |
| *TaCBL6* | F:acgtgcgggagagacgagcgatg  R:gaacgaacgaaacagtagaacgataaa | | F:ttctgatgaggttatagagggta  R:taggtatgtgaagagcgtttat | F:ggaattccatatgatggtggatttcccggaagg  R:aaaactgcagtcaagcatcctcgacctgaga | F:ctagtctagaatggtggatttcccggaagg  R:cggggtaccagcatcctcgacctgagagt |  |
| *TaCBL7* | F:gcggaaagacggttgttaactg  R:ttcggtgacaaaataaataaattgc | | F:gtagatcagacattcaagcaggc  R:tgatgtgtaccatttctcagcg | F:ggaattccatatgatgggatgtgcatcatcaaag  R:aaaactgcagctacaactcttcgtcgccg | F:ctagtctagaatgggctgtgtatcatcgaa  R:cgcggatcccaactcttcgtcgccggctc |  |
| *TaCBL9* | F:gcggcagggagggaggggagga  R:tcctcccctccctccctgccgc | | F:cgttcccaagttttgttttc  R:gggttgattatctgatgtcca | F:cggaattcatggcctcacgcttcaactc  R:cgggatccttagtcttcaaccgcggtatt | F:ctagtctagaatggcctcacgcttcaactc  R:cgcggatccgtcttcaaccgcggtattga |  |
| *TaCIPK2* | F:accttgatgacaccgcttcacagca  R:cggacgcctggtaattgattgcctc | | F:ggattggcattcctgttga  R:ccctgtcccaccgtaagt | - |  |  |
| *TaCIPK3* | F:aaatggcaggtcttcggagactggc  R:cccgatttgcctgttcttttcctcc | | F:ctgcacagataaaggatgttg  R:atgaaccagcctaaagctct | F:atcgatacatgtacaaggcaaggaggca  R:ctcgagtcatgtggagcctctttctt |  |  |
| *TaCIPK5* | F:ggaaagctctagccaccgcagact  R:tgctaccacaaaagggaaactgcaa | | F:ttggagtacgaacggttctgcaaa  R:tcagctctttgaggagggaagaaag | F:atcgatacatggagaggaagtccgccat  R:ctcgagttaaatgacatttcttttgg |  |  |
| *TaCIPK7* | F:taaaccacagcaccaccgccttgc  R:cccccttcaatctgccacaaagtcc | | F:gacgaggaggtccaggcgttc  R:atacatgtacatcccccaacccc | F:ggatccccatggccgtcgccaagagcaa  R:ctcgagtcacaattcctcgcatccat |  |  |
| *TaCIPK8* | F:gcagccgcagtacgactccaaagct  R:tgcagcaaacccacatgg | | F:actagccatctcacaattatgctt  R:cagcaaacccacatggttacag | F:atcgatacatgattgggggaggaggggg  R:ctcgagttagcgcttcgatagccggg |  |  |
| *TaCIPK10* | F1:tgatcatctgcaggtgttgtccaca  F2:tgttgtccacatcattgagtgcccc  R：3^，^RACE | | F:ggcagcagcacaagcatc  R:gcaagtacggaatagtgagaaaa | F:ggatccatggtagagaagaagggaaa  R:cccgggctagggctgctcaccttgcc |  |  |
| *TaCIPK11* | F:taagcggtgtgtgtggtgttggctt  R:tcccggaaacgtgttctttgacca | | F:cactcaaggatattgtatgggta  R:acacgaggtactttcatccg | F:ggatccccatgatggatgagaggaggac  R:ctcgagtcaatcttggtgtttgaagc |  |  |
| *TaCIPK14* | - | | F:ctccgcagccttagattattg  R:cgaaccctacgagcacca | F:ggatcccatgggctgtggttttatgca  R:ctcgagctactctagctgctggtggt |  |  |
| *TaCIPK15* | F:actctgcgtgggcccatttggt  R:catcagccccatgccatgcccagac | | F:cgccattcagactccgcta  R:accttgccaaatgctcct | F:atcgatacatggagaacagtgggaagat  R:ctcgagtcagccccatgccatgccc | F:cgcggatccatggagaacagtgggaagat  R:cggggtaccgccccatgccatgcccagac |  |
| *TaCIPK17* | F:taacccccctccacatcccaattc  R:gcctttatccttctcgcgcaaaa | | F:cgacaccactggttgccttg  R:atgaccctagcgtcctacaaca |  |  |  |
| *TaCIPK19* | F:ggctgctcctccacctaatcccctt  R:tcaccgtcagtgcccgagactacaa | | - |  |  |  |
| *TaCIPK21* | F:ctagctgctcccgcgtcttcttgac  R:ggcctggtgggtcctcttgtttaaa | | F:gtcatgtggagactgctcccta  R:gcctggtgggtcctcttgttt | F:gaattcatggcggcggcggagggggc  R:cccgggtcaaaagctactattaaatc |  |  |
| *TaCIPK22* | F:agcagagtcgtccctcctacctgca  R:agtacgtgcgtcatggcttggtccc | | F:atcatcgccatggttagggtta  R:atggttgaggtggaaagagcta | F:ggaattcatggggccggaagattcacc  R:ggatccatcatttgagaatttaagac |  |  |
| *TaCIPK23* | - | | F:ggactcttctccgccgttat  R:tcaggcgacgaagtcaaca | - |  |  |
| *TaCIPK24* | F:gacagacggcggaggggggc  R1:cagtcaccgcagaagcaactggga  R2:gcagcaaagctccaacttggcaatt | | F:atggcactaccacggatacctt  R:tcagtcaccgcagaagcaact | F:cccggggatggcgggcgcggcgaggaa  R:ggatccctagcaagtagttgtcctca |  | F:ggatccatggcgggcgcggcgaggaa  R:cccgggctagcaagtagttgtcctca |
| *TaCIPK25* | F:tccaaacacccgcccaagaagc  R:tgccatgatacatcggtgatccacc | | F:gccttcctccgacgttgaatg  R:tgatccaccgccaacactgt | F:cccggggatgggggatcggccaaagct  R:ggatcctcacgcaacattcaacgtcg |  |  |
| *TaCIPK26* | F:gtgcgggatattgtcgtggtaggcc  R1:gcagtggttgcatctcgtcgtgttc  R2:ggtagcagatttggaatggggccg | | F:gcacccgctccctgagaaat  R:ccgattactcctgtggctgct | F:cccggggatggaggataggaggacaat  R:ggatccttactcctgtggctgctgcgat |  |  |
| *TaCIPK27* | F:aattgccaccgcaagcacgtcgag  R:accagcagcagccatcaacttcgg | | F:cgctcaaggacatcgtgtgg  R:cggatagcaacagtgacagacag | F:cccgggtcaaaagctactattaaatc  R:gaattcatggaggacgcggcggaggg | F:tgctctagaatggaggacgcggcggaggg  R:cgcggatccagtgggggcaggggggt |  |
| *TaCIPK28* | F:gtgctcgtgctggaagaggcaa  R1:tgcagcgataacaatctgaagaggc  R2:gccgagccttcgtttccaacaa | | F:atctttaatggaggtgggt  R:ctctgctgttgtaactttgc | F:gaattcatggaagagaggagtgtttt  R:ctcgagttattgtggcggtggggcat |  |  |
| *TaCIPK29* | F:ttactaccccacagtcacgaccgcg  R:cgccgaccaggtagcagtcacca | | F:agcgttagcagtcacaggtca  R:caccaatcccatctccgaat | F:ggatccccatgccgtccgcctccagcgc  R:gagctctcacactgcctgaggcttca |  |  |
| *TaCIPK31* | F:gcatttgtcctgctcctcctcctcc  R:cgatctgcacaacccattccgca | | - | F:atcgatacatgtatcgggccaagagagc  R:ctcgagtcatgccacggtgctgttat | F:tgctctagaatgtatcgggccaagagagc  R:cggggtacctgccacggtgctgttatctt |  |
| *TaCIPK32* | F:cccgagcccccttccctcctg  R1:ggacaagcacagctgacaacagggc  R2:cgacttgcgaaaacccacatgcc | | F:tagcagcagcttgggagaagg  R:acaacaccagtggaggacagg | F:cccggggatgagtacaaccaaggtg  R:ggatccctaggaaggttggatctgaa |  |  |
|  |  | |  |  |  |  |
