# Supplementary material for: Identification and comprehensive analyses of the CBL and CIPK gene families in wheat (Triticum aestivum L.)
Source: BMC Plant Biol. 2015 Nov 4;15:269. doi: 10.1186/s12870-015-0657-4 (PMC4634908; doi:10.1186/s12870-015-0657-4)
Supplement: Additional file 3: — The genome DNA, cDNA and protein sequences of all identified CBL and CIPK of T. aestivum, T. urartu and A. tauschii. (DOC 1296 kb) [file 12870_2015_657_MOESM3_ESM.doc]

Additional file 3: Genome sequences (1), the putative CDS sequences (2) and 1kb promoter region sequences (3) of *TaCBL*s and *TaCIPK*s are listed with FASTA format.

**1. *TaCBL*, *TaCIPK* genome sequences**

>TaCBL1_1AL_3974931

tacgagggagggggcaccacacaaaggcacgacaatctcttagctgtgtgtggcgcccctctccacggtttcgttccttggtcatattttcggagtgcttaggcgaagccctgcggagatagcttcatcaccaccatcatcacgtcgtcgtgctgcaggaactcatctactacttcgtccgtcttgctggttcaagaaggcgaggatgtcatcgagctgaacgtgtgctgaacacggaggtgccgtacgttcgatacttgatcggttggatcgcggagaagttcgactacatcaaccacgttagtaaatgcttccgcttacgatctacgagggtatgttgacacactctcccctctcgttgttgtgcatctccatggatagatcttgtgtgtgcgtagaatttttttgttttccatgcaatgttccccaacaccacgagcctcctccactgtgctcctttggctccgcgggtgtcttctgtccggaaaaaaatctccaaaaagtttcattgcatttgtacttcgtttggtattgatattctggaaagtaaaaaacaagcagaaaagaacaactggcaggcactatgttaatagatagtcccaaaaaatgatataaagttgctaataaatgaatataaaacatctaagattaataatataacaacatggaacaaacaaaaattatagatacgttagagacatatcagtctgctaccagatgggacactgaatatcttcttaaccatgtctcttgaaaagtggatctccttgttgtcattaatcccacaattcacaaaaattaaacaaacgagtagatgttgcaagaaaaagagaaaaggtgttgtgcaacagagagaagaacataaggagatattgcatacttaagcacacgactcttagtgttaatactcataactatgaaatcgagaagcccttcaggaggaggaaccaaaggagagatgtatagcaaatccccgaatctggacttgcttattacatacctcttcttccctttgatggcattggctatgtcgcgcagccactttgccgaccatcgagtttggtagatgggcatgctgcgaacacaaaacaaaaatgaaaaaaagcaggatgctatgagcatgcatctctagtgatctaaagaaaatgaaaaacaatactgagattagtggaaaattgtatacatgatgcaaaaatcaggatttttttagctaactcaagcaggcatctaatttcagaaaatgaaagctgtttataacattccttattcagaagcatataacctaaatcccctctgctaaaagctacaggaaatgcagcaagaaatgagctatgagcaagtctagcaaatttcctctactgaaaactaaagctaaaattcacaacccatgagctgcaaaaaaatgacctaacaagcatgagctaaaaaaggtagaatgagctatagaaactatgagctattcagaaattaacacggtgggatgctgcaactttaagtttatcacaagctatgagctttagagaaaatcataaggaatcctacagaaactatgaactccagaaattcagaaataatacgacggatcctgcagctttaagtccctttgcatgacattaccaatgcagaaaattcagcaagcacaactaaaaaatcaaacctgaaactcagtaaaattcagcaggatatctacaaacacctaaaactcattggaacaatacaacaaaagcacaaactctgctcacatgtgccctggcaaaaaacatgttaatctgcaaaaaatatgtaaacaaattctgcaacacatccaaatcatgcaacctaactacaccggcgacctacaacaccaccgaactatctacaccgacaaccactctccaacaaactagtcgtctccctgctagtcgcgtcgtcgtcgtcatctcggtggatatgccacccctatggatccagctgccccgggggagaaccgccatgaacgcctacagaatcaccagaaatgcgggaaaaaggggaggggaggggtctcagatccatacctgctggagtcgacgtcggcgttgccactgcgctggatatgcccggtcgcccaatgccttgcgccgctggccccaccgccctcccagatccagaagtgtcgccctccaagagatacgagtcgacggagctgccagttgcatgaatgttggtcctgaaacaagggctctgtttcagaaaacggacgcgcgtgacgcggaagtgggacgctcttgtctgtctaatcaacagactatccgacggacgttgagctggcggacgcggcgcgtgatcggtcggctggctgaaggtaagcttttcggtagcaatataggtagaaagaaatattgtgttgttttgaatgatttaaaaattgctcaccacgcgtgcctcgcagtaatatgcagggttgatctacaacgggagggcgacaagtggaatgcatatgcgttccaagctagtgcggcatggacgcgcgtcgtcttttatgcgcccacactgcacgactcgggccaggttcagacttttgggccggtctctgattcgaagcagactcgctcccggtccccggtggatttggaccgaccgtcacgtgcgtaaagggtgggggcttgagccggaaggcgactactactaccaccagctctcgctgttggttgttccgctacagagagattccgcgactccatatcccaccagccaaaaccagaccgccgaccgcccgcccgcccgcacgcctcccctgaccgccggaatcccgcgctgacggcccggatccgcccgcccgcccgcttgctgcccgctgcccgctgcctcccctgctggcgctgcggctccacacttctatgcagtgggtggcggcggcggtgatgcgtgattggtcgcggcggcgctaggttctcttctccggccgcggcgagctaggccatggggtgcatccagtccacgccgaagcggaggcagcaccctgccggctacgaggaccccgtccacctcgcctcccagaccgcctgtgagctcgctcgctcgcatcccttttacccttttctacgcaacccattccgtgaatccagctcccgcttcatttctcgaaaaagggtaaatggatccagcttctgcttccgcctacgcgtgctagctctcgcgcactcgggattaggatttggaatggaacttgtttcgcgctgccagccgtgatcctgtagacagtggaggcaaatcgaataatctgaccagtagttatgattaacttatgttttagtttctttttgcgggggaacttatgttttagttgctgtgatattcatcggacatctgctattgatccacctcctgccttgtgaattctcattgggtgttttctgactccgtgcacttgctgttctgttcatgtagtcagcgtcagcgaagttgaggctttgttcgagctgttcaagagcattagtggctcagtgatcgatgacgggctgatcaacaaggtaccactttcttacgaacgtgtgattccttatcaggttgacatacacggacatcaaataagcacccgagacctgtttttgcgtactttttgtcggtaccattgaaacgaaatctgacatcaacgatgctatacacacgacacatgtgcacgatttgtggacgatgtagttaatcaagccgttggtttgtgggaataaactgcaggacatcgtctgattttgaatcgtgcatgaatcggccacacaggcgtcgtgcgcaaagcagtttcgatctgacattgctatgctgaaattacattgcgcatgtaccgacccttttacaactgagcaacttatgcagcaataccctttttcggtgattacatttagccatcagtagttctaatttctctaacatagtaactgaacatggattcttgttgtatatgtcaccaatcgtttattcttgctgtacaactcaatgagcctgtttgataaatagctacaacttagagccattattatagcataatgactatgacacggtgaagcctagtagagctcgggagcacatgctcctggctctaaataagtattttatctcttccaaaaaaatattagaagtttcacacatttaggtgagtattaggtagattttgagttttatgcaaaatttccattcttgtggtctgtgcaaaatatagaataaaaattatagcacaaaaatgccttttggctccttattatggtcttttccatacataagtcaacacatcttctcacgaagaacttgaaagaacttgttcctaagataaccgtgtcacgaacccacaatgtttcctgcatcagatattattgcaagttatatgaacatcatgtagctcaaggttttagccgttaccttgcatctcacgttatcgtagtctagctaaacttccaaggctcgtttcatggtttacttcttacataagccaactgcttgcttcgcatgttcataatgcaaaatttgctttgatcattcatggtagagttctagacaccttgggagaatgcgttccttctccagtgtaattataaatgtatcttatggtcatatatgtcatagaatttattacttgttgtcatgttaaccggtacatgtctgtgcaattatatgtaccacagtgaactatcatctcgttgtcattgggtactatattcactggcaactgaaactatgcgtaatttatattcatatatacgcaggaagagttccagcttgcattgttcaaaaacacaaggaaggaaaatctttttgctaatcgggtatacatctccttgtcaactattctcatttgcaatattacatattatatgctggggctcaatgttatcaatacaattgacttatatgaaattgaccttgtagatattcgacctgttcgatgtcaagaaaaggggtgtcattgattttggcgactttgttcgagctttaaatgtattccatccaaattttccagtggaagagaaaattgattgtaagttgttacgtttctgtcttgtcttcagtttgtagataccattctctctctttctgaaggttttgagctgcttgatacttacagtttccttcaagctatatgatatggatggtacaggctttattgaacggaaggaggtaatgtctggccttcttggtttgtgcctctgcgcatgttttttgcttctgaatttttctgaattagtcgtatatacaaaagaaatgcgttgctgtgtgactgaaaaatccagcagcgtgtgcacttatgtactactgaagcaaaataataattaaagagacaaggagatgtactcgtctcagcttccattctagatacataattcatatcttccaacgattgcggcacatgcttcacaaataaaacattgctagtacgacaatgactgcgattggcttttttctgactacggaagcaggactgacctaacactgaaagtgtagctactaattaagagcatctacatggaccagcatgcagcagcacacctaacaatatttgctagaccctaaaaccaggcagccccgcagatgctctataccatctggaagccaagccagaccaaaaagatcaggctgctgaactctgtagaaaccaagccatagcatataacaacaacaaatatttaagagtttttattggctcaaagtgagaattctggtgctcaccaagaaccaaatgataacggtaaacaatcgaaccaattatcatctcaaaagttgacattggtgccatcaccattttgtgtgtataaagtataggaatacttattagccttcgtttatcaattaaaatttctatcatcctcttgtacaggtaaagcagatgttgattgctcttctaggcgaatcagagatgagattatctgacgagattgttgagacaatcctggacaaggtattggatacttttttaagttgttagttgcttaggctgttgacaattcttgtgtccagtattgaagtgtagaccgataatgactggttaatggtgacagaccttttcagatgctgatacgaatcaggatggtaaaatagacagaacagaatgggagaattttgtgtccaggaatccttccttgttgaagataatgactctttcgtacctcaagtgagtatgatgtaacatataaattcagaagcattggccgcaactacgtttacgtgtccccatgcggctatttccagagaaatcaatttctagagcttaaactgagagtaattacttttggccgctgagctccagtgatcattatgttttgaaaatttcaaaaggtgcatttacgactttcaggcaaatctttaaaacaatcccacatgttcacgtgttcacgtggcaattcatagtgtattcatacatgcacatgtgggatttgttttgattttctgaaacctgtaaatgcacttctcaagaatttcaaattaaagggatgactggaggtcatgagcgaaatatctgcacccagacaagcacattctagttacatgtggtagtaattgtttatgtgaccttcactactattactgttatattgaactagcataaaaagtagtttaccataaaggccgtggcttcttgtaccttttccataggatgtttgatgacagtgtggtgcacgggttttttccatgaacatttgatacgaaggaacgaaaattcttgctaaccatttgtgttgatgtttcagggacataacgaccacgtttcccagctttgtgtttcactcggaggttgatgatatcgttacatgacgatgatcctcacaaagatgtacagttaacagaaagaaagaacgtatcatcagaatatctgcaacgctgcaacatgcttgatgctagctaatgttgctggagagtcgagagatagttttttttcctgggtggtgttcgttacaccgagagttagtcttgttaaatattaatgtatgtttgtgagcttgaatttgcaagtacttggcacaacttattatttgtcgagaatatggctgctaaataaactgttgttcaacctgactaacataattcgagcgtgcttcatgaatctactaccactatctgaaaaagatgaagaagcaaaacagcaccagaaagacgtttgagaacgcggccttagcaaatcagacaactaacaaatgcctttcaaagcctatatattactgtagatatcgaaatgttatgtctactgcagactactggagtattgtgttcacagcaacctgataaatttctaaagcatgtatggttgcaagtttgtgatcgatgaagttgttttatgagttgccgcgtaaaagtggttgcaaaaataatttactagcttcttgttgacaaattgcatcagtctcaacgaccaccacaagaaatgtattcttatggaactatttttctgcggattgcactcggctaatcaaacttgttttcttttgcagaaaaagttcaatcaaatggaattatcacaatctgtcgcaatgacaaacacaagacaatgcacacttgtgtgtaccgagtgtcgtaacgtctacattgtgctcggaagaaaactcatcaatcattcaaaactttgactatctatattttcccagatattatagatttttttgacatcatcctagatattatagatgaaattgggggcttctcctcctttcacctgggaaaaagtatttcccaagatatttctattgaacgcttgagaatcacatgtgctgatttgtctctatacttcataattaaaaagagattatatattcgtggtcaaagttgtgtattgacaccacgcgacaagtgtggtaaaagcaatctgaaattgacaaaaaaaagttgtgtattgatgagtttttttttgcatggtaatacgtgtctcatttatatcacaagggtcatagtaaaagccacgtacataccgacctgatagaactgaacaaatagcattacgatagtctttgcacgaaggaaccaagaagtaaaattacaaataagaccgaagaaacctctgagcttgacaccaacgactgtcacttgcctctggcaccaccataacagccaccaaagaaagaaattgacggatcacctccacaatcgagcttgacgcggctccatcgctgatatgcagctttgcggacctccgaggtggctcgctaaaggcgaaaccattaccgttgaatgaatcagaccatagcaacactccggatacgtcatcaaactccagatctaataccccacatgactaagacgccggaggaggaaaccatacttgacatccatgggccacaaaaccagtacacggtccaccatcttctagatgccgccgagaatatgcatccgctcctgtactacctcccaagcttcgcaccgtcgctggagcaaacgtcgttgcaatggcggagcccgaggacacaggtccatcacgaggatgctgccgccgccacaccatcattgcttgaacagactggattccaaattcatccccaaccatagaaccatcacctcatcggagtagtatctgacgaaattttattcagcgccaccatcgccgccgtcgaatccaagacgatgaacaacctaaaaaataagctacttcacgctaaaactattcacgcgcgtagattcgatgaccctcctcaccaccgacgaccgaggttgtcagcggaggagagctgccggaggacggggcggaggaacttcgtggcggtgcaggcgagatcgcctctttcttcgatagggagaaagagcgatacgtagcggacaaacatgacgagttttttttaagcatcgtgtattgacgaactgatttgtctaaatacttcctccgtcccgcaataacatagcttgcaaaatgttcttagcttgcaaaatattcttatattgtgggacagagggagtacttcatatcatgtctttttttttgaaccgacggtattgcggggaagcagtttttttttaagaggggaagcagcctttttttttagtggatgctgggaagcagttgggcttttggcttttgagggagcttctctttatttaattttcagggcccaacagtggagtaaaaatatcttgcatggctggcgtgtcatctttgcgggaaaccccccgggcgcctgtcgtaattacaacagccagccgcccacgctctccctccccctcccccgcgcctcgtcaccgttccgatctctctctcctctccatctcgttcctcccccacctgcgcagccaagaagttcaaaccctagtccagcctgcgagcgccaacacgccgcgccgtgcggcttcccgcaagcaggtgctccggcagaccgcgggggatgagcggagcggcctacagggaccggggattcggcggcgccgcggagatggaccggaagcgcatcaaggaggcgctcgagaagcacacggagaggccgtccccgtccacctccaggggggcgtccagggagaaggagctgctcgccgccggcaagataaccactcagatcggcaaggtccccaaggtctccgacgtcggtgaggatccagccgttcgcccggcctctgtttcttgttttttgttgtgctccgcattgcctgcttttgccagaacgacgcggctcctgttattctagtggtagaggaaatcatatggtgcactgtcattgcatgcccgtgggaagcgacttgcgacgttcccccgtggttgcagtgagacgcggggaaacaggaagttcactgcattgcgtggagctgaggaatcgattccgtattgctctgtttactttacgtctgacgtccatgcttcatgtgcctatcatcagtaagtatcatgctgcatgggggaaagaaacagaggatagtaggcgaaacgaagtagggttgctgaagatggaatggcagagatggcttgaagactgtcgaggaataaagccttgtttttctttcgcctgtctagcatttttagctactggattacatgattgtctggtagagaatttctgattcttttctggtattggtttgctacaagtcatctcagtcttcttttcgtggctgattgtctgttaggtcagtagggctctcaagcttcagtcttttatcttttagactatggaatagtatttaaggcaaatgcaacatttgctgcaggataattttgggtatagaagcacgatttgattcactcctttacgatgctttccttcagcaggaaagatgctccatggggagtaaagtattttcctcgtgtaataccgtagattaaccagggagttacactaatgattttttggatcaatgacaagcgtgaccaaaaatcagtttggtttcatgcctgggaggtcgaccatggaagccattttcttggtatggcaacttatggagagatacagggagcaaaagaagggcttgcatatggtgctcactgacctggagaagacctatgataagatactgcggaatgtcttgtggtgggccttggtgaaacacaaagtcccagcaaagtacattaccctcatcaaggacatgtacgataatgttgtgacaagtgtttgaacaagtgatgtcgagacactgatgacttcccgattaagatagtactgcatcaggggtcagatttgagcccttatatttttgccttggtgatggatgaggtcacaagggatataacaaggagatatcccatggtgtatgctctttgcggatgatgtggtgctagttgacaatagtcgaacgggggtaaataggaagttagagttatggagacaaactttggaatcgaaagggtttaggcttagtagaactaaaaccgagtacatgatgtgcagtttcagtactactaggtgtgaggaggaggaggttagccttgacgggcaggtggtgcctcagaaggacacctttcgatatttggggtcaatgctgcaggaggatgggtgtattgatgaagatgtgaaccatcgaatcaaagccggatggatgaagtggctccaagcttctggcattctttgtgactacagagtgccacaaaagcttaaaggcaagttctacaggacggcggttcgacccgcaatgttgtatggcgctgggtgttggccgactaaaaggcgacatgttcaacagttaggtgtggcggagatgcgtatgttgagatggatgtgtggccacacgaggaaggatcgagtccggaatgatgatatatgagatagaattggggtagcaccaattgaagagaagcttgtccaacatcgtctgagatggtttgggcatattcagcgcaggcctccaggagctccagtgcatagcggacggctaaagcgtgcggagaatgtcaagagaggtcggggtagaccgaatttgacatgggaggaatccgttaagagagacctgaaggattggagtatcaccacagaactagcaatggacaggggtgcgtggaagcttgctatccatgtgccagagccatgagttggtcgcgagatcttatgggtttcacctctagcctaccccaacttgtttgggactaaaggctttgtgttgttgtgttgtgttgccactttgtttgcttctttctccaaccaagacacgcgtttaaccaaacataccaacatttattgccaaacctatttcaatagtctccgttccaaaataagcgtcgtggttttagttcaaatttggacttatttgaactaaaacaatggcacttattttgggatggagggagtattaactgagcatactttggctaatttaaccgcttgttttgcaagattgcaaatcagttagtttcaataatgatttttggccaattacattatttacttgtttctccatcctgacacatgtttaaccaaaattaccaagtgttgtcgctgaacctaattaataagtggtgaaacattagcaaaggttgactaattctaacttcatggggaaaaaaggacaattggagtatgcagtggatcgaacccgggatcacttgctttaggaagggcatctgtggctcttggccagtaggagacattggagctctttgcgtttattttggctaacaatcttatatgtagccacatgagttgaaacatgcatacatggaaagaggttagaatcggtatctacaaggagattggttggtttgagtcagcttctcgggttttagtttgctcctatgttttaaggcgtcgcctagtcgtcgcctaagcgtcgaggcgccccaggatggcaaggcgtctaccccgccttagacagacatgaagtgtacggctgtggctgctcttggggcttggagatgacggccccgctgtctcccagcaccatgaagaggagggaggactggaggctcctggctgccctagttgtggctgctcctggggcttgctgctgtgtgggagaagggtggggccatgaagaggaggggggactggaggagggaggaccagaagggtgtggcaccgtggcggctgtggtgggaaagaagggaagaagatagtggaattaggtcacgggggtgtatatggccactagatgggcttttgggccactgggaggtgcataagtagtggcccatctctcagccattgctctttttctagtattaatatgtgcagacctattgataactcttttcctgcaatgtttaaggcgttgccttgcctcgccttaccgcctcgccttggcgcttaggcgtcagagcggccctcctccgccttatgacgctttaccgccttaaaaacgtaggtttgctcacttctttatgtagccatgtatggaatcatacacatatcgaaagagggtagtatcaagacaaaagatgcgcatcaaaatactatccaaaaatgatatttatttgctctagaaaagttatggtagcagtagatcgaacccaaaatcatgcgcctccctgctgcccaccgctaccttgcccgcatgacgatggcggccacggctggaagggatgccgcgctcatggagggctacacaaggacacacaagtgttcgtcaaccgcctgaccgcggaagtgaaagcagccgaagcatcgatgtcacttccaaacgaaggaagaggccggactagttgaagtcgtagtagaatcctagttcaaattgtagtagaaatccttgttgaaatcatttggatatctttgttgaaatccgagtaataatccttgttgaaatcaagttcgaaacttcttccattgttgtttgatgagatagggaagaaagaggtcttgttagccaatgacaaaccgattccacatagagaattttttgaaggctatgtttatggcgattatctgtttgtgcaaaggctcgccgacctccaaaacattatggagtgtgctccatatgcaatatgatgactctacttatggtgactctgctagagttgctcttatcttttctgttcgaactggtcagttctgtttagtcttgtgttgtttgacgattgaacaccttagccttgccctagacacccttaggcgtatcgaacactataaattcaaaattatgtggagtaactcaataagagatttttttctcttcgctttatcgctatagtgatgttctttgtgtttttttaatctgtagaatatcatgcgtttttacttttgtgagccacatggatttttgtagtgtttgtcaggatgtcgctttactggaatagttgtgattactagcacaatgctcatgcgttgctatgaaactataaaatatgaggagttaggtcactggcttagaaagtcgttaaaatacattggtgtcctgcggcctttgcgtgcggttcacggtttaaaagagagaaaatacaatgaaagatcatgaaacgtgaattgaagaatcttaagtggtcacagtaggtcctttgcaagacataataacatccgaatgttgaaacatatgagaataggcattagtaagttgtctgcacaagatcatggatcgggtaggaagacaaggtctatcgtcgctttggaatcacagtttattcactcttgttctgtgatacagaactggaagaacttggttgggaagagaaacggaaaataagagcataagtttgagttggagtcgcatttctacatttgttatttgtgtcttctactacctctgtaacttaatataagacagttttttgacactgtgacagtgtcaaaagatgtcttatatttagttacggagggagtatatgtgttggtctcacctgaaattcgtagtgtacttgtttgtatttgttaggtttcacatgttagtaaaagtacatttgtttgtttttgtctcttgaaggttgttagtccatacatgtggataggggtgcatttgtttattgatgaaggttgttgattccacgtgtaaactcaccttcaactccaatctttataagtaggaaaagataaatatcattttttactatgtagactcctctaaccttcttattttggcactgcagaggaattcgaaactgacagtgaagattctgatgttagtggttctgaaggagaggatacatcttggatttcatggttctgtagcttgcgaggcaatgaattcttctgtgagattgatgatgattatatacaggatgatttcaatctctgtggcctaagcagtcaggtgccatattatgattatgcacttgatctcatcctagacattgagtcttctaatggtgagattgtttcttcttaagaagctacctttttttactgtaaatactaaatacaccagtaatggtgtaatacaattgacatgttatttctgtttggaaaattttccacttttcaaccttgaactctaaaagcgctttaaatgcaaccttgaactattgaaactgtccacttaaagctcaagcggtttcacagcggtggttttgactgatgtggaagccacgcagatgcaacctcaatgtcacgttgctgcatgtcatcaaattccattattttatctgtttgtacaattctggtatatctttttgtgaaagtttttaatccggatggtatagtttgttttagaattgcttgtgtctttagtgtgtgttcatggtggtgacatgccaatggtgtggacgacatgtggcatccatgttacccaaaaaaaaaagtgaaaccgtccaagggtgaaaagtgtgctatttcagtaatttgcagtagcaattcaaacggttttagagttcaggttgaaaactgaactttctccaagtttagggttataaagtggattttttcctttctgaatttatcatctattatttgtattttcagatcacataattatttttttaataacatatttttttatgctcatcaatttcatttcatttagaagcaaatttccagcaacctgtgtatattcatcctgcactctagtatccacagaaggtactgttcaggtttactattgaattcatctaagtggggccatttttgtgcaggtgatgtattcactgaggagcaaaatgaattaattgagtcatctgcagagatgctgtatggtttaattcatgcacggtacatcttaaccagcaagggtctagctgcaatggtaatgcttgttttaatctatgcgtcaagatacaattctttcttaccttatgtactgaattactacattttcatttgttgcagttagaaaagttcaagaactatgattttggcagatgccctcgagtatactgctgtggtcaaccctgtcttccagcagggcaatcagacattcctaggtcaagcacagtgaaggtgttttgtccaaaatgtgaagatttacactatccaaggtccaagtaccaaggcagtatcctttggatttttcactctgctcaaattattagtactccctccgatctgaattaattgatgcagcgtctgtacaatgtaaaatggacgatgtatagaggctgcgtcaattaattctgatcggagggagtaatatactaatatatccatccatcgtaagagtattaacttgcgtggcccattaaacaaggtcttcacaaattatgatttcttatgtaactctgatgctgtttcatgggaattttagctaagatgtttccatccacctgtgataagattcttgcgtgtcccatcccatgcggtttcttatcaaattgtcatttccacattatgtaaaatctgaaaccactgaaatatacagccctataatagtgaaatgccattatggtttctccatcgcaacagtacagtgatataaccgtatagaaaaaggtttaggtagtaatttgtggtccacactaaacatgattcaactttatttcatcctgcattttggaccttttagtagtttgtgttacatattcatacatgtgtttggtggttacggttatactagaaccaaatattgtaccgctgttgcatccttgactcagccaagacattgatggagcatactttggtacgacatttcctcatctcttcttgatgacatacccacacctgaagccacagaagccatcacagcaatacgtcccaagggtttttggcttcaaacttcacaagcagtcatgacgaacttttaccagtgggcgctatttgtttttacctagtgttagagatggaacaccttggcctgaaaatgttgtggcctcttgattcgagtactcgcgagctcgaatcttgctgtaaactgctgagcaactgtatctgttttgttgtatgactattgtgtaatctaggtttcctcacttgatcaatctatgcacatttgcatgttagcagggtgtttttctgtgtccgaatcggatacatctagatcacaccccagcaagttagtttcctatgtttttatgctattattttctagagtttttcgttctcctaccttctcggctagtaaggcacttaaattttcatcgcgtatttgtgtttgacgttgcctcccaggttttggtatatggtatgcgcaagttcagagttttactgttagtatgttgctggagcttaacgttgaaaaaatatacacaaattgtaaagtaatttcctcctagttttttttaatgtgcagagaacaatgtactccttttggtcagcaacaagtaatatgaatcagagggagtattctttcccctgcaaatagaaagaaagaaaaatggtattgtaggagatttagttctttcccctgcaaatagaaagaaagaaaattggtcctgtaggagaatttacgtgttattaatatatatatataatatggcagagccgtggctgatgttggataagacggtgatgagccaacatgttggcccattttctccatgattagttgaagcaaacacacggtatcactgtcatactaaaacccaggaccaattgcctattctgattcatgtacttggacaccttacattaaataatcctgtggcacacgtaggatgctatccatcatgccatggatgtggataagcatggcgatgttgcctgctcatcaacatcacagcagcacgacgaacacgtcctcttttgacccggtgacgccccaattctcaacaaattatcatccccattatatattatgatgaactcacatgttcatcctgaatcatcaaatggaatattcctaggtcacgactctgtcctgaactcaactcatgagtaatgtaataagagcaactccaacgggtcgacccaaatggacggcgtttttgtccgctttttattcgtttgggtcggacgcccgcccgccgtccgccctcttataaatttaggtcggcagtgcgcgcaacgggccaacccatttcatgaccgcacacgctttagatcatgctagcggccatgccgtcgctctgtttttggcgctccagcggacgggaaaggttcgcgcgcgcggacgaaaagcggcctagcgcatgctggttttggcgctctagcgtgcaggaaaggttcgcgcgcgcgccgcggccggcgctcgttataagaaggagctccctccacactctgtctgtcgcccactcgtctcgccccctctcactagcctcgtcgcctctgcgccagcatgtcgatgcgccgcctgggcgcttcggattttcgcggagtctgcgagcgtcgctccagcgccttctccttcgagatctggtttggcgagaaacgcctcatccttggcaccttcgacaccgcagaggaggcggcccgcgcgcacgacgtagcggcgtggcgcctcccgaggccttgtcgggatatgaattttcccgacgtgtcgagccaacgggcgtaggatctcacgcctctcctgcggcttttcaccgacgaggatcatcgtgtccaccagaggcggcagcgtcgcctcgccatcaccgagatggacgtggaagccatggtggtgtggcgcgaatgcttcccgcaggacatcatcgacgagcgccagttctataagcaaaggaggacgaagagggacgcaagtaggacagaacgagctgcctatcgggaggacaagcgttcgcggaagctggccactcaattgaaactgaagctacgagaaacgtcgggttgggacttcgaagacgagcagcatgctgacacctacattcaaacgtcggagaaggacattaccgagtcggagtcggaaagcgacgagtagttggtcttttcttttatctgtgtacgctagaactatctatgtatccatttttatcggaaaaaatggccggcggcgtcggcgacgaagcaggcgggcgagggtgtgttgtttgatgtgaagaggtcaatgtgccaccgaccagcgggcccgatgaggaaagaggacgagcgcgcgcgtctgtcttgtgtctgcaccgacgcaaatccggctaaaaaatgggtcgggaatgggtcggcaggtggatgaaagcggacgcgcgcccgtttgggtcgacgcgttgggccgacttttctgtccacgccgatccaaacagacggtcacggacgaaatggggtcgccccattggagttgctctaatataccgattcgcaaaataagattactcgggccaatgtaagctcacacacacaaaagaaaaatctttactgattcacccaacttagctgtagctccgccagtgcatacaccacacaaccaaacttagaaacgatcttctcttgaagctccaaccactagtgcagccacacactgtggcactaaacttaggaacgacgacctcgtcgtgagctcgtcgcagcacttggaccaccacagccgcgtggtagtactgtgcgctgctaaacttagaaacagcattcattcatcttggcactcaatagctcacccggggcaccgcaagtgtgtggtactgtcgccgctaaacttaggagcggtttcgtcgtgagctcgtaacttgagccaccaaaaccatgtgtaccccgcccctaaacttagaagcggtgctcgttacttcactcgtcgcatgccgttcacttgtaacttgaagcgccgtggccacgtgtgctgcgcaaacttagaaactatcttgtcgtgagctcctaacttcagccactttggctcaaccatgtatgcactagagcgcaccacgaggaccacacacgcggatgtagggtgatactaggaagtactacttcctctatttcaaaacgtagtgcctaaagttaaacaatgcaaactctgatcaagttttatgaagaaaaggatctacaaacagaataccagctgcatattgattaggtagatatatcatgagatttattttcatgttgtatatattttttatattctagatatggataatttcttctatgaacttagtcaaagtttgcttattttgactttaatttcgttttttttaatacacactatgtcatactagaacggaggaagtgataaactgatcgaccaaaatggccgagggctcgcttttgcagggaaatattctaatttcaggccggtgaagacgaccccatccatttccctctgtattccttgcacacacacatgacagagccagatcgaggcaaggcgaaggcgcacggatatgggagacctccaaggtgctggccctatcggcctgccacttttcatccttccatgtgcaggcaaacagacccgcaaaacctaacaggcacaccatgagtaaaagtgtgagtttgctagctaaatgcatgcatcccacgctaagtcgctgtgatctggtagcccctgtgtctgaagtgtttcttagtttcttttcggtcggtgagaaaagtgtactactccctccgatccaaattaattggcataattctaacacaacttcacatttcttgtattagcatctttgtcttctatgacattcgtgcataagaaacttcacacatacgacgcgtgcattgtcctcatgtttttaaagtacgttacttccaatgcactaatactgttgggcttcatttagtattctcaataattgcaatttgattagggttgtaaacaagggacaaacgctgcatacatcagcaaaaagagcggaaaggagttctgacaagactatcactgcctaacaagtgctccctctataaagaaatataaaagtgtttagatctctttactgatctagacactcttattgtttagtgagggagattttacaccagatgattacttatgtgtagcaacaagggaatacatatttttgttaggttagcctgtgaattaagtgcgcatattaatttaatagtgtataaaatatagttgttagatcaataaaaatttatttggcggtaaacacttgtttgataatcatttattggcttatcaaagaaaaaaattatttggtacataaattagtggtggggcagttgaggcggaaaaaaaaatcagaacagagatagagtgggcatatgtcatgttggacgaagaacgagcggacaactgaatgttacatacttctgagagatgaaacaaaccggcaagagtagaacaaattcaaaactgggccatttcaccactcatcttattggtcaaaattgcataacccctagcgaaaaatattcttggagtattcatatcgaatgttgtttaaccactttaaggccacgcaaaaaaaaatcccactttaagaattgtttttcttcctgcaaaaaaataacttcacaaattgtttaaagcacataaattttagaaggaaaacaaaatccacacagtgcagacgaggtccaagggcccgctttgacccgcaaaactttaaatacaatagtacaattccgtcccatgttcaaaaaacatgcctaaatttatatttaattactcaaagacaaatataggaatctttgttagtgtagatagactttttctttgaaatattgtacgaaaaatatctccccaaaccgcaaagcccataaataaaagcacacaaagatagaaaaacaaaataaggaaaacagccgcacaatttgagattccgttgaactgaatcgagggaccatccttcgcaaaaataaaataatgaaactgtttttttttccggctaggatctatttggttcaatggaatgttagggcatctatgattcgcatgatttgtacaatacatgaatagggaaaaacatgagaatgaagttgcatgtcaacttggatactatcacactattagtatagctgtttgattgtgcccaaggaaacgtagaagtattcatatgaggttagagcggatgaaaatttccatatgatatgtagtatatatgaatcctaagataaattttctatgagctgtaatcctatgaaaattataagaattagaattctccaagatccgtttaggattcctttgaatcaaagaggccctcacagttaaaagtcttcatgaaccatacgtagatgccttaattatcacactgaggttgtatgcattaattacactaatcacccgttgcacggggaagaaggaaggaaggaaggaggagaagaaaacgagcgagaagacagggagcaagagagaa

>TaCBL1_1BL_3900149

tgatcaatagttatgctaaacttatgttttagttttagttttttattttgcgggggaacctatgttttagttgctgtgatattcattctattgatccacctcctgccttgtgaattctgattgggtgttgtctgactccgtgcacttgctgttctgttcatgcagtcagcgtcagcgaagttgaggcgttgttcgagctgttcaagagcattagtggctcagtgatcgatgacgggctgatcaacaaggtaccactttcttacgaacgtgtgattccttaccaggttgacatatacagacatcaaataagcacccgagacctgtttttgcgtactttttgtcggtaccattgaaacgaaatctcacattgctatgctgaaattacattccgcatgtaccgaccctcttacaactgagcaacttatgcggcaattcccttttttggtgattacatttagccatcaatagttctactttctctaacatagtaagtgaacatggattcttgttgtatatgtcgccaatcgtttattcttgatgtcaactcaatgagcccgtttgataaatagctacaaattagagccgttattgtagcataataactatgacacggtgaagccttagtagagctcgggagcacatgctcctggctctaaataagtatcttatctcttccaaaaaaatattaatagtttcacacatttaggtgagtattaggtagattttgagttttatgcaaagttttccattctcgtggcctgtgcaaaatatagaataaaaattatagcacaaaaatgccttttggctccttattattgtcttttccatacagactataagtaacacatcttctcacggagaacttgaaagaacttgttcctaagataaccgtgtcacaaactcacaatgtgtcctgcatcagatattattgcaagttatatgaacatcatgtagctcaaggttttagccgttatcttgcatctcacgttatcgtagtctagctaaacttcatggctcgtttcctggcttacttcttacataagctaactgcttacttcgcatgttcataatgcaaaatttgatttgatcattcatggtagagctctagataccttgggagaatgccttccttgtccagcgtaattataaatgtatattatggtcatatatattatagaatttattatttgttgtcatgttaaccggtacatgtctgcaattatttgtaccacagtgaactatcatcttgatgtcattgggtactatattcactggcaactgaaactatgagtaatttgtattcatatatatatgcaggaagagttccagcttgcattgttcaaaaacacaaggaaggaaaatcttttcgctaatcgggtatacatccccttgtcaactattctcatttgcgatattacatataatatgctggggctcaatgttatcaatacaattgacttatatgaaattgaccttgtagatattcgacctttttgatgtcaagaaaaggggcgtcattgattttggcgactttgttcgagcgttaaatgtattccatccaaattttccagtggaagagaaaattgattgtaagttgttacgtttctgtcttgtcttcagtttatagacaccattctctctctttctgaaggttttgagctgcttgatacttacagtttccttcaagctatatgatatggatggtacagggtttattgaacggaaggaggtaatgtctggccttcttggtttgtgcctctgcgcatgttttttgcttctgaaattttctgaattagtcgtagatacaaaagaaatgcattgctttgtgactgaaaaatccagtagcgtgtgcacttatgtactgctgaagcaaaaaaaattaaaaaaattaaaaagacaaggagatgacctcgtctcagcttccattctagaaacataactcatatcttccaacgattgcggcacatgcttcacaaatcaaacattgctagtacgacaatgactgcgattggcttttttctgacttaaggaagcaggactgacctaacactgaaagtgtagctaccaattcagagcatctacatggaccagcatgcagcagcacacctaacaatatttgctagaccctaaaaccaggcagctccacagatgctctgtaccatctggaagccaagccaaaccaaaaaagaacaggctgctgaactccgtagaaaccaagccatagcatataacaacaacaaatatttatgagtttttattggctcaaagtgagaattctgatgctcaccaagaaccaaatgataacgatagacaatcgaaccaattatcatcccaaaagttgatattggtgccatcaccattttgtgtgtataaagtattaaagtataggagccttcgtttatcagttaaaatttctataatcctcttgtacaggtaaagcagatgttgattgctcttctaggcgaatcagagatgagattatctgacgagattgttgagacaatcctggacaaggtattggatacttttttaagttgttagttgcttaagttgttgataattcttgtgtccagtattgaagtgtagaacgataatgactggttaatggtgacagaccttttcagatgctgatacgaatcaggatggtaaaatagacagaacagaatgggagaattttgtgtccaggaatccttccttgttgaagataatgactctttcgtacctcaagtgagtatgacgtaacatatgaattcagaagcattcgccgcaactacgtttatgtgtccccatgtggctatttccagagaaatcaatttctagagcttaactgagactaattacttttggctgctgagctccagtgaatgttttgaaaatttcaaaagacgcatttacgactttcaagcaaatctttaaaacaatcccacatgttcacgtggcaattcatagtgtattcatacatgcacatgtgggatttgtttttattttctgaaacctagaaatgcacttctcaagaatttcaaattaaagggatgactggaggtcatgagccaaatatccgcacccagacaagcacattctagttacatgtggatgtggtagtaattgtttatgtgacctccactactattactgttatcttgaactagcatagtaagtagttcaccttaaatgccatggcttcttgtaccctttccatatgatgtttgatgacagtgtggtgcctgggttctttccatgaacatttgatacgaagcaacggaaattcttgctaatcatttgtgttggcatttcagggacataacgaccacgtttcccagctttgtgtttcactcggaggttgatgatattgttacatgacggtgatcctcacaaagatgtacaggtaacagaaggaaagaacctatcatcagaatatctgcaacgctgcaacatgcttgatgctggctaatgttgctcgagagtcaagagatagtttttttctgggtggtgttcattacaccgagagttagtcttgttaaatatttatgtatgtttgtgagcttcaatttgcaagtacttggcacaacttattacttgtcgagaatatggctgcttaataaactgttgttcaacctgactaatataattcgagcgtgcttcatgaatctactaccactgccagaaaaaaaaaaagcaaaacagcaccacaaagaaatgtatccttagaaactccaactcgcacgagtctgtctcggtcgttgcagacagaaaagtcggcccaacgcgccgactcaaacggacgcgtgtccgctttttgtctggctggcgacccattcctggcccaatttgggcctcatttgcattggcacggacgcgtgcgtactcctcccccaggcctgccagtcggtggcacattggccattctcttccacccccatctacaacaagccctcgcccgccccaccctgtcgtcaccgccgcccagtttcggtgcccttgccagcagtctgcacccacacacctcctaca

>*TaCBL1*_1DL_1737972

tcagcgtcagcgaagttgaggcgttgttcgagctgttcaagagcattagtggctcagtgatcgatgacgggctgatcaacaaggaagagttccagcttgcattgttcaaaaacacaaggaaggaaaatcttttcgctaatcggatattcgacctttttgatgtcaagaaaaggggcgtcattgattttggcgactttgttcgagcgttaaatgtattccatccaaattttccagtggaagagaaaattgatttttccttcaagctatatgatatggatggtacagggtttattgaacggaaggaggtaaagcagatgttgattgctcttctaggcgaatcagagatgagattatctgacgagattgttgagacaatcctggacaagaccttttcagatgctgatacgaatcaggatggtaaaatagacagaacagaatgggagaattttgtgtccaggaatccttccttgttgaagataatgactctttcgtacctcaaggacataacgaccacgtttcccagctttgtgtttcactcggaggttgatgatattgttacatgacggtgatcctcacaaagatgtacaggtaacagaaggaaagaacctatcatcagaatatctgcaacgctgcaacatgcttgatgctggctaatgttgctcgagagtcaagagatagtttttttctgggtggtgttcatta

>*TaCBL1*_1DL_464815

atggggtgcatccagtccacgccgaagcggaggcagcaccctgccggctacgaggaccccgtccacctcgcctcccagaccgccttcagcgtcagcgaagttgaggcgttgttcgagctgttcaagagcattagtggctcagtgatcgatgacgggctgatcaacaaggaagagttccagcttgcattgttcaaaaacacaaggaaggaaaatcttttcgctaatcggatattcgaccttttcgatgtcaagaaaaggggtgtcattgattttggcgactttgttcgagctttaaatgtattccatccaaattttccagtggaagagaaaattgatttttccttcaagctatatgatatggatggtacagggtttattgaacggaaggaggtaa

>*TaCBL2*_5AS_1464783

gttcgctaaaatattgctgcaactcaaaatctgcaagctctgctctaggtactttctgtgaccaaccaggaggaaatagtagaatgttgtcgaaaagaaggtttggttatgcatgcttgccgatgaagtttgcacttggtgaactgcggtagttttgtgacacggacgtgtgtgtagacatcctacatttgtataaatgtatttacaggtataccaagacaagctctatatggaggacatgaagttcagttctttcaagaaagttgccagatcacttgggtatgacgagaatggcatctccatagtgttatttcattcgggggcaattttttgttcgttagaaacactgcagaaggggaagctacatgttgatatgtaaaattcccttcgtgacttgtttaggaatgacggcgctacacctagctagcgccatggacggctcaaggtcgcttgtgatgagatcatcaatcttgaaggacaggttgagaatatggacctcttggacgacaatgtcgttgttgttgtaaaacatggtgttggtgtttcagttgaaataggcaagggcagtcgtcccgaggctgcccttattcaggcttccagatcgagcggcacctttatcgattgaagtcatatcctctgacatgagtaacttattcccccttatttctatcttatatcttcaactgtattgacatcaaaacagtgtcaggattatagacatgtatcgtcgttattaccaatcagatcagtggtacaagtctttgttccttgatgccgagtgtttagccacaagaggcgttagaagagccccgacggaatctatcgagccttctgtctgtgtagtctgcaacttgacaaggttaaatgtgagattcttttcattctgaataatatctatggcaacatcttacatttaatccaattagcctttccttttaatttcccagtattctagggaaagcaaacggagctcatggaactgatgcaaccgtaatcacacacttcgacggtactgttgcttataagcatgccttttgctgatatgatagtttgcattaactttgtaatctttaattgcatgccagcagttctctgactatgctttaaatgagagcagtttacatcgctaggatatgggcatcagagattaagaagaaaatgggcttgacagaatttgttatatacatctttcatgttccctctgtagagcgcccttgggtagttacttcgttttgatgttttaggtgctatccttattgcttcactgtcaaaatataattattgccttggccaatagaataagatgatgacttgtggaacatacattttaataggtcactacagaccagaatcagcggcggagccaacgtataagcgttgggttcacttgaacccaacaaattttgcttcgtacgtgcgcgtagggacatgtttgactaacctgaacccagtaaaaaattctagctgaccccatcaaatatcgtgagcagctaagcaaagggcccaaaagcccacaggcacttgatgcccaggcggccagccggccaggcctaaactatgattactcacgtactctgtactcgtgttttgttgtttattatacatgcacacacacgtttccctaaaaaaatatacacacgcacgtaggcaaacacttcgtcgagtcgtcgtctgttagttttccgattcgcatctctcctcgtctgtttgtgttccttgcgtctagatcgacagggacaacaattttcaccaaaatttgaggtaattaggttacattgccacatctcgtcatcttctgtctattctatctagcctaatagcctagtataccatagtgatttttttgccctagtttttgctatatatttttcaaatttgttatgtggggaaagatatattttctacaattagtaatgtggtagtgttcaatctttttaaggcgatgaaagattggaaggggtcgctctaaatgtgagtgggtttacaagcttcgtttgcttgatggtatttattttaaacttgtcaatctaagttgtcattgtttcaatatctcgggaagatgagaagctaaatctggagctttaaagttatttgacactactctattatttataatatgtaaaaatgtgcttttataattttatggaactggtatgccttgcactcttcagtttttaatgcagcaccaaatgttcgtcatagtgtttatgggtgttataattttgttgcaactagactttaatcttcataatttagtgtcaagttctcataaaagagtgaatgggtgaacccaatggccaaattttctggctccgcccctgaccagaatacgagtgatatctatataacataaaaagttacccagggaatcggactaattggttttgcttgcttagtgcgtgaggggtgcactggttgtaatcgcccctaggggcatcctgctgtactttgtcttcttccttatttcagtgaagtgctgctccgacagcttttcgtcaaaaaaaagtgctaagcaccagattcccatttcttgataatggcaagagtctctggaattggattgtggtgaagagacttattatccgtcgatacatgttggttcagagagatcttgtgttgtggtatgtataagtatgccatttttatccctcttaagctgagcctgaagattaaacttatatgggatcgctgttatagaactttggtcaagttttaaaataaattcattcacaaaaatgcacaccgaatgattgaagacatgctaccatgctgcttgcaggatgtctttgatactaaagagaaagaacatggcataaggccatcgatgatgtgctgcacaaaatacatgctctccatccgtcaaggaagcaagtcgtaaatcaacatggtcaagttgtagtgacgtgttaaaggcaaaaaactttcacacattttctgttccattcacaattatttgaagggctgcttatcggttttaaatcttgaagcttgcacggtcactaatatgaccatgctattttagagtgataaccttcatctcaggcagtaatgaaacatgaaatgccagttgactaacagtaataaacaccaagaatactggttgatgttaatgggaattaggaggttatatttagacatgattagcgatgattcattttatgctctggtggaatgggtcaagccggtttgcgtggtgagtattgtccgttacatgttttaagaccttcggcgtgaaattttcatactaactacatatattgtttgtgcaggatgaatatgagttcagaaaacagtttttattatacttgctaaaacattatggaaatgaagctaaagacaacttttcatatattgtgaaagaatttcttaaacgcatcatctagatattaataattttataatagatgtttagttagaacatcactcagtttgttacatggacatgccattaatgtttctttttggcaatttacattgcaaaaatgtactttaattagtactaattctatttaaattgtgtttgtgttatattgtctgtatgtgtgaaatttaacatgcattatctttataaaactcttataaactattttaagagtccgtggcaacgcacgggcattgtactagttctatttatcccacacaatcaatccttgatttaattagcagcccgccccattctatccactatgtggcccagtacagactggctatatgaaagtgtgtgtgcatgcagacttaaatgggtttttcgacttgctaaaaaaatatgtttttcgaggctaactttaacctaatgttagagcaataatatatgacatgcaacttacataaagataccgtcaaattcgtatgtgaaaggagctaatgatataattttcacattatacatctcatattgaagagaagcttgtccaacatcgtctgagatggtttaggcatattcagcgcaagcctccagaagctccggtgcatagtggacggctaaagcgtgcagagaatgtcaagaaagggcggggtagaccgaatttgacatgagagaagtcagttaagagagacctgaaggattggagtatcactaaagagctagctatggacagaggtgcgtggaagcttgctatccatgtgccagagccatgagttggttgcgagatcttatgggtttcacctctagcctatcccaacttgtttggaactaaaggctttgttgtttttgttgttgtagtcaaaagcggtcttaaaaaccgcattagacctatagatggaaggagggagtacagaacatagcatggccactgccacatatgattcagacggatataaaagttaggagtaagaatattacctcaagtgtccgtgtacaatacttacgcgtaataaaacaactcaatctcgaagccgatctaaataggaaaacccagacatgggtgtcccttgatcttgtagatctatttttaaatctcagccatcaatctttatgattaacataaataaaatcaatacatataagagtgacagaaggagaactatgaaaacctccgtcctaatattaaggtaaagataaaatgcgactcgacctaacagaggaatattttgtaaccctaatttctgtaagatgaacaaagggttgatcaatactccctccatcccataatgcgagacgttttttttactctagtgtagtgtcaaaaaacatcttacattatgtgaatcatgcgaggaaacgggtaaacaaaatccaatccattttctgctccactttactatagattgcaaagcaatggacaaacttcccagcaaaagataacaaacaatgtcaaggctgtttacatttttgtacacacagcagaattcccagaggattggacctgatcgctaaccagccaaaaaccaaagaggggaagaggagaaactgggaacttcagataagagataacagcttacccaaaggctcccaacaatactaaattcttcaaatgaaagaagcaaagttatttgacaacaatttgcgttctcaaattcaacatcctccagagcactgaggcctctagctctctgccgatccaaagttccaaacagtccgatgccaaaactttacaaaattaccgcaagcttatcttctgatgttccgattcgaaataactgcaagaatttcaggtatcgtcaacctgagaatggaagacgaagcttggaaatgttgtggtgatgtccctgctcacgatggcaaagttagcttgagaattatataaagaatagaggtactggaaatatgccatatactgctcacgtctaggaatctgtcttgacaaaaataaaagacataaaacctaactattttctattaaagacttgtagccttctggctcccgacatgcagtggtttgcttgaggctgaagcactaactgagcacacatgtttgaaataatttggtgcagttcatcaacagtgcatgaattcccaaatacttgagccaacatgctttactcaatagcactatttgcatgtacacagctgaagtttgattaaccacatctcaagtatgtgactctccagacaacactgtttcacattacaagatatttcaatggtaatttggcactaaatcgagaatgaaatttctatcaatctttattcaagagcagcaggtaactggcaaacataagcgaagagcactgtgttgaggtgtggaagatacataaaaagaaccaacatatagaaaaaactaaatttcttgagtaaaatcaaagaaaaactttgtcacttgacactaactgagaaataaattcttatccacgagcatcagtaaaccgttaaaagtaaatgcactgagtagaagatacacataaaaagaaccatcatatatagaaaaacaaaaggaaatatcttacttgaggtactggagagtcatgttcttcagtaaagatggatgtcgaagaaccagattgtgccactcttccctgtcaatttttccatcatgctttgtgtctgcctcctcaaacgtcttatcatgaggggaaaaacaacaaatttaacatgggttacaatcatcatgcaacaggaggaaaacagggaatgaggaatacttgtattgaaaaagaaagaaaggaaaacagtattcgtgcaatttaataatagaaatgctatgtggaactgtcatatacaagaatcgagaattgcagcactacataatacaagttagagaattaatgcctaatacgtgctataatcaagggttaacaaaacaatcatggacaactgtaacacaccttatcaattatgttctctataatttcatcagacagattcattcctgactccgcaagtgtggcaacaaccatctgcttaacctgataaccaaatatatgcgttaagatgaagcaaacagagaaagctcaaccaaacgattgacacactgcacaaacttagcctcaagaatacaatagcagttcttcaaagctaaacgatataattcctgaatctcaacaataaggccaactgcaatatgttaactccctccgtcccaaaatataagatcattttttagacactatgatagtctaaaaaatgatcttatattttgggacggagggagtaggaattaaacctcaatctgttgggaacaagactgataattcaccttttttttaaatagtaacaagcctgattgaactactggtctcttaactttagcataaccattaaccagcactacccagatactgcctctacattcagagaattgtttcataactttgtaaaccctcattcaatcagtgattatcagaaagcctgggccttcctatcatcagttgtgtgggcatacatggtcaccataccataactactcaagataactgacaaggccatcaagttcaaaagacattaacttgaataaagcataatttcatgtatcagagacaatacaggaacattttgaattatttacaatgctgtacaagctaatatatttagccaacatgctagacctgacgggttacaacactggaaatagtatcaatacctcctgtctctcaatgaagccttgttgcttaagatcatatagctggaatgaaactgtaaaagatgacatgaacttcagatcttccagctcataaagaggatgtgacaaggcaggagcataacataaaatattaagatccttaaattcctctatttaaccacaaacacaagtacagtatccagaaattgatgattaaactagggttggtgagaatccttgcatacatttatatttgaagaggtatcaccattatgatgatacaatctagagtctggatataattgcatatttgggaaccaaattctctagctacaagataaatgaactcacagtcaattttctcttcaactgaagcatttgggtgaaacactgagagtgcacgagcaaactcttcaaaccctaaaattccattgtgctttgtgtcgaacaaatcgaatacctgcacaggcagggaaaatatcaagaacacaagaacataatgtgtaaaaaaggtaaactatttgtcaattcatgcagcaatagtaaaagaaatgatattcacgcagctgttccttttacatataatgacataatatggtcctcaacatttttggcttccaagttctcaaaaggaaactctccagctaaaattgatagatccatcttggtaagttgatgaccttccatacagaggaacacagccatgtaatttgaataaacttcaagtatcaaaaaatagctatggactatgcatttagcaccagtgtctagtaaccatgaagaactagccaaatgttggataaccacatcacaactcaaatatagaagcacactgagttttgacaagagatacacaaggcattcctgctgataataaaataaactacagggcaaatcagtcaagtgatgcacatatgcagcttccagttagaaataacacaatactgcatgaacatgctgaatcaaggaaaaaactaactctggtgcatgcgatgcacgtcacacttttaggagctatgatatgtcatcaaactggtacattatgtaagaagagtatcatctcaaagttattgctatattcttttattcaagaaacttgacatataacagtattgctttaaagcaagcatgcatgataacacatgctcagggtaaaagagcgagaagcctacaaatttctgagctgggcaaaaaaggaagcatgtggagtaacagacaggacaaagggcaccccatgggccacagaaggtgaatacttgaaatattcactgtctgatcaaattgtcttgaccactggttgaaggtagctaacaggtagacatagactgcacatgattaaccaatgatggacattatttagaaaattgtctagcgaaaatacaagaaagaaagggtagatcctatttcagactcctgaaaatcatatagtaatgctcgcgaaaccatgccatgggctgaatcggatactccgaatccatatcggaatttgtatgttattagctctatccaaaatcctgccaaatcggagtcggactccgagaaacgcataaatggctgagtaatactatgatgcatgaatatctgaagcgagggtcagcggaattctgctaactcatactatttccacaaaataattacgagtcaattagtgcatgtgttgcatcaataagtattgaacagagttcaattgccgatacaacatcaacaatcaagaacccctgaatctcattgattttgaccaggtccctaaaaatagcaagtgcatggcagtaatactgataccaaatatgcaacgacaacatcatgacaagcagcagtacacagcaatcaactgcgttgaactaaagcggacttacacgatcagcgaagaggctctccttcttgctcgtcttgaagagcgctaattggaactcctcctgcacagtgcgaataaatcagaacgacagagccacagcacccggcggatgattcacaatacctaaatacaatcctcagaacataacctacagtgatacgcaatctacagtgcttacatacacagaattgagctgcgaaacaagcactcgtcagcaaccacctaccttgttaatcagcccgtcatcgatcacggcgctgcttatcttcttgaagagttcgtacagcgcctcgacctcgctcacgctaactgcgccacgcacagaaatccaacccagttcagcgctcaattcagttcagttcccccgccacgaaattgaagcagaaaacaacggcgaccaaaagcgcagagggacgtacagacggtctcccgcgcgaggacctgggggtcctcgagcccccgcggctgcttgagctcgaggtcgcagcacttgaag

>*TaCBL2*_5BS_2281375

cggcctcggaatgggcggccgcgatgactagcgggggtcgcgcggcggcgttgttggcggaggcagcggagcgcataccatggtgcagtgtctcgacggcgtgaagcacctcctcgcggtcctcttcaagtgctgcgacctcgagctcaagcagccgcgggggctcgaggacccccaggtcctcgcgcgggagaccgtctgtacgtccctcttcgcttttcgtcgcccttgttttctgctcatccgtagcgggggaattgaactgaactgggtcggatttctgtgcgtggcgcagttagcgtgagcgaggtcgaggcgctgtacgagctcttcaagaagataagcagcgccgtgatcgatgacgggctgattaacaaggtaggtggttgctgacgagtgcttgtttcgcaactcaattctgtgtgtaagcactgtagattgcatatcactgtagtaggttttgttctgaggattgtatttaggtattgtgaatcgtcccgtgggtgctgcggttctgatttgttcgcactgtgcaggaggagttccaattagcgctcttcaagacgagcaagaaggagagcctcttcgctgatcgtgtaagtccgctttagttcatattgttgtgtactgctgcttgtcatgatgttgtcgttgcatatttggtatcagtattactaccatgcacttgctatttttagggacctggtcaaaatcgatgagattcaggggttcttgattgttgacgttgcatccacaatttaactctgttcaatacttatcgatgcaacacatgcagtaattgactcgtaattattttgtggaaatagtatgagttagcagaattccgccgaccctcgcttcagatattcatgcatcacagtattactcagccatttatgcctttctcggagtccgactccgattcggcaagatttttggatagagctaataacatacggattccgatatggattcggagtatccgattcagcccatgacatggtttcacgagtattactatatgattttcagtagtctgaaataggatttaccctttctgtcttgtatttccgctggacaattttctaaataatgtccatcattggttaatcatgtgaggtctatgtctacctgttagctaccttcaaccagtggtcaagacaatttgatcagacagtgaatatttcaagtattcaccttctgtggcccatgtgatgccctttgtcctgtgtgttacttcccacatgcttccttttttgcccagctcagaaatttgcaggcttctcgcacttttaccctgagcacgtgttatcatacatgcttgctttaaagcaatactattagagcatctccaatagcatgtgtatatttggatgtctatatattcatatagacaatggtctaaaaagattccctcatatacacatccagttttgcatcagaacgtctatatacacggaccatgacaggtgggccattgctgggagaggaaaaaatcatgactacagctgagtttagacaacgccttcacattgtccaggcgtggacattgtcgaggtcgatttagaggatgtgtatatttggacgaccatatagacaagctgttggacgtctgttttgggctcacgtcgtggaaaacgagtatagacatccatatagacaagctattggagatgctcttatatgtcaagtttcttgaataaaagaatatagcaataactttgagatgatactcttcttacataatgtaccagtttgatgacaatatcatagctcctgaaagtgtgtcgtgcattgcatgcatcagagttagtttttttccttgattcagcatgttcatgcagtattgtgttatttctaattggaagctgcatatgtgcatctcttgactgatttgccctgtagtttactttattatcagcaggaatgccttgtatatctcttgtcaaaactcagtgtgcttctatatttgagttgtgatgaggttatccaacatttggctagttcttcatggttcctagacactggtgctaaatgcatagtgcatagctattttttgatactcgaagtttattcaaattaaatggctgtgttcctctgtatggaaggtcatcaacttaccaagatggatctatcaattttagccggagagtttcctttagagaacttggaagccaaaaatgttgaggaccatattatatcatgatatgtaaaagaaacagctgcgtgaatatcatctcttttactatttgctgcatgaattgacaaatagtttaccttttttacacattatgttcttgtgttcttgatattttccctgcctgtgcaggtattcgatttgttcgacacaaagcacaatggaattttagggtttgaagagtttgctcgtgcactctcagtgtttcacccaaatgcttcagttgaagagaaaattgactgtgagttcatttatcttgtagctagaaaatgtggttctgcaaattatatccagactctagattgtatcatcataatggtgatacctcttcaaatataaatgtatgccaggattctcacctaccctagtctaatcatcaatttctggatactgtacttgtgtttgtggttaaatagaggaatttaaggatcttaatattttatgttatgctcctgcctcgtcacatcctctttatgagctggaagatctgaagttcatgtcatcttttacagtttcattccagctatatgatcttaagcaacaaggcttcattgagagacaagaggtattgatactatttccagtgttgtaatccgtcaggtctagcatgttggctaaatatattagcttgtacagcattgtaaataattcagaatgttcctgtattgtctctgatacatgaaattatgctttattcaagttaatgtcctttgaagttgatggccttgtcagttactcttgagtagttatgaatggtgaccatgtatgcccacacaactgatgaaaggaaggcccaggctttctgataatcactgattgaatgagggtttacaaagttatgaaacaattctctgaatgtagaggcagtatctgggtagtgctggttaatggttatgctaaagttaagagaccagtagttcaatcaggcttcttactatttaaaaaaaattaggtgaattatcagtcttgttcccaacagattgaggtttaattcctactccctccgtcccaaaatataagatcattttttacactacccaaaatataagatcattttttacactgtcatagtgtaaaaaatgatcttatattttgggacggagggagttaacatattgcagtgggccttattgttgagattcaggaattatatcgtttagctttgaagaactgctattgtattcttgaggctaagtttgtgcagtgcgtcaatcgtttggttgagctttctctgtttgcttcatcttaacgcatatatttggttatcaggttaagcagatggttgttgccacacttgcggagtcaggaatgaatctgtctgatgaaattatagagaacataattgataaggtgtgttacagttgccgatgattgttttgttaacccttgattatagcacgtattaggcattaattctctaacttgtattatgtagtgctgtaattctcaattcttgtatatgacagttccacatagcatttctattattaaatcgcatgaatactgttttcctttcttttctttcttttcaatacaagtattcctcattccctgttttcctcctgttgcatgatgattgtaacccatgttaaatttgttgttttttcccctcatgataagacgtttgaggaggcagacacaaagcatgatggaaaaattgacagggaagagtggcacaatctggttcttcgacatccatctttactgaagaacatgactctccagtacctcaagtaagatatttccttttgtttttctatatatgatggttctttttatgtgcatcttctactcagtgcatttgctttaacggtttactgatgctcgtggataagaatttatttctgcagttagtgtcaagttacaaagtttttctttgattttactcaagatatttagttttttctatatgttggttctttttatgtatcttccacacctcaacacagtgctcttcgctttatgtttcccagttacctgctgctcttgaataaagactggtagaaatttcattctcgatttagtgccaaattaccattgaaatatcttgtaatgtgaaacagtgttgtctggagagtcacatacttgagatgtggttaatcaaacttcagctgtgtacatgcaaatagtgctattgagtaaagcgtgctggctcaagtatttgggaattcatgcactgttgatgagctacaccaaattatttcaaacatgtgtgctcagttagtgcttcagcctcaagccaaccacttcatgtcaggagccagaaggctacaagtctttaatagaaaatagttaggttttatgtcttttatgtttgtcagatagattcctagacgtgagcagtatatgacatatttctagtacctctgttcttttatatatttctcaagctaactttgccattgtgagcagggacatcaccacaacatttccaagcttcgtcttccattctcaggtcgacgatacctgaaattcttgcagttctttcgaatcggaacatcagaagataagcttgcggtaattttgtaaagttttggcatcggactgtttggaactttggatcggcagagagctagaggcctcggtgctctggaggatgttgaatttgagaacgcaaattgttgtcaaataactttgcttctttcatttgaagaatttagtattgttgggagcctttgggcaagctgttatctcttatctgaagttcccattttctcctctccccctttttggttttttgcctggttagcgatcaggtccaatcctctgggaattctgctttgtgtacaaaaatggaaacagccttgacattgtttgttatcttttgctgcgaagtttctccatcgctttgcatctatagtaaagtggagtagaaaatggattggattttgtttacccgtttccctcacatgataaatactcccttcgtctcataatgtaagaagttttttgacactacactagagtaaaaaaaacgtctcacattatgggacggagggagtactgatcaaccctttgttcatcttacagaaattagggttacaaaatattcctctgctaggtcgagtcgcatcttttctattacagaaattatgggactcaggcttttatagttctccttctgtcacccttttatatccattgattttatgttaaccataaaaattgatggctgaaatttaaaaatcttcgagattgagttgttttattacgcgaaagtattgcacatggacacttgaggtaatattcttactgctaatttttatgcctgtctaaattacactccatccgttctaaaatagatgaccaacttagtactaactttatgctaaagttagtataaagttgaatcatttattttggaacggtgggagtacgtggcagtggccacgctatgttttgtaagtcgcacgcacgcgcacattcgtacggccagcccgtactgggccatatagtggatagaatgggagcgacttgctaattaaatcaaggattgattgtgtgggataaatagtagaatacaaggagatggaggacggacggcaaatcaatcgagctctctcaataagggtcaaggagcatgaggagatggaggagtcgatcaaaaatcaggccagcagccaagctttggtagacaacgaacggcgaggcgtgccgacgatcgatgccctcaacgaaagaagcaggaaaggcgctggtgccaatggtcgatgcccttaacgaaaaagagcagcaataactggccgctggcctcacaaggacgtcgtctgcctctcgtgcgcagtggtgtccaaggccgtcgtcttcctgcttgggatactgttggtgaggtgccgagcccgtcggccttcacggttgtgttggccacaccaacgagaacgatatctgttagaatgatattgtgaagggagtccatcacccgtctacgtgcgatcgagaatgacacggtgttcgttggccgaggccagcccgagctgcatggcactgttgtcatgctactcaaacgaacagacaatgcaaatgaacctacacagctaatgttgtcattttatgccccacaaccctgcaaggatgaggacgttgatattttctttcagactaccccttccgtttccccctcatcgggaagtgcacgccatggacactgtgaaagcacaagtgctccctggattattttgttaattaatatcaacatatctcttgttggactaatatctttatctagtatatttcagaaaattcaacaatggcgtggcatggacaaaaggatgtggaaccccttcaaaatgctaaggacaaagattggcaaaagctcaagactcttcatttctattttagtgatccaagatcacattgagttcataggaaaagccaatactattaaaaggggacgaagtgttgcttaatggtctacttgctcaaagtgcttagtgatatgctccaaagccctcaaccactttctcaatttcaaatttgtccaaaacctaaagtcaaactcggccccaccgatttgatctatccggcgccacagagttcctttgacatagccactgccagaaaccctaatcaattcgatctcaccgataggatctcggtctcaccgagatggccttgcaaactctctgttgtccgttgcaattatttcggtctcaccgaaataagcaatcggtcccaccgagtttgcctgacgaactctctgtttgctcattgctgaaatcggtcccaccgagttcatgcaatcggtcaaactgagatgaggttttgccttaaccctagcacatcggtcccaccaagttgatcttgttggtcccaccaagatttctaacgttcacatttttgaactgagtcggtctcaccgagttcacctattcagtctgaccgagttgggtcaaatatgtgtaacagttagattttgtgtgtaggctatatatacccctccacccccttctctattctagaaagagccatcagaacatgcctacacttcctctactcatttttgagagagaacaacctactcatgtgttgagactaagacattccaatccaactacaagaatcttgatctctagccttccccaagttgctttccactcgaatcatctttccaccatagccaaatctgtgagagagagttgagtgttggagactatcatttgaagcacaagagcaaggagttcatcatcaacacactatctattaccttttggagggtggtgtctcctagattggttaggtatcacttgggagcctccgtcaagattgtggagttgaaccaaggagtttgtatgggcaaggagatcgcctacttcgtgaagatctacccgagtgaggcaagtccttcgtgggcgatggccatggtgggatagacaaggttgcttctttgtggacccttcgtgggtggagccctccatggactcgcgcaaccgttacccttcatgggttgaagtctccaccaacgtggatgtacgatagcaccacctatcggaaccatgccaaaaatcttcgtgtctccaattacatttgcacactccaatcccatccctttactttcttgcaacctgcatgctttactttccgctgctcatatactcttgtcatgcttgcttaaaatgtattgtgaatgcttaaacttctgctaaaactccacctcaacttaaagaaattaaaaacttctacttttgcttgttgagggtctaatcaccccgctctagacacctcttctcgatcctttcaattggtatcagagcatcggtctccattgctttggtttaatcaccattggaggaagatggatgagtcgacgattaggagtattagacgtagagtgcctatgcttgatggagagttctatagtgcttggaaaaatgagatgcttgagattttcaacgaatatcacttgaacaagtatattactagcccttgtgtgcctcctattgatcctttgcatcctacccctgatgagtctcttgacatgatctgcaatcttagaactattaatcttatcataagaggattgcctagaaatttgattgtatgtttgcccactcttgaatgtgcttacaccatatggagatatcttgaggaatgctttccaaattattccttgaaaaacttaaatgatattcttcaaaagtccatagcttttcataaaatgaaacctagtgaccctaaatttgatgattgtctatttgagcttcatggccttatgcgtgccaaaggagatgttggagtcattagtagcatcatttctcaagtcattagaattcataaagatgcacattgccatggtcatacatctaatgaatcactctctctaggtgttgatcaatcacaagatgatgttgaacatggatactatgatgaggatgatgatagtgactttgatctcgatgagtctatgagacactttggtcttatggcgggaggaaaggaatgtgttcttgatagtggatgtactaatcatatgaccagagataaggacatgttctgtgagcttgctgaaaacaacgaccctcaaaagtatgtcactttttggtgataactcaaagggtaaggtggttggccttggtaaggtggccatctcacatgacagctccatcaaaatgttatgctcgttgaatctcttggctataatttacttttcatatctagactagccgattttggttttaatgtcttatttactgaaatagattgccaagtgttttgaagagataatcataatatggtctttaccggtatacgtagaggtgatctatacactgttgatttcacaaaaaaggctcaacctagaacttgcttaattgctaaatcttctaaaggttggttgtggcatagaaggtccggtcacgtgggcatgcgaaatcttgataagcttattaaaggtgatcatatccttggtgttaaacatgttatatttgaccaggatagactttgcagtgcttgtcaagcaggaaaacaagttggaggaagtcatcccgtgaaaaacattatgaccacgagaagaccgcttgagctacttcatatggatctttttggtcctaatgcctacaagagtctcggtggtaactcgttcagtttggtcattgttgatgatttttcaagatttacgtgggtgttctgtcttgatgataaatcgcaggtccaaaagatcttcaagaacttcgttaggaaggcccaaaatcaatttgaagtgaagatcaagaaggttcgaagcgacaatagaacggagttcaagaacgccaatgtggatacctttcttgacgaagaagggatttcacatgagttctcggctacgtacacacctcaacaaaatggagttgttgagaggaagaaccggacactcatcaatatggcaagaacaatgcttgatgaatacaagacgccaaaacacttttgggcagaagtggttgagacagcttgtcatgcaactaatcgactatatcttcataagcttctcggcaagacggcatacgagcttctcactggtaacaaactccaagttgcatacttttgagtatttggctcaaagtgctacattcttgataagtatcatcgttcgaaatttgctcctaaatctcatgaaggtttcctactcggttatggttcaaactctcacacttaccgtgtctacaaaaatttcacccgaaaggttgaagagacgatagatgtgaagtttgatgaatctaacggctcgcaagtagagcaattgccaattgatgtaggacataaagacccctaggaagcaattcaagacttgtttattggcaagattcgcccaacgaaggtgaaggagagtacctcgtctgttcaagtggaagcctcaacgtcgcaccaaggtgaaccacaagttgacttggaggcatccacaagtggaacacaccaagatgatggaaacgaggaagtacaacaagatgaacctcatcgacctccttctccacctccacaagagaacaatgacaccaacaatgaagaggaagaacaagatgaagaagaggatgttgataacccacaagtgtaggggatcgcaatagctttcgagggtaaagtattcaacccaaatttattgattcgacacaaggggagccaaagaatattcttgagtattagcagttgagttgtcaattcaaccacacctggataacttagtatctgcagcaaagtatttagtagcaaagcaatatgataataaaggtaacgatagcaaaagtaatgtttttgggttttgtagtgattgtaacaagagcaacagaaaagtaaataagcgaaacacaatatgtgaaaaactcataggcattggatcagtgatggataattatgtcggatgcgattcctcatgtaatagctataacatagggtgacacagaactagctccaattcatcaatgtaatgtaggcatgtattccgtttatagtcatacgtgcttatggaaaagaacttgcatgacatcttttgtcctaccctcccgtggcagcgggg

>*TaCBL2*_5DS_2741958

tctttcacgacactccaccaccagcagtagcaacctctcgcccgcgcacgcgatgtacacgaagtagagggtttgaaccttgcggcccagcacgagaaaagcaatctggacgaaggtactcacgcgcaaaacaatttccacgaagagaaatcgcaacacagaggttttctaaagatccctccaaaacttgatacgggtgtctaccctgaaaaacacatcgtgtctatttcatataaaaataacctgtcgtatcatggctttatatagtagccgaccaaaccttgacctcgtacacgcacaaaagaaaaataaaataactcctaaacaaatctagactctcattaaagatacttggcctagtaattttggtacaagcgctcccgacttgtgcacatcaggagggccccacccgacatctcttaggcttaaaaactcccaaatacgtgtagtccttttgttgatcttttccttgcgtggcagaaataaaaacagaaaggatctactcctagtagttttaatccttacgtagcatgcaagattggataggtaagcttgacatgtagctttattggactccttcccatacacgaattgatcctgttccggtgtccatgacaatatagataaatattcaaagtgaacaccatctggactccaaccgaaaattggtggtggtagctgaacttgttttatatctgctcgcacatcaacgactgcttggtcatcagtacgaatgcatgcattgttagacatcttctgcatacctagtgcatggttcatggtctttctcacgcctaaacattgcaaaacagtaacatggtctgtagtacttggtagtaaatcttctgcatcattgtaagtaatggacaaatacgctggtgtataagctgcatattttggcacaacctcgacaacaatagggtcgacaactacattggtatttggtatagtcatggacctatcaaatattatgctgcttacctaaattgtacacacctctgagaaaatatggaaggttgaatggtgtcaagggattgagtaaaacaagggtaactgcagttatgttatcataaagtgtagctagtgaaatggaaaccaagttattggagtacatttgatttaccttttctagggaacacactgactttaaatatgcaatgtaatacttgtatctgaaatgtcctacaatgcgttggagagattgttggatgctccaggaaataacgagaaaggcaaagaaagctacatttgtggtggtggccaaataccccaccctacatttgaatcttctagcttgcggcctggaaacatgcctgagatatatgtggtgtcatgaaagttttgcctcgttgtcatgtttatttatgtttgcccttcatttgttctagaaggtggatatccataacagttggacctgtacattaatattctaaatatttttccttttaagaggctttatcatggtcaacttctcgtacatatatttagaataaacaatttcaaagcttataaaaaaaatacctgggtcttacttatttgattctttgttgaaatatctctgaaacaactcctagcaggctatttctttttgaaaagtcggtcctagcagactattcactatcatgtttaagattctataatctagccctgttttatgtattatcctgtattcatggtcagcttctcatgtgtagatttggtataaacaattttcaatctcgttaaatacgtgcggggctttcagtttttttgcttccttaatagaacgcttctgaagaaaacccctagctgattgccgtatttatttccagcctcttgatgccagcggttacgcaacggttgagttcggcagcccagacctgctcctccgttacaggagaaaggaaacccatcctgatccctgtatgtgggccttccagaatctccacgcgacgcccgtggagaaagccgcatggcgcataccctgtggcgacgaagcgcacacgggatttgtatctagcctcaccttcatttcagctctggtttgctccatgtccatagtcttagcagcttccctaactacttgatacaagtactggaaacgcaggacacggtgcattgatatagtcggcagaaaatgactttttttgtcgcaagacttgtgcacatcatttcgcgcgtacttttacgcgtcatttgcaaaaaaagaaaagaaggatgaaaattcaatttcgtattcgggacgtgacccagatctgaaattctgaagccacactgttccgtgataccatcatctttgaggaagccaggtagaggcagttgaagagcatcagctgcatgattggtgtgaccttgtcctgttgtcgctgcacgcttataactaactgacgggcagaagaaggggggtcgtcctggaagaagaataagatgtgttgctgcaacttgcaggttgaagcgagcacttgtgctgagtggatccgctggacagggcctccatgaagcctctctgcacttcacctgatcacctcccttattttactagttgtgccgagggcttcttctattatatatttatgtatataaagggatcttgtatgactttccaagcaattttccgataaaatttttgatctttaatatttgaaactctcacatttcgcctaccgggcacttttcacctaattccagtttttcttaaaaaaaaacaagtgattccctatattgtttcttttgtgcaatcaatggtaccctatcatgtaggatgaactgacagcgatgaaatcacttatttttcatcatgttcttatgtcattagaatcccgcgaatcaaagagtccacgagatacgtaaccctgcgaatcagatggtccacacgatgtagaatcatcgaatcaaagatgtccacacaagatatgaattatgatatgcgcagtggtgggtctcggtcacggcagtaatctccacgtcggtcaggttcatcgtccgcctcgctgagtgctgcttcgcctagtgaatgatgtcgctgttattctacatacatggccacggccatggatagggacctcgctgacacggcacacccgtggcctgtgtggactagatcgactgtccatttgccgcggccagtatcaacttcgacccatatttggtgtcgacagcgcaaggcgtgaagatccaaaagactagcgaggcggattgatgtgaccgtggttccaagtcatcaatctcaagaatatcaaacatgtaataagataatccgaaagcttctgaagagccgctcaggttgttgaacagtagtactacatcgttggcatattatcgacgagaaggaccgagacaacaccagaatgaaatctattacttcctcttttcgcaaacaaaaaagaatctactactgcctccattccaaattaagtgacatggttttagttggaacgaaggaactaacatactctatccgtccgaaaatacttgtccgaggaatgaatgtatctagatgtatttttattctagatacatccatttgtatccattttaatgacaagtatttccggacggagttagtactaagctctcttgatttgaagttattttttctttttcacgggactctagatttggagcctcctcatgcattgagggttgtgggagatgctatgaccccattcgtaaggatgtgttgttgggccagccatttttttttctccagagaaggtattgcctgattttttattttcttcctatatgtgtttttcaccggtttaaatagtttttcttttgggttttcttgcttttatctttcttgtttcttttcttgtttattttttcttctctttttattcttacatttaattttgtttctttcttttctatgcttttcgctggttgtaatggtttttctattggttatttcctgctttttttctttttatctttctatttttttttctcatgataagtcgaaaactatggaaaattttgaattcatgaatattttgaaagactttgaacattttaaaatttaagaagttttttcaaatttgtgaacatgcatggttgaaatccacaaaaaatttgaactcctgacgatttttctaaaattcctattttaaattttaaaaatattaaaatccaagattttttgaaacacgtaaacttctttgaataaaaaagtgaaaaacaaaactaggcgagctagtggagcagggagctgagtgagagcttcatgggcgacccaggtaagcgtgatagcagcaactccatggtttcaatatggaataaaatctcccaaagggcacacacaaccaccacttgctgttgtcgaggctgaaatatgtaaggactctataaccacacacgtaacaacatcactctcatctcagaataaaatattgtctccaatagtcccaacacaatatatgaatgaaaaaaacatcaccataaaataattaaaaccaagtcaaggtagataaaccaaaacgaaatagctggccgcaatgacccgctagagagaaaagagttaatcttacgagtgaagaacggggcggcaaagcgcacattaacctctagtcatacatagaactctgtgctttgtggcgacaatataagatgtgctaattctcatctagatgagaattaacaaagtcacgtctaacttatacattatttaatcaattaaacagaaaaaagaaaaaataactccacgaatctccatgtaaaatcaaatgatgtaaacgttagacgtggctttgtaactctcatctagatgagagctagtcacactcatatgtacttaatataacatggaatacatagacatgaagaaaaagttaaaaaaaggagagaaaaaagtctatgtatcatctataaaaaactagacagtatgtaaaataagtgatatattcactctatctcttttcaaaagtttccattgccttatcaacaaaatcagctgtcacctcttataggcagacaagcaaacggcagaccgcgacggaaaataaaaaataaagcgagtggcacggggagaagcgtaaagtatcccggggaacagttggcctccctccctcgctcgctcgctcgctctccccctcctcttccttccggctcggctcctctgctgcgtgcggtggctcgccgcggtgggccccaccccctgcgaacccgcagcccaatcatgagcccgaaacggcaacgaggcccggacggggggtcggcgcaccccaaacgaacctcccactcctgatcccaccccagcgaaacaaaacgacggggcggagacgagacgcgtcccgtccgcgcacgctactcgattgattgcttccttccgcccgctgctgctgctgccgctgccgccagagctcccctctcccccctccccctcctcgaatcccccggtgccctagccagcccgccgcccccgacgacgaccggcggcgagcgagagggcggatcgtgccccacgcgccgctccttcccctcccggagggagaccctgccgaggtacgcccgcccgctccccccttgctgctccagagtccagacccgcacgagcgatcggatcttcgcgggtacggcgctgtctgaccaggtggtggtgctggtgcaggatcgagaccggccggccgcggaatgggcggccgcgatgactagcgggggtcgcgcggcggcgttgttggcggaggcagcggagcgcataccatggtgcagtgtctcgacggcgtgaagcacctcctcgcggtcctcttcaagtgctgcgacctcgagctcaagcagccgcgggggctcgaggacccccaggtcctcgcgcgggagaccgtctgtacgtccctcttcgcttttggtcgcccttgttttctgcttcattcgtagcgggggaactgaattgaattgaactggatttctgtgcgtggcgcagttagcgtgagcgaggtcgaggcgctgtacgagctcttcaagaagataagcagcgccgtgatcgatgacgggctgattaacaaggtaggtggttgctgacgagtgcttgtttcgcaactcaattttgtgtataggcactgtagattgcataccactgtactaggttatgctctgaggattgtatttaggtattgtgaatcatccggcgggtgctgcggttctgatttactcgcattgtgcaggaggagttccaattagcgctcttcaagacgagcaagaaggagagcctcttcgctgatcgtgtaagtccgctttagttcaacccagttgattgctgtgtactgctgcttgtcatgatgttgtcgttgcatatttggtatcagtattactaccacgcacttgctatttttagggacctggtcaaaatcgatgagattcaggggttctcagattgttggtgttgcatccgcaattgaactctgtcaatacttatcgatgcaacacatgcagtaattgactcgtaattattttgtggaaatagtatgagttagcagaattccgctgaccctcgcttcagatattcatgcatcacagtattactcagccatttatgcgtttctcggagtccgactccgattcggcaagatttttggatagagctaataacatacggattccgatatggattcggagtatacgattcagcccatggcatggtttcacgagcattactatatgattttcggtagtctgaaataggatttaccctttgtcttgtatttccgctagacaattttctaaataatgtccatcattggttaatcatgtgaggtctatgtctacctgttagctaccttcaaccagtggtcaagacaatttgatcagacagtgaatatttcaagtattcaccttctgtggcccatgcggtgccctttgtcctgtgtgttacttcccacatgcttccttttttgcccagctcagaaatttgtaggcttctcgcacttttaccctgagcacgtgttatcatacatgcttgctttaaatcaatactattatatgtcaagtttcttgaataaaagaatatagcaataactttgagatgatactcttcttacataatgtaccagtttgatgacaatatcatagctcctaaacgtgtgacgtgcattgcatgcaccagagttagtttttttccttgattcagcatgttcatgcagtattgtgttatttttaattggaagctgcatatgtgcatctcttgactgatttgccctgtagtttattttattatcagcaggaatgccttgtatatctcttgtcaaaactcagtgtgcttctatatttgagttgtgatgtggttatccaacatttggctagttcttcatggttcctagacactggtgctaaatgcatagtccatagctattttttgatactcgaagtttattcaaattaaatggctgtgttcctctgtatggaaggtcatcaacttaccaagatgaatctatcaattttagccggagagtttccttttgagaacttggaagccaaaaatgttgaggaccatattatatcattatatgtaaaagaaacagctgcgtgaatatcatttcttttactatttgctgcatgaattgacaaatagtttaccttttttacacattatgttcttgtgttcttgatattttccctgcctgtgcaggtattcgatttgttcgacacaaagcacaatggaattttagggtttgaagagtttgctcgtgcactctcagtgtttcacccaaatgcttcagttgaagagaaaattgactgtgagttcatttatcttgtagctagaaaatttggttctgcaaattatatccagactctagattgtatcatcataatggtgatacctcttcaaatataaatgtatgcaaggattctcaccaaccctagtctaatcatcaatttctggatactgtacttgtgtttgtggtcaaatagaggaatttaaggatcttaatattttatgttatgctcctgcctcgtcacatcctctttatgagctggaagatctgaagttcatgtcatcttttacagtttcattccagctatatgatcttaagcaacaaggcttcattgagagacaagaggtattgatactatttccagtgttgtaatccgtcaggtctagcatgttggctaaatatattagcttgtacagcattgtaaataattcagaatgttcctgtattgtctctgatacatgaaattatgctttattcaagttaatgtcttttgaacttgatggccttgtcagttatcctgagtagttatggtatggtgaccatgtatgcccacacaactgatgaaaggaaggcccaggctttctgataatcactgattgaatgagggtttacaaagttatgaaacaattctctgaatgtagaggcagtatctgggtagtgctggttaatggttatgctaaagttaagagaccagtagttcaatcaggcttgttactatttaaaaaaaaggtgaattatcagtcttgttcccaacagattgaggtttaattcctactccctccgtcccaaaatataagatcatcttttacactacccaaaatataagatcatcttttacactacccaaaatataagatcattttttacactatcatagtgtcaaaatgatcttatattttggtacggagggagttaacatattgcagtgggccttattgttgagattcaggaattatatcgtttagctttgaagaactgctattgtattcttgaggctaagtttgtgcagtgcgtcaatcgtttggttgagctttctctgtttgcttcatcttaacgcatacatttggttatcaggttaagcagatggttgttgccacacttgcggagtcaggaatgaatctgtctgatgaaattatagagaacataattgataaggtgtgttacagttgcccatgattgttttgttaacccttgattatagcacgtattaggcattaattctctaacttgtattatgtagtgccgtaattctcaattcttgtatattacagttccgcatagtatttctattattaaatcgcatgaatactgttttcctttcttttctttctttttcaatacaagtattcctcattccctgttttcctcctgttgcatgatgatcgtaacccatgttaaatttgttgttttttccctcatgataagacgtttgaggaggcagacacaaagcatgatggaaaaattgacagggaa

>*TaCBL3*_4BS_4897686

ttcaaatttgaaattagttggtatgagtaaatacagtgatgcaagagcaaaggagtatgaaccatagctacggaacctgacccgagaacaattcttcttctgttttaagcgttgacttggggttcagagtggtggtcctcgtgctttgggcactgcaattgccttactaactgctcatgtttgggtgctctatggtggagatggtgttgtgtcaactggaactgggttactatctgctggggttggggttagaaggggtgtagctggttctctgtccaactgggtaggggtacaatctgcgtgagcctgtgttatgtgtggtggggctcgttcttgggcaagtacaatagtggttctatctgcatcaacaggtagcaggatcttttttgaagaccgtgtttttactcccacatatactgccatcaccccctccaattcaaatggctgataaacaagaaaagtaattgaatatacaaacattgtaagatagacatgtgcaatggatagtagggaagaaaacaaggaatgacataattcaaattgaagggagagaggagggggagggggagagggatggaaaatgtggctgctctgccttaacagtagtgcgggtcagcaaacggcgctattgctaaagagttagcagtagcgcggtcctgcaagcagcggtttgctaagcagggcccgccatgtggcatagtacaaacttagttgtagcgttgtccctgagaaatacgctactgctatttttttagcagtagcgcgacaccttatggtcgtgctgctagtatacgtagcctgttggcgaagttgtggttgtcatagtcgtagcgcctttggggagggctgcgctattgctaaggaaatagcagtagcgcgtttgagctccccgcgctactgtaaataacagtagcactttgtacagaaccgcgctattgataagcctccacgtataaggttttccctagtactgtaagtttccaaggcccacgacggtgaccatccaaacttccgtatttgaggctagacaataccaattcacgattccagggtcgaatacaaacatcgaaatacagcatgagacatatggcaagatatactaagttttcttcggaatgcaatggaaatgatggattctccacaacagttgagttttttctctttgaaaaggaggccgaaacgctcggactctgtgtatccaattctcatgtttttgtgtaaccatggtgcgtattcggctagatttaatggagtcatcttattcattttccacatctcgtggactgtgcctaggtgatcttttgtcaccattcctttttctctttgttgttgatggttcaccccccatgttgcatgatgcggtaaattcacaacagattgtgttgatgtaggtgtgcgggcgagtgtcgaggatttcacatattgacatatacactttacatacaacattttattcttccaaaccaaagcaagaaactcttgttgtgaataaaatttggatacatatgaatcggcaaaatcaccagcttataaatccaagaaaatgttccatatgcttttgtgagttattaacaaatgaggttagacgagaatacgagaaattttgcaagttgagaagctagaatttgaagctaagtatttgggcctgccaacactcgaggagagaatgctcaaaaggaagttccaaattatacatggcaagctaagcaaaagtttaggctatagggggaaatttacaatgcacatggtacaacttgaagatctagaatgaccattcctcccgttggcgcaactgaattatgtccaccattctactagatttctaggaaaaatctagtatgtacataataaaattacacatgtgaaacttgcctctatcaatggaggatatgagtgattagtgattgttgacatggtaggttgattccttaattctcatattgcaatttaccacaatcgaacctaaaaaaagggaagtagttaatcaactactattagtgtggtgcaagaagccaatggaacatagcggcaaacaattagtgatggagctggctcgcacttgggaaccaccactttgaagattggttgaaactaaatacctatagctcatataacatgttgacaatactataagagcaggtatgatcttgcaggataataatgaaaatgataatataacaacttattagatggttgcgtagttgtcctgatgctcttgaaatgtagttagtagctcgcgaaaagggattcatccggccctccaatggtcactaaagccaattatgctcttgatggatggcagggaggtcttgtccatgattcccatagaagaaagtcatgttcatatatgcttcatgagttgtcgatattagagcacacccaaacaaaactagggaagttagaatttgtaagattgatagagattagaatagaaggggctcactagcttttgttgcttaaattcaagactctcttccaataaagatgaatattgcagttgatagatgttgaatggatcaacattgtctaatcaatatattcccagacctcatgcagtgggggtgggggtgggtcatggggttaaatatcatgaaaaataaagtgaagtgttttttcttccaaaaaatagcggtttggctaccacggcttacttgtctgtaatatacaagatgagacacgaagaattgattgtttcgttgtggcaaattagattgtctcaacaaagcataacaagagtgttaagtgtgcgaagagtcttttcgccaaaaaaaattatgagaagagttagtgtatctacttgcaacacatggtgttcttgtatttatgcatcattatgaattatccagttcaaaatattcagatacgctacttgatatctctctttttcataaatgtgaacatgtagttattgtttcattacaagtaaattgcacacatatttatattctagttatgagagttgatcttaatatgttcactattcttttgtagtctttcttttctcaacccacggaggtgggaccataaaaacccatataaacactacaacaatagacccttttagaatccttctcattttatcgtcttgcatcactcaatcatcccgtgccttctcccatcactaatctcatccgcgccaattaccaaaccccatccaaattgacccacaaggctcttgagtaaactaatttcatcacaaaataaaaaacttgcacacccgtccaataaacccttccggaatcaccctttttttatcatgtctcgcccctcaataattttgtgccttctcccatctcttatctcatctgtgcaactgtcaaacccaatttaagttggtgtatgcggctcttgagtaaaccaaattgtaccttgtttcccatggttccgtggcgtagcactgtcaacttgctagtgtatatcgcaaaataacataaaaacttgtacgcccttcctaacagacccttcccgaatcactcttcctttatcgtcgcgcgtcccccaatcatcccgtgtcttctcacatcgctaatcttatctgtgccaaccggccagacccccaatcaagttgacgtactaggctctcgagtaaaccaaattctaccttgttcccacggttctgtgccgtagcactcgcaacttgctggtgtatatcgcaaaataaaatgagaaacttgctggtgtgcatgaacctgtaaacacaacgtggccgcacgtcctcccttggatcgaatagcaagggaggggattgcgccgacctagcttatcacgactcgccgaaagggaaattcaaaatcgcaaacgcaacaacggaaacacgcacacgcaaaaaagaaaataaataaatagtaagagaaatcctcgatccaatccgttccagcccataccaatcgtacaaccagccgacgactgcggcatcgacggcgacggcgtcgacaggaacccagcggcgaccccctccggccgcccctctgctccgcgccgcatcgggaaaccctgccgcgatcctccagccacgccgccccccgcgtgcccactcggctcgttcggccaaatccggtcctccgcccgcggggtcccgatttgcggctagtcctgcggattgcgcggcgccaaccctggagagagagacgggcgttgacggacggttgcggcgcacagtggtgccaccatgttgcactgcctggagggggtgaggcacctgtgcgcggtgctcctcaacctcaagtgttgcgacatcgacctcaagcagcccaagggcctggaggatcccgaagtcctcgccagggagaccgtgtgtatgtccacgccaccagctctctctttgctattaatagcgtctaagaatagcccaagccgtgattcactgtgttctgtttatcctctgatgtgtaatgtgatttgcgtgcagttagcgtcagtgaggtcgaagcgctgtacgagctgttcaagaagatcagcagtgctgtgatagatgacgggctgatcaacaaggttagtggatcaacacccatttcgtcggaagtaaaattaatgtagtatatatgatgagtgatgaataacccgaattgtgattcttgtgcgtcgacatggtgcaggaggagtttcagttggcgcttttcaagaccagtaagaaggagagtttgtttgctgatcgtgtaagcccacattacacacatgtgaacacgaatttgctgtctgtacagtgaaaatgcctctctgttgttttctcttctttttggtacaattctaatgtcattactgtggagtcactctcactccccttttgggcatattatttggaccttggagagaaagaagcactgtttaacattctgtaggaatcatgagctatagatggcaaagggcgtctgcctaattgtagaatcgtgcaggatttttgggtgctcaagtacatgtatggttaatatgtaataagaaacagatgatttaaaaaagcttggaagcaaatgtactcaccactttctggggacacttaattgtcaatagaattgcaccagctttgctagctaggactggaagggtagttatctctttaattgaaagttatgatcatttatactaagaccataacactccacatcctaacagtttcttatgaaaaaagtccttcacaaaagctgatcacagtggcaacacatgccattggcatctcgctaccccttatatacaccctttagtcaggcattgcaattataacttaaacctgtattatatccttttagtaaatttgtgtgcataggtgaaacagtgaagtgggaatgatgttgatacttgatacataacatgatttctttcaatatatagagatacataacattttcctatttcttctaggtctttgatttgtttgacacaaaacacaacggaattctaggatttgaagaatttgcccgtgcgttatcagtatttcatccaagtgccgctcctgatgagaaaattgactgtaagttcagagaatcatgacatttgcatagttagcttaacttttaaatatggcagtagattagtgaaaagtttctgcttgttggcatagttaggttaacttttaaattgacagtagattagcttaacttttaaatatgtaccaacaaatctatcatatttaccatgataataactttaagaattcttgtaacgcccaagcttgtataatatgcacacatcaagtcagccctcttgtttgcatgatcctctgacatgtgtttatgtatttgattttgtgtggatgtgaatggattggatttaggcctaaactgtaagaaaacagaagattcatgatatccttgagttagtaggttcatacttgcacaaatcacattctgttattccatttttgagccttttggtgctgtctgaggatggtctgtcactctgtcttaaacaataaacaaacaaataaataagctgtaactctttctgcttgtttgatgtgatatatgtaatgatccaacttttttatttttttattttttggcatatgttaattcccttaagctcttttagtcttttatatctgattgttgtaagcggaaaaaaaatcttacacggtctaatccttgcagtctctttccagctttatgatctgaagcaacaaggtttcattgaaagacaagaggtattttgctaatcagaatgtgatatgtgaagttaacatgttgattaggatcaaggataccattcatctcattatttgtttctattattccatatttttttatgttcagttatctcccttaatcgcttaacgataaagtatcatcaatgaatccaactccagcctgcattgtgagtacttgttgactatgataagtttgacaggtggtggtaggtggccatttaccaccttacaccactttctggaaccctagttaggacaattcaaagtctgtgttcccttacctctattcaacttgatattgtaccaagtgtagccttgtagccctgatctgtatatctcctataggtagctttataaattagaactccccccaaaaaagagagcagaggcaatcttttgcataattgtggaactgatgttacttccttttagctcgtgtatgagcaagaatatacgttctgaagtcaaagaactgtggcatatagtctcctttgtgcaaaaaattgttatcttttcccttattttattccaatgatttttcttgtttggctattaggttaagcaaatggttgttgctacacttgctgaatcaggaatgaatctttcagacgaagtcatagagagcatcattgataaggtctggttaaattccttgatttcctatactactatggctatgcattgtaccatgtagaatctttctgttcaatcatgttaagcaccaattcccttttctgttctgaacacaagattttgtttatgttgtgctaaagtgaggccatcatttgttagcatgtcttcaccttgatagatcgaactagaaagcttgatccatatatttgacttacaatatttgattaaactcagttaaaaatatatttgatcaaatgctcgaatggaggtttgcaaatggctatgtgcctatagctatagctatcagtactgcctatttcattaatgctgttgagtaggtaatgaacttcagagctatgccattgtgccaaagcttgttattgcagtgcattggttgtaatattttctcaaaattatttccctcttgttataagactttcgaggaggcagacacaaaacatgatgggaaaattgacaaagaagaatggcgcaatctagtcctcagacatccctcattactgaagaacatgacactacaatatctcaagtaagatatgctaatgtatttgttgttaactctttaattttcatcagtttattatgctcaagtggattgaactttgcggggttcagaatgaataagagtatcaactgtccttgaattttattgccaaaatacttgtgcattatctcatagagccgagtcttcagcagcactgtcaggaaataaagaagcatgatattgttcagctataaagtgaaaaccagcaaacagaatatgaaacagtgattagttaaacgacagttttgtacacattggaggctggagagtatgctgaaccaacccattgttttcttctacattagaagttcatttacatcagttaactattagcgtatgtcgtattgtttctcaaggcaggtgtgactacgaagtgttaagtagtatttctgtttcaagttcaaccaagggagtgcgtagtagttccggcttcaaaggggatatactggaggcatgtgaatattgtttcattgcttcagttcagtctgtatcataacattactattgcatatgctgttattgccctactctatattcactaatcaggtcaacgtcgcagggacatcaccaccacatttccaagctttgtgttccattctcaggtcgacgatacataaagtcctgagagtgttttcacttcgatggaatatcagaagacggtctcactgctttgttaagctttccaattgccggccgatggttcagcggaagtcatcgtagtgttccgaagcatactgcatactgtgttcgattattccgctggtttatttgtgttcataatgttttttttctttagcttagggtggggtggtagtagatcagaatgttcaaagtttcttttgtttttgtacaaaaaaaaactggtttgaccctttagttaactgtgtatgtattctgtgtggggaactttcctactgtatattatggattgaactgaacggagcagaaatgcctttgctgataaagtttgtcacaatttattgttttctttaaggggatacttttttttcttattgctctgcttgatgttcagggacgcttataacatacacaaaagggtactccggtttcaggtctaacatcgatggaataactcagagttacatggaataggtactccaaaaattgatattattgaaatttatttgccgttttcccttgagaaaaaacatttttgtaggggaaagccacctggcgcactttaataactcaaatcaatgttacatcatctataatgtcctgaagcaaaatactgggacgatctcaccatcccaaacaaaaagttgaaatcaaatcaaaactatgtctagctataatttggacaatgagcaaaagatacaacacttatgtggaagaccttttgaaatcaatcagccaatatagcgtgtacgggctccgaatttcaatcacaccattgcaagcttgggcaacttccagtgaatctgattcaacgatcaccggttgacagccaaggccttccaccagttccaaaccttgccgaattgcaatcgcctctgcggttgttgcgtcatggagatgggatagtagccatgacgccccagcaagagcttcgctcctgctatctgagcattgcagctgctgagcccgagccattatcaaaataagcagtatcgacttctttttatacgagttaactggaggtttcttccacatcaccttctctggttccttaatttatactccctccattcggaaatacatgtcatcaaaatggacaagaagtgatttatctaaaactaaaatacgtatagatacattcccttttattcattttgatgacaagtatttccggacgaagggagtatgagttaataggaccgcttgagaaaaaaacattgttagcatcacacaaggggatgttgggaaacattgaccttgagtttaaaagattcttcggattcattataggaaccggaaaatattgcacaatttgctgcaattctcaaggagaatcaaagtccgtgtttcctgagcattcatcctttctcattggagaaagaatctggtagcattaataaaagaggcggtgacagagtgtgcgcaaagatggaaaattagcaaaagaaacagccaacaccctcggtgcaatcttttctcatgcatttccgcctaagcagcccttagggcaactccaacatggatcgccttcatcaaatgtacatgtacatatttggacgtggatacgaaaagtgggctgagcggagaccatccaatcaagcatgtccctcaaattcggactgtgggctatgactgggccaggcgggccttgtagagtaaaaaaaaaagagtatgacgcatgggctgtggcctgtgggaaacatatggttcaactgacagcggactgattcttaaaaaaaaaaacggacagcggaccaagcaggcgcttggacttccccaaacctcccattgattcggtggttggattttgggcgttttgccccaccccaccacatagctccagggccgttgaagggcctgtgcgaactgtgcgctcgcacagggcccccaaaattcaggggccccaaatagggttcatatactagtattagtctgaaaaatagttttgggccaacaagcactcctttttgggcttggttcgctcaatgcctcaatcccgtatgcgcatgcacactatctctcgtggatcaatctgactgatagacgaaacggcctgcctaatcccttcgctaattcccttcccatctgtgatcgcttccagccaaaggagcagaactaccgccgacccgccgtgccggacgccgccccgccggccacgttgccgaggtgccaccgaagagaaggccaaccggtgcggtgccgcccctacactccctttttctctagctatttttgatacaaaataacttgcaaagttgtacaacagtttgttttagaagaattttttgtctagttgttgaagttcagaagttgtcacagattcgagatattcaatagtcgcttgtccaagtccaaaattaaggttctatctttttctttataactatctatacaagttacaagcctataattcatggagtttatcagttcaaattcattgatattatttcatgttttgttcataaacagaaacaataatgcattcttgttctagaagaaactatgattccggggctttcaagcgtaagaagaaattaaaattataagaggatgctcaattattgataaatttgttgtgaaaagaccctgaactaattctgaaaatcaaacaccagaagctaatattgatgatggtcatggtaataatgcagaagaggctgaggttgcagctacaatcaaaggacatgcttattgatattgcaattgagtctatacaaggcctgctttcatttttcagtaagtatagagatactggattttcaaaagcactagaagctgcaagagaaattccacttgtgatggatattcatccagagttccgcaccaagagtaaactcaaaagaagaagacaatttgatgagggtgtagatgatgcatctattgtttcacaatctgcagaggagtcatttatggtcaactattttctgcaagtggtcgatcaagctatagttcacttaccaggagatttgaggaatatcaggggtatgaaaatacttttggtttcttatctacttcagataggctgcgatctttggatgataagagtttgttggctgcttgtgttaatcttgagaatgcacttaagagtagagaacagaaagacatcaatggagtagaactgttttgcgagttagttttcatccaggatttagttaagaattctaagggccctgttttcttaatagcctgcttataagggtggatattaggctagtcatagtggggagtaatttagactagtgtcatgcatatgacactagtctttgttactgccttcatagtgcaaagtaacatacaagtagtattatagagataggatttattacattatagactcatttttctttcggttgcgttatgttacagtagtaacatattatgttactccaaacacctctctcctcattaactatatgctacacaagcaaacttgttttggaatgtgctatattacttgctaagttactcccactatgacagtcttatagcacgagttgggcgcaaattactgcaattctccttacaaaacatgattttgcagacgaaaaaaaatacaaagcccgcacgttccctagaaattcacattctcattgatgaacgaattcgatagcttttgaaaaggcggcgaaggagcgtgcagagatggagaattagcaggtagtcaaaaaccttcggtgctatcctttctcatgcatttctgcgtaaggcagcacaagtgcagcgtgcacgtaccgagatgtatgcgcaggcgcgttgccagttaacgcagtgtatcggcgtaagagctaggttggccggtcgagaagcacagaaaggctagagattccgttccaagatgccaatataaataattaacaattatgctcgcgcctcacgtgtgcatgcatggactaactaagtgaccaaagtatcaagagtctcctgcgaaaacgtactagcatgcaatgcaagaagttgccaggctgctgcaacacaacgtgtcccagcttgcaaagctaccttgcatgcatgatcgaacgtaaaaaagactcatgatggaacaacagaagatgggagcaaaagccattgaacgtgtgtccatgacgtagtcaccggacctgggcgatctccaatggtcgcgtcaccgatcgtctcaccgattgctgtaacgacgaagaccatcccctcgccatttcccacgagtgtcgatctgtacacaccgatactgatacacgtgtagagggcaggaggtgtggttgctccgtcctatatatagtggatgcgtgggggcatgcttagctatcctattgcaccatcacgatctcatcgccagctacacacaaacacaagcactcagcgtagatcatatacagagagagttcgacgaccaaaactatttcgctgcaatcaagggcatcatggctttgctcaagtctctgtgcaccttcctcgtggcatttcttgtgatcttgtcggccggggattcccttgctgctgtggcggcgaggccggtacgactccacggtgccgacacggaggcgttgctcggtgaacgaggcggccggaggagggatgccgagacgttcagggcgtccaaaggcgctacgcgatcagatacttcgaacagcggcaacaacacgccaaagggccacccatgatcgccgatgtcgatcgatcggctatactctggtggcgcgcggtcgggtcgacacttatttgcgtatggtgattcagtatataatcggactatggatgcatgcatgctgactcatagcttttaagtaataatgcgcctcttttcgaaatgcatgctaactcatactatagcatttacatattgtatatgctcgtgtgttggtttagctttgtgtacgatgacacatgcatggacatgtacgtgaatatatacacaaatattagtagtacgcatgcgtgttccattattgcttgtacggtttgggattgagattactttgtaagatggacttacttgagttaagttctatgtaatattacccgaaaattcacagcctgtccattctttgttccggcagaaattggttaaaaatgaatgcaaatactcaacactctcattagacacaatatacaatttttatcctgtaccaatacttcggcggactgttcttgtacatccttcctcctatgcgtatggtgcatcccttatcctgttagatatctttttggccctttgttaaattttgcttccttttttgacccaaaaaacctttttcttcctttttcgacactcaaagttcattttgtttcctatgtgacactttcatccaagggtgttaaatggcatgtagaaagacaattttgcccctgatgatagtatgaacaggaaagaggaaaagaaggggtgtgaacgaccaaaaatatatgaggcggaacctacactcctgcctcttttcttcctcaccacggctcactcgggcgctaagccgagtgttttctggccatatgaagcgtgcacataggtagatgtacgaaagctaacttcctaagcaggcatgcatgcaccttgattgattctggagcacacgtaaagctagctagcttggccctagccaatgacacacacgtcagggcagctagtaagggcaaagccgacgggtgctaggtggacaatggcatgagcgtgtgcagcaatttaatcgtgtatatatgggtagtaactcgattcatgtaagtagtttagataattatttggactcgatggacacatttttcggaatgagtcaatgtgctcttggcttcttgcagattgcaattaaggcaatgcatgagcgtgtgcaacaattgaggcgcgaacacacattagtgcagaaccgggctttagtgccggttcgtgacggcctttagtgccggttggcgaaccggcactaaagagtggggactaaaggtccccggcctttagtaccggttcgtcacgaaccggcgctaaagtgccaccacgtggcacgagccagacccgtttgcgtggagagcattagtaccggttggtaacaccaaccgatactaaatgtttgggtgtttttttaatttttctttaattttgtgttttcagtttaatttagtgattgttttacattataatgagttgttaaatcattaggtgaaagcaccacggattagtttcgactgaatgcattattggatatctctatatatagctagctagctagagtatatgccatatccatattatacttgatcaactatataatatatacggatatgacatatacttgatcacttagctaggtaggattcatccagctgaaactaatctgcggtttctttcatcaaatgatataataattaactagctatgtaatcaccaccagcactactagcttaaagaagaaacattcacttgtataccagaagcaaaaatattatcgatcgagttcaacatgattgtcatgatattataagcgttcatatatatatataacaccacaaaagcaaatcacttaagttcagaacaaagaacacggacatgaaaggacaagtgctaattaagagcggcatgaagaactagctaaatcactcctgctagctactctctctggtaaaatagcatagaacatgtatagctctcctgattgatcatactggagcataccgatgaacctgtctcctaatcgtgggctgcgcttctcattgctgccccctagtacttctctgcgatcattaacaatttttctccaatctttcactattaagcattcatcgcttctagaaatcctgaatgtattcatgtgcgatgcatgatatcttggccgtaagctaacaattgacatctgacctttagtctcaatcccctgaggcacaactgtcattgggagtccctgttgaagaacattgtatagtacttaattaatatacttagcaatgaaagtttagcaaaaaaattatgtatg

>*TaCBL3*_4DS_2274776

tgcacgtaccacggccgacgtagcagttgcgccgatacactgctaagggcaactacaaagtgcatcacctattaacccgcaaatacttggataaaatgattcaatcacttttagttatccaatgtgggttactaaatttgtgtggacaggtcaagaccaaaaggtggagctatgtggtgccccccacccccagctcttcatcatttcgtttcacccctcaagtggttttgtctagctccgtcactggtcaagacgtccaaaatccgacaccgaaatgatgggaggtttggggaagtccaagcgttctgcttggtccattagggaggtcctagtcggcgccttaagcgccccgaagcgaactggcgcccgcaggagaactagtgggctggcacatagcgcacagaaattcgcaaaaaccaacaaaaaaacaacgtgcgacgctacgagtcgaactcaggccgctcctttcccacaagataatggataccactggaccattcgccacctacttattgatggcagcgcgcattacttaagaactattctagcggtggtatttgaaagaaaacaagatcactagtacgggaaaaggtgtcatcgattgaccggtctggccagttgaccgttgactgttcaaaaaatatttgcgttttctgaaaaattaggaattaaaaagatcgttcacaaaaaaagtccagcggttaaaaaaaaggttgacgaatttaaaagagaaaactcacaaaaatggaaaaagttcatcgatcttcaataaaagttcatcaattttggaaaaagttcattcggtttgaaaaaagttcatcaattttgcaaaaaagttcatcataattgaaaaaaaatcatcgaatctaaaaaaatgtgtatcgagtttgaaaaaagttcatcgaatctgaaagaaagttcaccaaatctgaaaataagttcatcgattttgaaaataaattcattgattttaaaaaaagttcatcgattttgaaaaaaagttcagcagatttgataaaaagccattgaattagaagtacgttcatcaaatttgtaaaaagttcatcgaatttgaaaaaagtacatcaaaatttaaaaagaaaaggaaaaagaaaaaggaaaatagaaaaaggaaatagcaaagtataaaagaatggaaacgttgcgtttgccacatcttgcttggtggcgtgttggtaaacatagcctcgtaacccgagggaggtctttggttcgattcaccagcagcgccttctttttgcgatttaagaaatgagaaggaaaattaaacgggccggcccagctggcgggaggtggtgtgcgctgcaggcgcccgttaacgaaaatgcacgttaaccggcgcctgcagcgcggaataggatttgccggtcattgtcagttgaaccacatgtttcccacagcagcccatgtgtcatactctctttttgttttttactctacaaggtccatctggcccaatcatagcccacagtccgaatttgagggacatgcatgattggatggtctccgctcggcccacttttcgtggatacgatcaaatatgtacatgtacatttgatgattcaaaataggtgatccatgttggagttgccccaagggctgcttaggcgaaaatgcatgagaaaagattgcaccgagggtgttggctgtttcttttgctaattttccatctctgcgcacactctgtcaccgcctcttttattaatgctaccagattctttcttcaatgagaaaaggtgaatgctcaggaaacacagactttgattttccttgagaattgcagcaaatttgtgcaatatttttcagttcctataacgaatccgaacaatctcctaaactcaaggtcaacctttctcaatatctctttgtgtgatgctaacaatgttttttctcaagcagtcctatcaactcatagaaattaaggaacttaaagaaccagagaaggtgatgtggaagaagcctcctgttaactcctataaattaaaagtcaatgctgcttatttcgatagtggctcgggctcggcggctgcaatacacagaaatagcagggcgcgaagctcttgctggggcgtcgtggctactattccgaatccatgacgcgacaactgcagaggcgattgcaattcggcgtggtttggaactggtggaaggccttggttgcaaaccgctgatcagattcaagttatccaagcttgcaatggtgcgattaaaatttggaaaccatacacgctatattggcgattgctttcaaaaggtcttccacataggtgttgtgtctttcgctcattgtccagatttctatctagacatagttttaatttaaattcgactttttatttgggattgtgatcctcccagtattttgcttcaggacattatagatgatgtaacattgatttgagttattaaagtgcgccaagtggctttcccctaaaaaacgatttttctcaagggaaaacgacaaataaatttcaataatatcaatttttggagtacatattccatgtaactctgagttattctatcgattgttagacctgaaaccggagtatccatttgtgtatgttaagcgtccctgaacatacacagacctccccacacagaatacatacacaggcattatcaacaaaggcatttctgctacgttcagttcagtccataatatacagtaggaaagttccccacacataatacatacacagttaactaaatggtcaaaccagttttttttgtacaaaaacgaaagaaactttgaacattctgatctactaccagcccaccctaagctaaagaagaagaaaaacattatgaacacgaagaaaccagcggaataatcgaatacagtatgcagtatgcttcggaactgaaccatcggccggcaattggaaacttaacaaagcagtgagactgtcttctgatattccatcgaagtgaaaacactctcaggagtttatgtatcgtcgacctgagaatggaacacaaagcttggaaatgtggtggtgatgtccctgcaaagttgacctgattagtgaatatagagtaaggcaataacaacatatgcaatagtaatgttatgatacagactgaactgaagcaatgaaataatattcacatgcctccagtatatcccctttgaagccggaactactacacactcccttggttgaacttgaaacagaaatactacttaacacttcgtagtcacacatgccttgagaaacaatatgacatacgctatttcactgatgtaaatgaacttctaatatagaagaaaacaatgagttggttcagcatactctccagcctccaatgtacaaaactgtcgtttaactaatcagtgtttcatattctgtctgctggttttcactttatagttgaacaatatcatgcttctttatttcctgacagtgctgctgaagacttgactctatgagataatgcataagtattttggcaatataattcaaggacagttgatactattattcattctgaaccccgcagatttcaatcaacttgagcataataagctgatgaaaagtaaagagttaacaacaaatacaatagcatatcttacttgagatattggagtgtcatgttcttcagtaatgagggatgtctgagaactagattgcgccattcttctttgtcaattttcccatcatgttttgtgtctgcctcctcgaaagtcttataacaacaggaaagtaattttgagaaaacaatacgaccaatgcataacaggtcagtcagttagtgcaatgtgccagggtagcatttgttcaacaatgcactgatataaatctgccttctgcaaaaccactgcaataacaagctttggcacaatgacataactctaaagttcattacctactcgacagcattaatgaaataggcagtactaatagctatagctatagctataagcacatagtcatttgcaaacctccattcgagcatttgatcaaatatatttttaactgaatttaatcaaatactgtaagtcaatatatggatatcaacctgtctagtttgatctatcaaggggattaacctcactttagcacaacataaacaaatcttgtttcagaacagaaaagggaattggtgctcaacatgattgaacagaaatattctacatggaacagtgcatagccatactagtatggcaaatgcaaggaattcaaccagaccttatcaatgatgctctctatgacttcatctgaaagattcattcctgattcagcaagtgtagcaacaaccatttgcttaacctaatagccaaacaagaaaaatcattggattataatatgggaaaagataaaagataaaaaaaatacacaaaggagcctatatgccacaattcattgacttcagaaaatatattcttgctcatacacgatctaaaagtaacatcagttccacaattatgcaaaagattgtggctgagcttttttctggagagttctttacaaatctatccatgagaaaacgatatacggatcagggctacaaagctacacttggtacaatatcaagttgaatagaggtaagggaacacagactttgaattgtcctaactagggttcaagaaagtggtgtaaggcggtaaatggccacctatcaccacctgtaagacttactcatagtcaacaagtactcacaatgctggctggagttgggttcattgaagatacttgatcattaagtgattaagggagataaaagaacatacaaaatatggaagaatataaattaatactgagctcattggaaaataatttgaaacaaactatgagataaatggcattcttgatcctgatcaacatgttaacttcacatattacatcctgattagcaacatacctcttgtctttcaatgaaaccttgttgcttcagatcataaagctggaaagagactgcaaggattatactgtgtaagattttttttttctttctgcttacaacaatcagataaaacactaaaagagcttaagggaattaacctatgccaaaaaagaaaagataatagagttggaccattttataatcacagtgcaccatcaaacaagcagaaacagttacaccttatttatttatttgtctaagatagagtaactgaccattctcacacagcaccaaaggactcaaaaatgaaataacaaaatgtgctttgggcaagtatgaacctactaatggaaggatatcatgaatcttttgtgttcttgcagtttatccctaagtccattcagatcaacacaagatcaaatacataaacacatgtcagaggatcatgcaaacaagagggctgacttgatatgtgcatattatacatgcttgggcgttacaagaattctagaagttattatcatggtaaatatgatagattagttggtacatcatctattatgatttactttatactagtgatggtgaatttttcaaaccccctacagagaaatgtgaattaagcacaatacatgtgcatgttactagtctccaccttgtaactttgcaggtagagggtgcattatgtcatttaaaataataaatatataaaattctgcagaccagatgtggctagataatgcagtaacacatgtcattccttttatatttactttcttccttactgtaaaatataacatatttttatgcaattagcgtgttgactgaaccatgtaatgtaattttgaaaggtataagaactttgaaatagcctttgcaacatatcgacatgttcagttctgttaaataattatccatttttattacatactccctccgtcccgaattacttgtcgcagaaatggatgtattatacgtctagatacatccatttctgcgacaagtaattagggagggagggagtattaccatacatgtagttgtgtcatttttatagtacaaaattttcactaatctactgccatatttaaagttaagctaactatgcaaatgtcatgattctctgaacttacagtcaattttctcttcaggaggggcacttggatgaaatactgatagcgcacgggcgaattcttcaaatcctagaattccgttgtgttttgtgtcaaataaatcaaagacctacaagaacagggaaaatgttgagagaaatacactagcatattatgtatcaactatcaacatcattcccgcttcactgtttctcctatgcacacaaatttactaaatggataaataatagaggtttaagttatcactgcaatgcctgactggatggtgtatatatgtggcttcgatgtgagtcactgtgaacagcttttatgaagagttttttttcataaggaactgttagcatgtgcagtgctatgatcttaatatatatgatcataactttcaattaaacagatagttttacttccagtcttagctagtaaagctggtgcaattctattgacaattaagtgtccccagaaagtggtgactacatttccttccaggctttttaaatcatctgtttcttattttgcatatcaccatacatgtacttgtgtacccaaaaatcctgcacgattctacaattaggcagatgccattcgccatatatagctcatgattcctacagaatgttaaacattgcttctttctctccaaggtccaaataatatgtcaaaaaaggagagtgagagtgactccacagtaatgacatcaaaattataccaaaaagaagaagaagagaaaacaacagaaaggcatttccactgtacaaacagcaaattcgtgttcacatgtgtgtaatgtgggcttacacgatcagcaaacaaactctccttcttactggtcttgaaaagcgccaactgaaactcctcctgcaccatgtcaacgcacaggcatcgcaattcaggttattcgtcactcatcatatatactacattaattttactttcaacgaaatgggtgttgatccactaaccttgttgatcagcccgtcgtctatcacagcactgctgatcttcttgaacagctcgtacagcgcttcgacctcactgacgctgactgcacgcaaatcacattacacaatcacaagataaacagagcacaatgaaacgctgcttgggctagcaagagaactattcttagatagacgtcattaatagcaaagagagctggtggcgcggacatacacacggtctccctggcgaggacttcgggatcctccaggcccttgggctgcttgaggtcgatgtcgcagcacttgaggagcacgccgcccaggtgcctcaccccctccaggcactgcaacatggtggtggcaccactgcgcgccgcaaccgtccgtcaacgcccgtctctctctctccagggtgggcgccgcgcaatccgcggggctagccgcaaatcgggaccccgcgggcggtggactggatttggccgaacgagccgagcggccacgcggggggcggcgtggctggaggatcgcggctgggtttcccgatgcggcgcggagcagaggggcggccggagggggtcgccgcggggttcctgttgttggcgccgccgtcgccgtcgatgccgcagtcgtcggctggtggtgcgattggtatgggctggaacggattggatcggggatttctcttatttatttattttcctttttgcgtgtgcgtgttcccgttgttgcgtttgcgattttgaatttttcccttttggcgagtcgtgactagctaggtcggcgcaatcccctcccttgctattcgatccaacggaggaagtgcgggcacgttgtgtttacaggttcatagacaccgcgaggtctttcattttattttgagatatacatgagctagttgtcggtgctacgccacggaaccatgagaaacaaggtaaaactcaagagcctctacgccaacttagatggggtttgacagttggcacagataagattagtgacgtgagaagccataagatgattgaggggagtgagacgataaaggaagagtgattcgggaagggtctgctgggaagggcgtacaagtttttatgttattttgcgatggatactagcaagttgagagtgctactccacggaaccatgggaaacaaggtagaatttgatttactcaacagcctcgtacgccaacttagattgggtttggcagttgcacggatgagattagcgacggaagaaggcacgagatgattgagggcgagacacgataaaagaagggtgatcctggaagggtatattggacgggcgtgcaagtttttttgcgatgaaattagtttacttaagagccttgtgggtcaacttcaatggggtttgatagttggcgcggatgagattagtgacggaggcacatgatgattgaggggcaagacatgataaaagaagggtgattttggaagggtatattggacgggcgtgcaagttttttttgatgaaattagttcactcaagagcctcgtgggtcaacttggatggggtttgatagttggcgcggatgagattaatgatggggggaggcacggaatgattgagtggcgcgagaggatagaatgagaaggatttgaaaagggtctagtttgccatgacgaaacaatcaattcttcgcatctcatcttgtatattacagacaagtaagccgtggtagccaaaccgctatttttttggaaaaaagcacttcactctatttagaaaagggtttggtagttggcgcgaatgagattagtgatgggagaaggcacgggatgaagaatagagtgaagtgtttatatgggtttttattgtcccacctttgtgggttgataaaaggaagactaaaagaatagtgaacatattaagatcaactctcataactaaaatataatatgtgtgcaatctacttgtaacgaaacaataactacacgttcacatttatcaaaaagagatataccaagtagtgcattcgaatatttgaactggataattcataatgatgcatgaatacaagtacaccatgtgttgcaagtagatagactaactcttctcatacttttcttgcgaaatgactcttctcacactcaactccctttgccggcaaaaaaacacttaccacttttgttggaaaaatgctttgttgagataatctagtttgccacgacgaaacaataaattcttcgcgtttcattttgtatattacagacaagtacgtcgtggtagtcaaaccgctattttttggaagaaaaaacacctcactctatttttcataatcttttaccccatgaccctcccccaccccaccgcatgaggtctggaaatatattgattagacactgttgatccattcaacatcaactgcaatattaatctctgttggaagagagtcttgaattttagcaacaaaagtgagtgagccccttgtattgtaatctctatcaatcttacaaagtctaacttccctagttttgtttgggtgtgctctaatctcgacaacttacgaagcataactagacatgaatactttcttcgaaagaaaatcatggacaagacttccctactatccatcaacagaataattggctttagtgaccattggagggccaactggatccctttcccgccagttactaactacgttccgatagcatcaggacaactacgcaaccatctaataggttgttatagtatcattttcactatgatcctgcaatatcatacctgctcttatagtattgtcaacatgttatatgagctattggtatttaatttcaaccagtcttcagagtggtggttcccaagtgcgagccagctccatcactagttgttcaccgctatgttccataggcttcttgcgccgcactaatagtagttgactaactacttctcttttttaggttcaattgtggaaattgtaatatgagaaggaatcaacctaccttgtcaacaaccactcatatccctctattgataggggcaagtttcacatgtgtaattttattatgcacataccagatttttccttgaaatctagtagaatgttggacataattcagttgcgccgccaatgggaggaatggtcattctagaccttcaagttgtacagtgtgcattgtaaatctcaccctattgcctaaaattttgcttagcttgccatgcatactctggaacttccttttatgcattctcttcttgagtgttggcagacccaaatactttgcttcaaattctagcttgtcaacttgcaaaattactcggattctcatctaaccacatttgttaataactcacaacactagtagagaaatgactagccacaattttagttggtggcatgtcattttcaaacaaagccagcactaatttttttcaaatagtggtgatattagcatatttggtatgtcagctttatatggctagtgttggcattgactttttttaacacgtcacaaatctgttgacccagttgtactcgatcacccattggaagatagtggtggcatcagattgatatgcacaccgtagctaattagctattgctggcgtgctaacttatgttacgccaaaaactttttttttattttatctaatatttaatctttattatagtagtagtattttttatcattttgggtcttttattctttactactagttatcattttgagtttttttattttgagtcttttattatattgagtttttttattacttgtagtcttttattaatcttagtcttttcttatttgggctgtagttagctatggcctcgcgttttaggccaaggtcagcacaggcctacctcggttaacccccctcctcaataaaagacctagtcgttgcctcatttcacccacccacacacactccccctcaaccaactgccgttgccacctccggacctcgccacccgtcgccacaccgccaacagcaaggttcgatcctctccttcccctcccatcgcacatctccttcctctccactcgcacctcctccgtctgcggacgccacgaaatccatgggattcatgtataagttgatttgtgtagaatttaggtagtgtttaatcaacatttttagtgatgtagaaacaaaagttagttccatggattgctttgcgtagaatccgattttagtggtgtagaatctgattgctactatgtagaatccgattttaatagtgtatttagcgtataaatttttgagtgtctggaatattaataattcaagttcaactttggtaatgtagtctggaatgcttggaataaataagatgaactcctataaattagattggtggtgtagaatttgtttatttctgtctccgtccgtggattttgtttatttgtctatacaattagtagtgtagaattagatttagcagtgtagtattcttgacatgttgtgaatggttattgagggtttgtttagtcagactttttttagaaataatttggttgattcgtagcaatgccacatcatgagttatacagaataacttatcgtgcacgtgtgtgcttccttcctgttgtcatatatagtttagttatcaaatgtcatattgtaggcatctgttgtcgtgcacaattcaattgtcatttgtggtcatagttcaattggtcctagagccaaacacctcagaggctcagagtactatagtattcttgacataatttgatgtaagtgcatctagtgccacccctagttggttttggagtattgacgacaaacttggttgagggactaatgtgtttgtgagaattgcaggataacacatgtagtagtctcttattgattcggtttacctaccagagatgacccctaaaaatgtgtgaagacattgaagacaatggtggttcgtgaagacattcacgatgaagattatgacatgagaagacattcatatgaagactatggagtgcgaaaacatagttgtttcatagtttccttttcttccttgttgagtcataggaaccaccgtactgttaagtggggtccaagtgaacaaagtcagagtgactgaagtgatgctcaaccaaatcctatgtcttcgagcgaagacaatgagagcaaatcttatccagagctggatgagccagctttacttgtagcccaagtcaagctgccgcgtgtgtttgaaatccgaccgttggacacgtgtcagttccttagtgacccagggtcatttcggacaa

>*TaCBL4*_1AL_3951410

gccgccgagacctcctgtgcgtgagcacccccattcccagctccagaagtttgcctctgcctttcctctgcaaccgactcaactgaactttgcgtgcagtcacggtgaacgaggtggaggcgctgtacgagctctacaagaagctcagcttctccatcttcaaggacggcctcatccacaaggtacgagctctacaagaagctcagcttctccttacatccatctcacgagctccatgatcatcttgtccgatgatctcgcccctgggttctgaacttgtggctggttgtttcgttcatgtcaggaggagttccggctggctctgttcaggaccagcagaggggcgaacctgttcgcggacagggtgttcgacctcttcgatctcaagcgcaacggcgtgatcgagttcggcgagttcgtgcgctcgctcagcatcttccaccccaaagcgcctgaatctgaaaagaccgcgtgtacgcctcctgtccattgttcgctcctagtttgccgtccattcatcttcttgtcctctcctccctatgaaaccaatcaaaaatctttcgtttgtctccatcatcgtcctccctgcagttgcattcaagctgtacgatctgcgggggacaggctacatcgagaaagaagaggcaagtactaaatcatttccgcatccaacctagcacatggcgcactttattgtaatgtcgacgcgttatttctgaattctaaaaatttatataatcaacacattgcctgcacagctccgggagatggtggtggcgcttcttgatgagtccgacctatgtctctccgatagcgccgtcgaggagattgtccataatgtatgtaatcaacaacattgatacttccctccatgtatcggaggtattttcccgttttccttcttaacatgttctgtgctgttctgctccttttggactgccagacgttcagtcaagcagactcggatggtgatggcaggatagaccccaaggagtgggaggagtttgtcaagcagaacccagcgtcgctgaggaacatgtcactgccctatctccagtgagtaccatcatctgctgctctactttccattactgctcacagctgatgtttccttttaatttctcagaatcggtagttcagcttcacctgcgatttcttagtttaatcttcttctgatacaaaaatgcggcatttagatgagtgccatcagttatgccatgctagagagtagaggtctttttaatttttaattttttttgcccattccatttcacgtgatcttattgttgttgtggttgtttattcagggacattacgacgacatttccgagctttgtaatgcattccgaagtcgaagactacagtggaatcagcaaataaggcacaattaacacttattttcggtgtcatatgatccgttggtcctgagctgggaggcagcgcattcaagagtgagctgcagcagaaatgtctggaactcaaataggaggacaagagatggaacgccgcgccacatggactaattagatatttagttttttttgtaatatttggtaagttgacccaagacttcttttgtggcaaggtagttcatacaagaacatgctacatctagttggtacataggtaggaaattgtgacaaagtatttcatacaagaaatccatctagttggtacatacatttgagatttacgacacatagtaatatagtagatattggacgcaaaattgagaagctctagctctcttcttagaactaagatgttgatcgtccggctgggtttgggtgatggggctgggaggccgggagtgcgagattcaggggactcatgataatgaggccttttatgagtgatacttggcgcagaattgggaagctcttgttctcttcttagaactgagatgttgatggtccggctgggtttgggtgatggggctggaaggccgggagtgtgagattcgggggaatcatgatagggaagccttatatgatttatttagcggcaaagcggcgcacgatctgatacaatctgacgggatccttcgagaaagacaaatctagaaaacaccatgcaaaatatctgaaaatcatcaccgtctagaaggccaggctaaagcagcaactgacatgaaagtataaatgtttttggaggaaaggtaactgtaagcaatacatttggccacaacttacatttttagagaaaaggtgatggaccttgcttatattataaccatttcaccaagttccttgtgtctcagcaatagctaagtacacaaatctgaaacagatactggaaaaccagattgcctgatgagtcctctgaaaatcatgtagcaagtccttccttctcacaatatagcactaataatcatgctcagatatttctctacaagagaaatgggtatagatggagttaattttgtgcagtatttatctgtagatgcatcattttcctataagctcctaatttagttcttgaaaagagtaagaggagcacaaaggaggattggtttttctttcttttgtctcagataattttacaagaaaaatgagaacacaagggagaaggaattggcttttcacacattgtgtgcgctacgccacgtcgcaccttgttgtatatttatgtgtacttacttgtttgattcgtaaatttaatgttgtttcacacatttagtggtagggttgaagaaatttgatagataaaacatttttggggtaataaagagcatgcccatttgatatagacaatggtgtatcacacgaacagaaaattacatgagaaaaaagagacacactactaataaactaagctaaacatgttacatagatatacaaagttagtgtggcttagacttgacgattctacacttctctggtgttattgagttgttcattcctgcacaattgcgggaaatgcgaagcggcagaatttatgagaagttgtcacaaatcttggttaaaattcttgtccgtcttgtaaactctttctggagggaaagagcatgttgatgcggcattgcttggtataactttactcctgggctgactgaatagtggcagcttgggcattttctttttgagtagaatgttatttttcttattagattcttcttgttacagatatcaaattattgttttccatttcattcagaagtagagaatgatatatctacatatccattaattcatatttgcacttcttcaatctcagtttgccgcgatagtaaaacggttgccgcgaagccgttgctgtcatactttggttcctaagtatggtctttaaaaataacagaatttcatactcatgcataaatctatgtgttatgtttaattgatgattatgaattagggcttgaatttcaacaccttgaacaaagcgtacaaattatcaatgcttacaaagcaacaaaaggtgtaggatatgtatgaaggtaagaatcctcgtttaaagatcccagccgaacaataattttaacaacccaatatttatcttctgggcaccatgccaacatcattcagaagttcagcatctttattttcctctatgaagcagcgatacggcaacatggatgcggcgataagggtacgacgacatgggatacaacaattcagcaatacggcgatacgacaagtatagataaaaaattaataaaatatcatgttataaagacaaagaataaatacatgcatcagatgagatcaaaatactgccccatatgctgcatccaatgttcttttcatcaagttctcgtctccttgatgactcaatgatcatcaatttactcaatcctcccacgacttgcaaaacacatctgctagtattagaatttgaaatatacagaatattaagcatgaaaagttggacaaattaaataagttagcttgtaccttttctaaccgaatgatatatatttggtgcttcccagcctcttaactgaagattgacctgcagatgccatgtctatttctccctggcagcaacttcaacaggtcagcagcaaggaaactgcattctttagcaaattatagttgcgtacagcaaaggccatggcccggtgcccagcagcagctagcaagcacgcacacgagcacaccacaagtacgtacacacagacacacacagcaacaggcacgcacgccgcacgcggaagcaagagcacacacagccaacacacaagcaaacgcgggaggaccggacacgaggatagcagggaatggagaaggtggacgggataccttgatggcggcgtcggagactccaagcaccagcgagacaaatccatggggcggttccagacaccggcgagcgaatccaggtagtggcggcagtggcagcaactccgcagtgtactcgtacggtcactggcgaatcctacatcaaatctgctgcacaacaaattgtgtgagagagtaatggctgaccaaatcgccgagcagccaaaaaagaaccacgacacgcgtgcggagcggcaccatgaaacaccagatgaagaatgaacgaaatcacggccagccgaacagccaagaccagccacacgtagagagagagtcgggctgtaggctggcgacccaaacgacggcggacggccgtgcaccctaccagcagactcccgccggggccgtgggtgttaccgtccatcggcgacctgtaccacctcgccggcgccctcccgcaccgtaacagagaccaaacgaacaggaaggacacaacaagagggacatgaacgaacaccagacaaggaagtacttataggcggccagaaaaagagaagaaaataaaacgaaacccacgagaaaagaccaaaaagcaacgtgtcaattgatggcataggcagtcaacgatacatgaagccaacgtgggcacatgcatgagccatgcaaaggcacgtggaacaacaccaggccacatacctctagaacgtccacgtgaattcaaccatcccaacacacagtgaaccaaacttttttaaaggacaaggggaatcttccaggagaccacacaacacacgcgtcatgtcaacaataaacaaaacatgactgcaggaaaaataaactcaatgacaccctaggagaccatcacacacacgtgtcaatcaaagaaacacggtaaaaaaaatccgaaaaaacaggaaaaatcaaacccttacaggcctagtcttctttgtatatgcacagttcaccgaagctgtgaccagcgagtggccatagattcattgtgctaagtagtcaactggtaatggcctgacagacctcttttgttgaaggttatgtggaggacaccatttctcctttgttgactgcaaacatacatatatatagacaatagcaagatgcccatgcgttgcacggaacatcaagatatatttgtatgattaatttatcttatgagagaaaaagatgaacaagggaagtccttatctacaagtgtggagagagatgcgggtatctttttgcaaaattgccatagtttgttttctatccgtcagatatagatctaacggtctatattacacgatggcaggcacaccgtcatccctaattcggtttttttaatataataatatagatagacaaaatgccgatcctcgcaacagatgttggtatcaactcccggcctctgcatactgaaatcgatgcacgcgggcggccagggcttactttgtgctcgatctcatgttagcatcattttcggcaaatgtcggtatccacctttgtgataggagaatacatatataggtgctagggtaatggagggttgtttgtcggagaaaatggtctctggctgctgcatatcatggctaggctgggtggtcgactagcccgtacgttagcatggttgtggtggcatccagagctgggcgagtttgaccttggagacgctctgcagcacctccagcggcgtcgcggcgccggtcaccaccaccttcttgcgcttcaaatccacctcgaaccatgccacgcctgcagcagcatcaccaacagcgcacactctgagacctgaattttaccacgacaccataacccagccgacaaattttaaaagaatgtgtggagtcaaagccgacgccaacaaatgaatggacacctttttcttattgcctattgcatattatagataaaaagaaaacttgattttgtgctcgtgtttttgcctctttacccaactgttctgaaagtgacattggtggtgcgcagcagcagactagctggacttgtgcagcaaatttccacgacatgatgccccaacgcatgcaggcatgtttcccccgtttgtaggaaggtggtggcatgtttcattcacttttaagaaagttgtgtagtttgcatcactccacctttcttccgtgcactggaaatttccatcatgtgcggaaagggtgttggttggttggttcagcgcatttaccatgtgcgcacacgctctgccgacatccaatttggatagacaaaacaaatcgaaaatctaggagtcgatgaatacttacaaacttgtcgcccctagttaaactttataatacaatttgataactcatttttatccccgggtaaaaaacacatgcaaaaggaactgaatacagattttgtaagaatggcaggtaaaaaggtggggccgatatgttttggttgtaagcagagcctcacatcgctcctaacggaccggcccatataagactccttcgtgttctgtttttcaaaatcctcctccaattttgggaagcttcccaaatattttttatgtgtttgtttttccggttttcacttggtttttatccagtttgtctttttttcctttccattttaatttatttcgcttcctttttattttttgcattccctgttctaaaattattatttttaaatatactctttttcaaaaatttaatgttttcacaaaaaatcatgaactttcttttcaattcatgaagtttttaaaaaaatcatgaagttatttttcaaattcatgcactttttcaaattggtgaactttttcaaaatgcacaaaaaaattataaattcatcaaacatttttggaaacaaatgagcttttttaaaaattcatgaacttttgaaaccgtgaaaatttccaaatgcatgaatgtattaaatccatgaactttctataaaacatccgtgatttatttccaaaatccataggattttcataaattcatgaactttttctggaaatttatgagcttttttaaaatccatgagatttttttacaaatttgtattttgtttaaaaaatcaatattttccaaatttgtatttttacaaatttatgagctaacgtacaacaccatgctaacttttgattcggtgaaaaaattattgaaacttacttcggtaacttctgtgtgagtagcattgttacttatggtacaagtattatgttaattttgacccatggaaattttttgtttaaaacatacccaatagataatgtggtgatgatctcaagtagtcctggccatctccgacccggccgtttcagcccacccaggcccggccctaaaatccagggcctaggcccgatgggcttgcccgtggaccgggattgggcctgcgtttggagcccaccagcaaggcctggacgggcttgggctcggcatattggcattttatcgaagaggcccggcccacgaccctaagacctaagggctttttcagggctttttactagatgggccgggcttgggcttgaaaagtaggccctatggtagggcatggacctcaattttctgccatgggcttcttaggcccggcccaagcccggcctggcccggcccatggccaggtataatctcaagccacgtccccattgtgtttacttgtcttttattctaactttgcatattcttgaaatgtcatttttcataaactttttggttaaaataactgcccgataagacttattatgtatttgtgatcatcaagctttattaactaaaaatcaggttttaaaagattggtgcatcttaggatcggaattttaggaaatatttttcattggtgttataaagaatagtaattaccttaagaatactaatttaattatattgtgttctgactgaatataccggagttatctatacatggttttaaaaaatattatgaacatattagtcatctatttaagcaaacatatgttgaagtttcttgaagtctacataaatcattttaaagtcagttcctacttatttttattttacttatttacatgtttcaagttgctgacatatacaaatttatttgtaagtaaattattcatgtcaatattttctataagacggataattttttgtattcattacttcgcttcatcaagctagaaaacccagtgtgagttatataacatatatttatttatactaaacttttgcatttattaataatttattttttgacatccaggctccttgctcgtttaccttcggattgctttaagatgcgtgccattgtgttagatgtagacactaacgggtgtacccgtttgcagtattaaagacgaagagttattgcaatttactaattaatttcatataaatgcatattattagcgaccacttgctttaatttggacggaggcattaattagcatgcacgcaccaaatactacttgctaagaaaacaagaaactgcctaatttattcatgcatgtacgtgtgaaacaataaaaaattctatagtaattaatgcatagaaggaaattgttagaaattgcctaattttgttgtacatattagtagtattactaacatggtttttaaatagaattaggtgtgcgtagagtggatattttcattttctgcctccgctatgggtataagaaaaacttgtttctctttgtactctacctagttgtttttagggtatatatacccagaaattgtgtaaggataatatgtattttttggtatcactcatcagaatcatcacatttgatacatctctatcgtggtatgttgtataggtatcatatttatatgaagataacatatcatttgtcaaggtatttggtttgcgtatctgaatccacttataaaaaggcagtgacatttacaggtatgtaccaataaatttcacgtcgaggtatttacagaaatatcttgagtatgcattgtttttttaaaacttgagtatccacttacaaatatatcaaactacgtttgtaggtttctaatacctagaataaatcataaggtatatacatgcatgcaaattatttggaataaattatatatctacgtctgtatgtatctaatacctagaagaaatcatagggtatatacatgcatgcaattatttggaataaattgtagggtacatacatttgtggatatctaaaagtacctagaatgaatcgtagggtatatacgtttgtaggtatatgttctaatacgtagaataaaccatgcaggaatcatctacaattaatggtcgtggaagatacaagtgcatgcacgtggaaaaggaaagcaaattatttgaaaggttagtagcgattatttataagcagggaatgcattagcgggttggacccgaacaaggtgcccaggcacctaggtgccccaaacattttacctatatatatatatatatatgagtgtgtgtgtgtgtgtgtgtgtgtgtgtgtgtgtgtttggttagtttggttgattttgagcgagttaaggtgaggaatacacgacacttacttatggggaggaattgcatattcgtctagagattgaaccatgataaaaccaccgtgtacaaatttccttgcaaataatacgattctattctactaagtgtcgagtatgcacactgcatatcaaacttagaaaaacaaaactgaaggcattcaatacaagttgaatgatgttatatttctagaacaaatgtatacaatgtcgacagattgttgaacccaggaaaaaaccaatgaataagcaaccccaacaaatgtgagaatagacgcttgtagatattttgtttactctaacattatcctttctaattattaacaatttaaaccatgcactccagtaacctctatcaactttagtactttctactatagtatttaacgatgctaaaatagacacaaacaaatacatatagatacacttcatccaagttgaataaattagtctataaagacatgtgattttattattttctcttttcccctcatctaggaaatctggtttaaaaaaagacaatgtgtctaaataaaataaaaggcgccattttatttgtaatgtatgccttaaataaatattagtgcatttttcaatattggcagtatgcataataaaaacattatatcttatgatatggccacattgttatgttattaaattaactaataaaataaaaatatta

>*TaCBL4*_1BL_3917583

cagcacactcacccaacatcaatgtaggttgtccaaaaaattcaactcaaaattcaaaacatagcttgaaaaacaaaaataacaaattcaacactgaatagtacacaacataactttggctttagatttggcccactatcacacttatttcaaatttgtcatttttgtatctcaaaaaatatttcaaatttttatacgatttttttgacatcatacattgatgttgcgttaacgtgctagatttttttcagaacttttaaaacattctatggcatccggtgcaccagtagcaccacacgtgagggtgcaccggatacattcccctgttcgctcgaacagaatgttcgcatgacacgtttcgtacttggcggttcactgggggcagccccagtgtcgttccccagatacgggcgtcctatttgttagactcataataacatggagattttttttcatggaaacatggagatcaaattagaacactttcagtactcaaataaaaattagaatactttcatcataatttttgtaaaacttcgaatatttaaagtgtaaagtgtggcaccccaattctacaatcctagtcgtccgtttctgatcaccgtgtcggtctaccgctcccgctatacctgaccatgggcagcccggcctggacggctcgaccttgccgccgggcctagattttgagcatgatgaccgggctgggtttggacccttcatatctacaatttaaggaagaggcccgagcccaaggcccgcgagctttttgactgatgggcctggcttgggcctgaattttaggnnnnnnnnnnnnnnnnnnnnnngcacacgtctaggttttttgtgtcgggctttgataggcccggcccgatgccgccacgcggggaggtgaacccggtttgtttacgaaatggcgcgtgacttaagtggcggtgcaaccctcgtcgttcccgtgcttacatgcgggcagcctacgggagcactggtcgacggtactagtagcagtacacacgtacgtacccatgtgcctgcgtgcgatgtgatccagactcatgaaaagcacgcgcatgacgagctgaggacggtgatggatgcagaacagtcggacacggcgagatctcgggagacagggacggccgccgcttgttgcctggacccgcacagtcgcactcgcactgcagcgggaaagaaccatttcacttggcgccccgttaaatcaggccccaaggaggaggaggagtcgagtccagccagccaagggaaagacggaggagacccagcagcgggaagaggagaagacagaacaggagaagctacgcgatctcagctcaggtcagattgtgtttccggtttcgtcatcacgtctttcttattcttgctacgccgttgtgctgcctcacgcctgcctgccttcagtgcctctcgcggcgccggccggggtgggaagggatcgaccgaaccaatgggctgcgcttggtcgtngtcgaggcgtcagcggcgcgctcaggggtacgaggagcccgccgtcctcgccgccgagacctcctgtacgcattcccagctccagcttcacctcgttgtttttctatacgcgcaactttatttacagaaatcctattcctatcaaaactggtagtaacttcgcgtcgcgcgcgtgcagtcacggtgagcgaggtggaggcgctgtacgagctctacaacaagctcagctactccatcttcaaagacggcctcatccataaggtaaactccacgaccgctatggatccatcttcaaagactgcatgctcccatctcatcatatctcatctgcgttcatcacaggaggagttccggctcgccctgttccggaccagcgagagagccaacctctttgcggacagggtgttccacctcttcgatctcaagcgcaacggggtcatcgagttcggcgagtttgtgcgctcgctcagcatcttccaccccaaagcgcttgtatcagacaaggccgcatgtattgtccattcctctgctcctacacctcttgtccattcgtcttaatttgatgtccatcaattatcatgccagagtagataaccattttcggccatcctctcctgcagttgcattcaaattgtacgatttgaggggcacaggctacatcgaaaaagaagaggtaaattcgtaatgttcatgagttgttctgaattctagtataacttgactgttgacacagcttagggagatggtggtggcacttcttgacgagtcggacctatgtctctcggatagcgctgttgatgagatcgttgacaatgtaattaacgagatagggctacttctttccaaacattcagtcttttaggagtaaatagtatttggtttccttcttcttttttgaacaaaatatataatctccttccttctaacatgacctgtgctgctctgctccctttgggctgtcagacgttcagtcaagcagacacgaatggcgatggcaggttagaccccaaggagtgggaggagtttgtcaagaagaacccgacatcactcaggaacatgtcactgccctatctccagtgagtaggcactccgcactcatctaatgagtttgccctttaccacatatcaagtgatttgatggttgttgttgctgtcgttgttgttgtttgtttagggacattacgaccacgtttccgagctttgcaacgcattcggaagtcaatgattgtggtggcgtcgacaaataaccaggcagagccttcctgcctgcaggtgcaggggttatcagatgattgtcatataatatattacctccgtctcaaattagttgttttatatttgtgtagacactaaaacttgtctagatgcatctgtatctagataaatttacgacaactaaaacatgtgtagatacatctatgtgctacatgtacaccacacacggtgtggacttaagacttaatttcaaaaaaagagtgacaaaatctcaaggcaatagaggcactttcatcgtcaatggtacatttgatgatatcttatgctccaaattcaagtataaagaggaaggctaatattcagtattgacctgggtcacctgtgttatatatacctcttgtaagccgccgcacggaataagttgatcagttgtaaatttccagcgtttgtaaccttccccgatatagtgaagtttggctggccggcgcgtgtgggttttttcccttcgtgttggacgggtttcccttgcttgttctttgttttccgttgagttgatttgcttgtcgtatctctaacatttgatgactgtaaaaactgcctgagtttttttatggcacggggactgatttagtttgctaggtagagaaagcacactatccatgtgacagcaataatcctaaacacaccggcatattatgcgagttttacaggcttccttctatctaatgtctctgcccccgaattatggtccacattatagaaagctatagtatgcaactcaatcgaaatttgcttttggagatcgagctccacgaaagccttcaaatgcaaaattcaaactttataaaaaattgtatttttataattcaaaaaattctgacgaaaaaatacagagatagatgtaggcataatgcacaagtctgcaaattttcaggacggagtacattgacatgagggctgcgcaaaaaaaaaatctaagacttcttaacacgtgatactattcatcatcgcagaccatgaaatttattttttgcaggtcgcatttcattttaaaagtttaagcacatacgcctcacatccttgtttacttgtgcaaaaaaatcagaattttctgaaaccaaaatttttgaattttgattttttcaaaaattctgcctttgtggaggctaagatccaaaacgccattcttgaactcaatttcattcatcggagtttggtacaatatatacaggagatgtcttgcgtgacaagccaaataaccctaccatatttctagtagttagactatacatggctagatatcataataagatcaatcgggaataaatacggatatgtcacgttccggaacgaaactcatggtatgcggcgattggcaacccttaggtcataatttcggcaaactgttaggtagtcggtacatgaagaacatggagctgacccaactggaccttctcatgcacaaagtgaacatccaactcaatgtatttgctgtgcttatgctgcaccggtcggctgcgagataggtagccgaaatattgtcacagaaaaccaccatcgccttcagaagaggaaccataagttcaccaagaaggtgacgcagccaacaagtgtcggcaataacgttgatcacagcgcagtactcaacctctgcactcgagcggtagacggtgagctgccgcttggaggaccaggagacgcgtgagtcacctaagagacacggtaaccggctatcgtgtgtcaggacagccatcccaatccgtgttcgtgtcagcaagaagctcgttgaaggcggagaagtgaagacgtagaccataaatcggtgtgccgcgaatgtagcaaagaatgtgcttgaccagagaccaatgagcatcacgtggcgaatgcatgtgcaagaagacttgctgcaccatgaactaaatgtcttgtttgtgagtgtgagatactaaagggcaccaaacaatgttgcgacagagagcgacatctgtggcaggctgtccagctatggcagagagcttcgacttggcctccgctgatgtggcggccggtttgcagtcagtcatgcccgcacggtccaacaggtcaagagcgtactgcccctgatgggggaagacaccggaggcatcacaatgcacattaaagtcgaggaagaagtgcaaagtgcccatgtcctttagctgaaactccgagccaagcctgaccgataatgtcgcgaaggaagtcctctcgagatgccgtgataacgatgtcgtcaacatacagaagtagcatgacgacatgatcagcccaatgaagaacgaagagcggcatctcagaagtggtgttgcaaatccgagagcggcgaggaacgtcgcgatgcgctggtaccacacgcgaggagcctgtttggggctgtagagggacctcgataacaagcagacatcattcgatcgctacgggtcgacaaaaccgattgactgctaatagagattacgctcttggagatggccgtacaagaaagtattgttgatatcgagctgatgtatgagccaaccgtcgagaggttgtaagctgtaagacaactcggatcgtggcgggtttgaagatggtagagaaggtctctgagaagtcaacactagcacgctgtgagaatccacgaacaacccagcaagccttgtacctgtcctgcgagccatccgggtgaagcttgttcctaaagacccatttttcggagatgacgttggcaccaggggccgagggacaagagtccaactccggttggtgacaagggcatcgtactccgcgcacacgacagtagtctagtgcaggtcgctcagtgcagaccacaccgagcgtgggactggaaagagcgtcatgaaactgaggttgtggtagtgcgggttcggctgatggatctcgtccttggcatgcgtcgtcatgggatgatgaggcggcaaaagtggcgggcgnnnagccacagctggtggcgcgggaagcggtgacggaggcgcagaaagcccagccgaggtggccgagggcggtgacgagggaaccgaaggcacggccgaggcaacagaaggtggtgacgggggcgccgccagtttggcagaggaagccgagctggtgatggggcgccgaggaatgtgccgaagagttgatcaaggcagctgagagtccggctgtggcaaccaagaacggtaacgtggccgcctaggaaccggctgagggcgatggtgtccgcaccaaggcgggcaagaaagccacgtcacactacaagaaatatgtcaactagtgaccttctgtcaatgaccctggaagaattggtcatagatctatgactatttcagaccaattggtcaaaagctgctcggggggctccaaaccctaaaccattgcgaccattttggtgagaaaggtcgtaatttccttacacgaaatggtcacaaagcaaacagtgctagtccgctgccttatttctagttgttaatgaccaatatagatggtcatadccttgtagattgtggtgggttgtgatgactaggcgccatctcatcagttttgcctatgtgtcatgtccatgtggcagtttttgccctaggttgtgaagcaacctatatttctgttattccaaaaattcccaaaaaattctcataaatgttttggatcatatattcatcaaatatgtcaaaaacattccttgcctagttcaaaaataattcgaaaatattcattttcctattcttttcagagcagcactttgtgaaggaagtaccactttggcatgtccaaatggtatccattttctacactgctttcctatgcccaaataaccatcctccaccaaatgccagctcaatccattcattattttgagcccagcttcaacattcgtatttatgtccagtgtgctactttgtaatgcaagtaccgcctaggctcctccttttgaggtgaaaatttgtgaagacagtcttcttagcaactgatcatcctcagccaaaactcacgcccattagccatgtgcatttcccataccgcaaatcaaacacttggctgcttattcatgtttgagcatcgatcggtctccccgtgagaatcttatgttgtgattttgttcctaacacctacctgcggagtgcccaacccactagacatgcctaggcctcccagaacacatggcaacgccacggtcacgtggtgaccacgcggcgggcatgcgagcttacgcgctctagagttgggcccctcggccaccgtccaaacctcgatgtcttgccatcaaatcatgtatttctgattaaatagatacttatttacctagaaatgatttttggaaaaaataaagagcaaactatgaggcagctgcagttcaaatttgacccgcttccaactgaatcgacggcaatttgtctttttcaccagaggtggatcaaaacttttttcacccaaccattttgtcaattatgcattatatatatatcctagtattttatgaaattgatttggtccatttttgcaacaattatttggtaggtccttcacaaaaaaacctccttttgggcactcggaaaatgaaaaatgaattttctatgcaaagaaaatgaaaacttacttaggaacattgtttggaattccaagatgcacccttgtgcacaatatgagatcatttgaacaaactatgccatgaat

>*TaCBL4*_1DL_2273437

gggttaaggaagggtctggaaggtgtgcccaagaaaaaggagaggaagagatcggggaagatagctgcctccaaacaagccagagcacatagcagccagacgatggattctgaagagcgtgtacccgtgaaaggtgcggccatgttccatctcacgggcgagccggtgctaccgccgaaaccgctggaggcactatcaggggatctcaggagactgcacgaccatgtgccgtcgactgagaaaagcctactagcctcgaaggatccaggatatccgacatacgcggctcatgtgcctgaggggaagtgctacgccgacacacggcccgcggaggtgttcttcctgcggtttgaccatatctttgagatgtttctgacaaggcggctcgattttacaatcgtccgcctttttgcgctacatatgagctccgtcatgaagagagaagaagtctcgcaaatctgtgtggcggatccgtactacatgcacgagtctttcttgagtctcgctgactttgagcgtaaaactgctagggactacctccaaaacttcatggtacagaataaggacgaggaaattgtcctcctgccttatcatccaaagtaagtcagttccggacaaccctttcatacatttcaatcattccttctcgctcatatggagaataatttgaggtgtcttttccccgcagcaacgggcgtgccgtccttatcgttctttacccgcgagtctcccacgtcgtgtatttcgacccttccagagactacgagaagagggactacacccacataatgaatattctagatgatgctctccaaggctttagcattagaggtggccacatgcagatcaggaaacaaaggaacaagaagatgggtttcgcgcataaaactaacttctgttgcatccatgtcccaaaaccaagcaagaaggatggattctacatcctccatctcatgattgagttcagcacggatcaccaaaagcttcgcatgacaagcagaaatgatgatcatatccacaagtggctagaatctcatggagaagcggattataaacttagagatgacttttttcgcatccaaagggacattgcgacgatcatcatgaaagaagtcgtcgatgagaaggggatgttccaccacggccctatatcgcgatctgacgtctgaactcgcataggcatgcaacgtcaagacctcacgccgttcaagaagctagggtccatcctcgatgatatggaaggatggaacttctagtgatttacgatgccgatgatgatgatatgtgtcggttgaacttgtatactttctgtagcaatgaaactttgtgatgtccatggtccccgccgaacttgcgtaacgctactttgttagtttggctacgatgacctgcgtacctctagttaattagtttgcgtactgtatgttgcatctagttgctaattaaccctttctttttcgtgttgctctaatatattttgttgcatatgattgtacatcctcttgatgaagatgcatctctaacaggtacctagtttcttgatggcgagatggcggtgctatgtcgtgtacaaagggaaggttccgggggtgtacaatgagtggcctgagtgtcaggcgcaagtcaatggggtctcgggcgccagccataaaggcttcaaaagcatacaagaaggagaagctagttacttgaggttcacgctagcgcgagagaggactcgtaaccgccgcctcatgtactgcatagttccgctctcactcatagtgatagctcttctcgcgtataccattgtttagatggatgacattgtagttgcaagtattcgagacttgcatgtatcgttattttctagatgatgacgagataccactgtgttggatgatgattatgatgagactatttgtatgtatatgctatgattacatttgtggtctcgcagccatttgtatgtgtatcatgatcatatttgtatctgctaaagattcttgtataaagcctgttcaaatacaaaacaaatatgcagaaaaaaaatcaaaaactactaaaattagcagtagcgagtggagaaaagttagcagcagcgtgacagtagcagtagcgcgtccagacaaagagcgctacaactattatcagtagcgagcttccacgcagcacgctgctgctacacttgtatagcagtagcgcgggcgtgcacgcgctgctgctaagggttagctgtagcgccttattagtagcgtcggtccccgcgctactgatatacctagaaaccgcgctgctgctaggcttttccctagttgtggtggtggtggtgatggtggccagtggcggagctacagtaaggcccaaagggccctggtcctcccaacctatgagattttctttaaggtatatctacctttgggccatagaacacaagcccaataaatatttctatcagggggcccgcccaactatttgggccaagctccgccactgatggtggccatgtgtacggtggtggttgtgatttctttggcggggatgttggttgagttcgtgcatgccattgattggtctcaatccacaacataacacatgctatatttgtggtgattttggatcaaacttgatcagtgaagtgatgccttttggatgaacaatgtatgttttaaagcgattgatgaaactatatgcttgttaacggttatgttatgaaatatatgcttgttattaatggatgagctatggatgtttgatgacatatactgcgaagtgtttgaagttcatgcggttaggacaaaaaacttatgcatttttttaactccactaagtttaggggatcaactaggtgcggccacctatagaggagtaaaagtatactcaactaactttactcggccagatactcctctatttttaggggatcggctaaagatgctcttagtcattagtttagaacaaaaatagctcacttctgaaagtacttcatgatttgaccttttttgtctaccgccgtcagtcacggcagtcgtagggtagatgagctaccaccatagtcaacatattagcataaaagccatcaatgtcattctccccacaatagtaaacacttacctttttaagtagttgatctttaatccagacataagctcaaacaaataaagcagcaatttgatgttaacagctttagaaggatcatgagtaaaacacaagaccatatcatccgcacattgcaagatagcaactccactctcaattaaatcagcaacaaacccctggataagaccttttttgtgcgcggcaatgaccattttagtgagacactcagcggccatattacacacacacacattcatacttaatgattatgtcttgtgggttagggttggggtgctcagatgtggtttgggaagcgacaaggatggactcttggtaccccggcgaattgtctctgttgcccctccgtgtctgtcacctagatgggccaaactcactgtttccatccttaatcatgaatttagcacgaaatgacccatttctgaatgtatttcacgaccagattcttttatttaccaccaatcacggcagcaacggtagatgagctaccaccagagtctaacggcgatagttctcaatccaggcccggtccgcggccgcttaccaagatgatgcttctactctcctcctacttgagtttcaggcatttctgtgtgcagctcagttctgaatgcactgcctccagatatgttcaaggccgataactgtgccttccttgttatttgctgactccagtgtaatcttcaacttccgaatgcattacaaagctcgggaacgtcgtcgtaatgtccctgaacaacatcaaccacaacaacgataaaatcgcgtcaaatgtaatgagaaaaaacatcaaaagctctttactctctagcatagcacaactgctgccactcatctaaatatggcgttcgcgtattatttggagactaaactaagaaagcctaggagaagctgaactaacaatgcttgagaaattgaaaactaaaggaactatcagccgcgagcagtaaggaagagtagagaacagcagacgagagcactcactggagatagggcagcgacatgttcctcagcgatgctgggttcttcttgacgaactcctgccactccttggggtctatcctgccgtcgccatccgagtctgcttgactgaacgtctgaaagtccaaaaggagcagagcagcacagaacatgttagaaggaaaacagggaaatagttcccaatgactgaatatatcttgttgattacatacattgtcgacgatctcctcgacggcgctgtcagagagacataggtcggactcatcaagaagcgccaccaccatctcccggagctgtgcaggcaatgtgttgattataaatttagaattcagaaacaacgcgtcgacgttagtaccttacaataaagtgcaccatgtactaggttggatgagaaatgatttagtacttgcctcttctttctcgatgtagcctgtccccctcaggtcgtacagcttgaatgcaactgcaaagaggacgatgatggagacaaacgaaagatttttgattggtttcacagggaggacaagaagatgaatggacagcaaagtaagagcgagcaatggacaggaggcgtacatgcggtcttttcagattcaggcgctttggggtggaagatgctgagcgagcgcacgaactcgccgaactcgatgaccccgttgcgcttgagatcgaagaggtcgaacaccctgtccgcgaagaggttcgcccctttgctggtcctgaacagcgccagccggaactcctcctgacatgaaccaagcaagcggatgagacgagagcacgcagtctttgaagatggatccatggcggttgtggggaggacctcaactaccttgtggatgaggccgtccttgaagatggagtagctgagcttcttgtagagctcgtacagcgcctccacctcgttcaccgtgactgcaaccacgcacacgcaatgcgacatgagcttccattcagttgaggtagtacagaggaaatcaaggaggagtatgggctgacgtacaggaggtctgggaggcgaggacggtgggctcctcgtaccccggcgtgcg

>*TaCBL4*_1DL_2274485

tttgaacagttcacccaatttcatgaagacatgcgtgattgggaaactcacgtgcagctgcaaaatgatttggttgagcatatgtgggctcatgttggcaaccaataaatgtatcatctttttattcgtttgcaaaactatgtgaaacatttttatttgtatccggcttgtaaaactataatatttttattcgataaaactatgttatttgataatatgtttgaatacaaatcaatgtaaatattgggcggccagccggccacgccggcacatatgggtcggcgcgttgggcgcgctnnnnnnnnnnnnnnnnnnnnnnnnnnnnnnnnnnnnnnnnnnnnnnnnnnnnnnnnnnnnnnnnnnnnnnnnnnnnnnnnnnnnnnnnnnngcccgttggagttgctctcatggactcctagattttcgatttgttttgtccatccaaattgaatgtcggcagagggtgttcgcacatggtaaatgagctgaaccaaccaaccagcagcctttccgcgcatgatggaaatttccagtgcaagcaaaacaaggtggagtgatactaactacacagctttcttaaaagtgaatgaaacatgccaccaccttcctacaaacgcgggatatatatgcgtgcatgcggcggggcatcctgtcatggaaatttgctgcacaagtccaactggtctgctgctgcgcaccaccaatgtcactttcagaatagttgggtaaaagaggcaagaacacgagcacaaaacaaaatcaagtttttcctttttggatgtagaatagagaattttgtccatttgtttgttgtcgctggatttgattccacatttcttttaatctgtcggcgtcgtaaaactcagatcgggtctcggcttgtgcgctcttggtttggtggtggtgcaggggtgacgtggttcgaggtggacctggagcgcaagaaggtggtggtgaccggcgacgctgcagagcgtctccaaggtcaagctcccttgctctctggtctctctcaagctcgatgggagcacaggggattatcatatattgccgaaaacaatgctgacatcgtttgttttacaagtcaacggaagacaagttttacaagtccaattagcgattttactacaacaacctacgctagacactcgtcactgcctagtcacctgctcgatcggtgttttgataacctctcgtacttactacggaggtacaaaaaaagcccatttcttagttaacctcagacagtctgatctgatgcctatctcgcatgggatgaagtcatcgtccggtggtcatcatctcacgcagtcacgctcaccggcagattggctcccttgcgttctcctcccattcgagctccaggccttccggttgcagctcacgcacttctgaatgcacggcctcccagtcccaagagcatattcatacatggccggggacgggtcatgaggcaggcaggcaggcaggcacggctattatttgctgattccactgtaatcgtcgacttccgaatgcatcacaaagctcggaaacgccgtcgtaatgtccctgaacaaacaacatcacccacagcagcagtcagatcagttgaatatttggtaaatggcaaaagcaaatgaagatctctactgctctccagttcacatatggcgtttgtgtttcagaacaagcgcgccaagtgtattacagaccatccagcccataaaggagaagcgtaaaggaacttggagagcctgagaagctgaagagtagagcggcacatgagtactcactggagatagggcagtgacatgttcctgagtgatgccgggttcttcttgacgaactcctcccactccttggggtctatcctgtcgtcgccattcgagtctgcttgactgaacgtctgacagtccaaaaggagcagagcagcacagggcatgttattagaaggaaaacaagaaaacacttctcaaagagagactgaataaatacatggagacaagtatctatctatcttgttggtcacatacattgtcgacgatctcctcgacggcgctgtcggagaggcacaggtcggactcgtcgaggagtgccaccaccatctcccggagctgtacgtgtcaacaacagttgaggcaatcagttcaccaaaattcatcaggataggtcatggacaatgcaaataaggtgcaaacttatttagcagtacctcttctttctcgatgtagcctgtccccctcaaatcatacaacttgaatgcaactacaaaataggatagtgacgaatgagagagatggcaactacaaatgcaaataaggatggtgacaaaccagagatgttgatttttgcagagcaaagacgacgaatggacagaaaagactagcagacaagaggcgtaggggcatacacgcggtcttgtccgattcaggcgctttggggtggaagatgctgagcgagcgcacgaactcgccgaactcgatgaccccgttgcgcttgagatcgaagaggtcgaacaccctgtccgcgaagaggttcgcccctttgctggtcctgaacagcgccagccggaactcctcctgacatgaaccaagcaagcggatgagacgagagcacgcagtctttgaagatggatccatggcggttgtggggaggacctcaactaccttgtggatgaggccgtccttgaagatggagtagctgagcttcttgtagagctcgtacagcgcctccacctcgttcaccgtgactgcaaccacgcacacgcaatgcgacatgagcttccattcagttgaggtagtacagaggaaatcaaggaggagtatgggctgacgtacaggaggtctgggaggcgaggacggtgggctcctcgtaccccggcgtgcg

>*TaCBL6*_5AL_2189286

ctcctcgcgtcgcggcgcggggatcccgatccgtggaggggtaagcgcgtcggaggagagcgcgattgaggcgagaatgtgcgcggaatccaagggatttctatatctggcttgatttcgcgtttggaatacacgcgggttgggattttttcggttggcaatcatgcatgcttgatccgcatctccttgttgatcgtcgcaggtgtgtgtgagccgcgttgatctggcttctggtggagtgtgaaatggtggatttcccggaagggttgcgacggctcgctgctctcctgctcaagtgctgcgatctcgacataccaaatcgtcccaagggcctcgaggatcctgagcgtctggcaagggaaacagtttgtatgtttcttcgtctccagtttcttacctccatttaccggatcagccaatatctcgtacctccatttatttataaattccttgttgtgcagttagcgtaaatgagattgaggcattgtatgagttgttcaagaagatcagcagtgctgtggttgatgatggtgtgattaataaggttagtaacttctgccgatttcgatgggtctgtagtgtggttgttgacaacttacactccatcgagctcaatctcataccaaggccttgcctgttgtatatgcaggaagaattccaactggcgctgtttaagactaacaggaaagatagcatgtttgctgaccgggtatgtctgcgtggcttctgttttttatgttgtcacaaaaagtccatgttgttttgttcgtgatatttggttggtacttgttttatttgttcttttgtgctattggttatttggttcacctttcatgtagcgtttcgtacttatatactgcatggaagcatcttgaatgcagtatgaacattggcagcgaggcagttttccgtggagttatctaaaacactataagcttgagctttgaagttttggtcatttgccatgtcacatgtgttatgtgcttgggcttgcactcccacatgtctccagttgctgacgcagcatttcggcatttcaccatgcactagctgacttttgagtagctttaggctgtcataagttattgcagcctaaaactaatgctaattgagctctaatatataataatgatataacgaaataggaaaaaccttaaaacaataaacatacatagtagataagtacacttgtgtatctaaattatgaatatttattccatcttatgctattattcctatatcaatattgtaatgagaaccaaaatctgcaaaggtaggcagtaaatcaaaatctaggtatatttactctaaaaacatggttatatataaatatgaagtaggcaaatttgcaaggacttaccacttcctgctgctgttacaaatcattacttcgtgaacatggacatataacacaattctttttgatgatctgatgtcaatacttattgatgctgtactttaatctatttaggtgtttgatttgttcgatacgaagcacaatgggattcttgaatttgaagaatttgtgcgagctctttctgtattccatcctaatgcaccagttgatgacaaaattgattgtaagccatcatgctttatttttttcatttattttacctagataatgtgacatgtttcagcgaggattaagtctaggttttccctcttcgatgcgacaatttttcaagacatggatcttcaattattgctgcttagcaaatgaatatgaaaagttagctggctgttgacttcactggcagatgcacacaaactgttatcattgatatactcaaccctgcaatttagacgaactatcattggaatcctagtggttgcagcttgcagccagacactatctcttttaatttagttgtatgtggagcacacaaactgttatcattgatatactcaaccctgcaatttagaataactatcattggaatcctggtggttgcagcttgcagctagacactatctcttttaatttagttgtatgtggagaccaccctggaagtgaggggccgagggagggtggtgtgtagatacatatagatccctgtcttaggcagttcatacacactcatctgttattacttaggccattttgcgttcaattttcttctttggtttcttgcacgcaagcacatgaatgcccttttgtctcaatctcttcttgagttcatgtatacatagcattggcatcagtcagaatttatgcttgtgtcgtactaaatcatctgttcttctaatcttgtgccatatatgaactacctgcagttgctttcaaactctatgatctcaaacaacaaggtttcattgaaaagcaagaggtactaccttcatttttcatattgtgaactctttttctgttctattgtattttgttatgagttagtagtccatttgttcatactgtcaatcgtgttcctgttcaaggaatttattttagtgacagtgaaaagtaagtgtggtgagagagctatacatgtcagcaataacttaatcgagttaaccgtgtgaagcactcgtacatagaaactgacgcccttctgcaacccttccatcttagggtgtgtttggttgaggaacgaagtggaacggaatgtcatggttccattcctttggaatgggtcggttccttcttcgcgtttggtaggagcaattagatggaatggaatggttatgttttattgtttggtatgagagacagaatagtatgcatttggtgtaggaatggtgagattattacttatatgcttgtgcttataacaattggttcaactttgacatcaactgacgcatacagtcgaaaatcgtcaactgcaaattgctcaaaaaaaaaaattctataaacattaaattggatttcaattggaaaaaattgcaaaattaagcggcatgcacatacgctggagcaaagatcactctgtataaagctagagcggctgcacaacagctcttcttcttgacaggagtggccggcgcatacacacagtggtgttcatgtccacgcgcgtgcagatgggggccatggggcgggtgctactgcttgacgaggtcccaatttgtagaccatcgaacgcccacgccggctaccccttttggccattgtgcctctcttccatgcgagcctccctgtctctctcttacctgcgagccgctgctgtcgccaggagtg

>*TaCBL6*_5BL_10906286

cccccttggccgccgccccccttggagattggatctcctagggccggcgcccccctaggggccctatataaagaggggggagggagggcagccgcacctagcccctgacgcctccctctccctcccgtgacacctctccctcttgctgtgcttggcgaagacctgccgagatcgctgctgcatccaccaccacgccgtcgtgctgctggatctccatcaacctctccttcccccttgctggatcaagaaggaggagacgtcttcccaaccgtacgtgtgttgaacgcggaggtgccgtccgttcggcgctagaatctccggtgatttggatcacgacgagtacgactccctcaaccccgttctcttgaacgcttccgcgcgcgatctacaagggtatgtagatgcactcctctctctcgttgctagatgactccatagattgatcttggtgaagcgtagaatttttttattttctgcaacgtaccccaacaatcggatcacttaagggactaatcaccttgcatgattattgctggactacagaccaactagctcgccgaggcctaacaaataatgagatttgcgcgtcgtgtgactaggagcatgaagatatccatcatctccttgtgggctactccttctcataacaagtttggcacatcactctgtcttcgtgcgagtcgacaatgatgccaccagacctagctacctccttcttgaactagtgggttgctcatgcctccatgcccctactttgcatcaccgaggaaactcctcggtcatcattctttgtgtgtgctagctttggaagcaccacaacggttgcatcttcgacgacatgaacccttcgaccacaaacatgttagagtccatccaacaggagacacgtgcttgggcaagggctggtgcatctgggatagacaaaattctccccgagacatagaccttagattttttttgggatgaccgtcccttcctcgctgctctagtcatattactcacgccattttctgcatgtgttgtatgatgtagccccacccttctactttcttctatcaatgcaaagatatgcaagctcttgcatatttgtgagaaaaaataactcgcaaaaaagacgtggcatcactatagccgagaaaagtaaacaataaggcccactaagcatgttatgggctccggcctagtacacatctactccctccgttcggaattacttgtcgcgaaaatggatgtatctagacgtattttagttctagatacattcatttctgagacaagtaattccgaacggagggagtagaagggttgaccccatcatcaaaacccaccatccatggtaacatcatcataccctccgcagaatattggaataggaaacaaatcatgcgagagctatacatcgcttgtattgagttttaatacggctcaccacaaggtttcactgttgaaccattttctatgccggcccattagtgtgttctaccgctgtagtcatttcgttcgctagtagtagtcattgtagattgaattcctacctcttcttttgttgtacactagctagagttgtctagttttatttgtttgctttccttctttctttgtgtatttgtttcatgggttttttgggtttcaatggttttccttctttattctgtgtattttcttaaaggtttcattggtttttcttttcttttgaattgtttttttccttcttcctttttctgtacctgttttcttggttttataggatttttttctttttatttcttctgtgtgttttctttatatttttcattggttttcatagtattttttcctttttgtcttctgcgtgttttctttatggttttcattggctttcatttttagtttttgattttttttgtgtcactttaccaattttcttcggttttttggtattcttatttttatttttttatcaacgcatgttacctctttctacacattgaacatttttttctatagaacaggaatcttgtttttatatatatttaaaaaaaatctaattcatgattcatttttttcaaatatatgttttgcatctattttttcatacacattgtacatatttttgtatatataaagaacattttgtgtacatgtttaaccttttttcaaataaatgaacaacattttcttatatttatgtttagatgtctacttttttcatatatttttcctatacatactaacttttttatacatgtttaacattttcaaatatgttattaacatattttcaaatatatgttttgatgtttactttttcatacacactgttcatctttttatatagctgaaacattttgttggtacatgttttacattttcaaatacatgattaacatatttctcaaacacatgcttttgaacattctttcgaatacgtgttaagtatttttgaaatacaagttgacacatttttaataagacaaaacatttttcttaaactatgctgatctttttttacattctataaacaattttctaaaagtcacgcacataatttccgaaacatgtgaacatttttctaaatgcaacgtactttttgtttgaatggtacaattttttttaattacacgaatattttctgacattgtataaatattttctagatatgtcatgtacaatttaaaaaatatgtcatcattttcttaatgtcacaaacatttcaactgttatcctcgctaacagatgttttatgccgcgttaacattttcccaattcgcagtgttcaattttttataaactaaattcaagtttttgaatatatgtatttagatttttttttcaaaaatatgaacacaataaataaatgaattctatctctaacagaagctcatgtacatatttgacttttttctaataggcacgtacacaagattactagcaattgatattctttttctagaatactagtaatcgatattcaacaattctttttgcgagtaaaacttttgatttattcatcaactgaactgtaaaggtagtacaaagaacaccaaaagtaaaaggtatattcaggttcatagaccatttattgatgactacaagcactagagtaagccgaaggcacgacgctatcatcgcccctccctcatcggagctgggtaacttgttgtagtagacagttgagaagtcgtcgtgttaaggccacataggaccagtctaaagagcaaccgccgtcgatgaagagaagcatagattagaatgatccaacctatagacacataaacacagacgaacaaagaccggatccaagtaaatacaccgaagacaaacgccaaccgaatcctgtgatatccatcggagacaaacctccatgcgccctccgacgatgctatgaacaccaacggggcatgggctagcgggaggaaccttattccatcttcagagagtccgccgccgcctcatctctccaaaaaaagagataaaccttaataaaactcaaaaagtaacaaaaaacgaagccctctcgctggtaagggccgggatccaccgcgccttcatgaccctaagaccatagaagacgaggtagaccggctgcaacgccacgggaggcacaagaaaccccaaaaggtatgggcatcccgtgtgtgtattgatattcaacaattcatacgctacacgcctactcttatgcatggtcgtgtgtatcgataaaagtacttctcttatgcaggttatctaaagatgtaccgagactcttcgtttatggtcctctctataatgcgtggagaatcgagacctgtttatggaggcgtgtatacgattgaaggctacgacgagggatacttttacccaacatgggtggcaacataatcttaggattggcccccctctgctttaggcgttatacggatactgcatgttttccttgtatttcgtctttttatatttttatttgaatgagaggacattgtttggctatgtgcatcttagctatgcagaggccgggtgtaatgcttaaatctttgtaagtaataaagtgctcatttttgaaaaacatgcctactttttttttccgagttgacgtcaacatgctcagtttagatttcttttttttaggaaaaacatgcaatgcttcattgatcaatggaacggtttacaagaataagcagagtgtcaaagggttgtagaagccaaatatggcgccttcacccaaatgcaaagcatgtatagctaaactatgcgccttcgtattggagcgacgtccttcatggacaaaagaaatagcatgaaaatcaccgcaatcgtcctttatttctctgacgattgagccagtgataccatgagttcctgagttgacggcggagatatagtccgaggccactgtgatgttttggagtagcaagtcccttcccagcgatagcgcctcacgacacgttaaagtcttcaaaatgtgtgggtcggtcagcccgtgaaaaaccacagccgaagaacccatatacacaccttgtccatcccggcatacagcgctgaccgcaccaatagtttagatttcttttaacgcagtacaatcaaaatcattcacataccaggttggttcgccatgcccattccagttttttttttcttgagtggcatgcccattcagatgctaggtggagccgagtgatttctgttttaatgggccggcccaggatgagcgacgcacccacgatagcaatggacaggttttgctaaaaaaatatataaaaaaacaatcgacaggaagagcggacgtgcgggagagacgagcgatggctcgataggcatcctccccaggttcagatcccgacggcatcctccccaggttccagaaccgctgacgtggcgctcctcttcctcctcctcccgcctctcccatcaccgcctcgtctgcctcaccgccggcgaccgtcgctcccgtacccacactccggcgcagggatggctcaatgaggtgacaccccaactcatctccctaatccccaattcatcaatcttggtgtcatcggccgtcgtcgcgcttctcctcgcgtcgcggcgcggggatcccgatccgtggaggggtaagcgcgtcggaggagagcgcgattgaggcgagaatgcgctcgaaatccaagggatttctatatctggcttgatttcgcgtttggaatacacgcgggttgggattttttcggttggcaatcatgcatggttgatccgcatctccttgttgatcgtcgcaggtgtgtgtgagccgcgttgatctggcttctggtggagtgtgaaatggtggatttcccggaagggttgcgacggctcgctgctctcctgctcaagtgctgcgatctcgacataccaaatcgtcccaagggcctcgaggatcctgagcgtctggcaagggaaacagtttgtatgtttctttgtctccagtttcttacctccatttaccggatcagccaatatctcgtacctccatttataaattccttgttgtgcagttagcgtaaatgagattgaggcattgtatgagctgttcaagaagatcagcagtgctgtggttgatgatggtgtgattaataaggttagtaagttctgccgatttcattgggtgtgtagtgtggttattgacaacttacactcccatagagctcaatctcataccatggccttgcctgttgtatatgcaggaagaattccaactggcgctgtttaagactaacaggaaagatagcatgtttgctgaccgggtatgtctgcgtggcttctgtttaatggagttatgctgtcacaaaaagtccatgttgttttgtttgtaatatttggttggtacttgttttatttgttcttttgcgctattggttatttggttcacctttcatgtagtgttttgtacttatatatggcatggaagcatcttgaatgcagtatgaacattggcaacgaggcagttttccgtggagttatctaaaacactataagcttgagctttgaagttttggtcatttgccatgtcacatgtgttatgcgtttgggcttgcactcccatatgtctccagttgctgacgcaacattttgacatttcaccatgcactagctgacttttgagtagctttaggctgtcataagttatgacagcctaaaactaatgctaattgagctctaatctataataatgatataacgaaatgggaaaaaccttaaaacaataaacatacatagtagataagtacacttgtgtatctaaattatgaatatttattccatcttatgctattattcctatatcaatattataatgagaaccaaaatctgcaaaggtaggcagtaaatcaaaatctaggtatatttactctaaaaacatggttatatgtaaatatgaagtaggcaaatttgcaaggacttaacttaccacttcctgctgctgttacaaatcattacttcgtgaacatggacatataacacaactctttttgatgatctgatgtcaatacttattgatgctctactttaatctatttaggtgtttgatttgttcgacacaaagcacaatgggattctagaatttgaagaatttgtgcgagctctttctgtattccatcctaatgcaccagttgatgacaaaattgattgtaagccatcatgcttttttttccatttatttacctagataatgtgacatggttcagcgaggattaagtctagtttttccctcttcgatgcgacaatttttcaagacatagatcttcaattattgctgcttagcaaatgaatatgaaaagttagctggctgttgacttcactggcagatgcacacaaactgttatcattgatatactcaaccctgcaatttagatgaactatcattggaatcctagtggttgcagcttgcagccagacactcttactcttacgaagccttttatcccaaacaagttggggtaggctagatatgaaaccctttcacgaggacttcccaggaggtcacccatcctagtactactctcgcccaaatgggtttcatacccaaaagactggctagtttttacgttggctcgccaagcctatcacaacccttagatatgaaaccctttcacgaggacttcctaggaggtcacccatcctagtactattctcgctcaaatgggtttcatacctatctcttttaatttagttgtatgtggagcacacaaactgttatcattgatatactcaaccctgcaatttagacgaactatcattggaatcctagtggttgcagcttgcagccagacactatctcttttaatttagttgtatgtggagcacacaaactgttatcattgatatactcaaccctgcaatttagacgaactatcattgggatcctagtggttgcagcttgcagctagacactatctcttttagttttgttgtatgtggagaccaccctggaagtgaggggccgagggagggtggtgtgtagatacatatggatcccagtcttaggcagttcatacacactcatctgttattacttaggccatgttgcgttcaattttcttctttggtttcttgcatgcaagcacatgaatgcccttttgtctcaatctcttcttgagttcttgtatacatagcattcacatcggtaagaatttatgcttgtgtcatactaaatcatctgttcttctaatcttatgccataactacttgcagttgctttcaaactctatgatctcaaacaacaaggtttcattgaaaagcaagaggtactaccttcattttgcctattgtgaactctttttctgttctattgtattttgttatgagttagaagtccatttgttcatactgtcaatcttgttcctgttcaaggaatttattttagtgacagtgagaagtacgtgtggtgagagagctatgcatgtcagcaataactaaatcgagttaaccgtgtgaagtactcgtacatagactgacgcccttctgcaatccttccatcttaatgttactcctttgttttgtgcttggagaaagcatgtctatataacataaagttcagctaggagcaatcaccttgggaaaaagtcattgtgtgccttcttaattgtcaggtgaagcaaatggtggttgccacacttgctgaatcaggaatgaacctttctgatgaggttatagagggtattatcgacaaggtatacttagagtgtacttttgctatgatgtactccctccgtccggaaatacttgtcggagaaatgaatgtatctagatgtattttagttctaagatacatccatttttgttcatttctccgacgagtattttcggacggagggagtatgtctgttgaatttattcttttgcttaagggggcaattttttttcttcatcactagacatttgaggaagcagatacaaagcacgatggtaaaatcgacaaggaggaatggcgcaatcttgtcttgaggcatccatctttgttgaagaatatgacactcccatatctaaggtcagtatggctgaccctgtttgtgccccccccccctaaaaaagaacttgagtcaagttgtcttactgatcagtacctacttcatctccgtgggatacatgtgtgaatctcagcatgctaaattcttaaggatcgatttgctctgcattttctgattcacagaacatagcccatttaactttggagtactataattcaacttgctcgattagcagaggggcacagcatgtgaaataatatagcacatggttgtaactcaaaactagatcgaaaatgctgatgtggttcgagattaaacattcagagtcagaaaacaggtccctttattttgcacagcagtaacaacagtttgattcccttcttacggcttgattaataactgtggatttgttctaattataaaatacttcatcaacagggatattaccacaacatttccaagctttgtcttcaactctcaggtcgaggatgcttgagaatccagtttctaggttaccactgaagcttgtgaaaggccacggcagaccatgtcgtaggtggaccttgtgtatttttatttctttattcctttttggcgatatccttcttgtgcttggaacaattcttgtaattttttcacttctttaaaatgtgaattcccagcaatctcctcgtgtgagggctcttcttttgtatatacccgagtataatctagaattaactgttccttatgtataaacgctcttcacatacctagttgcccatgcgagtagcaactgataagcagcagttatattttatcgttctactgtttcgttcgttcatttggttcttgtggtgttgcttgttactcccgtgccatcgttcagttttggtggctgcatagctggaagcttttctttctttaaggtgaaatatacgttacgactttggtcacgccttgatattcttcaggtgaagctttgtccttttcatcttttactttgcacatttacattgtttagcatgccttgtccgaagcaagtgcctgcatagggcgtttgtgttgacatgattgctcttcctatgtgcggtgataattgcccatccttatgtttcttggtcttaacatggccgttggtcatggttggttacggtggtaagaaccggtgtgtacatctgaagtcaaccttagagcatctatagccggactgtgcaaatccgcacccctaaacgtctgcggacgtgtccccggatagcttgcttctcatttgtcctcccgagcaagcacaccctattgaaacccttgaatccaaagcacacccctcatattgaaacccttgaatccatgcaggtccatcatatatcataaattcatcaccaaattagcaatgcaaaaatggatgcaaaataaagcacaattcaacacatgtcaaaagaaaatatgacaattaataaacaagtgatacaagaatgaatcattcggtgtttgtattgcgcttgttcctctttcgtctcaagtcacttgttgcctttgggatccaagagggaagtgttgggcaacatgatcttcgaatcctccgtgtcccgggctagttgcaacctctctttctcgagctcaattgatccaaaagtctttgccttctccaactcctatttgacatacgcctcctccaagtcgattctccttctgaagctgcaacttattgttcatcctctcctgctcctaaccatcttttctttttgcgcatccaagaagagctttctatctcccttccttcttctcctccttagtggagaaaatgctcgtccatgtgggggacattttgctcattgtggcgtcacgcacggccctaaccttctcccacttgttttccatcacttagcggttcatgtttggcacgatggctaggacctcctcccctccaccttcctcatctttgtcatccaatccaatagacagttgggagcttgagccatcgtttttcttcaaattcttgttgttgttctcgaggtcatcgatgagttgttgccactttggtttctgtaggatcgcaagtaggtctagagaggggggggggtgattaaattacttgaccaaataaaacttagctttttcccaattttagttcttggcaactttcaacaattcacacaagttaagcaacaccctacacatgcaagtctagatagtaggtagcggaaaataaagactttgcatatgaacgtaaaggaggggattggagatatcaaacgcaatgaagacacggagatttttggcatggttccgataggtgctatcgtacatccacgttgatggagacttcaacccacggggtaacggctacgtgagtccacagatggctccacccacgaagggtccactaagaagcaactttgtatatgccatcatggcttacgtccacgaaggactagcctcacttaggtagatcttcatgaagtaggcgatctccttgcccttacaaactcttggttcaactccacaagctttggaggctcccaactgacacataaccaatgtaggagacaccactctccaaaaggtaataaatgttgttgttgatgatgacctctttgctcttgtgcttcaaaagatagtctcctcaaaactcagccactctcccacagatttggcatgcatggtgttgggagaaggattggagtggaaatcaacttgagggggctagaaatcaaggatcaagtgattggagtggaattccttgatctcaacacaagtgtaggtggttctctctcagaaaatggacatgggaagtggtggcttcatcctggttgctctctctgagagtaggtggtgtgggtggggtatatatagctatcaccaaagatctagccgttacacacctttatgcacaacttggtggaaccgggtgaaaaacttggtgggaccgactaatgtaaaaggtttgaactttagacgcttctgtgagaccggatgaatccacttagtgggaccgattagtgatgacctaagaactttcaaaatcctcgtaattccaatcgttttcactcggtaattctgaaatgaacacaatgagttacagagagttggcacacacattttggtgggaccgagtggcatcttagtgagaccgagtttgtagggtttaggcagcggctctgtccagtgtatctcgatgggtccggatgtaagtgtttcagtgggaccgttaactttagggttttggacaaattggattgtgagggtgtggctggggtttttggagcaatatcttcaagaacttgagtaaatgaatcaataattgctgcccttttgcttagttccacatatagggccaatgcttgtggccaaccaaacttcacacaagaaaacatcatgcttgcttgtcggacctcttgtctttggtatcttttttttctgtttacgagtggtcggtgtttacccaacatcatatgtaggagagttgatatcccccaagtcataatgcataggctcttgaccatcattgatcatgtccgtcatgatagcccccctaaaatgatcatgatcactaataatgtatgcaacaaaaagatttgatggcatatgcaatgctaaaatgattcaatggcatatgcatccctaaactgattcaatggcatattgaacaagcagacgaataaatagaaagaggggcaaaaattataccgtgtcttgctccatcatttcattgaacaagacctgggaagatccggagtcagcgattcccgaagctattctcgcccattcgagttgcgacgagtcatacaccacctctagtgcctcctgttgcgtccagccatgcgtaactagccaatcgtctggccgaacggagtcggtcatgaccagggcggatgacaccttccttctaggcggcggagtggatgcgaacttgatcgcgaccggagtggacgcatccatgtcggcgtcgaccacctccttgtctgtagcatccttcatcgcagcttcccactcgcgccgctttctttgccgctccctccaggtgaagtacttcgccttgcaactcaccctcatgacgggcccaccatggacaacactgatgcatcatctatgtcgttgggggaaggggatggggtactcagcgatggcatatacgatggctcagatgcgggcaacaagggtggacaatgatgatgtctcggatgcgggaaacggcggacagttgcgagaggagggcgactgattttggtggaataggtcaatttggagggatttggcgtgggtccggtctatcgaatctgacgtggcggacgcgttcgaggtgcccagacctccccatatccgccccagatatgggctagtttgaggggtactagacaacgcaaacatatagggccggcttgaggggtgatttcaggggtctggctatagatgttcttaaggtatgatccacccaatattgtttagcaagctgaaaatattcccaatgcactcgagtgcttaagactcctttgggccagctggagtccatgtgtgaattgttttccttttctctctcttcaaccattcaaattcatgattttgcatttacatgaagaggagagagaagtacttgttttctatgtgctatgggttggtcttagtgcaaagttcggtcttaaaagtcaaaactttgcaaacttgaccaattttatggtatcttcaatgtgaaaagacatggatcttataagcccaaaaatagactttttacccagcctcaagcattatcagaagggttcttagcaacaaaatcttaagactgggtcatagttctaagaccaggtaataaggaatttaaaaacaatttctattaagcatgtgggctccaccctaagactcataggggtattgagcattgaagaattcttgtttgctttagcacattgagtggggcctattcattcgtcagtaaagtttctacatatagaatcttttttgcatggatagaatattaaatgcatatatacatcattagacattttctcatgttttatatatttagttttattgatatagatagtttctttataatcttgatcaggtgaacgaatcttgacttttgaaacatttgtgtgcactttattatgaaacaaaacttcacaaacatttttagcccacctttttgcattataaataatctctccagacgacctcaatgttctaactcattccggaaattgttttgagctataattaacaatcaattcaatgcactactagtcaaaactagttaagatcaagcaatcacaagcaaattgcaaaatatctaatagttcaaatcccaaaacatggaaaatttgggatgtaagggaatctcctactctgaccctcaaatcgcccgcaaaagtttgggccgagacatatggatgttttttgccatccacagcggtgcccaaaacatctgtggacgtgttcgctcgccctaattctcacaaaattgttttgagagggagaggggggttgcgggcgtccagactttgtgccacgctcggcttcacccaaagtcatcaccacacgttctgctctagaacagtcatcgcgcattcatgccggtctgccctccaaagcagacgcgactcgacggccttgatgctggtcagagtgacataatcatacgggagggaagcactgcttcaatgcagatgtggcgtccgaaaaacttactccagccgctgtgtcgcattgaagcggtgtcgctccgcatgccccaactttttaagcagggctttggtgtcccaaaatgcatctcatctctcggaccccgctgcctggctcctctcctatcctctcaccaccgtcacctctcctctatcctctgtagtgatagcaagcccggctagagcagctggttttttatgcgggcggtggatctgctctccctctgaatcggcatgccaccgccaatgttctatgggaccttccttctccaccaccctgatggacaacgaggagatcatcctatatacggcgacgaggaggaccaggcacagccccgcctggtcctcctcggaggccaccgcgacagacgagtaccaggcccattcggcgcgtgcctcgtcgcctgtgcaccacgtgaaggaggagccattgtcgttgcctcaccactgcatcaagcatgagccccttcccttggtgcaaccctggcgtccaaatcgaacaccgcgtcaagggcgagccggcatcatcgctatcccatttctggcacggcaatgaaagggtgccaccatcaccgcctcgtcggcgcccggttggtgtgaagcaggagccctccctagcgtctgcaaacacaagcatgcgcctcctgcattacctcggccgcgagggcggcgttaccacattgaccaaggaggagcgagcagtgttcgccacgtcgcgccggactgggtcagtgttagactatgtatagcttctgtacctatgtacgtatatggtacatattgtaacacaaccactatatataatgagataagccacccctagagggttgtgctggttccccaaaacttattgtcttacatggtatcacgctaggttacgatcgcttccgcttctaaaccctaatacccgcaccgccnnnnnnnnnnnnnnnnnnnnnnnnnnngccgccgcgccaccgatcgcgccgccgccatgtcgagcgccgccaccaccggttccactgctgcgggcttcctcccggcctctcttgcggctctgctcaacctcccgctcgatgccgtctctgttccggctccgatcgggacaaggagcatcggctccgtcttctccacgccgccggcg

>*TaCBL6*_5DL_4595540

tccaaatgaggtgcggttttcgcccacacgatcgtgatcgaacgacctacagcatggaagagattttggtgggttttgggctggtttttgagggggtttttgctggaacacatagaaggcatctgcggttacccggttaaccgttggagtaccaaacgacctccaaatggaacgaaacttgaccggtggtctccgggtggtatactaaggccacttgacaagcctcggtccattccgagaatgtttgatacccgctcacgaaagaaaacaagaggggtgcaccggaggagagaggagcgccggattgcaaaacggacaacggggaaaatgttcggatgcaagaggcgaacacgtatgcaaatgtaatgcacatgatgacatgatatgaaatgcatgacatgaacaaaatgcaaaacgaaaacaaaacccgaccacgaagggaatatcatatcacatagccgaaaatggcaagagtcggagttacaaatatggaaagttacatgcggggtgttacacaacagctcatcgatgacctcgagaacgacaacaagaaggtgaagaaaaatgatggctcaagctcccaacaatctattggattggatgacaaagatgaggaaggtggaggggaggagggccaagccaccgcgccaaagatgaactgctaagtgatgggaaacaagtgggagaagggtagggccgtgcgtgacgctgcaacgagcaaaattgtcccccacatggaagagcattttctccaccaaaggaggagaagaaggaagggagatagaaagctcttcttggatgcgcaaaaagaaaagatggttaggagcgggagaggatgaacaataagttgcagcatcggaaggagaatcgacttgaaggaggcgtatgtcaaataggagctggagaaggcaaagacttttggatcaattgagctcgagaaagggaggttgcaactagccccggacacggaggattcgaagatcatgttgccaacgcttccctcttggatccgaaaggcaataagtgacttgaggcaaagggagatgaacgggcgcaatgcaaacacccaatgattcattcttgtatcgcatgtttatcaattgtcatattttcttttgccatgtgttgaattgtgctttattttgcattgctaaattggcgatgaatttgtgatatatgatggacctgcatggattcaagggtttcaatattaggggtgtgcttgccgagtaggacaaatgagaagcaagctatctggggacacgccggcggacgtttaggggtgcagattttacagagtccggctatagatgctctaaggttgacttaagatgtacacagcggtgcttaccaccgtaaccaaccatgaccaacagccatgttaagaccaagaaacataaggatgggcaattatcaccacacataggaagagcaaccatgtcaacacaaacaccctatgcaggcacttgcttcggacaaggcatgccaaacaatgcaaatgtgtaaagtaaaaagaaaaagacaaagcttcacctgaagaatatcaaggcgtcaccaaagtcgtaacgtatatttcaccttaaagaaagaaaagcttccagctatgcagccactaaaactggacgatggcacgggagtacaagcaacaccacaagaacaaccaaatgaatgaacgaaagagtagaacgataaaatataactgctgattatcagttgctactcgcatgagcaactaggtatatgaagagcgtttatacataaggaacagttaattctagattatactcgggtatatacaaaagaagagccctcacacgaggagattgctgggaattcacattttaaagaagtgaaaaaattacaagaattgttcaaagcacaagaaggatatcgccaaaaaggaataaagaaataaaaatacacaaggtccacctacgacatggtctgccgtggcctttcacaagcttcagtggtaacctagaaactggattctcaagcatcctcgacctgagagttgaagacaaagcttggaaatgttgtggtaatatccctgttgatgaaatattttataattagaacaaatccacagttattaatcaagccgtaagaagggaatcaaactgttgttactgctgtgcaaaataaagggacctgttttctgactctgaatgtttaatctcgaaccacatcagcattttcgacctagtttcgagttacaaccatgtatattattcacatgctgtgcccctctgctaatcgagcaagttgaattatagtactccaaagttaaatgggctatgttctgtgagatcagaaaatgcagagcaaatcgatccttaagaatttagcatgctgaacttcacacatgtatcccacggagatgaagtaggtactgatcagtaagacaacttgactcaagttgtggggtgggggggacaaacagggtcagccatacagaccttagatatgggagtgtcatattcttcaacaaagatggatgcctcaagacaagattgcgccattcctccttgtcgattttaccatcgtgctttgtatctgcttcctcaaatgtctagcgatgaagaaaaaaattgcccccttaagcaaaagaataaattcaacagacatacatcatagcaaaagtatactctaagtataccttgtcgataataccctctataacctcatcagaaaggttcattcctgattcagcaagtgtggcaaccaccatttgcttcacctgacaattaagaaggcacacaatgactttttcccaaggtgattgctcctagctgaactttatgttatatagacatgctttctccaagcacaaaaaaaaggagtaacattaagatggaaggattgcagaagggcgtcagtttctatgtatgagtgcttcacacggttaactcgatttagttattgctgacacgcatagctctctcaccacacttacttttcactgtcactaaaataaattccttgaacaggaacacgattgacagtatgaacaaatggactactagctcataacaaaatacaagagaacagaaagagttcacaataggcaaaatgaaggtagtacctcttgcttttcaatgaaaccttgttgtttgagatcatagagtttgaaagcaactgcaagtagttcatatatggcataagattagaagaacagatgatttagtatgacacaagcataaattctgaccgatgcaaatgctatgcatacaagaactcaagaagagattgagacaaaagggcattcgtgtgcttgcatgcaagaaaccaaagaagaaatttgaacgcaacatggcccaagtaataacagatgagtgtgtatgaactgtctaagactgggatccataagtatctacacaccaccctccctcggcccctcacttccagggtggtctccacatacaactaaactaaaagagatagtgtctagctgcaagctgcaaccactaggattccaatgatagttcgtctaaattacagggttgagtatatcaatgataacagtttgtgtgctccacatacaactaaattaaaagagatagtgtctggctgcaagctgcaaccactaggattccaatgatagttcgtctaaattgcagcgttgagtatatcaatgacaacagtttgtgtgcatctgccaatgaagtcaacagccagctagcttttcatattcattttctaagcagcaataattgaagatccatgtcttggaaaattgtcacatcgaagagggaaaaactagacttaatcctcgctgaaccatgtcacattatctaggtaaataaatgaaaataaataaataaagcatgatggcttacaatcaattttgtcatcaactggtgcattaggatggaatacagaaagagctcgcacaaattcttcaaattcgagaatcccattgtgcttcgtgtcgaacaaatcaaacacctaaatagattaaagtacagcattaataagtattgacatcagatcatcaaaaagaattgtgttatatgtccatgttcacgaagtaatgatttgtaacagcagcaggaagtggtaagttaagtccttgcaaatttgcctatttcatatttatatataaccatgtttttagagtaagcctacctttgcagattttggttctcattataatattgatataggaataatagcataagatggaataaatattcataatttagatacacaagtgtacttatctactatgtatgtttattgttttaaggtttttcctatttcgttatatcattattatagattagagctcaattagcattagttttaggctgccataacttatgacacgataaagctactcaaaagttagctagtgcatggtgaaatgccgaaatgctgcgtcagcaactggagacatgtgggagtgcaaacccaaacacataacacatgtgacatggcaaatgaccaaaacttcaaagctcaaacttatagtgttttagataactccacggaaaactgcctcactgccaatgttcatactgcattcaagatgcttccatgccatatataagtacgaaacgctacatgaaaggtgaaccaaataaccaatagcgcaaaagaacaaataaaacgagtaccaaccgaatatcacgaacaaaacaacatggactttttgtgacaacataactccattaaacagaagccacgcagacatacccggtcagcaaacatgctatctttcctgttagtcttaaacagcgccagttggaattcttcctgcatatacaacaggcaaggccttggtatgagattgagctctatgggagtgcaagttgtcagcaaccacactacacacccaacgaaatcggcataacttactaactttattaatcacaccatcatcaaccacagcactgctgatcttcttgaacagctcatacaatgcctcaatctcatttacgctaactgcacaacaaggaatttataaatggaggtacgagatattggctgatccagtaaatggaggtaagaaactggagacaaagaaacatacaaactgtttcccttgccagacgctcaggatcctcgaggcccttgggacgatttggtatgtcgagatcgcagcacttgagcatgagagcagcgagccgtcgcaacccttccgggaaatccaccatttcacactccaccagaagccagatcaacgcggctcacacacacctgcgacgatcaacaaggagatgcgaaatcaagccagatatagaaatcccttggattcctcgcgcattctcgcctcaatcgcgctctcctccggcgcccttacccctccacggatcggggtccccgcgccgcgcgacgcgaggagaagcgcgacgacggccgatgacaccaagattgatgaattggggattagggagatgagttggggtgtcacctcattgagccatccctgcggcggagtgtgggtacgggagcgacggtcgccggcgctgaggcagacgaggcggtgatgggagaggcgagaggaggaggaagaggagcgccacgtcagcggttctggaacctgcggaggatgccgtcgggatctgaacctggggaggatgcctatcgagccatcgctcgtctctcccgcacgtccgctcttcctgtcgatttttttttagcaaaacctgtcgattgctatcgtgggtgcgtcgctcatcctgggccggcccattaaaacagaaatcactcggctccacctagcatgccagtcaagaaaattttctatttctcaaaaaaaaaactcaagaaaaaaaatctgaaatgggcatggcgaaccaacctggcatgtgaaccgactttgattgtacagtgttaaaagaaaatctgaaccgggcatgttgacgtcaactcggaaataaagaagtaaaggcacgtagcatattaattcttgaatatcaatatacacacgggacgctcgtaccttttggctcgactgagcacccagccgccatagcgtttcttgtgcctcccgcggcgttgccgccggtctacgtcgtcatctgtggtcttagggtcatgaaggcgcggtggatcccggcccctgccgacaagagggcttcgttttttgatattttttgagtttattaaggtttgtgtcttgcttggagagatgaggcggcggcggactctctgaagatggaataagattcttcctgcttagcccctgtactggtggtgtttatagcatcatcggagggcgcgtgaaggtttgtctccgatggatctcacgggatccggttggtgtttgtcttcggtggatttacttgggtccagtctttgtttgtttgtgttcatgtgtatacaggttggatccttctgatttatgcttctcttcatcgacgggcggttgctcttctgactggtcctatgtggccttaacacgacgacttctcaactatctactacagcaaggtttacccagctccgacgagagaggggcgatgacagcgtcgcgccttcggctcgctccagtgcttgtagtcatcgataggtggtctacaaacctgaatgtactttttacttttggtgttttctgtactaccttgacagttgatgaatagatccaaagttttcctcgcaatttttttattgaatatcaattactagtattctagaaaaagaatatctattgctggtaatcttgtggacgtgcctattagacaaaagtcaaagatgtacatgagcttctgtgagagatagaattcatttttttttgtgttcatatttttggaaaaaaaacccaaatacatatattcaaaaacttgaatttagtttataaaaaattaaacatcacgaatttgaaaaatgttaacgcggcataaaacatatgtaagcatagttttataaaaggttgataacagttaaaatgtttgggacattaagaaaatgatcacatatttttttaaatgtacatgacatacttagaaaatatttatataatgtcataaaatattcgtgtaactaaaaatatttgtaccattcaaacaaaaagtacgttgcattagaaaaatgttcacatgtttcggaaattatgtgcgtgactttaaaaaaattgtttatacaatgtaaaaaagtcagcatggtttaagaaacatgttttgttttattaaaaatgtgtctacttgtatttcaaaaatacttaacacgtattcgaaagaaagttcaaaaacacgtgtttgaaaaagatgttaatcatgtatttaaaaatgtaaaacatgtattaacaaaatgtttcaggtatataaaaagatgtacagtgtgtataaaaaagtaaacatcaaaacatatattttaaaatatgttaataatgtatttgaaaatgttaaacatgtataaaaatgttagtatgtacagcaaaaaaatatggaaaatgtagacatctaaatataaatataagaaaatggtgttcatttatttgaaaaaaagttaaacatgtacacaaaatgttccttatatatgcgaaaatatgtacaatgtgtatgaaaaaaatagatacaaaacatatatttgaaaaaaaagaatcatgaattagaaacattttaaatatatataaaacaagattcctgctgtataggataatgtacaatgtgtagaatgaggtaacatgtgttgataaaaaaaagaataccaaaaaaccgaagaaaatttgtaaagcgacacaaaaaataaaaaactaaaaatgaaaagaaaacacacgaaagaaaaaaagaaaaaaaacatactatgaaaaccgatgaaaattaaaacacacggaagaaatagaaagaaaaaatcctataaaaaccaagaaaacatgtacagaaaaaggaagaaggaaaaaacaaatcaaaacaaaagaaaagaaaaaacaatgaaacctttaagcaaatacacagaataaagaaggaaaagaaaaaaccattaaaaaccaaagaaaaccataaaaacaaatacacagaaaaataaagagagcaaacagataataccggacaactccacctagtgtacaacaaaagaagagggtgggaattcaatctacagcaaccagaactagcgaacgaaaagactacaacgatagaacgtgctaatgggccggcctagaaaacgcttcaacatcgaaaccttgtggcgaccgtactaaaactcaatacaagcgatgttatagctctcgcgtgatttgtttcctattccggtattttgcggagggtatgatgatgttaccatggatggtgggttttgatgatagggtcaacccttctagatgtgtactaggccagagcccataacgtgcttagtgggccatattgtttacttttctcagcctataataatgccacgtctttttgagtgttgtttttttcacgaatacgcaagagcttgcatatctttccattgatagaagaaagcagaagggtggggctacatcataaaacacatgcagaaaattgcgcgagtaatatgactagagcagcgaggaagggacggtcgtcccaaaaaagtctatggtctgtatctctgtgacgcccggataattaagctacagtaattctacactaatgatgccacgtcacctcggttactgttgttaatctcgcgttagtttaaaaccgattcaaatttgaatttgaattaaagtcaaacgataaaagttttcaaacattaaaactaaaatgttcatcatgtggaaaatattcatttggtaatattggtggtgaaccaacatttttgaaaagtgtttaaatgccctaaactatcgaaaactgtggcaaacagccccaaattttatcatttctatttaaacaaaatttcaaatgaattcaagtacctctcaaactttttgtggcagtgcctaaaaatgcaacatcaattttggaccaagttccacattttgcaaaaatcatttgatggcttagttaaatacaaacagtgcatattaaaagaaaaagcaaagaacagaaaagttttagaaaaggaaaagaaaacaagaatgaaatcatgcacttggcccattgctatagtaggagcccaacccacctagggccttttcttcaatcttctgttcacaaggagacaaagtgcgcgcgtgtcctacatccatggcatcctggccatggatggcctccacgtcgccccctgcgacctatatctacgtttcccctcaaaccctagtcgtctctcgatcgtttccccctctttccccgtcgagcctcctcctcctctgctcgaaattgagctgggaaccgagtgcggcgaccacgttgttcaccgtcgttgccgtcgcttgggagcatatcggggggatgcgccttcatcgactacgttgccttcgccctttggatcgagcagcaccgcagcaacgccactggatcgacgcgtcttcctcgcctacggccgccgccgccccgcgcccaatctacctcaccggaactcctctgccacgccggctgcgtccttcgcctcccctcccgtgagcgccactcccatttgcctcgaaccccctctgttccgtcgccgtttcgccgtggccgcgtgctcgccgtgtccgcgcgcgcccctgctaggtggcgcctgcccgcgcccatccccgcgttcggcagcgccgccgtgtgtctccgcgcccgtcgcccgcgcccgtccgtgcctccgcgctgcccccggttcgctgctgttgctgctgcactactcagccaccgctgctgctttgcccgccactgctgctccctattgttactgctgctgcccatagccacgcatcgcgtccctgcgcccgcccgtgcacgcccagccgtgctcgctcctatgcgcccctcccacacgcagccactcctggctgagcatggcggccgtggccttgcccgtgcgaactggccatgctagttcaagcacaccgtgcacaactggctgggctctgcccattaattaaggcatgctctattatgtttagtagtaatactaattagattagttgattatttagtctaatagaaataacatgcaggaccaccccggttaaatacatagtatttagttaatccgaagtcaatgacaaccgggccccactggtactgtttatcaatggaccagtcaatgttgactgggtcaacccagtcaacaggccacacatgtcagcctctgtggcaccctgatgtgtacacttcagtgtgtgcacatagcattttgctcttaattttgaattaatataactccagaaaattctaggatatcaataaaactttgaaaattaatataaaataaaccgtaagttagatgaaaatactttgtacatgaaagttgctcagaacgacgagacgaatccggatacgcaacccgttcgtccgccacacatagcgagcacgcaactttccccctccggttcacctgtccgaaaacgcgaaacaccgggaatactttcccggatgttttcccccttcgccggtatcacctcctaccgcgttagggaactcctagcgccgttacttgacatgtcatgcatcgttatgcatcagtttgcattgtattcattgtttctcccccttcttctctctggtagacaacgagactaacgccgctgctcgtgccccgatcgactacgtcgaagacgacccctccttgccagagcaactaggcaagcccccccttgatcaccagatatcgcctattcttctctatacagcttgcattagagtagtgtagcatgttactgctttcggttaatcctattctgctgcatagcctgtc

>*TaCBL7*_1AL_3887581

gccaatgttatcaccggcaagtgggtttttcgccacaaggctcgcctcaacgattctcttgtgcgctacaaggcgcgttaggtggtgtgcggcttctgccagcgcgccgacgtggacttcaccgacacctttgccccggttatcaaaccgggctaaggtgtccgccttcgagggctcttcggcgtccgacgcccccttctaccgctcttcagtatctcacgctgacgcgtccggatattcagtatgctgtccagcaggtgtgtctccacatgcatgctcctcgtgangggctctggtgaaacatattctccgatatatacgtggcaccacggctatgagtctcaccctggcggcatcacctgacaccagcctcgtcgcctactctgacgccgactaggttgggtgtcctaaaaattccttatataacattgttttaaattttgtttccctatttgacactgaataatttgttcttccctatctaacacagtctaaattttaggccctttataacactttcgtccattttaaaccttaaagatgttaaataacatctgaaaagacctatttgtccctcatgtgataggtgaccaaaatattacgtgctagtttagctttaggtcagcagggtagccggtgtacatcaatagtacatggtacactacatactggatcgatatatacagcacgtaagtatgttaaaatcatagtcagtgcttgtaatttttttgtgaaaaggcagtgcttttatacgcacgtacacagctattactaggtatagcctagtatatatttgcatatgcccagtcattttgtaacagccgtagttgagattgaaaaaggaatgtaaataggaccgacatggtcccatggccatcaatcgcatcgtgtgtgttcgagttgtagtcgggattgtatcccaaacttgctttttttacgggtatggctagctttagctaggcaacgaacgtagaaatgatcaagggagagatcggctgctagctaacttcatctattacattttagcagtacgcgtccaactagaatcgatccacctgcctgcctacttgtttcttggtgtatacgcggaaccctcggaaagtatttgagctaacctcgtatgcctgtacgctggaaaacacccggctataacgccatcaaaaggtccaggtcgaggtcgggaccaaggccaacgcaaaacgagtcgaccagatacacacgtacatataaagtttggttaaatatcacataaagggcaaataggttttttcatgtgtcatttaacaccgttatggtttaaaatggacgaaagtgttataaagggcataaaatttaaactctatgttaaataaggaaggaaaagtttttcggtgttaaatagggcaacaaattttaagacaatgttaaataaggaattatctcaagatagtgccgtatgtgcaagttttgaaataccgacgaaagtgcagcaacccacaaattctaaactgaactcgcctagcagatacaccgattgaaaaaattatcaagtttgcactacaacgagtttttgcatttttggaactttggactcctcctaagaattgacacttttaaccatccaatgggaggcacccaacatcagcagcgcgcaccattaagctcctgcgcatggttggctaaccactagaccaataaagccattccataatgattgcaacgcggaacttctaacaactatattttagccgcgctcttgagaaaaatgggatttcttttgaaaagaaatctaaacaaatttcgaatgtccacagattttaaaaaacatcacgaattagaaaaaaaaaagttcatgtattcaaaaaacattgacgaattcaaaaagttcatcaaaattggaaaaggttcatcgattttcaaaaaagttcataaattttgaaaaagttcatcgattttgaaaaaacattcatcgactttgaaaaaagttcattgattttgaaaaaaattcatcgaacttgaaaaaaagttcatcaaatttgcaaaaacttcattgattttgaaaaaagttcatcaaatttcaataaaagttcgtgaatttgaaaaaagttcatcaaaatttgaaaaaaaaaatcatcaaaaatgaaaaatagttcactgaatttgaaaaaaagttcatggaatttcaaaaaagttcatcaaaatttgaaaaagttcatcgaatttggaaaacagttcacaaagccaactcattgaatttgaaaaagttcatcaaaaattgaaaaacgttcatcaattttgcagaaaaaaatcatgaagaaagaaagtttctcgaattagaaaaataaagaataaaagaacagaaaaaagaaaaaaaagaaaggaggaagaagaaaagaaggaaaaacatacacataatcacaacatgagaagtggtctggtggttgtcccgacttacacttgccagctcgtcgcgggttcgaatcccagatctcgcgctgtttttgcttcttagaaacagaaaaatgggccggcccagacgtgacgaggggtgtgcgcccgttagtagattgaacagtatcgggcgcaaaaggcgccgaataggatttggcccggacattgaaagtccccgaacttgaaaaaaactaggttttttttagggatgggaaaaaactaagtgagctctggaggagtccggacaatcaacttggcccactgacaattggaccacatgtcctcacggcccatgtgcctttttctttaggtctgtctggcccacttctgttgttcggatttgataactatgattggatggtggttggtgtctggggagtctgcttcacacatgttcgtgcacattttgtgtcgttcatttgatgaacagtgttggagttaaactaatttgtgccagtttcggtccctcgtgcccactaatggatgttctggatcgaggcccactaacactcccttcgctcgcccatctctctctcgctagttgcctctgctcgtttcaattttattttggaaccagctcacaggccaccttgaccgttcacttctctaaaagaaaattgtaggtttgaggaaaagtttgtgtatttgaaaaaggttcacggatttgaaaaaagtttcgtgtggttgaaaaaggattcacgaatttgaaaaaaactttatgcattttgaattgtgagttttataaatttcatgaatttgaaaaataatcataaatttcaaaaagtatgtgtatttgaaaaccttaatgcatttgaaaaaaagttacggattaaaaaaagttcacggatttgaaaatgtttgtgcatttggaaaactattgtaagtttcaaaaaaaatgagaatttaaaaacatgcatggcttttgaaaacaagttcacaaatgcccacaaatttaaaataacaaaacaaaaaccaacatagaaaaccaaaaaactaaccaaacaagaagcatctagaagcttcccaaaattatagaaagagctaaaaagcgcacccctcgctccccacgcgagcccttttgggccgatccgtgtgcggggcggggcgccctttttgtttttctattttctcgttttcttttttcctttttctttatttttctattttgcttatttaccgcaaattctggatgttgaaaatgtcataattttaagaaacccacaaatttaaatatattcatgacttaaaaaatgttcccatatttcaaaaaatgttcacaaatttagaacattcttcacaaaaaggttcactaatttgtgaaaacgttcataaatttcacgaaatgctccttggttctaaaaaatgttcgcaaatttctaaaaattaatattttgtatttgatgaacaaattttgaattgcatgaaaaattattgtatccgaagattttctttttgaatttcatgaaaaaaaatcgagaaaaaattgatgaacatcttttatattttatgaaaaaaatttgaaattgatgaaaaaaattgaatttgatgaacatctttaaattcagtgaaatttttttggatttgatgaataaaaatgttcacaattggaaaaaaatcatgacaatgaaaaaccgaaaaagtaaaatagatgaggtaaagcttatgtctccattttggaaaaaagaagaagatatctctcccacctaatgaatttgcactaggcatacgtaaccatcatagctcgtcagtgtcgaggacttctcggtgcagctgacaggactaactaaacattctttgtcactccctccgtttcaaaataagtttgatcaagtttatattaaaatatattaacatgtaggagtacaacataaaaaaaaacttgacattgtacatcttagcggatattttaggcttggttaaactttaaaggtttgaattaagacaattctggaatttcaattattttagagcggagggagtagcacagttgtactgcacatacatacatgccttttcttttccctcagattatacaaactgattttctccccatcttataacccaaacaaaacagcacgcgtcacccacgcttttgatagatacaagccgacattgacaatttttatatcgagcagttcaactcatggtggttgtaaacatcttgggtagggaggccaagcctagtgtaacaagcaattatttcttttcatttctttttgtttaacaatggtatatgtaattctttctctcttttttccccttttttttgtttgacagtggttctagcaattcttgccctcgtggttgatgtcttaacacattacttcctcctttccaaaatatttgaagtcctgggtttgtcgtaagttaaactttcctaagtttaaccaagtttatagaaaaaattactactaatatccacaacatcaaatactctctccatccggaaatacttgtcggagaaatgaattacaagtattttcggacggagggagtacatatagtaggaacctagtagaacttgaaatatttgaaacggatggagtgctacttatatggcctgtattccatagtgaaaagagggaatgcatagacgtgaggatgtggttaaatttcgtgttttggcccttttctgaaacctattcgagatttgaccctagtttgaaaaaaaatcgagatctgactcttttgctaccgccagggaccatggcggtagggtataacagcctaccgtcaagatccctggcggtagggttgcatgctctaccgccaaaaacctcttaagtattgaacacagtgcgtgctcgtgcctaccgccaagcatcttgacggtaaggttgtgcaggctaccgccagcccggttggcgttagcgatgtttcctaccggcaaagtccttggcggtaggctgttagaccctaccgccatggactctgccggtagcaaaagggtcagatctcaaaaaaaaaattaaactagaggcaaatcttgaataggtttcagaaaagggtcaaaacacgaaaatttgcgtgaggatgctgccgcatgcaagtcaactctcgccttgtcccgtgtgttgtcacctgtcagcactgcaatattctgtgccgcacttgtgacgtagtaggtccctgttggctgttgctgtgcacgcgcataaatatatggtgtccttcttctagctcctccttccccgtcgcatcagccataagcttggagccgtccatctttctcccgagccacctgcagatctttaccagtgtggatccaacaaagcataagcatcctttgtttgttggttcccaggtgagctttgtgtcttggtgtttccctgttgtttcttggctgtttcgctctcgccggatttccctttttccaggtaaaattttataatagattggctttgtgttggtgccaatgtctttgcccttttgtcttaggtgtgttttcttcatcccctttctgcggaaagacggttggtaattgtaagaaaaaggcttggtttgtgagctggtctgtatgggttgtgtatcatcgaagcagttcagacgagctccaccgcatgaggatgcggctctcctggccaaagagaccacatgtgagaactcctagcttctgtttttcttcctggtgatctattcagtacaaaaatgagtccataagcttttttgccatcgtttaatgtctaatttgttccccaaatgcacgcagtttccttgaatgaagtggaggccctctacgagctgttcaaaaagattagctattctatattcaaggatggccttattcacaaggtatctatcttgtcttgctcatcatcttcttttagtcacacagcaaattcaccatgtggtttccttctgcaaactctaatcagcgccaatttgcaaactgccattttcttcaacaggaggagttccaacttgctctcttcaggaacagcaaccggaagaaccttttcgccgatcgggtcagtctatttatctgtgggactgtaaaattgtcccaaacttatgcacaaatatctcggatcagatcaacttgtttacatgtgtgttcgactggtgcagatatttgatctgtttgatctgaagcgaaacggcgtgattgaattcgaggagtttgttcggtccctccacatttttcacccggatacacccatggcagacaagattgcatgtatgaagcctgcctttttctcccaatctttttgcgtgtgtaaatgttttaagaaatgactggtatttcataaattctgggtgcaattcaagtagcagtagcctggtatatatatttgggaggacttctgtccatatatgcttctcctatctgacatctgaaggattccttctcgcagttgcattcagactatatgacctgcgaggcaccggcagcattgaacgtgaagaggtctgtatctttttgtactgaaatgaattattctgcacataatcttgtcaactgaatatactactacaagatgtttttaacatactaccacacactagatttgccgatttaaatgcttatccttgagctctgtcagtacattttgtttcactggaaaaataacacaaaacccattatatgcaccaactatagcacacatactacctttacaatttgacagttcgagcacttactgaaactggccgttcatgcagttgaaggaaatggtgcttgcaatcctgaacgaatcggacctacttctttctgatgatgccgtcgaacagattgtagatcaggtacatacaccacatgcagatgaaatgttgctgcccaagaagcattaccactctgcatacatttatgctaacttatattgtgacagacattcaagcaggcagacctgaacagcgacgggaggatagatcccgacgaatggaaagagtttgcaagtaagaatccagccttgctgaagaacatgactctcccatacctgaagtaagtagctctatatttttcagctttggaaaacaatgcctgagaacatcactcttgactgacagctgagcatcttgtgatgcagggacataaccatgtcattccccagctttgttgtctactctggagccggcgacgaagagttgtagcagccatcggcacaagcatctgtgactctgacctcccaagtctcaggcatacagcaccatgctcacagtacaaatttttggattatgaaacaccgctttgtagcagaaactgcaatagcagaatgtgcagtgagaaatggaacacatcagctcaacagctgggcaattttttttgtcaccgaatgggctgatcactctcatatttacatttgcaatatctaacagctcgaccatatagattcttcacggtacatttagaggcaaatatacgtgcatttaacctgaactgtcaaaatctgactactgaacttcgtgactcgatatacatttcactcaaaatttgactactgaactccgtgattctcgatatacatttcactgtcccactcgcagaagttctttgtcaaaaatgaaaacacagaagttgagcaagtaactgcaatgacgatccaggttggttctactgcttcagcttcaccatctcctgcaagttcactggatagcacatcctggtgtgctcaccagaccacacattttctgccaggatctggttcacagcatcggtcgggtggaattcatcccaccacacatggcttgacgcgtcgctgcacgccatctgtggaagaacgcagatgaatagcccgccatacttgccaagcccacagcaagcatcagtggtggtcagaaagcctgcagttgcaattccaccaaaatgagaagacttagcactccatttaaatgtactacaaatgttacagttgcgaaccggcactcaatttaaatgcagtactaatgatgcatttactcaccatagcggtcacggttctctagtatgtccactgacccctcaaatgtatcacagtagctgatcatcgagtctgggtactggcggatgaactcacttgacatgtgtctcagtccatagttgaactcgatcacgacgttgttgatgtagtcgatgcattccccattttggccgccgtagtccgaaaggaagtgcggggcacagccaacaggaggaaggcccatcagcacgacctttcgaacattgatattgtacagattctgcataaatcatcattgtcagaaggtagtaggagacacatgagtccacaactagtttagacatcaaaagtggagtaatctgacaagaaccacaaatagaactaaacagtaatggatgcaaaatggcaattaatttgctacggtattgcgaagaataaagaaccgaagcaatccattacaatttgcatcagcatataaatgagattatggaatgaacacaaagaaaatttctacatacaacaaactactggtatatgcatcttcaatcaccatgcatcacatgttctgcttaagattaggtaacagagataaattaggcatagagaatcaaatgatccatgacacatgaactgagaagcatgcttgaaccatggtcagatcatgaacagttcaatgcagcgagataaatgtgcaaggtgttatacagttatagctgaaagtacaacatactttttctttttcttttgaggcaacgagcctcctcatcgatttccattaaagaaactggcacagttgtacagagtatgagaaacataaaaggcacaaaacaggaagctaaacctgcaagcatacaggtaaagcaactaccaggaaccaacagcacgcaggcagatcaaacacgactatcaagcatgagcgccagaaattacagaccagtgaaaatacatcaagttccacaggtaaaacactgcatcctagaacgagggttccatccatgaactcagaaagtacaacagacttaacttgaacctaaaacagatggtagcacaaaaaaaaaagtgcagcctgagcatttcagaatataccacagtttaagatagtatgctgtggtgtagctcaggtagcatgagtagcaccttgtgcagtttcagtttttcagttgggctgcctttgtattgcttcgttttcaataatttaggacttttgcttttcagggttgtatttgctttgtactttgcgtcatcatgatgccttcttctaatatacaacaacacacatttaagtgtgcatttgagagaaaaaaaagttttaagacagtataatgtcagtttggtttgagcccatgtttgccttaccaaaaagttaggccaaaattttggtcaaagttgtgcttgtccatgagttggccaatgttggaaagaaaaatgaactagagttggtaaagagctagtggcatgcaaaaaaaaaaatggagataagtttttggctaccatccaaacatacccttacatactaactaaacaccattacacctcattgtagaatcaggatcatcaatccaaccattttcatttcatattttatcaaaatgccgcatctaacatcagtctgaaacattttgcaattgcaatacttgtgcaatattgcaagaaataaccccaaattcgacagtaagaacaattagtatgtaaaccataggtgaaggcgttaatgttctagaactgcaaaccaacaaggaaacctcattcatttaaatcactttcatgtgttaatttcccagataaactacgttatgtccaaaacgaaattcgtagttacagagatccctgactacgcgtggtgcatctccacaagtaaaacaatgcgcagcacactggtgatcaaaaaggaggcgagcaatggtacacagaacaaactgaggagcaagaagcacaccttgatttcctgcctcactgcattaacaaggagctgattgaactcccatgggagataatgcatctgcacgcctgacacattgcgcaggtagtagtggatgaagtcgttgctcccgatcgacacaaagaacaccgacctcctgaacaggtcgactgtagctgcctccccaagagccagcgccaactgctcatatgtgtcctcaacctgctgcacctgctgggtcagcgacacatgcatccactgcaagcgcaaccaaatcgcaaaaaccccagccgattagcatcaaatttggtgtgaggaaaccacagattgacagcagaatcatccccaaacagatctctcgacagcaccaggacgttatgagattgacgcagagttggcgggnnnnnnnnnnnnnnnnnnnnnnnnnnnnnnnnnnnnnnnnnnnnnnnnnnnnnnnnnnnnnnnnnnnnnnnnnnnnnnnnnnnnnnnnnnnnnnnnnnnnnnnnnnnnnnnnnnnnnnnnnnnnnnnnnnnnnnnnnnnnnnnnnnnnnnnncccagcttctccgctgtggtacaacagaaggggtcagaaatcaagagaagagaaactaggaacgtcacatggggctgccatattcctgcggattcagaccacagcagcgacagaagattacacggaacaatcaaggagaagaagcaaacaatcatccgtaatcacgatgaaccatggagacctaataaaccgaaacgatccgtatctgcaagcaaggacgcgtaaaatcgagnnnnnnnnnnnnnnnnnnnnnnnnnnnnnnnnnnnnnnnnnnnnnnnnnnnnnnnnnnnnnnnnnnnnnngtcgacggggatgcggccgttggagaagcgtccggtggggcggtgggtgtcaaagtcccggccatagggctcgcggtcggctcgggcgagcgtgccgaggtagttgttggtgccgacgtcggacgtggagtcgctgatgacgaagagcgcggggacgagcggggtgggcggcgccgacggcggcgcggaccggggggtcgaggcggctgcggagaggggtagagcggagaggaggaggagaaggaggaggaggaggcacacgaggtgagcgagggagggcgccatttccggcgaggcggtggcgagttcgcgcgctctgggttgggaaaggtgggggaggagaccggtctggtttatgcaagaggatggactcgccgcagactcgagtcgctaaccgcagtgaggatggaggaaaaggcctgcatttggtgggctagggttgtggactagagcaactcagcaaacagggccggaaatgggccccttcacagccgattgatgcgcggccccttggcctgtttgatatccttgcttgatctccaagaatgacaaaacgtataagaatcctcaaaaaaaaaagacaaaatgtataagaataggaggtctaagatagggaaatacccattggtttctggttgtatctacactctatatgggatttttttaaataaaaaagtcaaaacaatctgaagttttatagaataaacttgactttcttttgcactaaaccagcactgactttaggggaaaaagttatttgctattataggtcactattcgcactatttttggcagaggttttgtctttttgaagaagtcaaagggcctttttctttttatgaaatttttcacaagtactccctccgtcccataatgtaagacgttttttgacactacactagggtcaaaaaatgccttacattatgggatagagggagtacaatggaaggtcaagtttatttcaaaaatgttttcattttttttttactttttatctaattacttatttttgcatatagggtgtatatacacccataaaccaaacgtctttgtgcgtctaggattcatagatactgagcttttctaattggctatgctcacgcgtcaaacgtgaagggttctttttttagcaactgacaggagctctacctttttcatcatcaagaagagaattggtcaatttctttttagcaaccggcaggaaattctcttcttttccccccatcaaaaagaagaagagaattggtcagtttctaagggaaactggccgaaaacctgatagattggtgctcgggtgctcaaagttgtctattgcccgagagttgatttcctaaagtttgaggtgggaccacacccttcacaaatttgtcgtgctatcgtgaatgaaaaatgtgttccacaacaaggatttattaagagggtaggtacaagagagcaaagcaacatatgtaatgagcaatggccatgggaatgtcttctgcgattgtactgtattcaaaaaatgagcaatggctacctagaagggggcatttcggtcgacacgatgcttgaccagcaatggtgagcaagttcattgtatcttcctcgaccgagtgggatgttcaaaagttgcagcggcaattttacctatggatggtgagaacatgaggaccatcccactaccaactagaaggcaaaatgatttttgggcttgcttccatgataaaaagaggtttcttgattgttcagtcaacatatgatatgcttatggtaggaccaaagttatcgatcagaggtgaggtgaataggagatttaacaaatccttaaaaaaatacaatccttcaaagaaagccattataacagatcaacacactagtataaaaacgacattagaagtgaaatgacctacatgtcgcctagaggggagtgaatatgcgttttaaaagtcttatggatttcactttatcataatgtggagttaaactaaatctttcaaagaaagccattataacagatcaacacactagtataaaaacgacattagaagtgaaatgacctacatgtcgcctagaggggagtgaatatgcgttttaaaaagtcttatggatttcactttatcataatgtggagttaaactaaatggatacttttcaagcatagatcctaaatatgctaggctcaattaaacgcaccaacaacctacactgaacaagataagcacttaaaggaaactagcaacaagggtgacaagtacttgccttgagcttgacaacatatatcgttaggcaaaaggaatatagcacatgccacattgtcaaggttcgtcaaatgattttgcacacatgggagttgacactttctgctggaaggcactatcgaccgatcgagtcggatgggatccaagtcatgtccatgtgtagtctcaaacaagttgcagtaggctagagctgaaacccataagatcttcaaatcaactcatggttctggcacgtggatagctaacttcatgcactcatgtccattgctagttctttggtgacaccacatgtcaagtttgatctaccccgacctctattgacattatcagtacgctttaatcatccgctatgcactgcagtttctagaggtccatgttgaatatgatcaaaacatctcagatggtattaaacaagtttcgcttcagtctgtgctaccctaactctatcatgtatatcatcgttccggacccgatcctttcttgtgtagtaacacatccatctcaacattgcattttcgctacacccaactattgaacatgtcgacttttagtcagctaacacccaatgccatacaacattgcgggtcgaatcgtcgtcctatagaaccttccttttggctttgtagactacaacctcttgatgacgatctctttatagcaacggtgatcagaatggataatttgaagtttcgtcccaggtgaaatcgccaaaaagtgtgttctcacatgtcacagtgtacccctgacaactaacatggtacacaagacacatcgttggaggaacctcgccaggagcacagtaagcaagacatgctggtgggatcttttgagaaccgacaccccccccccgaagggaaaccgtcagggcacacgacgcttataggctgccctaggttggcttgactgcccctatgtccttgatgtcgaacacaaagaaaatcaaagaacgaggaagaaaaaaacaaggatgaaggagaaaaggtaaagataaaagggattgagggaatcctggattagggggtctccgaatagcgggactatatccattggccggactgttagactatgaagatacaagattgaagatttcgtctcgtgttcggatgggactcgacttggcgtggaaggcaagctaggcaatacggatatggatatctcctcctttataaccgaccttgtgtaaccctaac

>TaCBL4_1BL_3917583

cagcacactcacccaacatcaatgtaggttgtccaaaaaattcaactcaaaattcaaaacatagcttgaaaaacaaaaataacaaattcaacactgaatagtacacaacataactttggctttagatttggcccactatcacacttatttcaaatttgtcatttttgtatctcaaaaaatatttcaaatttttatacgatttttttgacatcatacattgatgttgcgttaacgtgctagatttttttcagaacttttaaaacattctatggcatccggtgcaccagtagcaccacacgtgagggtgcaccggatacattcccctgttcgctcgaacagaatgttcgcatgacacgtttcgtacttggcggttcactgggggcagccccagtgtcgttccccagatacgggcgtcctatttgttagactcataataacatggagattttttttcatggaaacatggagatcaaattagaacactttcagtactcaaataaaaattagaatactttcatcataatttttgtaaaacttcgaatatttaaagtgtaaagtgtggcaccccaattctacaatcctagtcgtccgtttctgatcaccgtgtcggtctaccgctcccgctatacctgaccatgggcagcccggcctggacggctcgaccttgccgccgggcctagattttgagcatgatgaccgggctgggtttggacccttcatatctacaatttaaggaagaggcccgagcccaaggcccgcgagctttttgactgatgggcctggcttgggcctgaattttaggnnnnnnnnnnnnnnnnnnnnnngcacacgtctaggttttttgtgtcgggctttgataggcccggcccgatgccgccacgcggggaggtgaacccggtttgtttacgaaatggcgcgtgacttaagtggcggtgcaaccctcgtcgttcccgtgcttacatgcgggcagcctacgggagcactggtcgacggtactagtagcagtacacacgtacgtacccatgtgcctgcgtgcgatgtgatccagactcatgaaaagcacgcgcatgacgagctgaggacggtgatggatgcagaacagtcggacacggcgagatctcgggagacagggacggccgccgcttgttgcctggacccgcacagtcgcactcgcactgcagcgggaaagaaccatttcacttggcgccccgttaaatcaggccccaaggaggaggaggagtcgagtccagccagccaagggaaagacggaggagacccagcagcgggaagaggagaagacagaacaggagaagctacgcgatctcagctcaggtcagattgtgtttccggtttcgtcatcacgtctttcttattcttgctacgccgttgtgctgcctcacgcctgcctgccttcagtgcctctcgcggcgccggccggggtgggaagggatcgaccgaaccaatgggctgcgcttggtcgtngtcgaggcgtcagcggcgcgctcaggggtacgaggagcccgccgtcctcgccgccgagacctcctgtacgcattcccagctccagcttcacctcgttgtttttctatacgcgcaactttatttacagaaatcctattcctatcaaaactggtagtaacttcgcgtcgcgcgcgtgcagtcacggtgagcgaggtggaggcgctgtacgagctctacaacaagctcagctactccatcttcaaagacggcctcatccataaggtaaactccacgaccgctatggatccatcttcaaagactgcatgctcccatctcatcatatctcatctgcgttcatcacaggaggagttccggctcgccctgttccggaccagcgagagagccaacctctttgcggacagggtgttccacctcttcgatctcaagcgcaacggggtcatcgagttcggcgagtttgtgcgctcgctcagcatcttccaccccaaagcgcttgtatcagacaaggccgcatgtattgtccattcctctgctcctacacctcttgtccattcgtcttaatttgatgtccatcaattatcatgccagagtagataaccattttcggccatcctctcctgcagttgcattcaaattgtacgatttgaggggcacaggctacatcgaaaaagaagaggtaaattcgtaatgttcatgagttgttctgaattctagtataacttgactgttgacacagcttagggagatggtggtggcacttcttgacgagtcggacctatgtctctcggatagcgctgttgatgagatcgttgacaatgtaattaacgagatagggctacttctttccaaacattcagtcttttaggagtaaatagtatttggtttccttcttcttttttgaacaaaatatataatctccttccttctaacatgacctgtgctgctctgctccctttgggctgtcagacgttcagtcaagcagacacgaatggcgatggcaggttagaccccaaggagtgggaggagtttgtcaagaagaacccgacatcactcaggaacatgtcactgccctatctccagtgagtaggcactccgcactcatctaatgagtttgccctttaccacatatcaagtgatttgatggttgttgttgctgtcgttgttgttgtttgtttagggacattacgaccacgtttccgagctttgcaacgcattcggaagtcaatgattgtggtggcgtcgacaaataaccaggcagagccttcctgcctgcaggtgcaggggttatcagatgattgtcatataatatattacctccgtctcaaattagttgttttatatttgtgtagacactaaaacttgtctagatgcatctgtatctagataaatttacgacaactaaaacatgtgtagatacatctatgtgctacatgtacaccacacacggtgtggacttaagacttaatttcaaaaaaagagtgacaaaatctcaaggcaatagaggcactttcatcgtcaatggtacatttgatgatatcttatgctccaaattcaagtataaagaggaaggctaatattcagtattgacctgggtcacctgtgttatatatacctcttgtaagccgccgcacggaataagttgatcagttgtaaatttccagcgtttgtaaccttccccgatatagtgaagtttggctggccggcgcgtgtgggttttttcccttcgtgttggacgggtttcccttgcttgttctttgttttccgttgagttgatttgcttgtcgtatctctaacatttgatgactgtaaaaactgcctgagtttttttatggcacggggactgatttagtttgctaggtagagaaagcacactatccatgtgacagcaataatcctaaacacaccggcatattatgcgagttttacaggcttccttctatctaatgtctctgcccccgaattatggtccacattatagaaagctatagtatgcaactcaatcgaaatttgcttttggagatcgagctccacgaaagccttcaaatgcaaaattcaaactttataaaaaattgtatttttataattcaaaaaattctgacgaaaaaatacagagatagatgtaggcataatgcacaagtctgcaaattttcaggacggagtacattgacatgagggctgcgcaaaaaaaaaatctaagacttcttaacacgtgatactattcatcatcgcagaccatgaaatttattttttgcaggtcgcatttcattttaaaagtttaagcacatacgcctcacatccttgtttacttgtgcaaaaaaatcagaattttctgaaaccaaaatttttgaattttgattttttcaaaaattctgcctttgtggaggctaagatccaaaacgccattcttgaactcaatttcattcatcggagtttggtacaatatatacaggagatgtcttgcgtgacaagccaaataaccctaccatatttctagtagttagactatacatggctagatatcataataagatcaatcgggaataaatacggatatgtcacgttccggaacgaaactcatggtatgcggcgattggcaacccttaggtcataatttcggcaaactgttaggtagtcggtacatgaagaacatggagctgacccaactggaccttctcatgcacaaagtgaacatccaactcaatgtatttgctgtgcttatgctgcaccggtcggctgcgagataggtagccgaaatattgtcacagaaaaccaccatcgccttcagaagaggaaccataagttcaccaagaaggtgacgcagccaacaagtgtcggcaataacgttgatcacagcgcagtactcaacctctgcactcgagcggtagacggtgagctgccgcttggaggaccaggagacgcgtgagtcacctaagagacacggtaaccggctatcgtgtgtcaggacagccatcccaatccgtgttcgtgtcagcaagaagctcgttgaaggcggagaagtgaagacgtagaccataaatcggtgtgccgcgaatgtagcaaagaatgtgcttgaccagagaccaatgagcatcacgtggcgaatgcatgtgcaagaagacttgctgcaccatgaactaaatgtcttgtttgtgagtgtgagatactaaagggcaccaaacaatgttgcgacagagagcgacatctgtggcaggctgtccagctatggcagagagcttcgacttggcctccgctgatgtggcggccggtttgcagtcagtcatgcccgcacggtccaacaggtcaagagcgtactgcccctgatgggggaagacaccggaggcatcacaatgcacattaaagtcgaggaagaagtgcaaagtgcccatgtcctttagctgaaactccgagccaagcctgaccgataatgtcgcgaaggaagtcctctcgagatgccgtgataacgatgtcgtcaacatacagaagtagcatgacgacatgatcagcccaatgaagaacgaagagcggcatctcagaagtggtgttgcaaatccgagagcggcgaggaacgtcgcgatgcgctggtaccacacgcgaggagcctgtttggggctgtagagggacctcgataacaagcagacatcattcgatcgctacgggtcgacaaaaccgattgactgctaatagagattacgctcttggagatggccgtacaagaaagtattgttgatatcgagctgatgtatgagccaaccgtcgagaggttgtaagctgtaagacaactcggatcgtggcgggtttgaagatggtagagaaggtctctgagaagtcaacactagcacgctgtgagaatccacgaacaacccagcaagccttgtacctgtcctgcgagccatccgggtgaagcttgttcctaaagacccatttttcggagatgacgttggcaccaggggccgagggacaagagtccaactccggttggtgacaagggcatcgtactccgcgcacacgacagtagtctagtgcaggtcgctcagtgcagaccacaccgagcgtgggactggaaagagcgtcatgaaactgaggttgtggtagtgcgggttcggctgatggatctcgtccttggcatgcgtcgtcatgggatgatgaggcggcaaaagtggcgggcgnnnagccacagctggtggcgcgggaagcggtgacggaggcgcagaaagcccagccgaggtggccgagggcggtgacgagggaaccgaaggcacggccgaggcaacagaaggtggtgacgggggcgccgccagtttggcagaggaagccgagctggtgatggggcgccgaggaatgtgccgaagagttgatcaaggcagctgagagtccggctgtggcaaccaagaacggtaacgtggccgcctaggaaccggctgagggcgatggtgtccgcaccaaggcgggcaagaaagccacgtcacactacaagaaatatgtcaactagtgaccttctgtcaatgaccctggaagaattggtcatagatctatgactatttcagaccaattggtcaaaagctgctcggggggctccaaaccctaaaccattgcgaccattttggtgagaaaggtcgtaatttccttacacgaaatggtcacaaagcaaacagtgctagtccgctgccttatttctagttgttaatgaccaatatagatggtcataccttgtagattgtggtgggttgtgatgactaggcgccatctcatcagttttgcctatgtgtcatgtccatgtggcagtttttgccctaggttgtgaagcaacctatatttctgttattccaaaaattcccaaaaaattctcataaatgttttggatcatatattcatcaaatatgtcaaaaacattccttgcctagttcaaaaataattcgaaaatattcattttcctattcttttcagagcagcactttgtgaaggaagtaccactttggcatgtccaaatggtatccattttctacactgctttcctatgcccaaataaccatcctccaccaaatgccagctcaatccattcattattttgagcccagcttcaacattcgtatttatgtccagtgtgctactttgtaatgcaagtaccgcctaggctcctccttttgaggtgaaaatttgtgaagacagtcttcttagcaactgatcatcctcagccaaaactcacgcccattagccatgtgcatttcccataccgcaaatcaaacacttggctgcttattcatgtttgagcatcgatcggtctccccgtgagaatcttatgttgtgattttgttcctaacacctacctgcggagtgcccaacccactagacatgcctaggcctcccagaacacatggcaacgccacggtcacgtggtgaccacgcggcgggcatgcgagcttacgcgctctagagttgggcccctcggccaccgtccaaacctcgatgtcttgccatcaaatcatgtatttctgattaaatagatacttatttacctagaaatgatttttggaaaaaataaagagcaaactatgaggcagctgcagttcaaatttgacccgcttccaactgaatcgacggcaatttgtctttttcaccagaggtggatcaaaacttttttcacccaaccattttgtcaattatgcattatatatatatcctagtattttatgaaattgatttggtccatttttgcaacaattatttggtaggtccttcacaaaaaaacctccttttgggcactcggaaaatgaaaaatgaattttctatgcaaagaaaatgaaaacttacttaggaacattgtttggaattccaagatgcacccttgtgcacaatatgagatcatttgaacaaactatgccatgaat

>TaCBL7_1BL_3897439

tcctacaaacctgtgcacttgcaggcccaacaatgtctacaagaagaaggttgtgtagtagacatcaatatggtcaagacatgatgtgatgtacgttattgtatgagatgatcatgtcttgtaatttatcggcaattggcaggagccttatggttgtctttattgtatgaagtgcaaacaccatgtaattgctttattttatcgctatgcattagcaatagttgtagaagcaatagttggcgagacgaccacgacgcaatgatggagatcaaagtgtcgagccggtgacgatggagatcatgacgatgctttggagatggagatcaaaagcacgagatgatgatggccatatcatgtcacatgtttgattgcatgtgatgtttatcttttatgcatcttattttgcttagaaccatggtagcattataagatgatcccttcactaaatttcaagatataagtgttctccccaagtatatgcaccgttgcgaaagttcggcgtttcgagacaccacgtgatgatcgggtgtgatagactctacgttcacatacaacgggtgtaagacggttttgcacatgcagaatacttgggttaaacttgactagcctagcatgtatagagatggtctcggaacactagagaccgaaaggtcgaacgtgagtcatatagtagatatgatcaacatagagatgttcaccattgataactaccccatctcacgtgatgatcggacatgggttagttgatttggatcatgtatcacttaaataacttgagggatgttaatttaagtgggagttcattagtaatttggttaattgaacttaaattatcatgaacttagtctcaatagtttttgcatatctattttgtagatcaatggctcgcgatatagctcccctatttttgatatgttcctagagaaaataagttgaaatatgatagttgcaatgatgcggactgggtccgtgatcagaggattttcctcattgatgcacataagaatatgtccttgatgcaccgctaggtgacggacctattgcaggagcagatgcagacgttatgaatgtttgacaagctcggtatgatgactacttgatagtttagtgttccatgctttacagcttagaatcgggacttcaaaaacgttttgaacgccatagagcatatgagatgttccaagagttgaaattggtatttcagactcatgcccatgtcgagaggtatgagacctctgacaagtactttgcctacaagatggaggagaatagctcagccagtgagcatgtgctcagaatgtctgggtactgcaatcgcttgaatcaagtgggagttaatcttccagataagatagtgattaacatagttctctagtcactatcaccatgctactagaacttcgtgataaactataatatgcaagagatgacaaaaatgattcccgagctcttcgcgatgctgaaatcagcaaaggtagaaatcaagaaagagcatcaagtgttgatggttaataagaccactagtttcaagaaaaagggcaagggaaagcaagagaagatcaagaagaatgaaaagcaagttgctgctcccatgaagaagcccaaagctgaacccgagcctgaaactaagtgcttctactgcaaagaaaatggtcactagaagcggaactgtcccaaatgcttggtggataataaggatggcaaagtaaacaaaagtatatttgatatacatgttattgatgtgtaccttactagtgttcctagtagcccctgggtatttgataccggttcagttgctaagattagtaattcgaaacaggagttacagaataaatagagactagttaagggcgaggtgacgatgagtgttggaagtgattccaaggttgctacgatcacgatagcacactccctctaccttcgggattagtgttgaagctaaataaaagttatttggtgtctccgttgagcatgaatatgattagatcatgtttattgcaatatggttattcatttaagtcagagaataattgttgttctgtttatgtgaataaaaccttctatggtcatacacccaatgtaatggtttattgaatctcgatcgtagtgatacacatattcataatattgatgccaaaagatgaaaagttggtaataatagtgcactgtcgtttaggtcatattggtgtaaagcgcatgaagaaactccatgcggatggacttttggaatcacttgattgtgaatcatttgatacttgcaaaccatgcctcatgggcaagatgactaaaactctattctctgaaacaatggagcgagccaatgacttattggaaataatatgtaccaacatatgcggtccgatgagtgttgaaacacgcggcgggtatcgttattttctgaccttcacagatgatttgagtaggtatgggtaatctacttgatgaaacacaagtctgaaacatttgaaaagttcaaagaacttcagagtgaagtggagaatcatcataacaagaaaataaagtttctacgatctgatcgtggaggtgaatatttgagttacgagtttggtcttgatttaaaaacagtgtgaaataattttcacaagtcacggcacgaggaacaccacagcataatggtgtgtccgaacattgtgaccgtactttattagatatggtgcgatctatgatgtctcttaccgatttaccactatcgttttggggttatgcattagagatagttgcattcacattaaatagggcaccatctaaatcagttgagacaacattgtctgaactatggtttggcaagaaacctaagtcaaaaggagatgcgatgcttatggtagggcaccatctaaacctaagtttggggatgcgacgcttatggtagggcaccatctaaacctaagtttggggatgcgacgcttatgtcaaaaggcttcagtctgataagctcgaacctaaatcggagaagtgtgtcttcataggataccctaaggaaacaattgggtacaccttctaccacagatctgaaggcaagatctttgtcgctaagaatgggtcgtttctagataaggagttttctctcgaaacaagtgagtgggaggaaagtagaacttgatgaggtaattgtaccttctctcgaactggagagtagcacatcagagaaatccattcccgcgatgcctacaccaactagagaggaagctaatgatgatgatcatgaaacttcggatcaatttactactagactccgtaggtcgaacagagcacattccgcaccagagtggtacggtaatcctgtcctggaagtcatgttattagaccatggcaaacctacaaactatgaagaagctatgatgagcccagatttctataaatggcttgaggccatgaaatctgagataggatccatgtatgagaacaaagtatggactttggtggacttgcccgatgattggcaagccatagaaaataaatggatctccaagaagaagactgacgttgatggtaatgttactgtctaaaaagcttgacttgttgtaaaaaggatttcgacaaatccaaggggttgactacggtgagactttctcacccgtatcaatgcttaagtccgtctgaatcatgttagcaattgtcgcattttataattatgaaatctagcaaatggatgtcaaaaactgcattccttaatggatattaaagaagagttgtatatgatgcaaccagaagtttttgttgatcctaaaggtgctaacaaagtgtgcaagctccaatgatccatctatgggctggtgcaagcatctcggggttggaatatacgctttgatgaagtgatcaaatcatatggttttatacagacttatggtgaagcttgtatttaaaacaaagtgagcgggagctctgtagcattttctgatattatatgtggatggcatattattgattggacatgatatagaatttttgaatagcataaaaaggatacttgaataagaatttttcagtgaaagacctcggtgaagctacttatatattgggcatcaagatctatagggatagatcaagatgcttaataggactttcacaaagcacataccttgagaagattttaaaggagttcaaaatggttcagtcaaagaaagggttcttgcctgtgttgcaaggtgtgaagttgagtaagactcaaaacccgaccatgacagaagatagagagagaatgaaagtcattccctatgcctcggccataggttctataaagtttgtcatgctgtgtacgaaacctattgtgcaccttgcaatgagtttggcaagggggtacaataatgatctaggagtagatcactgggtagcggtcaaaattatccttagttacctaagaggactaaggacactacaccatgacactcaaagcagcgtcaaacaatctgtcggcataggtcacctaaggcgacagatttaatgtcggtaaagatctttcgcgacagaaaggagctgtcgcctatagtcctgccgggaaaggtcttcgccgacagataaatgtatgacgacagacattatgacaacgttggtgagatctacaccgacagacatttctacaccgacatacaccacaacaccgacagattatctgacaatgtgctaccccgacagatatagcaatgcccattgttatgccgacagttcctgtgtcaatgtgatataccgacagaaagtgcaacgtccaatacttttgccgacacatttgctgccagggttgtacatttcagcataaatgcaaatacaccatattttaacatgaaatcacattcataatatttaacagggttgtacatttcagcacagatgcaaatacaccatattttaacattaaatcacattcataatatttaatacatgttcagtatacaatgcatatatacaaacaattgactcttctattcatcacaaaatgtcatccatgcgtacattcattcaacatacataaagattcatttattttcttcgctcaacatacaagataagttgttacaaaatgtgatggctaagatcctaacagaccaattgtttgatcacttgactactactaaatattatggaagagaccaagtctttgatcaccaagccaacatcaagttctgtatgaacaaaaggtgtcaaccatgttatgaacaaaagcgatcatggaacatcttagatcaacttgtggacttgttatttcctgccatttgtccctgcaaaagaacttattgccttgttggattaaagcaccaaatgctcataaaaggaaaaacctttacacacactagagagtaataaatcaatcagggcatgtcacctttgaagaaaccaatggagagctctccaggtatagtaacagcatgaaaggtaactaatgtctaaagcaataaggtacatgcttggtcaagcttgatgtaaagtatccagtagtttgtgcataaggcaaaacaaatatggatgtaagctggcacatatatcggcataaaacattaacatgacaaattagtttgtgatctaaataagtagcgtaggatccgaaatatataccatgatatgttagcaatgcaaataatacagttctcagaattacaatcagccaacaatttaagtatggtaataatacaatggcggccctactttggacggttaaatatgtaaatagcaagcaaacataaaaggaatagggaaagtatcaatgcattgtttcttttgcaatatcatgtcccaatagagatactaatttttggacagcaagtttgaggtggtcgatgctctcaaactacccagtaaaaaaatactctctggaaagcttcagaagtacactacaacttctatctggagactaactcaaaattcagcgtgtatgaaaccgatatttaacccaaatcttactacaccataactattacgaggtgatttaactgccatatgaaattgcaatcaacactggagatgaacatctcctagcaagcagcaaaattgacataccaacggatacaaacagatgttgcatcctaatctacccactatggccataatgttgtagtggctaaacatactagtacttgtgtacatacttctgttcttagtgtaactagagaatatagtaggatttacacaaagaggatgtggtctggttgaaacaacttttaaactgccagaatatcttatttagttaactagaaaattttcaagttgttaaccttagcatgttttcctatcagcaagttcagtagctcaactgaacaattatgttccaactgagaatatttgcatatatagtagctccctttgtaccacttaacatataaggtgtattagattagaaacaaattaccttatctttatggctagatctttatgtagccacattggaaaataaagttcatctttttcctctggtgactacaccgggtagttttgctagcattagttccatacttctatgatagttgttccagaagtgcacaatgatactttactagcctgaaaagaaacaacacttaattcactcacatagtactgaacgcagaatctaattggcagcaagcattcaacatcaatatcaaagtaccatgtagactattaacataagctgaatttagctgctataggttccgcatactaacaatcaattaagtcgtacaaacttcacatgtggctcttttggttagtacactttgctgcaaaaggtgtggttgctttgagaagtaacagaggggccttaacactaccaaccatgaaataaacatttcgtctagaaataatgaatgaagacttcttgtttttttctattactgatctaattgttgatagaaatactgtatgctaatataattacgcaaccatatgattcaggtgcttataatgcataatgcaattaattaaatgcatgttacttttcagtacgtaattgaaccaaaattcctagtatatccctagagagaaccactcattttagtctagtggcctgcacttccttgggattggatccaattcatgatatgtggcacattttgtgtttttggtaggaaagcagaagagagggcattgttcttctaaatggaggaaagtagtgacgcctcacccttgcaagtcttggcatggccaaccaatctgtgtagtgttccatccatcttaggtgaattctttttgagcaaaatacagagtataattttctatttgaacaatatacggagtacactacaggctacagtctgaacatggatgagtactgaactagcaaatgtgatgttcgcactgatggtttatgcaaaaccatacaagtgaaggtggtgggatcacatgcaaaacagagatagaaacaatacatacctcaagcattaccactcagcatggttggggatccttgcagcctgcgaatggaaataggaacattgagacacaatcatttcaaaggtagtagtaattagtaaatactaaaaccctgaaactgaatattggatatatatgtgataagagaagggagaggcctgggaggttgagctcagctacaagcatacagtccacgagcttcagaacatccgcaacaatagtcaaacctcattaaaagttgaaatcagttgcaaagttctacttatgagaagaaagctagcctgaactaaaaacagtagcatgtttctagacaggcacgacacccatggaagagcaagtgaaatataatgcatcttgttgaatccacaaaacacccaggccaaggtcaggcgcacaaatctactaatcgtcatgcagtcttatagtaaccattctctcgaacaacacgtgttgtagaacacatcccaaatcaacagcaacaaaaacaacattaaaataacttgacggtgctaatcgagaacaaagagttgcaatatagctatgtgaataaacaatgtagatgggactagaagaattacataaacatataataacatagcacataacactggttagtaaaatatatatacttcagtctgtccagaaatcaactaaatacttaatttaccctaattataacatgagagatggatggttagcactatgaaatctccaatctcaaacgaactaacatgatcttgtttagttcatcgttgagccaacaagatttaatgggcaatcacattcacggggaaacaagaaataacatatgaaatggaaaagaaaaaagggatctattttcatgcttacctacaggggctggatttgggggcgaagcaagacaaagggaaggagaacttcttgatgtagagggctaggtggctccttggttgcagacagcatcaccctgcgttggatcgaagcttcaggtggcactactggtagtcagagaagcagaggagtgaccatatcggcctgaatcttacctacagcgccctggatcttgaggaagtacggcggggttgttcggcgatggtcgactccacgctggcggttggcaacgcggatccttggtggcggtcgactcgactccttggctgcgggcagtggaggggctcctttgctgcggacgatgaggcataaccttgggattggaccgtggggaagcaccacatggtagaaggaggcttcgggaggtgccgccgttgatgggacaggaggcggcacgagcaaatcgacaggcaggtcaggggacggcggcggcggcaggcgacctaggtttaggttgatgtgtggcgtgggaaagagataagatggggtgggggaaagaggggtctgggaggcttagccaaaattttagtctttgagtctcccacgcgcaacttctcccgcatggcccgaaataagtgggccaggcccaaacatacgccgacactctttctctcgtagagcaacagcgacacataatctaacagcaataaaacttatactgacacataatatgtccgtaaaccggtcatccatttccgacagacagatcatcgaaagactagaacaacatcgaccattagtgtgtcggtattgttacgaacagataaagtgtcggtaatgtacacttataccaacatatgcatgtcgtcattgtggtgtcgtggtgtagtgggagatatttatcgattatggaggtaataaagagttcgtcgtaaaggtttacatggatgcaagctttgacgctgattcggatgactctgagtctcaatctggatatatattgaaagtgggagcaattagctagagtatctccataaagagctttgtagacatagaaatttgcaaaatacatatggacctgaatatgacagagccgttgacaaaatttctctcacaagaaaaacatgatcactccttagtactctttgggtgttaatcacatggtgatgtgaaataaattattgactctagtaaactctttgggtgttggtcacatggcgatgtgaaataaattattgactctagtaaactctttgggtgttggtcacatggcgatgtgaactatgggtgttaaatcacatggtgatgtgaactagattattgactctagtgcaagtgggagactggaggaaatatgccctagaggcaataataaagttgttattttatatttccttactcatgataaaggtttattattcatgctagaattgtattgatcggaaaattaaatacatgcgtgaatacataaacaaataccgtgtccctagtaagcgtctactagactagctcattgatcaaagatggttaaggtttcctaaccatagacatgtgttgtcatttgataacgggatcacatcattaggagaatgatgtgatggacaagacccacccgttagcttagcataatgatcattccgtttattgctattgctttcttcatgtcaaatacatattcctttaactatgagattatgcaactcccggataccggaggaataccttatgtgctatcaaacgtcacaacatgactgggtgatcataaagatgctctacaggtatctccgaaggtgttcgttgagttggcatagatcaagaataggattgtcactccgagttttggagaggtatctctgggccctctcggtaatacacatcataatcttgcaagcaaatgactaagaagttagttacaaggtgatgtattatggaactagcaaagatacttgccggtaacgagattgaactaggtatagagataccgatgatcgaatctcgggcaagtaacatacggatggacaaaggtaattacgtttgttgtcataatggtttgaccaataaagatattcgtagaatatgtaggagcaacatgggcatctaggttccgctattggttattgaccgaagagttgtctcggtcatgtctagatagttctcgaacccgtatggtccacacgcttaacgttcgttgacgatatagtgttatatgagttatatgatttggtgaccgaatgttgttcggagtcctggatgagatcacggacatgacgaggattctcaaaatggttgagacgtaaagattgatatataggacggtggtattcaaacaccggaagagtttcggagtgcaccgggtagtcatcgagtcaccagaaggggttccggacaccctcgataagtgtatgggcctaatgggccaaagtggggacagaccagccactggtgttacccttctttggacagcaggccctaagggaaggaaaggagggagggctagcccctcctgcctttccctctcatgggagaaaggaaaggggggcgccaccctcccgtgcctttcccccgcacctatataaggcagggggaggacggcttgggaaggactccaagtaggattcctcctacttgggcacctccttggctgctcctccctcccttccacctatatatatgtgggaggggacgcctagcacataacagacaattgcctagccatgtgcggcgcccccctccaccgtctacaaccacggtcctattttcgtagtgcttaggcgaagccctgcaaagattacttcaccatcaccatcaccatgccgtcgtgctgccagaactcatctaatacctcgccagcttgctggatcaagaaggtggaggatgtcaccgagctgaatgtgtgaagaacgcggaggtgtcgtgcgttcggtactagatcggttggagcgcgaagaaagttcgactacatcaatcgcgttgtgaaacgcttccgcttacggtctacaagggtacatatacacactctccccctttcgttgctatgcatctccatggatagatcttgcatgtgcgtataattttttttgttttccatgcaactattcccaacagtgtgaccctgcaggttcggtgaaatatagacatgactgaaacacctttcgtcagtgaccaatagcaaaaccgtggacacccatattgactgttctactcacacgaattattattgagtgaacctatagctaccatatgctattcgttttgcttgacaatactttacaaacccgaggtgagatatatcggcatctccattagtcaacacatggtcactatgtgagtctcctcgctaccacttttgttctcttttctcattatcatgtttcgacatccccatgagcaagtcgataatggctacatcccaccaagatggccctaagagtatctctccatcgccggaggagcaaatcctagtctcgagtatctagtcccttgtcatagtttcgtatgaacctgtaagttgccgctatgatcaccctattatggttgacattagaaacaacgacactctcatggtctaaagaattatgcatacgttaactttctctgtgttttggactatagacttgtgacaaaatcatctcctagtataacatataacttgggtcgattcaacacaagggttcttcaaacatcgggcccttaatattgtcggcatcatcgctacacttaactacgcacatgatcagaacagaaccatgatgcaacacttgagctagccctagagtcattttctattttaccgtttattattctacagatacacatgagttttcctcttagcctcatggactcgagaacaatatcagttatagcatagaatataagcattaatcataaacttagaaataattaatacttttaggtaacaccctactcctattatttggactagttaattgcctcgtgcattgctacggggtttggggatcaaaggagggtccttcattcgatcgatgaacatgtgagtaccaacgagggctacgtacgcattcccctttaataatagagatgtagtgcaatatatgccggtcgatctcagcggagtaacttggttacaaggcaccaccagtggaggcagtgagaggaagtcgaaggtttatgtgacgaaccataagtctcgatgcgtgtggaagaactgaaatgatctactgattcaacttaccaactaagaggccactagcctaccttatatacctggctcagctagggttacatggctcatgtcgatttacatatgggttcttgtgttaccgagttgcgttggacgccaaatcgcttagtcagccagtcggtcgccttgtccttcaagatggtcgtggcatcatgtcttgatggtgggcttgagtcgtcgatccggcccaagacacannnnnnnnnnnnnnnnnnnngtcgctcgacgggactccttcgggcaacgatgagtggctagggccgtggctctcgaggtttttaggtggcgggaagagggtgtggctccagagccactcaagatgagtgacctgagaggccggctagggtaaccaactattgttgcgaccacttatgtcgactcccaagattactgaaaaaggctttcaccccgctttatatatagaaacaacgatccacaagcatcacgatacaaactcacgccgccaccacacacgacacacacacccaaggcgagatacaaaggtgacaagcaccgccacaccaccctaacgactaccaagcnnnnnnnnnnnnnnnnnnnnnnnnnnnnnnnnnnnnnnnnnnnnnnagacctccaattctccgaggagaggtgagacggaaaatgacggagcaaggactccaagatggtgccgcctagaaggaaacatgctgaggtgatgtgtagttgaattgacatatacacctgttttgctagatttttttacttctttgtatacctttcttaggatatatactcagaattttcattcaagactgactattggtcggggaaagcatgccccctgtcagatataccctttagctctgcagaatccaagtgtaaacctcattttgtaagtatacattccattagcaagtgtagtagaaaatgtcactgcttttctgtttatcgttctatttattcagcgaaaatgcccacggtgatgccgcatgggggtgcagatgcggcgtcctcgagctgccatacaaaacgggaacttgagctaacaaactagctactgacctgactacggggtgcagccaatttatttccaacaataagcgaacgtgtagccggaaaaaaaacaataagcagacgatttatcatcccgtgctgttgtgcatggctcatccgggcaaagatcataacgtatgcgcaagttttgaaataccaaccccgcaaaaaaagagttttgaaataccaacgaacgtgcagcaaaccacagattctaaattgaacaagcctaccagatgcacgggttggaaaaattatcaagtttgcactacagttagttttcacatttttgaaagtttggagtcctcctataagaactccaacacggttcaccaaatcgtccccaaatgtctggactacacggtccggaaacttttagccatccaatgggagacatccaacatcggcagcatggtccggacatcccctaacttgaaaaaaaaactaggttttctttttttgagctatggaaaaaactaggttcgtttcatgaacagtgttagagttgcactgggcgccactttcggtcctcgtgcccactaatggatgttctgggcccaggcccagtaacactcccttcactcaccgacctgctctcgctagctgcctctgctcgtttcattttatttcaaccagctcactagccgacttaactgttcacttttctagaaaaagtttgtaagttcgagaaaaagtttgtgtattcaaaaaaggatcacggatttgaaaaaaggttcatgaatttgaaaaaagtttgtgcattttgaaaacagtcatgaatttggaaaaaatgtgagttttaaaaagttcatgaatttgaaaaataatcaaaaatttgaaaaatgtatgcctatttgaaaaccttaatacaagtgaataaatttgcggattcaaacaaaatttaggaatttgaaaattcgtgcatttggaaaatttcataaatttgtaaaaatgagaattttagaaagttcatggcttttgaaaaaagttaaggaattataaaaagtctacatttcaaatttaaaaataaaataaaaataaaaacaaaaaactaaccaaaagcgttgaaatgtgcactactcgctccccatatgcgcccatttgggctggcctatgtgcggggtggcgcgccctttttcattttctctttcattcctcctttttgtttccttttccctttttatttgtttccttatttacctaaaataaggttttataaaatattcatgatgtcgaaaatgttcataaattttagagaactaaattttaaaatatattcatgaccgaatattctcggatttcaaaaaatgttcacaaatttagaaaattattcacaaaaatgttcactactttggaaaaaaaacttcataaatttcacgaaatgtttctttattcaaaaggatgttcgcaaacatctaaaatttaaaaaattgtatttgatgaacaaattttgaattgcatgaaaattattgtatccgatgaacatttaaaattcatgaaaaatttcgagaaaaatatgatgaaagtttttctatatttgatgaacaatatttgaatttgatgaatagttttgaaattgatgaaatttggtaaattcggtaaacggttttgaatttgatgaataaaaatattcgagaaaataaaaaaataaaaattaacatggaaatataaaaccaaaaggaaaatgaaaaaaaactagtatgtgaatgttctagaatgttcccaaaatcggaagagggattgcgcgttgagtcgacccaattcacttagcgagtgtaaggggtgggcgtttggtcgacatgtgtcaacgcacaggtgcaaatgggaagaaagaaaaggtaaatggtccgagaccaggtcgaagggaaggtgtgcactaggtttgcagaaacaatagcaaataattctaaaaaaacaatagcaagctcgcaccaaaagtaccaaattgggattggtggaagtttgaattttaatcattttcgaagatgcctgggccactatttagaattttgggtcagtccatcttgttccgcacaagtacatgaagtgtaactatgtggtggccacagacggacttgtggttctcgaagtatttccacctaagggctcggtcaaaggaatttcatagggttcttggagagttataattttttttctatgttggtcttttaagtcataggattgaatcctatatggtttctccctacgaaatcatgtgcactacatctcataggaaatgtaacattcactccaaccttttttttaaatttctttgtttttcctatggcatcaacactctctgttgatcctacaggattcaaatgggcatgtcactttaattctgtaattttctatccctgcattttgagagccatgccattcaaagagtgcctaattttttctagcgcagagctacttcccccgccctagataatgggatatctgtggcccgtgcgccaaattgccgcactccaccctggatcggtgccttgggaaaaaaattgtattgattacctagaactaatggtgtagataggtcccagacttccttaggtggtcggctgggtcatcacgtcggatctctttgctgactgtagagccctcaacactgtttaggctacttacagtgggagtaacatagatagtaacatcacacatatgttagataaactcgatgatgtggcatgcaataaatgaagaaagagaggaaaatggtaacatagctagttactagtagtatgagtaacatcacacatatcaaggcaagatgaatctatagactaataaatgaagtattgcatgtcaccacacatatgttactccccactttagaagtaataacatagaccagtaacatatgcatgttactcgtatatgttactacccattgtggctagtcttagcactatagcgtgtatttggctcagggcttgcctaggtcctggcctaatcttgatgacactactgcctggaattattcctattactgaaaatagtaatattgccttgtcttgataacaattcaatcgaatcggcatttttctactgagttgcgattttggtccagtgcaccttatacatgataaaaaaagggagaaaactctattctagtcaattagtagtcccactcgggatttctttgaatttgcacgcatagccacacacgacaggtcagacctatccgtcgccgccaacttttccagccctctcgtcgccgccaggccaagaccatgtggctcctgcaaaggcaaggaggatctctccacaaaggagtttgatgcagcggggttggccggctatggcggatcgtggctgctggcgacgacaaatgtatggctaggcggtggcagttctggactagcggcacacgcttgcgatgttgcctatgtcgacaaagtccaaaggtccctaaagacggggtcctagatggtggcgggctcgacctctagcccagatttggcgtgcaatggcatgggcgtcttgatttaggctggtttagggagtctatgtgtcttgggcgaggcggcgttgccccaggttaggaataatgtcctcccatcctatccatgtttttggttcgctttgcttgggaggggcatgtggatatgtatctccgatggatcctcctggatccagtcggctccggtctccggtgaatccttatggattcagtatgtgttcgtggtctttggagtatctacacgatcttttcggcatcttcttcttcggggtgatgatttgttacgtagattgaggtcgcatgcatcttctgctctacatcaacgattgtctgatctttgcttctcctcatacatagaatcgatccatcccacaaaagttttcacgaccacccacgagccttagatggccctccctcgaccggcattcgtcatttccatgggctttgcctccaatctggagctcccgacccctctacaacttccgccaccaccccatccacctccatagttcctgacggcatcgccgcctacaactctatgccccaccaaagttggtgactacaagtccatgtaggtgtcatcttttgagctcctgcgagttgtgacatgttttgtgggcctctactgctgacaatgagggggagccagtccctaagcacgatcagcttccaccaatgccattgacttctagagagtgtgcatggagcgcaaggcggaagcgtgtgggggttggcaggaggtgatgccgcaccatagcccatgtcgtcaacttcgccaaatttggctttggcttatcttctattcccccacatggcttcatgatagattctacagatgtctcgcttctctgcgccgcacgtcagattgcatagactcctttaggttctctcatttctttccaaacagtcattgggcttgtgataacatctggcatcttctcagcctattggaaaaccctactgcctcattggcatcctgcctcgtcatcaagcatcctcaagctccagctctatgttaggcccattccaaggtgattcatcatgattcatgggtgtcggtcatcttctctcccactatcttggcaagcccaagtaatgtggtcccgtggtccgtctttgttgtgcaagccaaactgctacactctcgggtgctagcattggcttcgcttgagctgaaggaggcggtccaccctcctcgtgacgtcgctggatatatgtaaggttggcttctttgggccgggagctttctagagcgtgtggaggttgctctgggcaattcctctcttgtacctaaggtgatgctgaccccacaagtgttgcctcctattgtagagcatggggttgactttgatgaccagagggatttagagctctatgatcgcttctcccctcgtgttggggcaagtttattgtcactacctgcctttccctctatgcgtactggctcgctagatgaggctactagtggagtggggcttcatgatcgtttctccctcgttctcaggctatctcactgtcgtcttcctttgaggatatgtctatcgatgtggtcatcactctggtgctgcagatcatgctcgagcttcaggggttgtgttcataactggttctacctctatccgtcgagcaagtcaagatgcactcgctcgatatctctgatgtgcttttactacccagccaggcggtcaacttcgaggagagccatgatttggactatgcagtctctcacttgcctcgtggtttctataggttttgagcttgttgcatccgagtgttggtacttgtgcctgtggctctctttggaaaagagatttgcagtttccttactactttggttgatgctaaccctggatcaggcaagatgattggttgtcttttgaaggggaacaccatcacgtaacaaaacttttcaaaatgaccgagtgattgtcaatatctctcctaattggtgacatgaacacctccacatcatccacatagagggttgatctcaccatggcacccctctccctcgaaatctcgtgccccttccgtgttgccaattcgaggatttgttgtagaggctcgatcatgatgataaagatgagggggggaagaaggggcacccgtggcgagagtcccgcccatgtttgatggggggggaccacaccattcaagaggatcctcgatgaggaactacaaagtagtgtcgtgatccaatctctaaacttgcttggaaaccctctccatggaaggagatctaggatgtattcccatttgacagaattgaaagccttacggatatcgaacttgaagagtacggatgaagtcttactcctgtgaagccgacggacaaggtcccaaacatgcatgaagttttcatgagtacttctcctcttaataaaggctctttggacgttggagacgagggtgttcatgtgcgggccaagatgaatggaaaccaccttcacaataatcttgggaatgacatggataaggctaatgactctatagtcggaaatcccttccgcctcttcgagcaagtgcacaacattttgcggagttgagtcaatgaagatttgaagtgtggagggagtcaaagaggtgtatcaccctcatgatgccacacttggtcgtatcctaacttttttgaagaagactcatgtaaagccatctgacccagctgccttatccttggcacttgatcgagtcaaagagtgccatgatgtcacccaagatactgagagccatccatcaagtaaccacagacgccttttatgttgcgacactcttcttgatttctccttcaagatgggtcgagggctagtgtcgcctctctagctactatgtcatatatgggtacctaatttggaaaaattctatgaacgtcatttcttaagcatggattgggtagcataaagttattttggatgatccaggaaagaggtagttatcgaggagtttttgtgtgtcctgtgttttcgagtcacgtttggtcgattttagggcaaattatgtacaagatgtcatatatgggtacctaatttggaaaaattatgtgaacgtcattttttaagcatggattgggtagcaaaaagttattttggacgagccacgaaagaggtagttatcgaggagtttttgcgtgtcctttgttttcgagtcgcgtttggtcggttttagggaaaattctgtacaagaatgtcatatatgggtacctaatttggaaaaattctgtgaacgtcattttttaagcatggattgggtagcaaaaagttattttggacgagccacgaaagaggtagttaccgaggagtttttgcgtgtcctttgttttcgagtcgcgcttggtcggttttagggcaaattctgtaaggtcacggatgtatgcaacaacttccataaaaggtggttcccatttgttgcaaccacttaaacatgggaaatattcttcaaagtttgtcagcaactagaacatcattgccattgagatatttgggccaactcaagaaggtggacctccaatttcactgaatcttgcacaccattattatgagcgaggcaaacctttgtatcacccccggtgccaagaagctagtgacaattatattgcgagagtaatcattcatgtttgcatgagggacagttgtaacacaaacaaactaagttcttcttttcttaattaatagatgaggcaaagcttatgtctccatttcggaagaagaagatatctcacgcatccaatgaattttcaataggcatacgtgaccgtcacagttcgtcactgtcgatgacttctcgatgcagctgacaggactaagtaaacattctctatcactccctcctttcaaaataagtttaatcaaatttatattaaaatatatttcacaaaaaaggtttatattaacatgtacaacataaaaaaaaacttgacattgtagatcttagcagattttttagacttggttaaactttaaaggtttgaattaatacaatcctggaatttcaattattttaaagcggagggagtagcacggttgtactgcgcatacatacatacctttccttttcactcagattatacaaactgattttctccccatcttatggcccaaacaagacagcacgcgtcacccacgcttttgatagatacaagccgacattgacaatttttatatcgagcagtacaactcatggtagttgtaaacatcttgggtagggaggccaagcctagcgtacaaacaatcgttttttttctttttttgtttgacaatggtatatgtaactctctctctctctctttctttttgtggttcgacagtggtactagcaattcttgtcctcatggtttatgccttgacacatagtaccaccgatgccagagaccaccgttcaacgctgcccgcatacatttcaaccacgattcaaactaaccgaacataattcaggcaaaacagcagaattcattcaagtttggttataatttacataaaaaggtcgatatttcgaccgtttgactaaaaaattaggaaaaaaaacataaaccctatatcagacgaccacctcgtatgcgtggccaccctactttatcgtgccgtcgtcaccgtccctccaacgacggaacgacgccggagggcagcaacaaccttgtttggtggctgcctgccgcttgcagaggaaggcgctctgcctcctgccgcgcgatgtgggggccgccgcggccactgccaatgttgccttcgggggaaggggcggaccggaagaaaatgggatagggagggtggatttagtgggctgggggtgtcggagtactacgtggctgttgtccaaactttctcaaagcccctctagtttgtctggaaaaaacgcgtctggaccgctcggcggaccgatacatgtccgtattggatgtcacggcacgtccgggcggacagcggtgcggacgtatgtggatggtttgaaggtccgtgttaaagatgcccttatatccacaacatcaaatacatatagtaggaatttagtagaacttcaaattggctacttaggctgagttcggtttctctcggctcctttaactccgctcccggagcggatcatactttagcttaactaggcagagctgccaaaacccagctcctcaactccacggagcggggaagttccaaacagggccttagtatatgtcctgtcttgcatagtgaaaagagggaatgcgtagacgtgaggatcttgctgcatgcaagtcaactctcgccttgtcatgtctgttgtgttgtcagtactgcaatattctgtaccgcacttgtgacgtaggtccctgctggctgtttgctgtgcacgcgcataaatatatggtatccttcttctagccccttcttccccgttgcatcagccataagcttggagccatccatctttctcccgagccacctgcagatctttaccagtgtggatcgaacaaagcataagcattctttgtttgttgtttcccaggtgagctttgtgtcttggtgtttccctgtggtttcttggctgtttcgctcttgctggatttccctttttccaggtaaaattttataatagattggctttgtgttgatgccaatgtctttgcccttttgtcttaggtgttttgtccatcccctttctgcggaaagacggttgttaactgtaagcaaaaggctgggtttgtgagctggtctgtatgggctgtgtatcatcgaagcagttcagacgagctccaccgcatgaggatgcggctcttctggccaaagagaccacatgtgagcttctgttttcctttctggtgatttgttcagtgcaaaaatgagcccagcatacgtttccgctgccgtttaggagtaatttctaatattttcttcccgcgtgctcgcagtttctttgaatgaagtggaggccctctacgagttgttcaaaaagattagctattctatattcaaggatgggcttattcacaaggtatgtatcatgtctttctcatcatcttcttctagtcagacggcaaattcaccatgtgtttccttctgcaaactctaaccagggtcaatttgcaaactgccattttgttcaacaggaggagttccaacttgctctcttcaggaacagcaaccggaagaaccttttcgctgatcgggtcagtctttaatttctacaccattccaacgctccaaatagctgtaatttcttagacaatttgggtgtccttcaattttggggtgacagcctgaatattatctgtgtgactgtaaaattgttccaaacttatgcacaaatatcaacttgtttatatgtgtgttggactagtgcagatatttgatctgtttgatctgaagcgaaacggtgtgattgaattcgaggagtttgttcggtccctccacatttttcacccggatacacccatggcagacaagattgcatgtacgaagcgaagccagaccccttttcctcccaatgtttgtgtgcgtgtgtgaatgttttagaaatgactggcatttcataaattctgggtgcaattcaagtagcagtagcctggtatatatatttgggaggacttctgtctgtatatgcttctcctatctgacatctgaagtattccttctcgcagttgcattcaggttatatgacctgcgaggcaccggcagcattgaacgtgaagaggtttgtatcttttagttactgaattgttctgcacataatgttttggaaacagttctgctcatagtcctctcaactgaatatagcacttcaagatgttttgaacatactaccacacactgcgatttgccgatttagatgcttattatccttcggctctgtcgttacattcagctccactggaaaaataacacaaagcccattacatacgcgaagtatagcacccacactaccttaacagtttgacagtttgagcacttactgaaactggccgttcatgcagttgaaggaaatggtacttgcaatcctgaatgaatcggacctccttctttctgatgatgccgtcgaacagattgtagatcaggtacatacaccacatccagatgaaatgttgctgcacaagaatagttaacactatgcatacatttatgctaacttatattctgacagacattcaagcaggcagacctgaacagcgacgggaggatagatcccgacgaatggaaggagtttgcaagtaagaatccagccttgctgaagaacatgactctcccatacctaaagtaagtagcttcatattttcagctttggaaaagagtgactgagaacattactcttgactgacacctgagcatattgtgatgcagggacataaccatgtcattccccagctttgttgtctactctggagccggcgacgaagagttgtagcagtagcagccatcggcacaagcatctgtgactctgacctctcaagtctcaggcatacaacatcatgttcacagtacaaatttttggattatgaaacaccgctttgtagcggaaactgcaatagcagaatgtgtagtgagaaatggtacacatcacctcaacagctgggcaattttttttgtcaccgaatgggctaatcactctcatatttacatttgcaatatctaacagtttggccatatagattcttcacagtacatttacaggcgaatatacgtgcatttaacctgaactgtcaaaatctgactactgaactccgtgactctccatacacatttcactcaaaatttgactattgaactccatggctctcgatacacatttcactgtccgactcgcagaagttctttgtcgaaaatgagaacacagaagttgagcaagtaactgcaatgacgatccaggttggttctactgcttcagcttcaccatctcctgcaagttcactggatagcacatcctggtgtgctcaccagaccacacgttttctgccaggatccggttcacagcatcggtcgggtggaattcatcccaccacacatggcttgacgcgtcgctgcacgccatctgtggaagaacacagatgaacagcccgccatacttgccaagcccacagcaagcatcagtggtggtcagaaagcctgcagttgcaattccaccaaaatgagaagacttagcactccatttaaatgtactacaaatgttacagttgcgaagacccggaactcaatttaaatgcagtactaattatgcatttgctcaccatagcggtcacggttctctagtatgtccactgacccctcaaatgtatcacagtaactgatcatcgagtctgggtactggcggatgaactcgctggacatgtgtctcagcccatagttgaactcgatcacgacgttgttgatgtagtcgatgcattccccattttggctgccgtagtctgagaggaagtgaggggcacagccaacaggaggaagacccatcagcacgaccttccggacattgatattgtacagattctgcataaatcatcattgtcagaaggtagtaggagacacatgagtccagaactagtttagacatcaaaagtggagtaatctgataagaaccacatgtatagaactaaacagtaatggatgcaaaatggcaattaatttgctacggtattgcgaagaataaagaaaccgaagcaatccattacaatttgcatcagcatagaaatgagattatggaatgaacacaaagaaaatttctacatacaacaaactactggtatatgcatcttcaatcaccatgcatcacatgttctgcttaagattaggtaacagagataaattaggcatagagaatcaaatgatccatgacacatgaactgagaagcacacttgtaccatggtcagatcatgaacagttcaatgcagcgagataaatgtgcaaggtgttatacagttatagctgaaaatacaacagactgttttttttagttttcaagcaacgagcctcctcatcgatttccattaaagaaaccggcatagttctacagagtatgagaaacattaaagacacaaaacaggaagctaaacctacaagcatacaggtaaagcaactaccaggaaccaactgcacacaggcagatcaaacacgacgatcaagcatgagcaccagaaattacagaccagtgaaaatacatcaagttccatagataaaacactgcatcccagaatgagggttccatccatgaattctgaaagtacaacagacttaacttgaacccaaaacagatggtagcacaaacaaaagtgctgcctaagcatttcagaatatatcacagtttaatatagtatgctgtggtgtagctcgggtagcatgagtagcaccttgtgtagtttcagtttttcagttgggctgcttttgtattgcttcgttttcaatagtttaggacttttgcttttcagggttgtatttgctttgtactttgcgtcatcatgacgccttcttctaatatacaacaacacacatttaagtgtgcatttgagaggaaaaaaaagttttaagacagtataatgtcagtttggtttgagcccatgtttgccttaccaaaatggccaaaattttggtcaaagtactggtccttgagttggccaatgttggaaagaaaaatgaactagagttggtaaagagctattggcatgcaaaaaaattggagagaagttttggctgccatccatatataaccttaaataccaactaaacaccattacacctcattgtagaatcgggatcatcaatccaaccatatctatttcatattttatcaaaatgccgcatctaacatcagtgtgaaacattttgcaatcgcaatacctgtgcaatattgcaagaaataaacccaaattcgacagtaagaacaatttgtatgtagaccataggtgaaggcgttaagttctagaactgcgaaccaacaaggaaacctcattcatttaaatcactttcatgtgttaatttcccagataatgatttctagtgtatccacacaagaaactaagttatgtccaaaacggaattcgtagttacagatatccctgactacgcgtggtgcgcatctccacaagtaaaacaatgcacagcacactgataataaaaaaggaggcgagcaatggtatacagaacaaactgaggagcaagaagcacaccttgatttcctgcctcactgcattaacaaggagctgattgaactcccatgggagataatgcatctgcacgcctgacacattgcgcaggtagtagtggatgaagtcgttgctcccgatcgacacaaagaacaccgacctcctgaacaggtcgactgtagctgcctccccaagagccagcgccaactgctcatatgtgtcctcaacctgctgcacctgctgggtcagcgacacatgcatcccctgcaagcgcaaccaaaatcgcaaaaaacccaaccgattagcatcaaatttggtgtgaagaaaccgcagattgacagcagaatcatccccaaacagatctctcgacagcaccaagacgtaattaataagattgacgcagagttattgggagcggtgggagaacataccagctcagagccactgctggagagaatgccggctgccgcggaagcgtagttgacgccttggatcattccgccaatgttgctgaggccaacaccgccgccgcccatgcgcatgctctgctccaggtacggaggcacgaagggaagccccagcttctccgctgtggtacaacaacaaaagggatcagaaatcaagagaaggggaactaggaatgtcgcatggggctgccatattcctgcggattcagaccacagcagtgacagaagattacacggaacaatcgaggagaagaaacaaacaatcatcagtagtcatgatgaaccatggagacctaataaaccgaaacgatccgcatctggaagcaaggacgcacaaaatcgagaaaccagcaccgcaccaatccatggagaaaccgagagaagcgagcagagacgaggtcgtgagcgacgcaccgaggtagtcgacggggatgcggccgttggagaagcgcccggtggggcggtgggtgtcgaagtcccggccgtagggctcgcggtcggcgcgggcgagcgtgccgaggtagttgttggtgccgacgtccgacgtggagtcgccgatgacgaagagcgcggggacgagcggggtggggggcgccgacggcggcgcggaccggggggtcgaggcggctgcggagaggggtagagcggagaggaggaggagaaggaggaggaggaggcacacgaggtgagcgagggaaggcgccatttccggcgaggcggtggcgagttcgcgcgctctgggttgggaaaggtggggggagaagaccggtcgggtttatggaagaggatggactcgccgcagactcgagtcgctaaccgcagtgaggatggaggaaaaggcctacatttggtgggctagggctatggactagagcaagtcagcaaacagtagcggaaacgggccccttctcagccgattgatgcacggccccttggcctgtttgacatccttacttgatcgccaagaatgacaaaacgtataagaatcctcaaaaaaaaaaaagacaaaacgtataagaataggaggtctaggatagggagatacccattggtttctggtatctacactctatatgggattttagaaatatacaaaagaagttttatagaataaacttactttcttttgcactaaaccaacgttgactttaggggaaaagttatttgttatcacaggtcactgtttgcactatttttttgacagaaattttatttttttaagaagtcaaaaggccttcttatttttgtgaattttttttgacaagtacaatgaaaggtcaagtttatttcaaaaatatgttcagttcttttttactttttatttaattacttttttgcatatagggtgtatatacacccatagaccaaacgtctgtgagcacctaggattcatagatactgagcttttctacttggctatgctcacgcgttaaacttgaagggttctttttttagcaacgggcaggagctctacttttcatcatcaagaagaggatttctctactttttttagcaaccgtcaggaaattctcttctttttttcccatcaaaaagaggaagagaattggtcagtttattagggaaactagtcgaaagcctgacaaattggtcatagggtgctcgaagttgtctattgcccgagagttgatttcttaaagcttgaggtgggatcacacccctcaaaatttggcgtgttatcgtgcatgacaaatgtgttctacaacaaggacttattaagaggatggggacaagaggtcaaagtaacatataataagcaatggccacgggaacgtcttctacgattgtactgtattaaaaatagtaatggctacctagaagggggcatttcagtcaatacgatgtttgatcagcgatggtgagcaagttcattgtctcttcctcgaccgagtgggatggtcagaagttgcaacaacatttttacctatggatggtgagaacatgaggaacatcccactaccactagaaggcaaaatgattttcgtgcttggttccatgataaaagaggtttcttcattgttcggtcaacatataatatgcttatggtaggaccaaagttatcgatcagaggtgaggtgaataggagatttaacaaattctccgagaaatacaatccttcaaagaaagccattgtaacagatcaacacactagtataaaactacattagaagtgaaaggacctacatgtcgcctagagggagtgaatgtgcattttaaaaactcttatggatttgaatttatcataatgtgatatttttcaagcatagatccttaatatgctaggctcaattaaatgcaccaacaacctacacaagatcctaaattttgggagtggcttattttttaagatgggagaggcagcttattttttacaccgcccaaaagaactgtccctaaacaagataagcacttgaaggaaactagcaacgagggtgacaagtactcgccttgaactcgacaacatatatcgtgagacaaaaggaatatagcacatgccacattgtcaaggttcatcaaatgattttgcacacatgggagtcgacactttctgttagaagccactaccgactaatcgagtcggatgggatccgagtcatgtccaagtctttagtcccaaacaagttgcagtaggctagagctgaaaacccataagatcttccaatcaagtcatggttccgacacgtggatagctaacttcacgcactcatgtccatggatagttctttcttgacattccagtcctttggaacttcttttatggactcctcccacatgtcaagtatgatccaccccgacctctattgacattatcagcacgctttaattgtttgttatgcactagagtttctagaggtctgtgttgaatatgctcaaaccatctcagatgatattaaacaagtttctcttcagtatgtgctaccacaactctatcatgtatatcgttgttccggacctgatcctttcttgtgtggcaacacatccatctcaacatgtgcatttttgctacacccaactattgaacatgtcgacttttagtcagccaacacccaagccatacaacattgcgggtcaaatcgtcgtcttatagaaccttccctttggctttgtagattgcaacctcttgatgacgatctctttatagcaacaatgatcagaatggataatttgaaatttcgtccggagtgaaatcgccaaaaagtgtgttctcacatgtcacagtgtacccctgacgactagcatggtacaaaagacacgtcgctggaggaacttcgccaggagcgcagtaagcaagacatgctggtgagatcttttgagaaccaaaaaccccacacacccggaagggaacccgccagggcacacgatgcctataggatgccctaggttggcttgactgcccctatgtcctcggggttgaacacaaagaaaaacgaagaacgaggaagaaaaaaacaagggtaaaggagaaaaggtaaagataaaagtgatatgttggtctgacggattgtgtgttgttgttcaattggccatcacctctcatctatatatgaggcacttggacttatcatacaagaaaatgactccaattctagttcgaactagtgggtcaggtcaaaattttgtcacattgcatcttgcggaggtcgcatatgaccttggattgagattatccaaaaaaagcaaagttgtttgtttcgacgagaagaattactttcatgttgaacactttttgcttgaagcattcttgaacctgatatatgccatgctttatgaaaggatcgagaagaggtgtctagnnnnnnnnnnnnnnngggggggattagacactaagtaccaaaagttgcagttttgaacttctttaagtttaagtggaatttaggcacaagtttaacactcacaatacataacaagcaagcatgcaaagagtatatgagcagcggaaagtaaagcatgcaacttgcaagaatgtaaagggatgggattgaaggattcaaacgcaattggagacacagatgtttttgtcatggttccgataggtggtgctatcgtacatccatgttgatggagacttcaacccacgaagggtaacggttgtgcgagtccacggagggatccacccacaaagggtccacgaagaagcaaccttgtctatcccaccatggccgtcgcccacgaaggactt

>TaCBL7_1DL_2195979

gtttctccatggatcattggtgcggtgctggtttctcgattttgcgcgtccttgcttccagatgcggatcgtttcggtttattaggcctccatggttcatcgtgactactgatgattgtttcttctgcttgattgttccgtgtaatcttctgtcgctgctgtggtctgaatccgcaggagtatggcagccccatgtgatattcctagtttcccttctcttgatttctgaccccttttgttgctgtaccacagcggagaagctggggcttcccttcgtgcctccgtacctcgagcagagcatgcgcatgggcgtcggcagcgttggcctcatcaacatcggcggaatgatccaaggagtcaactacgcttccgcggcagccggcattctctccagcagtggctctgagctggtccgttctcccaccgctcccgataactgcgtcaatcttataacgtcttggtgctgtcgagagatctgtttggggatgattctgctgtcaatctgtggtttcttcacaccaaatttgatgctaatcggttggggtttttgcgattttggtcgcgcttgcaggggatgcacgtgtcgctgacccagcaggtgcagcaggttgaggacacatatgagcagttggcgctggctcttggggaggcagctacagtcgacttgttcaagaggtcggtattctttgtgtcgatcgggagcaacgacttcatccactactacctgcgcaatgtgtcaggcgtgcagatgcattacctcccatgggagttcaatcagctccttgttaatgcagtgaggcaggaaatcaaggtgtgcttcttgctcctcagtttgttatgtgtaccattgctcgcctcctttttgattatcagtgtgttgtgcattgttttacttgtggagatgcaccacacgtagtcagggatctctgtaactacgaatttcgttttggacataacgtagtttcttgtgtggatacactagaaaaccattatctgggaaattaacacatgaaagtgatttaaatgaatgaggtttccttgttggtttgcagttctagaacatcaacggcttcacctatggtttgcagttcacagatggaataatcccattgttcttactgtcgaatttggggttatttctttcaatattgcacaggtattgcgattgcaaaatgtttcagactgatgttagatgcggcattttgataaaatatgaaataaaaatggttggattgatgatcccgattctacaatgaggtgtaatggtgtttggttgctatgtaagggtatgtttggatggtagccaaaacttatctccaatttttttgcatgccaatagccaatattggccaactctagttcattttcctttccaacattggccaactcatggaccagcacaactttgaccaaaattttggcctaactttttggtaaggcaaacatgggctcaaacaaaactgacattatactgtcttaaaactttttttctctcaaacgcacacttaaatgtgtgttgttgtatattagaagaaagcgtcatgatgatgcaaagcaaagcaaatacaaccctgaaaagcagaagtcctaaactattgaaaacgaagcaatacaaaaggcagcccaactgaaaaactgaaactgcacaaggtgctactcatgctacccgagctacaccacagcatactatcttaaaatgtgatatattctgaaatgctcaggctgcacttttttttgtgctaccatctgttttgggttcaagttaagtctgttgtactttcagagttcatggatggaaccctcgttctaggatgcagtgttttatctgtggaacttgatgtattttcactggtctgtaatttctggagctcatgcttgatcgtcgtgtttgatctgcctgcgtgctgttggttcctggtagttgctttacctgtatgcttgtaggtttagcttcctgttttgtgtctttaatgtttctcatactctgtagaactatgccggtttctttaatggaaatcgatgaggaggctcgttgcttaaaaactaaaaaaaaaacagtctgttgtactttcagctataactgtataacaccttgcacatttatctcgctgcattgaactgttcatgatctgaccatggtacaagtgtgcttctcagttcatgtgtcatggatcatttgattctctatgcctaatttatctctgttacctaatcttgagcagaacatgtgatgcatggtgattgaagatgcatataccagtagtttgttgtatgtagaaattttctttgtgtttattccataatctcatttatatgctgatgcaaattgtaatggattgcttcggtttctttattcttcgcagtaccgtagcaaattaattgccattttgcatccattactgtttagttctatacatgtggttcttgtcagattactccacttttgatgtctaaactagttctggactcatgtgtctcctactaccttctgacaatgatgatttatgcagaatctgtacaatatcaacgtccgaaaggtcgtgctgatgggccttcctcctgttggctgtgcccctcacttcctctcagactacggcagccaaaatggggaatgcatcgactacatcaacaacgtcgtgatcgagttcaactatgggctgagatacatgtccagcgagttcatccgccagtacccagactcgatgatcagctactgtgatacatttgaggggtcagtggacatactagagaaccgtgaccgctatggtgagtaaatgcatcattagtactgcatttaaattgcgtgccggttcgcaactgtaacatttgtagtacatttaaatggagtgctaagtcttctcatttttggtggacttgcaactgcaggctttctgaccaccactgatgcttgctgtgggcttggcaagtatggcgggctattcatctgtgttcttccacagatggcgtgcagcgacgcgtcaagccatgtgtggtgggatgaattccacccgaccgatgctgtgaaccggatcctggcagaaaacgtgtggtctggtgagcacaccaggatgtgctatccagtgaacttgcaggagatggtgaagctgaagcagtagaaccaacctggatcgtcattgcagttacttgctcaacttctgtgttttcattttcgacaaagaacttctgcgagtcggacagtgaaatgtgtgtcaagagtcatggagttcaatagtcaaattttgagtgaaatgtatatcgagagtcacggagttcagtagtcagattttgacagttcaggttaaatgcacgtatattcgcctgtaaatgcaccatgaagaatctatatggccaaactgttagatactgcaaatgtaagtatgagagagatcagcccattcggtgacaaaataaataaattgcccagctgttgagctgatgtgtaccatttctcagcgcacattctgctattgcagtttctgctacaaagcggtgtttcataatccaaaaatttgtactgtgaacatgatgctgtatgcctgagacttgagaggtcagagtcacagatgcttgtgcagatggctgctactgctacaactcttcgtcgccggctccagagtagacaacaaagctggggaatgacatggttatgtccctgcatcacaacatgctcagctgtcagtcaagagtaatgttctcaggcattgttttccaaagctgaaaaatatagagctacttacttcaggtacgggagagtcatgttcttcagcaaggctggattcttacttgcaaactctttccattcgtcgggatctatcctcccgtcgctgttcaggtctgcctgcttgaatgtctgtcagaattgtaagttagcataaatgcattcatagtgtcaacgattcttgcgcagcaacatttcatctggatgtggtgtatgtacctgatctacaatctgttcgacagcatcatcagaaagaagtaggtctgattcattcaggattgcaagcaccatttccttcaactgcatgaacgaccagtttcggtaagtgctcaaactgtcaaattgtgaaggtagtgtgcgtgctatacttggtgcatataattggttttgtgttatttttccagtgaaacaaaatgtactgacagagctcaaggataagcatctaaatcggcaaatcgagtgtgtggtagactggtagtatgttaaaaacatcttgaagtagtatgttcagtagacaagattatgtgcagaataattcagttcagtacaaaagatgcagacctcttcacgttcaatgctgccggtgcctcgcaggtcatatagtctgaatgcaactgcgagaaggaatccttcagatgtcagataggagaagcatatacagacagaagtcctcccaaatatatataccaggctactgctactgaatcacacccggaatttatgaaatatgccagtcatttcttaaaacattcacacactcacacacaaaagaacattgggaggaaaaggggctggcttcgtacatgcaatcttgtctgccatgggtgtatccgggtgaaaaatgtggagggaccgaacaaactcctcgaattcaatcacaccatttcgcttcagatcaaacagatcaaatatctgcactagtcaaatacaaatataaacaggtcggtctgatccgagatatttgtgcataagtttggaacaattttacagtcaggcaggtaatattcaggctgtcacgccaaagttaaaggacacccaaatcgcccgagaaattacagctattcggagcgttggagtggtgtgaaaattatagactgacccgatcagcgaaaaggttcttccggttgctgttcctgaagagagcaagttggaactcctcctgttgaacaaaatggcagtttgcaaattgaccacatcgtgaatttgcagcctaactaaaagaagatgatgagcaagacaagataggtaccttgtgaataagcccatccttgaatatagaatagctaatctttttgaacaactcgtagagggcctccacttcattcaaagaaactgcgtcatgcatgcgggaagaaattaatagaaattaaacggcagcggaaacgtatgctgggctcatttttgcactgaacaaataaatcagcagaaagaaaaacagaagctcacatgtggtctctttggccagaagagccgcatcctcatgcggtggagctcgtctgaactgctttgatgatgcacatcccatacagaccagctcacaagcccagccttttgcttacagttaacaatcgtctttcgcagaaaggggatgaagaaaacacacctaggacaaaagggcaaagacattggtatcaacacaaaaccaatctattataaaattttacctggaaaagggaaatccagcgagagcgaaatagccaagaaacaacagggaaacaccaagacacaaagctcacctgggaaacaacaaacaaaggatacttatgctttgttcgatccacactggtaaagatctgcaggtggctcgggagaaagattgacggggaaggaggagctagaagaaggacaaccatatatttatgcgcgtgcgcagcaacagccaacagggacctacgtcacaagtgcggcacagaatattgtagtactgacaggtgacaacacacaggacaaggcgagagttgacttgcatgcagcaagatcatcacatccatgcattccctcttttcactatggaatacaggccatataagtagtactccatccgtttcaaatatttcaagttctactaggttcctactatatgtactccctccgtcccaaaatacttgtaatccatttcaccgacgagtatttccggacgaagagagtatttgaggttgtggatattagtagtaattctttttataaacttggtcaaacttaaaaaagttttggaacggaggaactatgtgttaaggcatcaaccatgagggcaagaattgctagtaccactgtcgaaccacaaaagagagagagagagagaga

>TaCBL9_3AL_4339532

tttattttactttgcatctttatcataaaaataccaaaaatattatcttatcatatctattagatctcactctcataagtggccgtgtagggattgataaccccttatcgcgttggttgcgaggatttatttgtttgtgtaggtacgagggactcgtgcgtggcctcctactggattgataccttggttctcaaaaaccgagggaaatacttacgctactctgctgcatcaccctttcctcttcagggaaaaaccaacgcaagtgctcaagaggtagcaggggccataaggcatcagggcgcgcccagggggtgaccaagccctgttgccttatgggcaactggtgcccccccttggtgtttctttgctctagtattttttatatattcaataaaagttcatcgtgaatttttaggtcatttggagctctgtagatagttatctctattatagctcttttaggttcagaactccagctgccagtaatctccctctccattcaatgcttgcaaatcaagagagaaaaggcatgagaattgcatcataaagtgaaatgacccaaaaataatataaatatcagtaggaaaacatgatgcaaaatggatgtatcagccatcgcttgcggcggatctcgcgggtgctacgcatggactagtacaacgtatgttgctcaactaggaatctgggatcctctccgaccatggccacgatggcacagtcgttcgcctactccccggtgatttgtccctccacgagtcggtgctcatcgaggagccgaaggttgtatctttagtcctcctgtgcttgtcggaacatcgacatcggcgagcctgctgcttttctgccaatacgacgttgaagtgcgctcatgcgtgctccaatgatatgccgacatggaccgacgagggcggtggtgtcggcttgtcgtcgtatgcctcgtccatcctctcgttggcgtcgctgcccgccggcgagctcgccggcaagcttgcctctattcgactggcacgacaagcctgctagttggccagctcttgcttttgtgcggtgaagaggctgtcccataacgctttggagctcgaggtcatggtagacgtggagcaactaggagatcaggagcggaggtggggtgtggtgcggtgcggaccgtgtcggcttggccatctataaatagcgggcgctggccaggccaactgataggatgtgtgaatgcggacgccagagtgggtttctccgcagttgtgctttctttaatgccgtcaggcggacagacgggcaacctatttgaatgcggggagagccgtccggtcacgccgctctagcattgaacaagtgcggagaccggacgtggcgcgagcagcattctcggtcgacgcgtgcgagagggtttcgtttgggaccgaggtgttgacgcgcgcgtgccaacggtccggactcctgtaaagcctcatccccatagatttgccttcggtttacgagaaaacagagttgagaccgatctgtgaagcaatgacaaaatgcgtctggaccgcttgatcaggacggatacaggcggtctgagagtccgctttgaagatgcccttaggcttaaaaaaagattaagaaagctgctttacttgcggtatccttgtgtcacgcacgccgtcatgtttttggccgtcctacatgtgttaagaaaaaggatattcccaaatcgacaaaagcagataaagcacgcaaagaagagaagaaaagaaactgctaaaagggaagagagaaaagaagaaagaaaaactaaaagcgggttgtggtttgtgcgtccgagcatgacacaaactgaaggaaatgatcattacggcaaggaaatctcagctgaaagcgagccggcgtcacactgccactgaactctacctagtactcctacaagcacaatgcccgagccctcaaaacgacacggccggtcacgcgggcgagccgttaaacgagcaaggggagggagcggtggtggggtacgccgccatggactcgcgcttctcctccggctccggggacgtggtgcgttgcgcttcccgccttcttcttctgatttggacggacgaaatccgaatgtcttttatgagctgcgatttcccgtgtttgtctgcagaggtcggggagctcgctgacggtgggggagcggctctgcgccgccttcttgcccttcgttgccgtcgccgaggccgtctttttcgtcctcgccgactgcctcgccgacatctgcccttcctcgtcctcctcctcgcgcctgcgcagagaatcctctgcttcgtccttcctcacggccaagaagaagagccatcacctgttccgccgccgcgtcgggccaggctgcacctccctcgacttccgcaaccttgcccgcctcgccgaggagtcccgttgctgtacttaaatccctcttccccccttccgggttcttagttcttacctgccgttctttgcaatgtcgtttgatccagttctccctccctctctctcttttccttttgaaattgaacgaagtctcggtgaacgaggtggaggcgctgttcgagctctacaagaagatcagctgctccatcatcgacgacgggctcatccacaaggtacctccaaaaaccgcttcagttcttacatctggcgcttcttccgatcagtaaattagtgatgaattgcttgcttctcgctcttcttttcagtgcagggagcagtttatatgtttttaaaacctcaagccgtgttctatcccgtggtcgtgggtaactgggttctgcctgcaggctgcagcctgaaaggaataggttttactggccgcatgtatagtgacactttgatagatatacatgatcgtttaggagtatctggttatacgagagcgatgcgcaaccaaattttttttttttggacaccagtgtgatgtgcaacttagcatctgatcacgaagtgttgggccgactgctaacgtatccagacatgaccagtgcacatgggagtattataaaattggtttgacagcacatagaatttttccaggttagcaagttgtgtggcttccatgtgcacaatcaaccttaaagcctgcatctctgtatgtgtggttgtcatgttccttcttgtgtcatcccaagtgatccaacataatggtgattggtcaagtattgagtgcatataagttttttatcaagctaacactatcgtaattttgcatttacttgactggtgtgcacatataacgtcttgggataaaaaatactagtggcttaagaacatcgtgagtaataaacaaagtttgaatgtcctatcttctcgaatactagtgtgattggaactgtcgattttttcctcttatctgtttcatctgttgaaatgttgtttaggaagaactgcagctagcattattcaagactcctagcgggcagaatctttttattgacagggtgagtgtttgaatcatttttctatctttaaatgctgtgaagttcattatttacacaaagttctgaattcttgttcttgttattatggaattattcacatcacatgttgctagaaataaaacacacggctaaagttgttctctgtaatccaggtttttgatttgtttgatgaaaagaagaatggagtaatagagtttgatgagtttattcatgctctcagtgtcttccatccatgttcacctgtggaggataagattaattgtaagtacggttcctgctgaaatcacctgcccatattaatgtgatgagatatttaaaggggccgctctctttctgttttctttttcagttgcatttaagctgtatgatttgaggcaaaccggatttattgagcgtgaagaggttagttcattatcatgttctatttttatatctattatggatgtaattctccttttaagctgatcctcggcaagatgatccattggcatgcctgtttctttaggttatgcaaatggttattgccattttgaatgaatctgatatgaaattgtctgacgagcttcttgaggccatcatagataaggtaaacaataacttgcttggccatctctggtaaattttcttaatcatcttgtgtggatgcatggttttcagtttcagtttcagaaaaatttcagtagatgtaagtgttacttgacgtttcagacgtttgaagacgctgatactgatagggatgggaaaatcagccaagaagaatggaaggaatttgtattgcgccaccctaatctgttaaagaacatgaccctaccctatcttaggtttgagtttgaaagctacaaccaattaactgcaattgttgttttgtaaattacagccagcatggtttatagctatccagagatttcttgtcattgttcttcaaagaatagatttgtcgttgtttactttactgatttcttctccatgtcatataattcagggacatcacaacatcttttcctagttttgtcttcaacacggcagttgaagactaaatagtgcaagattcctacatgacggcgacaagttggcagcatattatgtctgtcagctctcttgttgaatatcgggtttcctgagaaaaagaacactcataaccacctactgaagaaaattaccatcggtgactgttgattgtgtctgtcatcagagaactgaatcggcgccgaaccaacgaccacgacggcaacttagaaatgaagcaacggtcatgagctcaaactggtattttcaaagctgtacatacatcactccttaagctctgaaaagctcaggagcaactgtacatatatgctcgagcttgtacaagggaatccagacaaggctccggaagtgccagccagttctggaatcagcagttagtttcttcaagtgccttcccgcctaacagactatcttatgaattctatacatttgaatcgacctgctccttgaggaacatgtcatgcattagctttggttgttgttgtttttttcttcttctttttgggtcagaaaccttccggagacgcatgtttttgcttggcggtagcagcaaaatactgctgaacaatctacctgatgttgtttcatggaccaatcatccagtccgtggtagggacatatatatagtactccctccgtcctaaaataaatgtcgtgggtttagtttaaatttggataaatttgaactaaaccacgacatttattttgggacggaggcagtagcaagtttttttttggaaatggaggtatatccccggcctctgcatcatgatgatgcatgcagcccttttattaaaaattcagaaagtatcatcataaaggttttacagctcgcaaacggagcaaagtcatagcataaagatctgaaaaaataaagggcctaaaaaaataaaacaaccgatacggtgataatagggacaagctctctagccctctatcctgttatgcgaccgccatccgaaccggttgaatatagcccgagttaccatctcctattggttgcacccagtaaccaaaggctccaagtccataggaggcagtagcaagtagtcctgttttataagatttcaagcctttggtgttccttggattatgcaaggacagttcttcatctaaaaaatgtcttcgactggtataagattgcacatcatatcaagtgtttcacatcatatttttctagtgctaatcgttgaagtgaagtaaagtaagtttcttcgactgggataacagattgcacatcatatcgagtgcttcactccctgcagaaagagaactcacaccaatcaccaaatgccagcattatggtgaaaagaatcatccctggtgggctgcacgtacaaccaaccttctaaccatccaattaaaatttgattcccatttataaaaggatcacaggttgattattcattatgcattatttctattatagtgtcaaaacaaacttgatcaacccagaatagtcgcatcatatactagaaactagaaacaaggaaatctgggggatacaacacgcgtaggtggatggaacctgatagatattatgatgagctgtcctcttggcttctagcggcactgttgaagttcactttcatgaagttttttgacaaaggaataataataataatatcaaaaagatattatggcctctacaccaaaatgactcaagagctacccaaaacatcgaggatgcacatagctaaattattacaaaacaaagaaaaaaaagatcaacacgacagcggcaacaaacaacatcaacaaccattaatgatggcaacccaaggtaccaagatagatgcttcaacaacggcgcctccataaatgaaacaaggcacggaccctgtcgtcgctggatccgatcatcgaggccagaccattggttttcacctcggagaagagtctagtaaactccaggcaataccttcaacaagggagcgcctcgtgaacgacaggttgaaccaatgaaagtcggacctgatagatcatgggttttcagccctggaactcgtgcccatgcactagaaaagcatctcctttgttttgaagcaaaacccatagaacaatcggttgtacggtgtacattattaataaaagaatagaagttcaaatatgtacatagaaaagcacctcctagatgatgtcaccttgttctgccaccacttgtcgatccaatatcgcacagtcattcagacgcacatttctggtagacctagcgacacaattagaaccatgcccacctaacacatgttgttgagcagtgtcgtaggtacactcgccatctccccgattccatgccatcaagagtccagactgcccacataaccgattttggtcaatcacatggggcaatgtgacgtccccgatttgaccgtacactaatcatacatgcaaacgtgtacgatcaagatcagggactcacgggaagatatcacaaca

>TaCBL9_3AS_3303239

gtttgaggagtttattcatgctctcagcgtcttccatccattggcacctgtggaagacaagatcaactgtaagtacgattcttgcaaaaactgtgtgctcgacatgaacatgttgcgatatacttacggttagggctccccttctgagttgtttcagttgcatttaggctctatgatttgagacagactggctttattgagcgcgaggaggttagttcagatggatgttgtatttctatctgtatcatgaacgtagtttcttcacctcagcaaggtgatctgttggtgcttctgttttttaggttatgcaaatggttatcgccattttgatggaatctgatgtggaattatcggatgaacttcttgaggctatcctagacaaggtacgcaatcatatccgtgggattctttggttacttatttacattttacataaacgtaagtttcaattggtttcagacatttgaagatgctgacaccgatagggatgggaaaatttgccaagaagaatggaaggaatttgtattacgccatcctaatctactgaagaacatgaccctaccatatctaaggttcgattctgaaagctgcagtcaaataattgtaaatattgtctgcaacttccaccaaatatgtttagaactagtcagaaatactttggcatttattttcttctacaaaatttcgtcagtgtttacttgagtgattttttgtccatttctatatcagagacgtcacaacagcgttcccaagttttgttttcaataccgcggttgaagactaagcatcacaagacttgtaattgatggtgacaagttggctgttgagtgctgacagtgtcagcagttgaaaattgggacgctgagaaacagagcagccatgttcgcttactgaagaatcgccattggtgacttttgctgctttaggatggctatctgataaccatttcggcaactgaagagtcccattcatgatggcaaatttggcaactaacagcttctggtatgcctgtcgctgtcccgcattgtgttgtaaatgtatatacacaattccttcagctctcagaagcacaggacagctctaggtacatatagatatgaggaatttaagttcctcaaatgccagccagtttcgggatcagtcaggtgcaatttctagaatgctaacttctgatgttgtagcgtccgttatagactatcttagtgctaacatgtcatggcttagccatgattgctgtatatataatcgaaaacctttccgagacgcatgcttttgctggtgatagctacaagattgatgccaagcatatccgccaagtgtttgttcatgaaccaatcatcccatctgtgatgcggacagttcacttgttcttttgtgatggtgttcgttgggtggtgcaagtgacagttgtttatgtacttcttaatgctaatcatagatgtgaaccaaataaagtaattgcgatcatagatttggacaaaacgaactaaatttctttcgatggggataaggctgcacagtttgtcgatagattcgccagcgtggacgatggaccccttccagaaagcaaagtcacttgcaaataaagaagcaaagtcaccgaaaatacaaagatgaacatgggtgcgcgcgcacccatgtgaatag

>TaCBL9_3B_10423444

tatatatttttaatattagaaataattgaagagaaaaataataaaatcaaattgatccttcttcaaatttgaaataaattcaaatatgaattttgtgaaacctctaactttctccttgggtccttgagttgtttaagatttctaggatcatagcaaaatgcataaataaatatgatacgcatatgatgatctatgtatgacatccaaattgaaaattgggatgttacactatcgatgtaataaagagatgtgtgttgtttctattatttcatcgagtattgtgtatgctagcaagtcgatccagggactagcacggtaagcacagagacttcaactcttaccaggtcgggtcgctacagttgcacgcgcatcagttattttactcccagcggcacgacttgaggctgctaacagtctctactgggggctagacaatcgacatatacactttaacatcccttcccacacgtgacgtgggaagtcaacacgtgaatagactcagaggtatggctcaagaggcctatacgtggatacagagggcgcaacaacaatttttggataaattgtgaaagtcgggacttgaactcaaaaacttatgccttgataccatgttaaacttcatgcactggccaacacaaccaaaaggctgaaatgatgcaaaaggctaggcaattcacgtatacacttcaacaaggtcgaccgcttttatattggaggttccaacgggacactatgggtgattggatgtctcccttctcagtgcaaaggtttggagtttttttgaaaaaggggataccccggcctctgcatcagtacgatgcatacggccctcttattaacaaaataaaatagtgccaacatggttccaaaggtcctcaaaagtgaaaaaaaaagcagaacaagctcacacagagcccgaagaagctagatacatcaactagccatatgaagatgccacaaccggctgggtaaactaggtaggaatactaaatgcctatcatattacatgaccgccatccaaaccggttgaagatatcccgagctaccatctcccaacggatagacccagtaaccaaatgctccctggcctccgtctgggtgagtagcgaccacgaacggatcagagccgtagtccggaataaaacctgcaaaaaatgtatacatgatattctgttaaagaccagatcatttctgcagttccagatagtccacagcaaagcacaaaccccaacccgaatatgtctcgctaagttaggctcaatcccattaagccatgtcccaaataaagcattaacagaattcggtggtttgatgttgaaagcaatgtcccaaataaagcaaaggtttggagtgttccacatggtccaagattgtgtctgtcacaaaagattaggcgacttaactttgcctgatgtgtgggttatgccttgtccaagttgccacatgtgattgaccaaaattggttacgtgggcggtctggactctcgatggcatgaatcagcgaagatggcgagtgtacctacgacactgctcaacaacatgtgttacgtgggcatggttctaattgtatcgccaagtctatcagaaatgtgcgtctgaacaactatgcgatgatggatggacaagtggtggcagaacaaggtgacatcttctaggaggtgcttttctatatacatatttgaacttctattcttttattaatatacatcgtagaaccgattgttctatgggttttgcttcaaaataaaggagttgcttttctagtgcatgggcacgagttccagggctgaaaacccatgatctatcatgtctgactttcattggttcaacctggcatgtcgttcacgaggcgcttccttgttgaaggcattgcatggagtttactagactcttctccgaggtgaaaaccagtgatctagccttgatggttggatccagcgccgacagggtccgtgccttgttttattatggaggcgccgttgttggagcatctatcttggtaccttgggttgccatcattaatggttgttgatgttgtttgctgcggctgttgtgttgacctttttttttctttgttttgtactccctttgtccgaaaatacttgtcatcaaaatggataaaaagagatgtatctaaaactaaaatacgtctagatgcatccccttttatccattttgatgacaaatatttccggacggagggagtaataatttagctatgtgcatcctcgatgttttgggtagctcttgagtcgttttggtgtagaggccataatatccttttgatattaatattattcctttgtcaaaaaacttcgtgaaagtaaacttcaacactgcgctagaagccaagacgacagctcatcataatatctatcaggttccatccacctacgcctgttgtacccccagatttccttgtttctagtttctagtatatgatgtgactattctgggttgatcaagtttgttttgacactataatagaaataatgcataatgaataatcaacatgtgatccttttttaaatgggaatcaaattgtgattggatgattagaaggttggttgtacgtgcagcccaccagggaccattcttttcacgataatgctggcatttggtgattggtgtgagttcgctttgtgcaggagtgaagcactcgatatgatgtgcaatctcttatcccagtcgaagaaacttactttacttcacgtcaatgattagcactggaaaaatatgatgtgaaacacttgatatgatgtgcaatcttatcccagtcgaagacattttttagataaagaactgtccttgcataatccaaggaacaccaaaggcttgaaatcttataaaacaggtctacttactactgcctccgtcccaaaataaggtttagttcaaatttatccaaatttgaactaaacccacgacatttattttgggacagagggagtactatataaatgtccctaccacggactggatgattggtccatgaaacaacattaggtagattgttcagcagtattttgctgctaccgccaaaaaaagacgaaaaaaacaaccaccaaggctaatgcatgacatgttcctcaaggagcaggtcgattcaaatgtatagaattcataatatagtctgttaggtgggaaggcacttgaagaaactaactgctgattccagaactggctggcacttccggagccttgtctgaattcccttgtacaagctcgagcatatatgtacagttgctcctgagcttctcagagctaaaggagtgatgtatgtacagctctgaaaataccagtttgagctcatgaccgttgcttcatttctaagttgccgtcgtggtccttggttcgggacgattcagttctctgatgacagacacaatcaacagtcacaccgatggcatcatggcaattttcttcagtaggtggctagtttctcaggaaacccgatattcaacaagagagctgtcagacataatatgctgctaacttgtcgccgtcatgtaggaatcttgcactatttagtcttcaactgccgtgttgaagacaaaacttggaaaagatgttgtgatgtccctgaattatatgacatggagaagaaatcagtaaagtaaacaacgacaaatctattctttgaagaacaatgacaagaaatctctggatagctataaaacatgctggctgtaatttacaaaacaacaattgcagttaattggctgtagctttcaaactcaaaccttagataaggtagggtcatgttctttaatagattagggtggcgcaatacaaattccttccattcttcttggctgattttcccatccctatctgtatcagcgtcttcaaacgtctgaaacgtcaagtcaacacttacatctaccgaaatttttctgaaactgaaagtgaaaaccatgcatccacacaagatgattatgaaaatttaccaaaggtgcccaagcaagttattgtttaccttatctatgatggcctcaagaagctcatcagacaatttcatatcagattcattcaaaatggcaataaccatttgcataacctaaaaaaacgggcatgccaatggatcatcttgccgaggatcagcttaaaaggagaattacatccataatagatataaaaataggacatgataatgaactaacctcttcacgctcaataaacccggtttgcctcaaatcatacagcttaaatgcaactgaaaaagaaaacagaaagagagcggcccccgtaaatatctcatcacattaatatggacaggtgaattcagcaggaaccgtacttacaattaatcttatcctccacagctgagcatggatggaagacactgagagtatgaataaactcatcaaactctattactccattcttcttttcatcaaacaaatcaaaaacctggattactgagaacaactttagctgtgtgtgttatttctagcaacatgtgatgtgaataattccataataataataacaagaattcagaactttgtgtaaataatgaacttcaaagcatttaaagatagaaaaatgatgcaaacactcaccctgtcaataaaaagattctgcccgctaggagtcttgaataatgctagctgcagttcttcctaaacaatatttcaacagatgaaacagataagaggaaaaaaatcgacagttccaatcatactagtatttgagaagataggacattcaaacttcgtttattactcacgatgttcttaagccgctagtatttttgatcccaagacgttatatgtgcacggataactgacttgcacaccagtcaagtaaatgcaaaatcacgatagtgttagcttgataaaaaacttgtatgcactccatacttgaccaatcaccattatgttggatcagttgggatgacacaagaaggaacatgacaaccacacatacagagatgcaggctttaaggttgattgtgcacatggaagccacacaacttgctaacctggaaaaaatgtatgtgctgtcaaaccaattttataatactcgtatgataagttggcccaacacttggtgatcagatgataagttctatgtgcactggtcatgtctggatacgttagcagtcggcccaacacttcgtgatcagatgataagttgcacatcacactggtgtccagaaaaaaaaaatggttgcgcatcgcactcgtataaccagatactcctaaacgatcatctatatctatcaaagtgtcactatccatgcggccaataaaacctattcctttcaggatgaagcctgcaggcagaacccagttacccacgggatagaacacggcttgaggttttaaaaacatataaactgccccctatactgaaaagaagagcgagaagcaagcaattcatcactaatttactgatcggaagaagcgccagatgtaagaagtgaagcggttttcggagggaggtaccttgtggatgagcccgtcgtcgatgatggagcagctgatcttcttgtagagctcgaacagcgcctccacctcgttcaccgaaacttcgttcaatttcaaaaggaaaagatgtctccctaaactctagcttgatcgtgaaagaaagagagaattcagagaggaagaacacgcagagacactgaagttttttgtgctgcgctcaacacctgcagcttttctgttttttgctcttcttattcatcaaaagtacaccaacgtgcccgagcaacgtgctccactactgaccacgtccatccccatgatccgagcttcgtgctgggcctactacgactgagccaaggcgcacaaaacgcaaccaattggcgaggaaaaaatgctaacagaaaaaacgactagctactgtcgaaactgaatgtgtctagcgacacttatctgatgagaacagactaagttcagagaatgcagagggagacagggtttaagccaacaatctccccctcaaccctggcgaacgagcttgacgtcgacgatcccagtcttgccaagcagctcctggaagcgaacacggctcaacggcttcgtcagtatgtctgctttctaatccctcgtaccgatgaactccacggccacccatctcttctcaacataatcacgaatgaaatgatatcgaaggtcgatgtgcttactgcgatcatggagaactgggttcttgcagagggagatcgcagatttgttgtcaatgaacagagagaagggcgccgctggttgattcatcagctcgccgaagagacgtgcaagccaaatgccttggcaagctgccgtcgccgccgcgatgtactccgcctcgcaagatgagattgcgacgaccttttgcttctgagattgccagctgacggggctttcaccaagaaagaacagcacgccagaagtgctcttcctggtgtccacgtcgccggcgtgatcggagtcgctgaagcctgtcagggactcgccgtcgccgtagcggtacatgcagccgtagctcagcgtgcccgctatgtatcgaaggaggtgcttcactgctgcatagtggtctgctgctggcgcctccatgaacctgctcaggaaacctacggcgaacgaaatgtccgggcgagtgtgtacgaggtacctcaggctgccgatcacgctgcggtagagcgttgcatccaccgcagcttccttgctttcctttctcaacgggcaccgtggctccataggagtgcgagtcggatagcaccctgtcagcccagttttgtcgagcagcttggaggcatacgctgcctgtgtgatcgtggtgcgtccacgctcctgcttcacttccaagccgaggtagtagcgaagcagtcccaagtcgctcatggagaagagttgtttcatctcctctttgaacccgaggacctcctgttcctttgcgccggtgatcaccagatcgtcgacgtacaccccaacgatgagcaggctctcgccagttccgcgcgcgtacatgccgtactctgcgacgctgcgcgtgaacccgagagatgtgagactagcatccagcttgtcgttccacgctcttggagcttgacacagaccatatagagctttgtgcagcctgtacacagcctgctcacgccccctccgcgacgaatccagatggctgggacacatagaccacttccttgaggtcgccgttcaagaacgccgatttgacgtccatatggtgcacttgccagccacggtgcgccgccggcgtcaggatgagccgcacggactccagtctagtcaccggcgcgaacacctcctcgaagtccacgccaaggcgctgcacgtggcctttagcaacgaggcgggctttgtgcttgatgacgttcccttcctcatccttcttcagcttgtacacccatttgagaccgatcgcttgatgacctgcagggagcgtggtgagcgtccaggtgttgttgtcgtcgatgaactgcagctcgtcgagcatcgcccggcgccaacagtcctcgtgctcggcttcggcgaatgatgtcggttcctcccccgtcacaaggtgcagctcctcatcatcaccactgtcctccgatggcgggggctctcagcacctcctggtacttcagaggttgagccgagcagctccttgaccaggcgatatcgatgtggtgcagagaccttgtccgcgttgtcgaacaagtccggctgtaccgccgatggtgacacgaagggcaccggcgtagtctctggcgtgactgcatctgctcctcccccatgcgtgggcggcgacgtg

>TaCBL9_3B_10535092

gttgctatgcatcaccatgatcttgcgtgtgcgtaggaatttttttgaaattactacgttccccaacattcgcccactactgctcctttcgctcgttcgcccgtgccgtcgatgtctttcgctgcagttttcccagtttgatttttcttctccctaatctttcatagtactttgaaaacaatggaaaaagtttatgaaatagaaaaacaatcatggatttgaaaaagtttgaaagggtagactatagaccggctcgccattgaccttgatctctaaccagatctgttgcccgttgacattttagaaacaattcactggtttgagaaaaagttcacaaatttttaaaaatttcgtaagtttgaaggaacttcatgagtttgaagaaaagaaaattttgaaaaaatctcatgccttggaaaagttaatgaaatcacaaagaagttcacaaattttaaaagaatgcaaaaatgtaaaagaagttcaaaaattaaaaaaatcacaattatttataaaaagttaatgaaattttggaatagtccttgaatttgagaaaagttcacaaaattttgggaaaatatatacaaatttttcaaaaggtgacgaatttcctctcccttttttctttgtccttttcgacgcgcaggtcagagaacagatgattgttgcatccattctcaggttatccacttccatgatctagctaagagctaactaaccatatgcttccaaaccttggcggtccatcaagaaagggacatcaaaaaatatggcagatactaccttccccgcaaaaaggaaaaaaaaaggacgatagatgctgcctcacctaggcccgccctgttttgttcttatcaacgaaaaggttttttttttcgtgcatgctgacacaagcagcattgcgtggtaggtagttttgttttgttttcgcccggtatggcctcgtgtagttcatgttccgcgcatttgtatgatcccaaaatgtttgatctgatgccatgaatctttccaaaagatttgagtgagtttgttttttcttgtatgaaatgtagtgtcggacaataggaagacgataattctaaaataaacaatcaacgtagaaaaaataacaaactactttagcgatctatataaacgatcttataagagttatagagggagtactcatgtaatgttctcctcactttcaatagttttgccacataatctggcgtgccgacttgtaaaaatgccaagcatattggtgattggtgtgtgacatggctttctgggaagcggtccacgttggtgaatccatgtgtgtgcagcgtttatcccccaatcgaaaggaaattactttattcagttcattcacatcaatgattgagttactttgattagttcacatctacgtatgattagcatcaggaaagcacataaagaactggcccttgcatcaccccaagggccaaaacaaagtccactgtcgccatcagcccatcacaggtgagatgattggttcatggacaaacacttggcgaatatgctttgcatcactcttgtacagtagctatcaccaagcaaaagcatgcgtctcggaaaggttttcgattatatatacatcaatcatggcttagccatgacatatgttggcgctaggattaggatagtctagataatggacgctacaacatcacaagttagcattcacaaaattgcgcggaactggctggcatttgcggaacttagattcctcctatctatgtatatgagcaacttagactcctcatatcacgctgtcctgttcttgaatgaatggtgtatatacagttagtacaacacaatgcacaccagacgccgttagctgccaatttgccatcatgaatgggactcttcagttgccgggttgattatctgatgtccatcctgaagcaacagccatccttaacatggctgctctctttctcagcatcccaattctcaacggctgacgcaatcagcaaccaacttgtcaccatcaagtacaagccttgtgatgtttagtcttcaaccgcggtattgaaaacaaaacttgggaacgctgttgtgacgtccctgatacacagattggcaagaaatcactcatgtaaacaccgacgaaatgttttcaaagagaagaaatgccaatgtatttctgactagctctaaacatatggaatgtaagttgcagacggtattgacaattatctgattgtagttttcagattttcaaaccttagatagggtagagtcatgttcttcaatagtttaggatggcgtaatacaaattccttccactcttcttggcaaatttttccatccctatcggtatcagcatcctcaaatgtctgaaaccaattcaaacttacatccatgtaaaatggaaataaataacgaaagaagcccaaggaaatgattgcgtaccttgtctaggatggcctcaagaaggtcatgcgataattccacatgagattcattcaaaatggcaataaccatttgcatcacctaagaaacaggagcaccaacagatcaccctgctgaagtaaaggaactacaggtacagacagaaatacaacatccatctgaactaacctcctcgcgctcaataaagccagtctgtctcaaatcatagagcctaaatgcaactgaaaaaactcagaaggagagccctaactgtaagtatatctcaacatgttcatgcagaagtcatagtttttgcaagaatagtacttacagttgatcttgtcttccacaggtgccaatggatgaaagacactgagagcatgaataaactcctcaaactctattactccatttttcttctcatcaaacaaatcaaaaacctgaatcatataggacaatctcagttggatgctatctcacaaaccaaaatgcaatgtgagcaattcagaattccgtgtatgacatggtaaataatgatcttgaagcacttaaaaatagcaaattgaagttgtcaacgcttaccctatcaagaaaaagattctgcccactaggagtcttgaataatgctagctgaagttcttcctaaataacaattcaacagaaaagacagataggtcatttttttatgctttgctagttcaatcatagatagtatacgaatataaaagggcattgaaacttttctaggtacccgctttcatccccgcacgtcatatgtgcacccataacagatggttcatatgtgcattataaaactaattactgttcctttgctggttcaaacttcacacggaatccacacaacgtgctgacctggaaatctaattgacggttcaatcatagtatgcgaaaagaatagaagaggacaatttcatccccgcacgtcatatgtgcacccataacagatcgttcatatgtgcactataaacttaattaatattctttcacagacttggacttgcacatcaatcatcaacaaagtaagatgcaaaattattgcaccattaggttaataaataacttagtcacgccacaagaatgaaggtgacaaatcaaacattcagacatgtaagcgccacacaacacaacgtactgacctggaaaaaatacaggccaataattccgtgggtcaagccaaatttattattgctccctctatacagctcatatgacagtgtctccttaatccatgtgctggggaaagaatccatcacatgtgatgcagcattttcagatggatctatgaagtaatccggatctgaatcgctaagcccaaacattgccggtcatgtatctctgtcgaattaggactgtgcactaatttgtttcagattacagaagaaacagaggcgctttagctttagtgggtaccttgtggatgaggccgtcgttgatgatggagcagctgatcttcttgtagagctcgaacagcgcctccacctcgttcaccgagacttgattcagtcggcaaggaaaagaagactcgtatcaaacgacattgcaataagaagtactaagatgaaatcgagggggaaaagagatggatggttttctatacagcatctggactcgtcggcgaggcgggcgaggtcgcggaagctgagggaggagcagcccggcgcgacgcggcggcggaggaggtggtggtggtggtggctccccttcttggccgtgaggaagaaggacggggcggagggatctctgcgcgggtgcgaggaggaggaggccgagggaggggagcagatgccggccaggcagtcggtgagcgcgaagaagacgaactcggcgatggcgacgaagggcaggaccaccgcgcagagccgctcccccaccgtcagcgagctccccgacctctgcgcgcggcgaccagaatcaaacaagtcagaattgcagatttggatttcgtccaaggaagacagaggaagaaggccagagctagccagctgggtgagccacccaccaagtcgccggaggagatgcgggagttgaagcgtgaggccatggcggcgctcctcccctccctccctgnnnnnnnctgcctccacttctcttctaacgactcgcctgcgtggtcgacggcggaccgtgtctttttggggcgcgcacacgctattcagaagggaaaagaaatggactgctcctctgcttcgagaaagcggccctgttccgttctttctttaagtgaaaaaaaagagagaatgagtcagatgaagatgatctgccttgtccacgtaacgtaggtggcattcatcgacggtcccccgtggcgacaacagatccgatccgccatgatttgcatcagggatggagtactccctccgtaaactaatataagagcgtttagaatactacaataatgatctaaacgttcttatattagtttgcagaaggagtacatcagatcatctctagccagccagatcctgtatcgcaaattcccattagggcttgtttggttccaataaattacctgatttataagttaggtgacttaaaaccagtgatttataagtcacgcctgtttggttgtcacctgacttataagtcacctgacacaccttttcacatcaatccatatcatgcaggttatgggatccacgcaaaaggaggcgatttataagttttaagttggggtggagcaacttatgacttataagttgaggtgacttataagtcaggtctgtttggcaaataagtcatttttttcacttttcgacttataagttggtgacttatttggaaccaaacagacccttaataccgcgaaacgcatggcagaacaaatttttcaaagaataatgacatagttctcaccaaaatttaaacatagttcatcgcacaaacttaaaatatactagatttaaacctacgctaaacgactcatccttcttgacatggagatgatactccaccgtggagtttcagaggacgaaggcgaagtggtactcctcttggccgcctcgagcggcgcggcggtgccgattcttcgtgctccgccttgcggaggcgcttcgtggttggccaaagaatgtagtggaggcagtcagactccctccacgccctccggccgccgctacgggagagggcgccgtgctcccgcacctgcatgcagcgccattgatacatccattgtgcatcatgttttcctactgatatttacaatattttttatcattataacactttataattcaattctaatgtctttcctctcttaatttgcaagatttacatgaagaggaagattgccggcagttgaaattttagacctggaaaatctacatcagagattgatggggaaccttgcatggaatttcaaaaaaaatctacgcacacgcaaggatctgcccatggagatgcataccaacgggagggagagtgtgtctacataccctggtagaccgtaagcggaagcgtttcacaatgcggttgacgtagtcgaacttcttcgtgcttcaactgatcaagtaccaaacgcacgacacctccgcgttctgcacacgttcagctcggtgacgttcctcgccttcttgatccagcaacacgtcgagctagtagatgagttccgtcagcacgacggcgtggtgacggtgatggtgaagtgatctccgcggggtttagcctaagcactacgaaaatatgacagggggtgtaaacgatggagggggacgccgcacattgctaacaattgatatgttttatgtgtggcgccccccttcatgtatatataggtgggagggagggaggagcagctctagggggcaccccaagtaggaggaatcctacttgagctccctcccaagcctgcgcctccccctgctatatataaccgacggggaaggaaagagagaga

>TaCBL9_3B_10578811

caaatacctgtgtcagcggaggatcaatcaaaaactacttttacttgccctttcggtagttttgcttatagatgtatgccttttggtttatgtaatgtacctgctacctttcaaagatgcatgatggcttatagatgtatttcaaagatgcaagatttgtgaggtattcatggatgatttctccatttatggatcctcttttgatgattgcttgagcaaccttgatcgagttttgcagagatgtgaagacactatcctcgtcttgaattgggaaaagtgccactttatggttaatgaaggcattgtcttggggcataaattttttgaaaaagttattgaagttgataaagctaaagttgatgctattgaaaaaatgccatgtcccaaggacattaaaggtataagaagtttccttggtcatgccggtttttataggaggttcattaaggacttttctgaaatgtctaggccttagactaatctattgcaaaaagatattcctcacaagagggttatttcattcaaatcatgcaaattagagtccaaaacaagaggaacaactgttaggaaaagtagatacgacggagacgtatcaggaagcaatatgaacctcagcgcccacaactcctttgtgttctacttgtgcatatcatctacgcatagacctggctctgataccactgttgggtaacgttgcatggaaaacaaaaaaatatctatgcacacgcaagatctatccatggagatgcatagctacgagagggcgagagcatctacatacccttgtagattgataagcagaagtgcttatcaatgcggttgatgtagtcgtacaccttcatgatccgtcccgatcaagtacgaatgtacgacacctccgcgttcagcttgatgacgtcctcgccttctcgatccagcaagagaggcaaagtagtagatgagttccggcagtatgacgacgtggtgacagtggtgatgaaactatccgcgggcttcgccaagctcaacggacgtggacagaggaggaactagaactagagggagacgggcgtcgcacacggctttgggattcgtggtgtgtgttgcccctctccctcctcacttatatataggtggggggaggggaggcagccctagggcgccccacgggtgccggcggccagccctggcatcctaccctaggcgcctccctcctttccatatttgggcatggaggagaaggcaggagggggccggcggctgccctaccttggcgccccccctttccttcctggcacctaggcaagggaggtggggcgcgcacaaccccttagtgggctggtgcgcctccctcctttggacaatggcaccaagtgccggtggcctcccgaaaccccttccggcactccgataaaaccccagtgcattccgaaacctttctggagaccaaatagtatcgtcttatatatcaatctttaccgccagaccattacagagctcctcatcatgtccgtgatctcatccgggagtccgaacaacattcggtcaccaacatgcacaactcatataatactgtattgtcaacgaacgttaagcgtgcgaaccctacgggttcgagaatgatgtagacatgaccgagacgcttctttggtcaataaccaatagcaggtcctagatgcccgtattggttcctacatattctacgaagatctatattggtcgaaccttaatgacaacatacttagttccttttgtttgtcggtatgttaattgcccgagattcgaccgtcggtatctccatatctagttcaatctcgttaccagcaagtctctttactcgttccgtaatacatcatctcataactaactccttagtcattttgcttgcaagcttcttgtgattctgtattaccgagagggcccagagatacctttccgcaacaaagagtgacaaatcctaagctcgatccatgccaaattaacacacaccttcggagatacccgtagagcatctttatgatcacccaattatgttgtgacgtttgatagcacacaaggtattcctacggtatcagggagttgcatgatctcatggtcgaagtaatatgtatttgatattaagaaagcagtagcaataaactgaacaatcatatgctacgctaacggatgggtcttgtccatcatatcattctcctaatgatgtgatcttgttatcaattcacaacacatgtctatggttaggaaaccttaaccatcttttgatcaacgagctagtctagtagaggctcactagtgacatgggatttgtttagctattttttaaaaataatacatttttttcgtaatttctagaaataatacatagatttgagaattttcgcaaatgatacggcctcggtttcggccaagccgattagggtctgtcggtttgtaagaggccgattaggtcctgtcagcctaggaaatgtcgattggtgctatcggcttgggcgaagccgatatgttagcctgtgatgtgacacaagcgtatatggataggatagctccccttgagccaatggattggaaactttctagcttggctgcacgctagtactagtgctctcattccatcggtggcagatgcactagtagtgttctcgttccattagaatactacgtcggcgtcctacttaggttagcataattatcatacgctcatgtccatgcacggatacacgacaacagatgcacacgttgactggttctccgggctgccaattcccgacatgagtgtttgattataccaaatattcatagtataaatatggatcatcccagctcctgtctatgacgtgtgggtcccacgacatgcaattttcttatgaaataatgatattgggctctttttatagaattgtcatccacatcatgatggtgcaaacggattaaaatttgaattcaccgtttgagaaatattatcttcagaagttttgaatcatacatgtacagagctagcaagtttctaatcaattggctcgctgtcctatgcaagtatgcttgtgtgagaacatatcggcttcgcccaagctgatagctactaatcggcatttcataggccgacagaccctaatcagcctcttacaaccgacagggcctaatcggcttagcagcaaccgaggccgtattatttgtgaaaattctcaaatctacgtattatttctagaaattatggggaaatgtattatttaaaaatattagcatttgtttatgtatccgcaaatgtatttaagtttccaggcaatacaattctagcatgaataataaaactttatcatgattaaggaaatataataataaccactttattattgtctctagggcatatttccaacaagagggtcgttggtgacgtcgatagcttcgatttccccctcccagggggaagtacttccggtggaatagctccaccggagcaaaagtgctcctgcccaggttctgcctggagacgacggcgcttcgtccctaaagccttctcttattttgttctaggtagaaacccttcatgtagcaggagatgggcactggaggctagctgtggccccataaggcattagggcgcgcctaaggggtggccgggccctgttgtcttatgggcaaccggtggcccccctctggtgtttctttgctccactattttttatttattcaataaaaatcaccgtgaagtttcaagtcatttggagctttgtagaatagttatctttgttgtagctcttttaggttcagaactccagctgccggtaatctccctctccatgtaaagcttgcaaatcaagaaagaaaaggcattagaattgcatcataaagtgaaatgatgacgcaaaaaatatataaatatcattaggaaaacatgatgcaaaatggatgtatcaaccatcgcttgcggcggatctcgcgggcgctacacatggactagtacaacgtctgttgctcaacttggaatctgggatcctctgcgaccatggtcgcgatggcatcttcgttcgcctactcgccgatgatttgcccctacgtgagtcggtgctcatcgaggagccgaaggttgtatccttagtcctcttgtggttgtcggaacgccgacatcggcgagcctgctgctcttctaccaatacgacgtcaaagtgcgctcgagcgtgctccaatgatatgccgacatggaccgacgagggcggtggtgccggctttgtcgtcgtatgcctcgtccatcctctcgtcggcgtcgctgccctccggcgagctcgccggcaagctcgccggcaagcttgcctcttcgactggcacgacaagcctgctagttggccagctcctgcttctgttcgatggagaggctgtcccataacgctttggagctcgaggtcatggcagacgtggtgcaactaggagatctggagcggacgtgtggtatggtgtggtgcggatcatgttggcttggccatgtataaatagcgggcgccggccaggccaagtgacaggatgtgtgaatgcggacgccagagtgggtttccgacagttgcgctttctttaatgtcgtcaggcgcacggacgggcaacctatttgaatgcgtagagagccgtcctgtcacgccgctctggcattgaacaggtgcgaagaccggatgtggcgcgagcggcattctcggtctgcgtgtgcgagagggttttgcatgggaccgaggtgttgacgtgtgcgtgcgaacggtccggatgcccgtaaagccccatccccactgatttgcctccagtttacgagaaaacagagtcgggatcggtctgtggaccaatgacaaaatgggtccgaaccgcttgatcaggacggatacacacggtttaagagtttgctttggagatgcctttaggcttaggcttaaaaggaagtttaagaaagctgttttacttgcgatatccttgtgtcaggcacgccgtcatgtttttggccgtccgacatgttttaagaaaaatgatattctcaaatggacaaaagcagataaagcacgcaaagaagagaagaaaaaaaactgctaaaaaagaagagaagagaagaaaagaagaaagaaaaactaaagtggtttgtgcgtccgagcatgacacaaactgaagaaaatgatcattacggcaaggaaatctcagctgaaagcgagccggcgtcacactgccactgaactctcctacaagcacaatgcctgagcccccaaaacgacacggccggtcacgcgggcgagccgttaaacgagcaagtggaggaagcggccgagcgggtggtggcgtacgccgccatggactcgcgcttctcctccggctccggggacgtggtgcgttgcgcttcccgccttcttcttctgccttggacggacgaaatccgaatgtcttttatgagctgcgatttcccgtgtttgtctgcagaggtcggggagctcgctgacggtgggggagcggctctgcgccgccttcttgccattcgttgctgtcgccgaggccgtcttcttcgtcctcaccgactgcctcgccgacatctgccctccttcgtcctcctcctcgcgcctgcgcagagaatcctctgcttcgtccttcctcacggccaagaagaagagccaccacctgttccgccgccgcgtcgggccaggctgcatctccctcgacttccacaaccttgcccgcctcgccgaggagtcccgttgctgtacttaaatccctcttcccccttccgggttcttagctcttacctgccgttctttgcaatgtcatttgatccagttctccctccctctctctctcttttgttggcttaaaccctgtctccctctgcattctctgaacttagtctgttttcatcagataagtgtcgctagacacattcagtttcgacagtagctagtcgttttttctgttagcattttttcctcgccaattggttgcgttttgtgcgccttggctcagtcgtagtaggcccagcacgaagctcggatcatggggatggacgtggtcagtagtggagcacgttgctcgggcacgttggtgtacttttgatgaataagaagagcaaaaaacagaaaagctgcaggtgttgagcgcagcacaaaaaacttcagtgtctctgcgtgttcttcctctctgaattctctctttctttcacgatcaagctagagtttagggagacatcttttccttttgaaattgaacgaagtttcggtgaacgaggtggaggcgctgttcgagctctacaagaagatcagctgctccatcatcgacgacgggctcatccacaaggtacctccctccgaaaaccgcttcacttcttacatctggcgcttcttccgatcagtaaattagtgatgaattgcttgcttctcgctcttcttttcagtatagggggcagtttatatgtttttaaaacctcaagccgtgttctatcccgtgggtaactgggttctgcctgcaggcttcatcctgaaaggaataggttttattggccgcatggatagtgacactttgatagatatagatgatcgtttaggagtatctggttatacgagtgcgatgcgcaaccattttttttttctggacaccagtgtgatgtgcaacttatcatctgatcacgaagtgttgggccgactgctaacgtatccagacatgaccagtgcacatagaacttatcatctgatcaccaagtgttgggccaacttatcatacgagtattataaaattggtttgacagcacatacattttttccaggttagcaagttgtgtggcttccatgtgcacaatcaaccttaaagcctgcatctctgtatgtgtggttgtcatgttccttcttgtgtcatcccaactgatccaacataatggtgattggtcaagtatggagtgcatacaagttttttatcaagctaacactatcgtgattttgcatttacttgactggtgtgcaagtcagttatccgtgcacatataacgtcttgggatcaaaaatactagcggcttaagaacatcgtgagtaataaacgaagtttgaatgtcctatcttctcaaatactagtatgattggaactgtcgatttttttcctcttatctgtttcatctgttgaaatattgtttaggaagaactgcagctagcattattcaagactcctagcgggcagaatctttttattgacagggtgagtgtttgcatcatttttctatctttaaatgctttgaagttcattatttacacaaagttctgaattcttgttattattattatggaattattcacatcacatgttgctagaaataacacacacagctaaagttgttctcagtaatccaggtttttgatttgtttgatgaaaagaagaatggagtaatagagtttgatgagtttattcatactctcagtgtcttccatccatgctcagctgtggaggataagattaattgtaagtacggttcctgctgaattcacctgtccatattaatgtgatgagatatttacgggggccgctctctttctgttttctttttcagttgcatttaagctgtatgatttgaggcaaaccgggtttattgagcgtgaagaggttagttcattatcatgtcctatttttatatctattatggatgtaattctccttttaagctgatcctcggcaagatgatccattggcatgcccgtttttttaggttatgcaaatggttattgccattttgaatgaatctgatatgaaattgtctgatgagcttcttgaggccatcatagataaggtaaacaataacttgcttgggcacctttggtaaattttcataatcatcttgtgtggatgcatggttttcactttcagtttcagaaaaatttcggtagatgtaagtgttgacttgacgtttcagacgtttgaagacgctgatacagatagggatgggaaaatcagccaagaagaatggaaggaatttgtattgcgccaccctaatctattaaagaacatgaccctaccttatctaaggtttgagtttgaaagctacagccaattaactgcaattgttgttttgtaaattacagccagcatgttttatagctatccagagatttcttgtcattgttcttcaaagaatagatttgtcgttgtttactttactgatttcttctccatgtcatataattcagggacatcacaacatcttttccaagttttgtcttcaacacggcagttgaagactaaatagtgcaagattcctacatgacggcgacaagttagcagcatattatgtctgacagctctcttgttgaatatcgggtttcctgagaaactagccacctactgaagaaaattgccatgatgccatcggtgtgactgttgattgtgtctgtcatcagagaactgaatcgtcccgaaccaaggaccacgacggcaacttagaaatgaagcaacggtcatgagctcaaactggtattttcagagctgtacatacatcactcctttagctctgagaagctcaggagcaactgtacatatatgctcgagcttgtacaagggaattcagacaaggctccggaagtgccagccagttctggaatcagcagttagtttcttcaagtgccttcccacctaacagactatattatgaattctatacatttgaatcgacctgctccttgaggaacatgtcatgcattagccttggtggttgtttttttcgtctttttttggcggtagcagcaaaatactgctgaacaatctacctaatgttgtttcatggaccaatcatccagtccgtggtagggacatttatatagtactccctctgtcccaaaataaatgtcgtgggtttagttcaaatttggataaatttgaactaaaccttattttgggacggaggcagtagtaagtagacctgttttataagatttcaagcctttggtgttccttggattatgcaaggacagttctttatctaaaaaatgtcttcgactgggataagattgcacatcatatcaagtgtttcacatcatatttttccagtgctaatcattgacgtgaagtaaagtaagtttcttcgactgggataagagattgcacatcatatcgagtgcttcactcctgcacaaagcgaactcacaccaatcaccaaatgccagcattatcgtgaaaagaatggtccctggtgggctgcacgtacaaccaaccttctaatcatccaatcacaatttgattcccatttaaaaaaggatcacatgttgattattcattatgcattatttctattatagtgtcaaaacaaacttgatcaacccagaatagtcacatcatatactagaaactagaaacaaggaaatctgggggtacaacaggcgtaggtggatggaacctgatagatattatgatgagctgtcgtcttggcttctagcgcagtgttgaagtttactttcacgaagttttttgacaaaggaataatattaatatcaaaaggatattatggcctctacaccaaaacgactcaagagctacccaaaacatcgaggatgcacatagctaaattattactccctccgtccggaaatatttgtcatcaaaatggataaaaggggatgcatctagacgtattttagttttagatacatctctttttatccattttgatgacaagtattttcggacaaagggagtacaaaacaaagaaaaaaaaaggtcaacacaacagccgcagcaaacaacatcaacaaccattaatgatggcaacccaaggtaccaagatagatgctccaacaacggcgcctccataataaaacaaggcacggaccctgtcggcgctggatccaaccatcaaggctagatcactggttttcacctcggagaagagtctagtaaactccatgcaatgccttcaacaaggaagcgcctcgtgaacgacatgccaggttgaaccaatgaaagtcagacatgatagatcatgggttttcagccctggaactcgtgcccatgcactagaaaagcaactcctttattttgaagcaaaacccatagaacaatcggttctacgatgtatattaataaaagaatagaagttcaaatatgtatatagaaaagcacctcctagaagatgtcaccttgttctgccaccacttgtccatccatcatcgcatagttgttcagacgcacatttctgatagacttggcgatacaattagaaccatgcccacgtaacacatgttgttgagcagtgtcgtaggtacactcgccatcttcgctgattcatgccatcgagagtccagaccgcccacgtaaccaattttggtcaatcacatgtggcaacttggacaaggcataacccacacatcaggcaaagttaagtcgcctaatcttttgtgacagacacaatcttggaccatgtggaacactccaaacctttgctttatttgggacattgctttcaacatcaaaccaccgaattctgttaatgctttatttgggacatggcttaatgggattgagcctaacttagcgagacatattcgggttggggtttgtgctttgctgtggactatctggaactgcagaaatgatctggtctttaacagaatatcatgtatacattttttgcaggttttattccggactacggctctgatccgttcgtggtcgctactcacccagacggaggccagggagcatttggttactgggtctatccgttgggagatggtagctcgggatatcttcaaccggtttggatggcggtcatgtaatatgataggcatttagtattcctacctagtttacccagccggttgtggcatcttcatatggctagttgatgtatctagcttcttcgggctctgtgtgagcttgttctgctttttttttcacttttgaggacctttggaaccatgttggcactattttattttgttaataagagggccgtatgcatcgtactgatgcagaggccggggtatcccctttttcaaaaaaactccaaacctttgcactgagaagggagacatccaatcacccatagtgtcccgttggaacctccaatataaaagcggtcgaccttgttgaagtgtatacgtgaattgcctagccttttgcatcatttcagccttttggttgtgttggccagtgcatgaagtttaacatggtatcaaggcataagtttttgagttcaagtcccgactttcacaatttatccaaaaattgttgttgcgccctctgtatccacgtataggcctcttgagccatacctctgagtctattcacgtgttgacttcccacgtcacgtgtgggaagggatgttaaagtgtatatgtcgattgtctagcccccagtagagactgttagcagcctcaagtcgtgccgctgggagtaaaataactgatgcgcgtgcaactgtagcgacccgacctggtaagagttgaagtctctgtgcttaccgtgctagtccctggatcgacttgctagcatacacaatactcgatgaaataatagaaacaacacacatctctttattacatcgatagtgtaacatcccaattttcaatttggatgtcatacatagatcatcatatgcgtatcatatttatttatgcattttgctatgatcctagaaatcttaaacaactcaaggacccaaggagaaagttagaggtttcacaaaattcatatttgaatttatttcaaatttgaagaaggatcaatttgattttattatttttctcttcaattatttctaatattaaaaatatata

>TaCIPK10_4AS_5974107

agattttttcctaagaagtggggtcggtcaagtaaatataagcggcggccgatgagaagagtaaaggtttttaggggctaatatcgataagaaatcggccacgtccggttcaagcccccaaaaccaaatctttacttctctaactgattttgaaggattggccagagatgcccttaatttccttctagacatgtggtcatgccattgtaattaaagcagatagccgacgtaaatgctagaaatgtttacatgtaaaagaacaaggatttacatgtaaaaggactatataatttgcacgctctcaataccaaaatatcaactgaacatatttgtatcgaaatggaaatttcatctgatacaatcaatatttagaatatggagagcctatttcccagtacaaatgtttgtacgaaaaatttgagagcccgtggaaaaccggagcctcgcaatcgcctagtggcgaggttgatcatcacatcggggcccgaggaagcgtccaatatagagccagtgcaaatccttgttctttccatttcgaatgagacttgtgatgcaaggcgactaaacaaagtatttttgacccgctaagaacgcaaaacaatacgagtagtaaatattgacgaactagcccctgctgggacccttgttcagcgggtgcaaagtcggcaccctttcgggaatcggagaaagatcccaccgtggagcgcctataaatacctcctagcgtggtatttgcgcatccaatactatttcttcctccccactctcccactaccgccgctcatcttcgattcttctcagaaccttcctgcaagactttgtttcatggcttttctaccggcgcggttctaatctcctctgcgtctatagaattccagtctggatcaagtatgtttctagctttctaccgcataatatgtttgcctttaatatatgctttatgattttaatctcggtttacgattttggtatgttccaatctctcaagaggttaggagcagcaaggatttgaaactttgacgtgtatcttgtagaaacctcagcgttttaattgcatctttccttggctctccgtacccataatactagtgattgcacaaccgccgaagtactcttttgaattccgttgattcggatctgttatttttcgtagatatgcatgcatagctttgtctgttgttccgctaaatatctgaacgatttgtttggtcggtactgtacaaattgttgagttttttttgtctgcaagcgtttggagcttttcagatatttttctgttcgtttgcaattgtacgtactagatgactgtggcatgaacgggacccagtaactagatcattttcttttgccatatgttctagaacaaatagttttctcttctagaacaaggatgtgatgttcacagatcatgttccatatcatgcagctctctctgccttgatatgtttgttcaaggcaacgagaaacacacacatctggatagttcagaccaaaagatgaatgaaaataatcatagttcataagactattttcaacagacgctcaaaaaaagctctgcacactaaaagtttgtttttttggcacggaacagctccagcagacgccgtagcgctaaaagatttagagcgcgcgctgaaaaacgctatcgcgcgcagcatattttggactccggattgcgcgcgcttcataatttgtgcacctgctaaagcaatgttggtcccggcgcattaaaaatactacaatggggcgctaaaacttttattgggcgcgaaatttttgtgcggccgttggagatgctctaagaaatctaatctatgcaacaagattgctagtgcttgcccttatttttcttttctttttctttaagaggcaagaattcgctttttggattcttctggaaaataataaataccattcatatgcataaaaactggaaagtttgcctcgttcaagtgtttttttttctttatattaaaaacaattataaactatttagtagctacgtacctaatcattcatgtatttcctcgcagtattaggctttcagtgcgaaggaaaatatctccttcacaggaatgccggaagatgtgcatgcgtgctagagggctaccttggttgggaactggcagtctagcttttgaagatactgaaatacaacagttgctccatgggcatacgcaggatgatccatggaccacctaccatcttggtgaagatggttgaaacgaattgatcatctgcaggtcttgtccacatcattgagtgcccctgatcagttctgatcacctcaagttacaacatcactgccttttgtgtacataagatctggccaggatggcagagaagaagggaaatatcttgatggagagatatgagatgggaagactgctggggcaagggagctttgctaaagtttactatggtcgtaatctcaagacttcacagagcgttgccatcaaggtgattgacaaagagaagatattcaagtgcgggcttatggatcaagtaaggcgagagatatctgtaatgaagttggtgaaacatccaaacattgtccaattgtatgaggtgatggccaccaaaaccaagatattttttgtgctggagtatgtgaaggggggcgagctgttcaacaaggtccaacgaggaaggctgaaagaagatgttgctcgcaagtacttccaacagctcaatagtgcagttgacttctgccacagccgaggcgtctatcaccgtgatttgaagcctgaaaatcttcttcttgatgagaaccgcaatttaaagatctctgactttggtttgagtgcacttgcagaatgcaagagacaagatgggctgcttcacacaacttgtggtacccctgcatatgttgctccggaactgattagtaagaaaggatatgatggtgcaaaagctgacatctgggcttgtggagtcatcttgtatgtattgttggctggttatctcccttttcaggacaagaatttgatggacatgtataagaagatttacaaagcagagctcaaatggccaagctggttttcttcagatgcccggaggcttctgagacgtattcttgatccgaaccctggtacacggatctccttttcagagatcttggaaaattcttggttccggacaggccttgacaagggactgattacctataacacacgaacggagggcattgttgctgttgacatggatccgacttgtgatccgttcagtagctgtacgaccgagacaatacaagaagcaacagagctcactaacttgaatgcttttgacatcatttctctttcttctgggtttgatctctctggcatgtttgaggataagtccaacaaagagtcaaaattcacatctaccaacacagctgcaacaatcatcacaaagcttgaagacattgcaaagcgcttgcggctaagactcattaagagagacggtgggttgttgaagatgcagagctcgcaaccaggaaggaaaggcgtaatgtccatagataccgagatatttcagattgcaccaaattttcatctggtggaaatcaggaagaccaatggtgatactctcgagtatcagaaggttgaacatgatatgagaccggctttgaaagacattgtttgggcttggaaaggtgagcagccctagtaatagtgtgactactgagtaagcgtagaaattgcaagaatgtgcaatgtttacatgttggaatcaataatgtacttatctaatttttgtaggtagtttaatgttttttttgaaaaatcacccggggggnnnnnnnnnnnnnnnngggagctccccacctgaatatatttctcagttttgtaacccaacgtttgcttgattacattacaaaggtgaactacataagcacgtgtagtcgtgagcagaagtaggaacaccatgagtgggtggcctgtttatgcgccggcttcctcatccggtgcgtccaaagcataaggtcatcgaggattcgcttcaaggtgaaaatgctatggtggttctgttgcttgaaggtcatagcatttctggagtcccatatcttccataggatgatgagaagtacggaatgccagattcttgcatcaacctggcaggggaggtggcagtcccacagttcatgaatggaggtgggaggtgagagtccaatccgttgccaaattcgctgagcgagtgggcaatcgatgaagatgtgagagctggtctcgccgtcaaaaccgcatcgtggacatagagagtctgcgacgatgtgcttgcgaaggaggttgcatttggagttgagacgtccacggaacaggagccatgcaaagatcttcacgcggtttgggacgcatgtagaccagatggcaacggcgtgcatgtcatcttcattttcgcccatggtcagctggtaggcggcccgcgtggagaagcggaggccaccgttaaggaagcgctggtccggtcctggcgatatctggaaatcctgcaagagagacaacaacgaacacaactcagaagcggcattagttgttaggcggttccgtaggattgattctaaaccgatattcaaaaccgcagccacacgagtgagaggtgtggtggtatgcgaatataggcagggaaatagagtagcaagcgattgctggtggagccatgtgtctaaccaaaaataggtgttggtaccggagtttgtgataacaaaggagatgtcatgtagtgctggtagttgctggtttatgattcggcagaggaaagagtggctgttttgtggagtgataagggcatttgggtgttgcaaggtgatccattcgagccaagggttttgttctggaagcaaagctttgacagcaaatttcattagaagacagttattctgaacatggagattttttaggcccaagccgccgatcttttttggtttacaaacatttttccaggcaactaagcattgagcaccggtgcatgtttcctccgcaacccagaagaaggctcgccggatagagtctagagtttttagaacacttttaggaatgaggaagatggacataaagtataccaggatggagtcgagaacggcagacagcaaaatgagcctgtctcgtttggagagaagtttggctcgccatcctgtcaggaatttttggcatcgttcaataatgggtaaaaaggcgttaggcgggagacgggtaggagccaggggtagacctaggtagatttgtggaaaggttgatgttgtgcattgtagagtggcggcaatggagtccgcaagtctggggcatacatgcataggcaccaacgttgtttttgtgaagtttatcatgaggccggtggccatggcaaagttgtgaagaatgttttttagctgcagggccgcgtcgttggacgcttttgcgataataagtgtgtcgtccgcatattgtaggactgtcggtggtaaatgtgtgaataggggatgtgtgaggtgtaggaggtcgcggtattgattgatcatctattgaaggaggtcagcaacaatgatgaatagatagggtgatatggggtctccttgtctaagagtgttattgcagtttatccagttctccggaataccgtttagcatgacggccgtttttccagataagaggagatcctggattcaggctgtaaattttggcgggaatccatggcagtgtaggatggtgagaagtgatgcctaagagagggagttgaaggctttggagaagtctagtttaatgatcatggttggggcttttcgggaatggcaggccccgatgaggtctgcggcgaagacgaaattttcagcgatgcaacgtcctgaaatgaaaccagtctggtttccatgtacgagtaacgggacaagcggttggagtctgttggtgaggatttttgcgaccgctttgatggggcaattttgaagagaaatcggacggtaagcgtcaggcgtatttgggaggtctttttttggcagcaagattaggtgagctctatttaagcattcagtttgggcagagaggttttggaaatcggtcataaaggagaagatgtgtttttaactagattccagtattttttgtagaactgggggccgaagccgtctggtccaggactagcgagggagttcatttgcataaaagcctctttgatttcactgtcatgttttcactgtcatgttttcattatgagatgctacttctatattgagcattattcaattgaaaacatgactattttaactttggtacttgctattatggattggagaagaatctgcatcattggcgtttttctttttctctttatattctttattgggcctgtttttcttcaatatctttttcgataaaggatgttttgttacttaaaaggtttttctttactatctattctagaaaaacatcatgcgaaaaataccatagatgctacgtgaaatattgattttgtggaaccaaattgatgcaagcattatttcttaaatcgtgctgggacactgattttatgcaaaggttgaaggccccatctggcaccaatggttcagaaaactatatgatgccaaacaagtggacaattattcccactagtatgacaggttagtctcaccttgaggctccatccatccagggtcatcaaatatgacgaggcatggaacacttcagaaacatctagatcagagatgactgtcatgcactttggacaggctgcagcaagaggcaaaggcatgacgatatctgctgcatccagctgttgacgcgggcatgctgtggtgaagagttaggccggggtgaagacctgccgtcgccgagacggaaatatcttgtgtgtggctggccgctggtgatagcaagtggtgtgccgttccatggactgaagcagcttgcgtgctgatgctgcggcctttgctttgcccgtagattttcttgcgatcgcgaaagcaaggcggacgaacgatgccaccacatatctatttattcattgtccgacgcctttttgattacccagggcatcttcaacgaagatccttaaaccacctgcaaccctttaaaccatactgcgctccactggcttcgtcggcattggccgcagcggcggtccgcaccgagtaggaggaagtgtagcggcttctacgcgagcggcacacaaatggccgaacaaggctggcgcggtggaccggtgccctgccggtggtggagggacaacgatgatgacaacgccacggacggagacggtggttggctaagcaaggaggtaacacgtacgaggtggtcgtgcggtacgagttgtaggtttttttagtgttattaagtaaaacggccgaaatatcagtcgtttatgtaaattatgaataaactgaaatgaattccgtcgtgtttgattaaattccgtctgattatttcaaatagtaaaaaatatgcgactagttttggatggccggctcctacatcagtgtccctgaaatgaccatcctagtctataaatggatgcgatgtctaaattgcgagtcagcattggagatgccctagctaccaactaccaggggactgagaatggaaaatcctgtattacgctccttgcgtgaaagtgtcggacctatacactcgagcttctcagcacgagagtttggaccgacccaatataccactgcgaggcagccagtttcggataccttctagaaggttttgtgaaccgattattttgtttcttttaacggttttccgacttcttttgggttttattcattttctcttttaccttttcttttttgttttcttttttactcttttctatttttttcttgttctgtttttcttttcttattttctttttaaaaaataattcatgtttccaaaaactgttttcaatcttgaaaaagtcttcaaatattattcatgaaccaaaattttaaatttccaaaaatgctcatgcttttaaaaaattgttcaagtttatggataatgttcccgcttttcatattttgttaaacttttaaaagaaaactgtcttttcaaattttgttcatatatttgaaaaatgtttgcactttgaaaaattgtttagaagttaaaaattcctgattgtgggtcataaactaaaaaaaaattggatttcaaaaattgtccacatatccaaaaatgttctattttttaaaattctagttcggagttctaaaaaatgttcccatttttcaaaaactttttagtgttctaaattgttcgtgtttttggaaaagaaattgtgtttttaaaaattgtttggactattcaattttttttgtgatataaaaaattgttcatgtttgtcaaaaaatgtttggaattccaaaagttgttcacgtgttttcaaaaatgtgttgaaattttaaaaattgtgacacatttaaaaatatgttaatttcagttttgtaaattatttgcatttagaaaacttattttgcatttcaagaaatgtttcgattttttttctaaaaatgatttcatgtatgaacgttgttgtctgttcttatatacttcgcgctaattatcatcaccaggactggccgagtgactagctttgcacatctccaggcaccctcatatccgctccaaatgcccgggcgacctgtgttgatagaaagttgtcacccaaacggacctggcaaactcggctcaaacgcttgggttgaccgacacccctatactcggcccatatctctggggatatggggacccctgacgcttccgccatgttggttccgacaagctagatccatctgaaatcctctcaaaattgactagtccaccacttcggccgtgctcctctcgcctctgcgtagctgtccacctccttgctcaccatccgtgacgctgtcctcgtctatcgccgctcgtttacagaagacaggtgtcttcgcgtgatgggtcggcccgatggtcggcccagttatgtgcagagcctgtgcgaagccgacccagttatgtgagaagcttgtgtgtcatccacccaactatgtgcggagcctgtgcggagcctgtgccaggtcctagtgagagcatctacaatggagctctcatatccgccccaaatgcccgagcggactgctcactgcttggacagaaatatgccacccaatcaaaactggcaaacacgacccaaacagccggtcggcctgctagtcactgtctggacagaaaactgccacccaatcgaacctggcaaacccgacccaaacacttgggctgactggcacccttatacccggcccatatccgtggaggatatggcccccccaaagcatccgccacatcggttccgacaagccgaacacatctaaaatcctctcaaaattgactaatccaccactttagccgcactcctcttgcgtccctcctccatttcccgcttccgcggaaaccgcccacctcctccctcactcaccatccgtgtcgctgatatcgtcgccgggtaccctgtccgccaccatcgacatggatgcttgatgacccacaagtataggggatcgcaacagtcttcgaggtagtatttcaaccaaatttattgattggacaccaaaggagccaaagaatatttataagccttagcagttgaggtgtcaattcaaaacctggtaagttatttctctgtgaaaaaatattagtactacagtaatatggtaattttgatagtagcgataacaataataatagtaatagaaacggtaacaatagcagttttgtagcagttggaacaacaatagcaatggtagtaacttagcaaagaaaatatgagaaaagcatagacatgaagtagagatggatatttggatgacattcattatgtgacagtcacaacctagagcgatacacaatagctccaattcatcaattatatgtcgtatatagccatacatgcttataatatgaacttgcataatatcttttatactaccctcccgtggcagcggggtccgaatgtaaactaagggtaactaaggtactccttttaacagagaaccaaaacaaagtattaacacatcgtgatacatgaactcgtcaaattacagtcatccttaaagagtatcccaactattgtcaccttgggggtctacggttcagaacaataacaggtgcatataacttgcaaataggatcaagaactcaaatatatccatgaaaacataataggttcaaatctcaagtcatgacactcggggcctagtgacaagcactaagcatagccaagtcatagcaacataaatcttagaacatagtggatagtagggatcaagccctaacaaattgactcgattacatgacgaatctcatccatctccatctacctccagtatgcctacaatgaaattactcactcctggtggtgagcatcatgacattgttgatgaaaaggggttgttgatgacagcagcgataatttgccctctccgaagtcctgaacagactccatatcatagctcttagtgaagaacaagaggtagtggcagctctgcatcgtaaaacatgatgatccatgccaactcaacagacaccttcggagatacctgtagagcatctttatgatcacccagttacgttgtgacgtttgatagcacacaaggtattcttctggtatccgggagttgcatgatcttatggtcgaaggaatatgtatttgacattaagaaagcaatagcaataaactgaatgatcatatgctaagctaacggatgagtcttgtccatcacatcatgctcctaatgatgtgatctcgttatcaagtgacaacacatgtgcatggttaggaaaccttaaccatctttgatcaacgagctagtctagtagagactcactagggacactgtatttgtttatgtatccacacatgtatctaagtttccgatcaatacaattctagcagcaataataaatctttatcatgaataaggaaatataataatgacaactttattattgcctttagggcatatttccatcacttgacgcagagggtatggtggtgcggcggctatgataggatttctagctcaagcgcatgtagctgccatgattgctgagaaacgatggcgacaacacgattgacttcaatttggtggtgcttctcgagtaccccgtgtcgagcttcggggtgaaaatcctatatctaacccaaactaattatacctatcaatggtgatgtttttgtgtcgtgaccttgttgaaggccttgatcggatatgctcatgcttgttcttgggtgtgaaaaccttgaatctcgccattagtggttagcacgggcgacgacggttccctttttgtaagcgttgtggtatttttgataaacctggcccctttgcacgacttgaggcacgtctggcccttgggcaaaactaatttgaaatctggcccctggggtcggcgccacatatcatggagccgaagttgtacatgttggcgccacactacacggagccggcatgtacaagttcgacgccgtgctagatgaagccgaccttaaatgggcccacagaaaccctaagggccagatttgctcggctccacatagtatggcgccaaacttatacatctcggtgccatgtagtgtggcgccgacatgtacaacttcggcgccatgatatgtggtgccgactcaaggggccagattccaaattagttttagttttgcccaagagccatacgtgccccaagttgctcaaaagggccagatttgtcaagaaacacagcgttgtgtagtttgatgcggatatgattgtcattgtagtttgtcgattgctgtttagattgctttgggattttcttttttcatgctaaggcatagtttggtctcgtgtgactttgctattcgccgacgtgtttagtttagttttttgtgggaacactaacgtgtttagttatatgtgtgtttatgttggttgtgtgaatcctaattatgcagagcttgagtttatgttcattgtgtttgtatacatttgatgcttcgtttgagttaataaaacccacacattctcttaaaaaaataagtagctgcgacagagttggtttgtggtagatcaccttcatttcaagcccggccggttgctgagcaaatcaaggcagcagatggcgcgattagttgctagcagtcgacagccgtcatgcattagctagagagaccataccgattagtcaattctgttagcaaagcctcaccgcacttcatcaaacgaccgagatctccatttttcctacctactatatggaaatcatgaacaacattccgtacaaagtcaaacacgcacacaactttaatgaattattagttgacgatggtgtctttcttgatttaatgaataatgatcatctttgtttgcgcaactctagctttgctggaactggaatcgagcctggcgacgagggaactggcatgagtgggctatataagcacaacccgccgtgccttgtgtcctcacccctcgctcttgcacacagatcgttgatctatcccgctcaggcttagtcagccaattaagctgatttgtagctaggtctaacgtgtgcagaagcagtacgtcgatcgtacttaagtatggcttcccgcagcagcttctctgtggcgtgtgctgtcgcagtgatggcgtttgccattttggccacggcgagcaatgcgcagccgctggaccctcatttctacgacaagttgtgcccggcggcgctccccgccatcaggaaggtcgttgaggaggccgtagcggtggagccgcgcatgggtgcctcgctcctgcgcctgcacttccacgactgcttcgtcaacgtatgcacatatctatatgagcgcgggtattgcatttagtttggtttgaagacctactctactcgtttgacgctggttgctgccggtttgcttgcaggggtgtgatgggtccatcctgcttgacgacacgcccttcttcacaggcgagaagaaggccgcgcccaacgtgaactccgttcgcggcttcgacgtgatcgatcgcatcaaggacgccgtcaacgccgcgtgcaggggaaacgtggtgtcctgcgccgacgtcgtggccgtcgcagcacgtgactctgtcgtcgcggttagcgcttcctcgtctccagtatactcatcaggagaaatttatccgcgtaacaatggatggaatatattattactactaactagcagtaagatattgttgcagctgggagggccgtcgtacgacgtgcttctgggccggagggacgcgcggggggcgagccaggcggcggcgaacaacagcatcccggcgccgaccttcgacctcgaccgcctcgtctccaacttcgcctcgcacggcctcactgtgcaggacctggtcgtgctctccggcggccacacgctgggcttctcccgctgcaccaacttccgcgaccgcctgtacaacgagacagccacactggacgcctccctcgccgcgcagctcaggggcccctgcccgctcgccggcggcgacgacaacctcgcgccgcttgacccgacgccagcgcggttcgacggcgggtactacggctcgttgctgcgcagcagggggctgctgcactcggaccagcagctgttcgctggcggtctcggccccacggacgcgctcgttaggttctatggcgccgacccggaggcgttcaggagggacttcgcggaggccatggtgaggatgggcggcctcagcccgctcaccgggagcagcggcgagattcgcgctaactgcaggaaagtgaactactactagtatgcgcgcacaacacgaagtttgaatcaaatgcagtgtgtttccggcaacggggcatagtcaaacagtgttctgcgtttttttagttgtcttgtatggattgcatttagtattgtggattcttgggctctagcactttatttgattccgttgtaggatttcttaagtggaaacagtgttcccggtcgtatatatatatactagccatgcccgcgcagcgccgtgcgatgttgatgtattgcagtgaaatttgttgcgtgtaatagtgaagaagttacaacatattgtgttggtactgctgagttgcctgtattttttgagtacaccacaacttacgataaaagatgtgtatatgtgattatatagctataataaggtaagtggtgtgccaaggcagatttgaacactttgatttattacattacacaattgatctctc

>TaCIPK10_4BL_7036526

gaggcgtgacctcggcatcggtcggtgatgatcgatggttctctgcagtgggagttgagatagtgggggctagctacccgggagacttgaccaggacagcagaggctcgacgcggtgatagcggcgaggcgtgcggtaagcacgggacacagcgacatgccaaggcactggtggtcagacatgcggccagataaagtgtgcaagggggtgctgaagcagtgttgacgagtaccggattcaagttggtgactgggtctatcggtgccggttgatggacaagagttgaccatggagaatctgagaaagtggcgaaaagtctggatttgtcaaaacagagagagtacatggttgtatattctgtggtgttcagtgcacatggcaaagtgcggatgacaagtacagaaggaggcggagtgctataattgtgggacatgtcagagactatccggggaaaaggatgagacaactgtgaatttgactcagggtgacacaaggcgacggtgaaattccttcaagtttcagacagatggtcaagaaagagcgacgatgttgagttcaggtaactcttgtatgtgacatccaatatgtgagttcgtttattttcacgcagataaattaccggtgtgtgatggcgttgaacggaaactccggaagttgggagcacaaagtagagcaatgagaaacttaattttgctcaagtgttgactgtgaagaagacgagaagggactacagttgcaggtggagtcatatggagtccgggagtagcagcggtgctcatggcgtatctcaagtccaatgtacatggaggttcgtcgcacgaacgaattcaaggtggtggagaatattcgtcaaggtggagtttgttagagttgtgtcgaatattattgtacaagttaggttacagttggacttgaaatcgtacgtgtgttgacaggatatgaaatcgtgtccaagtaggacacttgtatcctaggcctctcatatatagcggggatagacacacgatgtaacctatgccaacataatagcacgggcacgcgggggagcaggcggcgtgtgccggcgcccgggcggccggggtgcgatattgtgacggtgtcatggggagaagcgcccgtagtcaatgcccggaggatgtagccatgatggtgaacctcggtaacaaatgttggtgttgtgcttgtgtgattgcttggtcctcggtagatcgacggaaatcctcgaataattctaacataccgtgcttatgttcgatcagtctttgcgcaaactctactccaacgttctgtctcgtcgtttggctcccggcctttcaaagattgttcacaaaaaaaaactgatattcagaaaaaaaaatgaaaattttcacatcaaagacgctcaaatgcgctggttgcatgaaaaaaattcatgttgaaattacatttgggaagcgctaaaaacaataaatggccgtcaaagaaactttgaaaaggtcagtgctgagacccgtattctttttgttgagagctactccggtgtcattttaccacaaaactttgcagccacttagcactcaagcatcttcgatgctgaaaaatttcgaattttatttttggccttttttggatttactgttcattgggagtatatgagcttgggcttagtggtgaattaccgtttctcatttgtcatctatatgaaaggccagtgctctcaccctactccccaaaacataatacttagggcatctctaaccgatgcctcaaaatttaggccgtcaaaagcctccttataccttaactctttatattggaggagctaaaaaaattatatggttcctagcccaaatcttaaatttaactcctcaaactttttttcacagttttattcaaacaaagattaaaaatagtcaattcaaccacacatttctattcaaactgtactgcataacatgaaatttaaactacattggataaactactcgatttagaaataaataaaaaaaaactcctcctctttcatgagcgtctcccagtccacgtcatcatcatagtggtcaccgaagcaaaggtcaatgacctcaccacctgacggcccagccttgttggtagccccttctggtacatctcgagcaactgacgctcctcggcgacgagctccgaatgcatcaagcggagctcggtcatgtagtcctcatcaaagcgcgccatctcaagccgttcaaaggcctcccggttctcccatacctcgccgcgggagaccactcgagggtcgatgcctatgaacgttaggaccacgcagctgctaaatgggaacttcatgttctcacggccttcatgtcgtaccctctcgcgacgagttcagcccactcgaaggagcgaagccagtaccgatggatagtgtcctggtccagtatactcctcttcggcagtggccgtggcaggttggtgaaggagtgggagttgccgccagcgcgagctcgggttggctgcacgccatggatggatccacgtttcgaatcggcggcagggacctccgacggtgatatcccggcatttaggggcggcgccgaccgattggaagcatcagtgggggtgtgggggtggttgcggtgcgggcgggccggagggtgtgggcaggagttgccgggtggatatgggcagcgccggagtggatgtggtcaaggcggaaagaggtagccgcagggaggggattgttttttttgagaagtggcgtcgttagtaatataagcggcggccaatgagaagagtaaagatattgaggggctaatatcgataaggaatgggctaggtcctgtttaaccccgcaaaaccaaatctttacttctctaactggttttgaaggattggctagagatgttcttaatttccttctagacatgtggccatgccattgtaaagcagagagccaacgtaaaagctacaaatcccaactttgtactaactttaatataaagttgggtacaaagttggatcatctattttggaacggagggagtaggaaataaaccatgtgtgtcatggctgtgtgtttagtgcgggcgtgtgtgcccgtgatgactagtgtgtgtgcgcgtgttggatgcatcagtgaaataagtctcgaggtagttagtacgtgcaagctaattgcatggatgcttggccgggtctcccggaggagtgagtggctcacgacgagaagaggccgattccggaggcagtgcgcgcgtggggttacgcatgcgtgggcgctgcgtcttctggtgtataaagtcaagatgatgtagttcttgagtgctgtcaaacccgtgaataaaggttgccaagatataggccggttcgccggccgcgatgttcgtcgccattgctccgagttcgtgctcgagttgagtttctttgtgcatatggtttccgggtttgcgccaacatatttcccagtatttctgtgaaacaaattgacagcctgtggaaaaccggagcctcacaatcgtctagtggcaactggcgaggtcgatcatcagatcggggctccaggaagcgtccaatatagagccagtgcaaatccttgttcttccatttcgaatgagacttgtgatgcaaggcgactaaaaaagtatttttgacccgctaagaacagaaaacaatacgagtaataaatattgacgaactagcccctgctgggacccttgttcagcgggtgcaaagtcggtaccctttcgggaatcggagaaagatctcaccgtggagcgcctataaatacttcccagcgtggtgtttgcgcatccaatactatttcttcctcctcactctccactgccgccgctcaccttcgattcttctcagaaccttcctgcaagactttgtttcttggcttttctaccggcgcggttctaatctcctctgcgtctagaattccaactcgtctggatcaagtatgtttctaactttctactatctcatatgtttaccttttatatatgctttatgattttaatcctggtttacgattttggtacgttccaatctctcaagaggttaggagcagcaaggatttgaatctttgacgtgaatcttgaagaaacctcagcgttttacacgcagctaattgcatctttccttggctctccttacgtgattacacaaccatcacccgcaaaaaaaaaagtgattacacaaccatgaagtactctttagaattccgttgattcagatctgttttttctagatatgcatgcatgccttggatgatcattggttgggagttgggactaactttgtctgttgttccgctaaatttctgaacgatttgtttggtcggtactgtacaaattgttgagtttttttgtctgcaagcttttggagcttttcagatatttttctgttcgtctgcaattgtacgtactagatgcatgaacgggactcagtaacaagatcatttcttttgccatatgttctagaacaaatgattttctcttctataacaaggatgttgttttcacagatcatgttccatgtcatccagctctctctgccttgatatgtttgtttatggcaaagagaaacacacacatcttgatagttcagaccaaaagatgaaaataaacatacttcataagaaatcttatctatgcaagattgctagtgcttgcccttatttttctttaagaggcaagaatctgctttctggattcttctggaaagcaataaataccattcatatgcgtaaaaactggaaagttttcctcgttcaagtgttttttctttattaaaaatatattctaaactctttagtagttacgtacctaatcattcgtgtgtttcctccttgcagtattaggctttcagtgcgaaggtaaatatctcctccacaggaatgccggaagatgtgcttgtgtgctagaggactaccttggctgggaactgacagtctagcttttgaagatactgaaatacaagagctgctccatgggcatacgcaggatggtgctatttgtggccgctcaaccatctaccatctttgtggagatggttgaaacaattgaccatctgtaggcgttgtccacatcattgagttctcgatcaccacctcaagttacaacatcactgcctttgtgtacgtaagaagcggccaagatggcagctgagaagaagggaaatatcttgatggagagatatgagatgggaagactgctggggcaagggagctttgctaaagtgtactatggtcgtaatctcaagacttcacagagcgtggccatcaaggtgatcgacaaagagaagatcttcaagtgtgggctatggatcaagtaaggcgagagatatctgtaatgaagttggtaaaacatccaaacattgtccaactgtatgaggtgatggccaccaaaaccaagatattttttgtgctagagtatgtgaaggggggcgagctgttcaacaaggttcaacgaggaaggctgaaagaagatgttgctcgcaagtacttccaacagctcaatagtgcggttgacttctgccacagccgaggcgtctatcaccgcgatttgaagcctgaaaatcttcttcttgatgagaaccgaaatttaaagatctctgactttggtttgagtgcacttgcagaatgcaagaggcaagatgggctgcttcacacaacttgtggtacccctgcatatgttgctccggaactgattagtaaaaaaggatatgatggtgcaaaagctgacatctgggcttgtggagtcatcttgtatgtactgttggctggttatctcccttttcaggacaaatttgatggacatgtattagaagatttacaaagcagaactcaaatggccaagctggttttcttcagatgcccggaggcttctgagacgtattcttgatccgaaccctggtacacggatctccttttcagagatcttggaaaattcttggttcaggactggccttgactcgggactgattagctataacacaccaacggaggacattgttgctgttgacatggatccgacttgtgatccattcagtagctgtacaaccgagacaatacaagaagcaacagagctcactaacttgaatgcttttgacatcatttctctttcttctgggtttgatctctctggcatgtttgaggataagtccaagaaagagtcaaaattcacatctaccaacacagctgcaacaatcatcacaaagcttgaagatattgcaaagcgcttgcggctaagactcatgaagagagatggtgggttgttgaagatgcagagcttgcaaccgggaaggaaaggcataatgtccatagataccgagatatttcagattgcaccgaattttcatctggtggaaatcaggaagaccagtggtgatactctcgagtatcagcaggttaaacatgatatgagaccggctttgaaagacattatttgggcttggcaaggtgagcagccctagtaatagtatgactactaagtaagcgtagaaattgcaagaatgtgtaatatttacatgttggaatcaataatgtgcttgtctaatttttgtaggtagtttaatgtgtttactgtcatgttttcattatgaaatgctacttctgtattgaccattattcaattgaaaacatgaatattttaactttggtacttgctactaaggattggagaagaatttccaccattggtgggttttctctctttatattctttcttaggtctgttttctctactactccttccgtaaaaaaatataagagcgtttagatcactagtatctccgtaaagaaatataagagcgtttagatcactagtagctctctccgtaaagaaatata

>TaCIPK10_4DL_14429928

cggtggtggctggcggctcaagggggcggaggctaaagatgaactgcaggcctttgatttcgcatccaacggctggaaaatcgactgcccagagatgaaaaactcagctgactgatttctagccattcctgagaaaaaagaacccaccaatgatgcaaattcctctccaatccatagtagtaacaagtaccaaagttaaaatagtcatgttttcaattgaataacggtcaatacagaagtagcatttcataataaaaacatgacagtgaacacactaaactacctacaaaaattagacaagtgcattattgattccaacatgtaaacattgcacattcttgcaacttctacgcttacttagtagtcacactattactagggctgctcaccttgccaagcccaaacaatgtctttcaaagccggtctcatatcatgtttaaccttctgatactcgagagtatcaccattggtcttcctgatttccaccagatgaaaatttggtgcaatctgaaatatctcggtatctatggacattacgcctttccttcccggttgcaagctctgcatcttcaacaacccaccgtctctcttcatgagtcttagccgcaagcgctttgcaatatcttcaagctttgtgatgattgttgcagctgtgttggtagatgtgaatttggactctttgttggacttatcctcaaacatgccagagagatcaaacccagaagaaagagaaatgatgtcaaaagcattcaagttagtgagctctgttgcttcttgtattgtctcggttgtacagctactgaatggatcacaagtcggatccatgtcaacagcaacaatgccctccgttggtgtgttatagctaatcagtcccttgtcaaggcctgtccggaaccaagaattgtccaagatctctgaaaaggagatccgtgtaccggggttcggatcaagaatacgcctcagaagcctccgggcatctgaagaaaaccagcttggccatttgagttctgctttgtaaatcttcttatacatgtccatcaaattcttgtcctgaaaagggagataaccagccaacaatacatacaagatgactccacaagcccagatgtcagcttttgcaccatcatatcctttcttactaatcagttccggagcaacatatgcaggggtaccacaagttgtgtgaatcagcccatcttgtctcttgcattctgcaagtgcactcagaccaaagtcagagatctttaaattgcggttctcatcaagaagaagattttcaggcttcaggtcacggtgatagacgcctcggctgtgacagaagtcaaccgcactattgagctgttggaagtacttgcgagcaacatcttctttcagccttcctcgttgaaccttgttgaacagctcgccccccttcacatactctagcacaaaaaatatcttggttttggtggccatcacctcatacaattggacaatgtttggatgtttcaccaacttcattacagatatctctcgccttacttgatccataagcccacacttgaagatcttctctttgtcgatcaccttgatggcaacgctctgtgaagtcttgagactacgaccatagtaaactttagcaaagctcccttgccccagcagtcttcccatctcatatctctccatcaagatatttcccttcttctctaccatcttggccagttcttatgtacacaaaaggcagtgatgttgtaagttgaggtggtcagaactgatcaggggcactcaatgatgtggacaacacctgcagatgatcaattcgtttcaaccatctccaccaagatggtaggtggtccatggagcatcctgcgtatgcccatggagcaactcttgtatttcagtgtcttcaaaagctagactgtcagttcccaaccaaggtagtcctctaccacacaagcacatcttccggcattcctgtggaggagatattttccttcgcactgaaagactaatactgcaaggaggaaacacacgaatgattaggtacgtaactactaaatagcttagaatattttttaataaataaaaacacttgaacgaggcaaactttccagtttttacgcatatgaatggtatttattactttccagaagaatccagaaagcaaattcttgcctcttaaagaaaaagaaaagaaaaataagggcaagcactagcaatcttgttgcatagataagatttcttatgaactatgattattttcatcttttggtctgaactatcaagatgtgtgtgtttctctttgccttaaacaaagatatcaaggcagagagagctgcatgacatggaacatgatccgtgaaaacaacatccttgttgtagaagagaaaattatttgttctagaacatacggcaaaagaaaatgatcttgttactgagtcccgttcatgcatctagtatgtacaactgcagacaaacagaaaaatatctgaaaagctccaaaagctcgcagacaaaaaaactcaacaatttgtacagtaccgaccaaacaaatcgttcagatatttagtggaacaacagacaaagttagtcccaactcccaaataatgatcatccaaggcatgcatgcatatctacgaaaaaacagatctgaatcaacggaattctaaagagtacttccgcgattgtgtaatcactagtattatgggcacggagagccaaggaaagatgcaattagctgcgtgtaaaacgctgaggtttctacaagattcacgtcaaagtttcaaatccttgctgctcctaacctcttagagagattggaacataccaaaatcgtaaacccagattaaaatcataaagcatatctaaaaggtaaacatattatgagatagaaagttagaaacatacttgatccagacgatttggaattctagacgcagaggagattagaaccgcgccggtagaaaagccaagaaacaaagtcttgcaggaaggttctgagaagaatcgaaggtgagcggcggtagtgggagagtggggaggaagaaatagtattggatgcgcaaacaccacgctaggaggtatttataggcgctccacggtgggatctttctccgattcccgaaagggtaccgattttgcacccgctgaacaagggtcccagcaggggctagttcgtcaatatttattactcgtattgttttctgttcttagcgggtcaaaaatacttttttagtcgccttgcatcacaagtctcattcgaaatggaaagaacaaggatttgcactggctctatattggacgcttcctcgagccccgatgtgatgatcgacctcgccactaggcgattgcgaggctccggttttccacaggctctcaaatttttcatacaaacatttgtgctgggaaataggctctgagttgttccactccatattctaaattttgattgtatcggatgaattttccatttcgaaacaaatatattcagttgatattttgatattgatagcgtgcaaattatatagttcttttacatgtaaatccttgtagtattatttttcctctaccaactaccgccgatcaagtcgatcacgtcagcccctccccctcgcgttgcctcccctcaggcggcgccaggggaccccaatcccgcgccgccaacccctcccctctcctccatccccagccgccgttgccgctggcaagggccgcnacggaggcggcgtctcacgtggaggccgggggcggcaatcaggggagcgcccctgctgcgcggtggcgatgggcaggggcggagctgggccccgggcatccagggcccaggcccggggcgtggagagattaatagactgtaatcaagtaagtttttctgcaggcccgggttgcagcccagcgaaaagaagaaggcccaaagctttgatgggctgtcgcacgcaggaggaaaaactccacccccacgatctagcacgcacgaggcacaagagactcatcgctgttggtaaaaaaataacatgaaactctcgcgcagccgccgccagtcgcacggacgcaggacggcaagatcgatctccacttctccaggagggactccaacgcccgcgccgccgtccaccgtggctacgcgcctccgctcccggcccacccattgccccccttgcctcccggacctgctcgtcgttcacacgcgtgtgctgcttcactgccgtctgccgcagcttgtacttgtaattttctttgatgctttccctgtttcccccttatgccttctacttatactagatgaccagttgcgccaatgacgcaaaggccaagagtaaaccatgtattaaaagagtgtacattaatattattcgggcacatattaaaatatatgaagaaaaaaatccacttactataaatacaagtgatacaatacccatataatgttcaaggaaatatccgtataaacagaaccgttaattagtcagacacaaatccacacccgagaagcacactgcacagggttgaaacttttaagcctcacattcccaagcaatcttaaggagggccaacagaattgaaatcacagatgcaaagtatcagcagttagtaatatttatacacacagcttctttattgtagtcatatgatgatgaaatcagtacgtaaatcagcaccaaggatagccgatcagacaaacaaaaattctgcatcccgctgaaaaacactattatgcaagactcattttaaaaggccctcagttctaatttctataagagcttgaagctttggagccaaggctacgaaccttcaaatccaaccaaacatcttaccatgcaaacacggtaaaatgggaaagaaagactaaatgtgacgcaccaaaagcggaagaaaatgttgaacgacctgggcgtgaggattgtgccggtgacctggtgacgccgagcagcgcgtggatgtggaccaggatgacggtggtggcggcgccctcctcgtcgaccttcagcagctcgctagagtctacaatgctcatactttgcatcaagtaaacccatctcactaagaacgggaatgagcatcgccctttggcatgctctgcctttggcatgatcttgatagcatccgcttcctacaatagaacatgcaaagagaacataatgaggataatattatgcatgcaccactctgcacaaaaacctctccaaggaagcagcttttattactctacctccagttctctagctttcaatattaaaggaaaaaagcaattcccactaattgtatagcacatagataaaccccagattacaatacacaaaacagagcaaaacttcttcataattcaattaaaatgaggccatatgcatcatgattaaagttgctccctgtattgtatatacaaccatgattcaaagtgctccttgtactgtatatacaaccatgtatatacaactatatcatttataggccatgtatatacaacttaagaaaacataaatacataactgcatatgcatctgcatataacaaagcaggtgatccaatctacaaatattacaatagcaattgtggcagcacaaaaagtgtaattggacatcaccatttaacaactaaggctataaaataatagatgattatgcagatagcaaagaaagattcaacattattgtgtcattgacaactttttcaggtagtagcaaatcaacatatctgctgctaatagaaacagaatccggatctcctttagggaggagcacatacagagaagagaaggagtaggtgttcgtcaccagatgatcattttgctcggagacaaagatgaggaactggacccagccgataggtcgttcttggtttccatgtctagttgggttaattcagaatctgtttagatcttttctacactactgccatgctgtgctataagccaaaaccagataaccaggtcaaaatcctagctgtgactttaacggttctgttggaaatatgccctagaggcagtaataaaatggttattattatatttccttgttcatgataattgtctattgttcatgctataatt

>TaCIPK11_2AS_5192052

attgatatgaaggaagggaatattagatttcaatttccattaaaaagggcatggaacactttccaagaaagaaaataaaattgccatatgaaactatcatgcgagccacttatggattgcctaccaaagatggcaatacctagatctatcctcacttttatgcctagctaggggcgttaaacgatatccccaattttgtttttattccttgctttttgctcttgtttagtaataaataaattatttagcctctgttttggttgtgttttttgtgtttaattagtgtttgtgccaagtagaaccgttgggaagacttggggaaagtcttgttgaacttgctataaaaatagaaactttagcgctcacgagaactgttgtcatttttatttgaagagtgatatttagttaatggaggaaagggtcagaaaaatcataaaaatatgaacaaaaaattaaaaccaatttttttgtgaatttcctgaaacattttttatttgtgtagatttttttcaaaataggaatattttgaattttagaacaaatattgaagcaagaacattatttggaacacccaaacattttttgagtttctgaacaagtttgaaaaaagtgaacattttttaatttgtgaatgaattttgaaaacaaagacatttatagaatcgttgaacttttttcaaaaccgtgagcgcttaacccgtccgaataggcgcctagccggagcgatgccatcgcctagttggcccaaagcctatgattttttttcttttcttcttttttccctgttaaatcaattttctccttttaaactttatttttcttttgaaacttgttcaaaaaattgaaatatattttggaataccataacagttttaagcaattgtcttacattacgatttcaattttttccttcagatattgttaaatgcttttttaaaagcatttactgctttcagttttatccctttgtatttattagttatgtttaaaacttaacataacaactgtaagtaccagtttaaattataattttaagttcttttttccttcaaacctgagatggatgccaatcatgggcatgcccagctactcgcaaaagttggggtcatttgaagaatgaccaaaaaacaccatgttcaacctagagtgtcagtgtgccataaggctttgttttagagcatgttgtttcttgtcatgcgtcaaatgagcctattttttcatatgcttatttgactaatttaagggaccatgccaagttgttttggtttctgacaatttttgtagtttctagagttttctcagtcaaaaatgccgataaaaggctggacgtgtccctacttgcacatggtgtcgcaaatcgttcaaacctgacatggatgccttgcaaaagctgaggtcatttcgaaatgtccaaaaatataccgtgtttaaaggagggtgtccaggtagggcagaaggctccgtctgacagtacgttgtttctgaacatctttgaaatgaccctaatttttttctcatggtcttgtgtgattagccaaagcaccatgtccaattgtttgggttttcaacaagtttacattttcctggagttttcttggtttaaaaggaccaacaaatgaccaaatgtctcccaacttgactacggtgtcgaaagtcattcaaacttggcatggatgtctatttgggaggttagtttgaagaaggagccttgtcataacgcttccgctgtcggtctacgagggtacgtagattacactctcccctcttgttgctatgcatcaccatgatcttgcgtgtgcgtaggattttttttgaaattaacgttccccaacagtggtatcagagcctaggttttatgtgttgatgttatatgcacgagtagaacacaagtgagttgtgggcgatataagtcatactacttaccagcatgtcatactttggttcggtggtattgttggatgaagcggcccggaccgacattacgcgtatgcttacgcgggactggttctaccgacgtgcttcgcacacaggtggctggcgggtgtcagtttctccaactttagttgaaccaagtgtggctacgcccggtccttgcgaaggttaaaacaacaccaattgacaaactatcattgtggtttcgatgcgtaggtaagattgattcttgcttaagcccgtagcagccacgtaaaacttgcaacaacaaagtagaggacgtctaacttgtttttgcagggcatgttgtgatgtgatatggtcaagacatatatattctgatcacaacctgaactgcctcggtaacacgcctggacttccctttgtacgaacccgaccttcgggctatcttcacaaccgtggcttcccccgcgaattattctgtttagtcgtgttctatatgatgttagtgtaatctaggaatgtttttagcaactaaataccggttttaaataggaatgtcgctcgttggcggattttgctctcgactcatgatgaaattgcgctttttgcaattttactccctgagttgttcgacctgaacttcttccagtgctaggttgaacttccgccaacatcgcccaaacggggcttgcgatattccgttggaaagctatgaacattagtatcatgacccaagttaaatttttggcaaaatgtaagcggtttaagagcagttttgaaaaccgttttttcttcatacaaaaaacgtgaatcgtatatttgatcgcatttttaaaccgtttatcggaatgaggcaaataatatgccgttggaaactcctgcaaaaccactccttccacatgttgaaagttttctctaattccctacggttaaagagtaattcggaaaatcgtagaatttcccaaacagaacatccgagttcacattttcgatgccatttctaaatggctaatccaattgaggcaaataatatggcgttggaaagcttatgaaaatgcgctacatttcatgttagaagttttttctaattttgtacggtttaaaagtaatttagaaaacggtacaagttccactgagttcgtattttcgagctaattttttaaccgtacgtccaaatgcagcaaatgatatggcgttagaaagcttgaagaaatgcgaaacttttttgtatatattgtttctcctaattcattacagttttatgtctgttatgaaaatggctaaaaacgtatttttgccataatttctacaaactttatcggaatggggcaaataatataccgctgaaaagctacggaaaattcgaaactttttcatgttgatggttttctctgaatcttggcctttttcaagtaatttcgaaaacggcgagatcatacattctgcctttatcgtgaaacagattcttcaaaaatgcatcacgtgaagaacctgaacttctcggtgcgtgtacctgaacttcactttgttttcacttttttttctcatagctctccattcactcaccggaattgagcaagtgatataccgatggaaaattgctgtaaacacgcaactttcccatgttgatcattttttcatacttgcgacggtttaaaaaaaattcatgtgaaattcatatagtgtaggagttgaacttcctgttttttcacgttgaacttctgtgacgtatttttcgtcagtgttacaccaataatatcccttgtatacaaacatctctcacaatatttcttttttaactttctccgactgaggacttgaacttataagaacaataaaattggaccttgcctttttattcatttttctcatattaaatatataccatgtagtacatgaacttgttccatttttataatttgattttttttcttttctttttaaatcaaattgctcccaaaaaaatctgaacttctttgtggattgagataattattttaaaactcaaaaaacattttttcttgtgtttttgaacatggaaatacaagatgaaccactctcgcaagaatttttgaacttccccagacatagcacgtgaacttcttgcaaaaaaccttttaagcgttttgttttccattcttattttcttatgtgaaatgcattccgtgtagtagttgaacttcccgctatttttacgttgaacttgtgtcacgtatttttgttcaacccctctcataagaatttttaaacttccccgagcacatcacttgaacttctagcaagaaaaattatagactttgccatttttattcttcttttcttgtatgaaatacacaccatgtagtacgtgaacttctggttgttattactttgaactactctctgatttttgccataactcataaagcgttcatcaaaattacgcatgtaaatcctaattttttttataaactatgaacctatctgttttttcaagtttgaacttcttagttttatttttaggaaagaaaaatgatttgtaactaaacatcgaacacctttttttgaaacttgatacattttttggcctaggaacaagaagattttgagtatttttgtatacatcaaactaatcctttttttagttggaacttcttgactttttaaatagaactacttaatttagaaaacgaaaaaacgacgttaccctttatcaacgaaaaaatatgattttttatagacatcaaaccactctatttttttcaacttgaaaccttttgtgtatttttagaaatgagaaaatttggatttttgtaataaatatcaaacttcttcgttatttcaaattgaaattcttggctattcgttagtaagtggaaaaattcgagtgttgtaataaatatcgatttgcttcgttttaaaaaattgaacttcttggttttattatctagtaatgcgtaaagtatgagttttttcatatacattgaactgctcaagttttttaaaattgaactttttggtttttttaaattggacttctttggttttgtaataaacaatgaacttctctgttgttaaatttaaacctttatgtttttttagaaatgggaataattcattttttatacaatgaacttctctattttttaaatttgaacttctgggttattctgggttatttttcagttttggtgccctggatgtatgaacttcttggacgaatggttcttttccatgattttttttggacaggatatgcactcctaaacagaacatatgaaattctaaaaaaattatatatttcttataaaaacttgaagaccacacaatcataacataaaacaaacgcatgacatgaaatcagtaaagaacacatacataaacttgtgtattttcttttttaggatcataacagtgacccatttctcttttagaaatttggaaatgacagtgcagcagtatgtaaataccatattcggttagaagttgtcagctgctgctgctgcttcaacaacaatacttagtactccaccagatcttgcctaattaatttagtggtgcatagtagttgatagcaacaatagtaccaagttagaatgccaccagatcttctctaattaacaaacaacagtagtgctgagtagtagggtcttgtgagttgtgtgcatacaccacttcaatttctacaagattcatgcaaactcgcgacaatatttttcactgaaccaacaacctaaactggtgcaagcctgcaacaatcacaaaaacaacatctgacatactattgtttttattctttcttctctaatatacattgaactgctgcaaaaaatagaagaattgtacactgctaggcatcaccgcgttggttgctgaagtgcgggagcaggtgaccaagcaggccagttgcaagatttcaggcataatacaccttcgcaaaagttccttgccccaattgcttcccgatttcataatggcccatcaagatagtcctcctcgcatccatcatatcaagttatctgaatggagtaaaacaagcgaacaccacacacaccgcttatataccagtatctaagactgtatgcctcatgaatggagcagatgttaactgtcttcacaaatgtgattatcaacaaatactgaccccttcggtatactgtgcttcacaccaaaaagaggagaaacagtaccagtatgcagcacattatcatgagagccccacagcatgagcttgaacgaagatatgatcttgtttgtgcaaagtaagtgtttgctgttatgcaagcacatctttctgcattcccaagtcttcttctggtgaacaactgccagccagaagatcagattaaattataatggatacatgtaatgcagggtgggattacgagctgtaatgaaaaacacaaatccagtggcctttgaggacattaagccaaatcttgggtgacaccccaatgtggtcgatcaaactgggacggtacagagtaaacaagagcatctacagcaatcagagccgccatggttggataattgggtgaagcaaagatgccatctcttgagttctagaaacgataatttcgaaaatacaactcacaaaccgagcattcctgcaacaggcatccaaaatcggcaaaattcacgcccagtacggggcgaaacagagctttcacaataaacatctgaaatcattccttgcaagagtcacgagataaactagagttcacaataatgttcttttttgtttggacggtgcctgcaccccaatgtggacttcagcgggccgcatggagcactccagagttttgactgcaggtttcgccggcacgacaagaacagaggtatattacagacgcggcaacagagagctattgacggcgtatgaggcagacgaggatgggcatgtctgtggatccccggactgctctttgtacagcctgaagtccaaatccacctcgccgtgaaggcgggggcgggggcgtgctaggagaaggttggagcggcggcctcggcccggtagtagttggaggcggtggcgggccaggaggaggcgggtgcgacctcgcgctggaaggatgcagggtcgtcagcgcaccaggacgaggcaagggcggcggcgagtcggaatgaagcgtgggcggcgatgagctggaaggaggcggggacgggaggtgcgccggagccgagcgg

>TaCIPK11_3AL_2838468

ttaaaaatgtttttaattagcttttaaaaataaaaaatagaaaaccaaacaaaagcgcaactaaaaaaccggctataaacagtaataaggaatatatcgttccctttcgtcagagtcgctgcagtcccaatgcgaaagggaaaatcttgttcttggcactctagattttaccaattattgttatgttactctagattttgacatttcactggtgccactcttagcttttgacaatgatcacaattgccattccatggcaaaagtaaaataattttatttcattgatcgggaagcaacagcaagccactaggacatgcggctgtcatgagaatcacaaatgcggtagccaatatgatctgtgaaagcatcatggcatgcttcactgtcttctccgttgctgttgttcactctgcgcctcggatgccatacattcatctgttcaagtggcaaactcacgagctagttacaattatccccaagaaggcttagttctaccccctctgtcctataatataagaacgttttttacactagtgtagtgtcaaaaacgttcttatattataggacggagggagtagttagcatgggtcctgaatcaaaagtcagaaccgattagatcagtcataagacttctcgaatcaaggagcatcgcacccagttttcatctctgaaaactgataaaacaacagacaagccggcctcacgttcagttcctgtcacgaacccacaagaaaaccagtctcgacgacagccactcggggggtaatcgtaatcggaactaacgcagcagcaaactccagctaccaaagccagcaaaaaacagcagagcttatcactaccagacagtagcagtaactcagaaacatcaaaacaagactaatcgggaactttcatctcggcgtagctcctccttgttctttttcccatcctttttgctggtgtcttgtcgatccttctacatccgcgcccaacctcgacgtctgctaaaaaaaaaccagctgccactttcaaccaagcaaacacatttatcctcctcttcccccagctcaaaatctacaaagaaattatgtaattcctgccagatgataacagttagctcccccaccaagtcgcgatctgaaatttaaacattcccaacccacctcaaatacagttgaaactgacattcttcctgaaggttagattatagaggcgattgaatttttacaaagcacaatacctccaaaccatcctcccagaaatgtgttctttgaccattaccaactttgatttctatcattacataggttcactctccttggaaaacaaattacaatagttagaaactccaagaactagatggtgctggtcaacagtcattttatttcaagaaggttcaaaaacggaatcaagagttcatttccccctatagtatgcaaaactgaaactatacattcacttatgttatacacacaagctttacaattcaagaggaaaaaagactgtctataaatcatggttgcaaagagtatggaattagaagtacagggaaacacacacgaggtactttcatccggtactgctatcatgtcaatcttggtgtttgaagccattctgtggtggcaatggtggttgttcctgcaagtcctgctgctcctgtggttgtaaaggtgggtgcaactgatcctgtccctcttgctgttgcaatggtggttgcaactcatcatgtggctgctgtgttggcgaccgtgactgatgttgctccccttgcagaatcggctctgaccgatggtgctgatcaccttgccatacccatacaatatccttgagtgctggccttacttcctctttcaccagcttttgatactccatagtgtctccattagtctttttcaactcaactaggagaaaagaaggggctacctcgaaaatctcggcatcaagctcgagaaaccccttctttccttcctttggagcagccaacttcaggactccattttctttcttcttaatttttagcttcaagcgtttggccagttccttcaactttgtaagtactattgttacaggctgcctagtggtaaaccgttcttccctgcgaccatactcatcctcaaaaaacccagatagattgaaccctgttgacagagagatgatgtcaaatgcattcaggtttgggaggcttaatgacccttgatttccctcagaagtgctgcatcctgtcgagcctggcgacgcagcttcagctgaaaaggtgttgtgctttgcttcaggctcggttttctttgcatttacctcaactggtcttctgtaccaagcacttctctttatcctagagattgtaatcctagtactgggatctggatcaaggattttatgtaatagctccttcagctcagcggaaaaataccgagggcatttgaagtccgctttggaaatccttctgtacatctctatcagatttgcctcatgaaaaggaaggtaaccagccacaagcacaaacagaattactccactggaccatacatctgcttttgcaccatcatagcctttcctgctaagcacttcaggggcaacataagctggagttccacatgtggtgtgcaggagaccatcctgcctcgcgcaatcagccagggcactcaaaccaaaatctgagacttttaggttttcattctcatccaggagtagattttcaggcttcagatcacggtgataaacacccctgctgtggcaatagtcaatagcactgatcaattggtggaaatacctcctagcagcatcctcattaagcttcccctcctttactattttgttgaaaagctcaccacctttagcatactccaatacaaaataaatcttgctcttggtagccattacctcaaaaagctgcaggacatttgggtgcttcaccaatctcattactgaaatctccctctttatctgctcgataagtccaaccttggtaaccttatctttattgatcatctttatagcaacagcttggccagttgcaagatttcgggcataatacaccttcgcaaaagttccttgtcccaattgcttcccgatttcataacggcccatcaagatagtcctcctctcatccatcatatcaagctatctgaacggagtaaaacaaacgaacaccacacacaccgcttatatagcagtatctagactgtatacctcatgaatggagcagatgtcaactgtcttcacaaatgtgattatcaacaaatactgaccccttcggtatactgtgcttcacaccacagagaggaggaacagtaccagtatgcagcacattatcatgagagccccacagcatgagcttgaacgaagctctgatcttgtttgcacaaagtaagtgtttgctgttacgccagcacatctttctgcattcccaagtcttcttccggtgaacaactgccagccagaagatcagattaaatcataacggatacatgtaatgcagggtgggattacgagctctaatgaaaaaacacaaattctgtggcctttgtggacattaaggcaaaatcttgggtgacaccccaatgttgtctatcaaactgagatggtacagaacaaacaagagcatccgccaaagctggataactgggtgaagcaaagatgccatctcttgaactctagaaacgattatttcgacaattctaactcaaaaaccgaccattcctgcaacaggcatccaaaatcggcaaaattcacgcccaggacggggctaaacagagcgctgccagcaagtagatctcttcctgtcggaagaacagtacctgatcaagaacgggagaaggaagcgccgcgaatcccacgggacgccatagaaatcgaccgggatcgatcccgtgaagcaggcgggcgccgatttggtgcgcgtttggccgccttgcaagcttgctgttggtggattctgggatttcctggctgtaggtgccgcgctctcatactcttcctcctctgggatggcgcttagccttttgtggctagatccctcggaatcttctaatttcggtcttagcgatgattgtagcccggcggcggcgcgcttctccagaatggagggagtgcagcaggtttggcagccgtttacgctgccgtgttgac

>TaCIPK11_3B_10507835

aaaaaagactgtccataaatcatggttgcaaagagtatggaattagcagtacagggaaatacacacgaggtactttcatccggtactgccatcatgtcaatcttggtgtttgaagccattctgtggtggcaatggtggttgttcctgcaagttctgctgctcctgtggttgtaaaggtgggtgcaactgatcctgtccctcttgctgttgcaatggcggttgcaactcatcatgtggctgttgtgttggcgacggtgagtgatgctgctccccttgcagaatcggctctgaccgctgctgctgatcaccttgccatacccatacaatatccttgagtgctggccttacttcctctttcaccaacttttgatactcgaaagtgtccccattagtctttttcaactcaactaggagaaaagaaggggctacatcaaaaatctcagcatcaagctcaagaaaccccttctttccttcctttggagcagccaacttcaggactccattttctttcttcttaatttttagcttcaagcgtttggccagttccttcaactttgtaagtactattgttacaggctgcctagtggtaaatcgttcttccctgcgaccatactcatcctcaaaaaaaccagatagattgaaccctgttgaaagagagatgatgtcaaatgcattcaggtttgggaggcttaatgacccttgatttcccccagaagtgctgcatcctgtcgagcctgacgacgcagcttcagctgaaaaggtgttgtgcgttgcttcaggctcagttttctttgcatttacctcaactggtcttctgtaccaagcacttctctttatcctagagattggaatcctagtactgggatctggatcaaggattttatataatagctccttcagctcagcggaaaaataccgagggcatttaaagtccgctttggaaatccttctatacatctctatcagatttgcctcatgaaaaggaaggtaaccagccacaagcacatacagaattaccccactggaccatacatctgcttttgcaccatcataacctttcctgctaagcacttcaggggcaacataagctggagttccacatgtggtgtgcaggagaccatcctgcctcgcgcaatcagccagggcactcaaaccaaaatctgagactttcaggttttcattctcatccaggagtagattttcaggcttcagatcacggtgataaacacccctgctgtggcaatagtcaatagcgctgatcaattggtggaaatacctcctggcggcatcctcattaagcttcccctcctttactatcttgttgaaaagctcaccacctttagcatactccaatacaaaataaatcttgctcttggtagccataacctcaaaaagctgcaggacatttgggtgcttcaccaatctcattactgaaatctccctctttatctgctccataagtccaaccttggtaaccttatctttattgatcatctttatagcaacagcttggccagttgcaagatttcgggcataatacaccttcgcaaaatttccttgtcccaattgcttcccaatttcataacggcccatcaagatagtcctcctctcatccatcatatcaagctatctgaatggagtaaaacaagccaacaccacacaccgcttatatagcagtatctagactgtatacctcatgaatggagcagatgttaactgtcttcacaaatgtgattatcaacaaatactgaccccttcgatatactgtgcttcagaccacagagagaagcaacagtaccagtatgcagcacattatcatgagagccccacagcatgagcttgaacgaagctctgatcttgtttgcgcaaagtaagtgtttgctgttacgcaagcacatctttctgcattcccaggtcttcgtccgatcaacaactgtcagccagaagatcagattaaattataacgtatacatgtaatgcagggtgggatacgagctctaataaaaaacacaaattcagtggcctttgaagacattaaggcaaaatcttgggtgataccccaatgttgtctatcaaacttagatggtacagaacaaacaaggcatctacagcaatcagagtcagcatagttggataattgggtgaagcaaagatgccatctcttgagttccagaaacgataatctcgaaaactctaactcaaaaaccgaccattcctgcaacaggcatccaaaatcggcaaaattcacgcccaggacggggcgaaacagagcgctgccagcaagtagatctcttcctgtcagaagaacagtacctgatcaagaacgggagaaggaagcgccgcgaatcccacggaacgccatagaaatcgaccgggatcgatcccgtgaagcgagcgggcgccgatttggtgcgcgcctgaccgccttgcaagcttgctcttggtagattctgggatttctgggctgtaggtgccgcgctctcatactcttcctctgggatggcgctcggcttttgtggctagatccctcggaatcttctaatttcgctcctagcgatgattatagcccggcggcggcgcgcttctccagaatggagggagtgcaggttcggcagccgtttacgctgtcgtgttgacgatgcgggctttttctcattttgcacccgaccttttgggatttcaccccattagaggtttggttctttttttggagacaaattagaggtttggtgaggatctactagcgacgaactaatcatactctgggctggccaagttttgggctaggctttggaaagcccaatgtggaaacaccaagcccgagcctggcccgaagtacttgaacaatgctttatttatttaatttgatttgtctcaaaaaaaatttatttaatttgattcacctaaaaaaaattaatttaatctcttggctattaaaaatacctatatatattttaaaaaattagtcaggggtgtcgtcccttatgggggcattttccctgggtgtggaagcgcttcaagtggtgcgtcccaatttcgtcatatgtcgcatgtggacgcacctccacacacgagcctgtttttttaatgttttgtttgattttttttctataaaagaaattgatttttgggatttgctcatttttatgagctgtcttaaaaaatttagggtttttttgggatttttcttaccttttaatttctcggattttcctgtttttagagcacatataggttgcagtggaggagtgtatattaattcgcgtttgctcatgtaggaagcacaatagtgtgctagtttggagcacatacacataggaagcacgtgttggtgcaagtttggagcaaagtttaattcacatagggagcccgggctagtgtcatttgggagcatagattagttcaggtaggaagcacaagtcaatgctagttgagacttgagagcacatgcacgtgagtagtacatgttgctcctacttctaggttagttcacttagagagcacatattagtgttggtttggagcatatattggaggcatgcggattagtgctagttggaaggacgcggcgtttcggctctcgggtgcatctgcactcaagggtgaacagtaacataaaaaactactagaaaaaaatcatgaaagatgttcgagtgtgtgatgtccgtgtcaaattcgtcatgtttgacatcacgcactcaaacatgttccatgaaaaaaatgatttttctgaatttttctagtattttttactttacagttcaccgggagcatatgagcccgggctcagaaacgaatattcatagttggaactacagcttggggagtacatattagtaatagtagggagtacatgtcatttcaggtaggagcacatgttagtgtagttgggagcacaaattttgcctaaaaaagttcgatgaaaactatcaacatggtatctagtttcgaagatgtcaacatgaaaaacacatagttaaaacggttcgtaatttagatgtgtgttttgagaaaaagatgtttaaaaaagtggatctacaaaaacacacgaacaaacccactcatccgtctcctgtagggggtatcccactgaaatttccagatgtcgacaagtggagcacctgcggtgtacaacttgtcgccaccagcagagatgagaagtgacctttgtaaagtgtacccctaaactagtgatttcgtaaataaaaacataattttacgacattttgggttgagcttcggatggaaaagttgatcccaagaactagctgagatgatgtgctttcatacctatattacccatgctcaggtttacatccaacggctagctgctgaggttgtgtctaaacttgtggttcaagaaaaaatatgatatttgttgtacagttcagtgtgtctttgaatcaccataactgacattgcatgtactctccacaacatagtacgcataaaaaacgtgtcaaacatcttccatccatgccgctccaacatgggacactaaggtcttcagaaccaaaatcataaatgcccatcaatgaccctactcgtcgacgatgtagcgggcctattgtcaaagcaagaacatggcataggggtgaagcgatcgagtgaatgcggtcagtgatgcgcttatcagtgacccgattgttctccttctctatggctcgaaggtgctattctcacaacttggtggcgcctcctctatcgactgatccgtcatcgtcggtgttttgccataacacaatatcccccagatttcataggtggttttgtccccatcaacatgacacacacacacacacactgttggaaatatgccctagaggtaataataaaatgattattattatatctccttgttcatgataattgtctattattcatgctataattgtgttatccggaaatcgtaatacatgtgtgaatacatagaccacaacatgttcctagtaagcctctagttgactagctcgttgatcaatagatagtcatggttttctgactatgggcataggatgtcattgataacgggatcacatcattaggataatgatgtgatggacaagacccaatcctaagcatagc

>TaCIPK11_3DL_6897880

tgttagtagtgatataggtaaagaagaacttaataaaaaatatgttcactccacatccacatatgcatgcaaaatacatgataaggcatattccatgtagcccacataggctctaaacaaacaaaaaatatcataaacaaactccactcaggtcaggcttgatactgggcggactcagagtcaactaaatatggcacatatcatatgcaccataaaaagcatcaatattgtgccagaaggcttcagtccattacgctacacacgggcaccacatcagtgtcgcgcagtgatagtagtaatgtgatgatgcaaaacttattaaaattgcattgtcacatatatccatgccaccaaaaccaaagatgtcatataaaattcccaaagaaaatatatacatatcctatatgccaaacgtaggataaaatgatgcctagccaaaatcaaccatcattctgcaccatcatataaatgaggttgaccacaactatacatgctaggaaaatatactccaacatgtcaaacatgaaatcatggaggagtatttgcatgaaaccaaacttcaagttcaccaagaaggagttcatcgtcgaccgcgaggaggatttctctcgtagaatattgtaataggcatgtgatgtaatcctatgtaatagatatgttggaattcttaatcaggggttttctcttcggagatgtaattaacagaatttttatgtaaatgtaagagttctggaaataatgtacctgcaatgtttgattttatttttattgtatgcatctacacatttaaatttattcactctgtatcaatgacgtggacacgtgtctcacgcccgccattttcccgtcatcagttatcttcattttttctatagttttccttggtttttatttttacttattctttttctttctttctttcctactttggctttcaccggcttccttcattattttgctttttcttaactcatgtcaactattttcatacacattacaaaaaaatttatacataggtaacaatttttatgcatgtgtaatagtttataaatatatattttgatgtctacttttcgaatacacactgtaaatttttcatatacatcagaggattattatatatgtacatatttaacatgattaacatttttttccaaattcataattttgatatcttttttctaatacatgtcaagcatttctcaaatgctcatttcaacatttcattttaactatgcgaacattttttgtacattctataaacattttattaaaatgtcacaaacatatttttgaaatatgtgaacattttttaaaaaaataatatgttttttgcgaaaaaggatcagatcttataaaaattcgccggaagtatcaaacataataaaatttacatcaaatttccaagaccgcttaaagaccactattgtcgccagcacgagccactgacgcgtcattgtcgccgctcctctaccgaagtcagcttgaccttatcaatgagagctgggaaggctttgtcatgtgcccctaaggaccaacgccctggagttgtagtcgtcgctgataaatccttgcatatatctgaagcacctgacacaaattgtcaccacatgatgagaaaccctaacgtcacaaccccaaggagacgacaaaaatttgtgtcggagctccatcgactacgtccagacggacgaactcgaggagaatcgaagcccaaaagataaacttgaagaagaagcgccgccatccgcctgagtgccgcacctacgaggaataaaaaaccctaacctaaactacaaatcaaagcgaaggcaccggaattcccatcacgaccaacgactgtcggagcagcaggcaaagaggaggcgaatcgacggactcgtcggtgaagcctggaggggagagtgtgccctaaccgcctagggttagggaggagaacgagaggaaatcacgtcgtatatgtctctgtttcggatgttaacatactttttttgaatggtatgaaacattttttctaattacactgacgttttctaacattgtataatattttttaagtgcttgaccatctttgaagtgtaaaaaaaattgaattctataaatatttgtaaaaagtacactatcggtttttttatatgtgttaaaatttccagtaactttatgtttatacacactaacgttttccaaaatgtaaataaacaaaataacgaattaaaaaacggagcagaaaaataaactaacatgcaggtaaccactcctgggacggggccacttttttttatttgtctttttattttctaatcttgttttttcttcttttcaaaaaattagaaattgaaaaaaaggcccaagtttaaaaaatgtttggggaataaaaaaaaagttcatgaatttcatgaaatgttcgcaggttcaacagagatgtttatgaattcaaagtatgttcatgaatttagcaaaatgtttccaaattaagaaaatattggttctaaaaagaatttccgttttaaaaaatgatcgcgatttcaacagaggttcgtgaattaaaaaaaatgatccaaaatttcataaaatattccgtttttttcaatttcacaaaacgtacgtcatttcaaaaattgtttgtgaactaaaaattatttgtaaatttcaaaaattcttctcatatttaagaaatcgtattcatggatttgaaaaaaaagattcctgaattttttaaaaaatagaaagaaacagtaagaaaaagaaaaaacaaaaaaaacaacaagatacaaaaaatgaaagaaaccggttgtgtgcggtggcaggagggtacgcgttgcctggccatccaccacggcatgcgcggtagatattcctaatacgtctcgcctatagcgcagttttacccggcgggcctaataatctagtttgccttttttgtttgatttttatcggtttttcactgttttgcattgattttctttgagttttcttcggattttctatggtataatttttttactttttacttttttttgttggttttctttgtttctttcttgggaaacactcatcatcacccgattgataaccacgcaagcgtctgccgccttcgtggccgtatgatttgatcgaatgacccgagcagcagcctatttacatagtgtaggtgtgcaactaagccattgttgcaatctcgcccactgcgacaaactaggttgttgcaggatttgaatctcatgcaacatgattgcctcaacgtgttcttgtggggtcatttgacgatggggaaattgcaacaacatctttcatcgcattctgcaacatcatccatgttgcaaaagtcatttccacaacatcatacatgttgcaaaaagtgaagaaaaaaaaactgcaacacaacctctgttgcaattattttctgcaacatgagcccagttgcaaagagttctacaatattgtctttgttgcaatgataaggacgtggctggatcattggattatgccacatctagctgttgcggaggcggcggatcttttagaaagatccgccgaccgacgcgtagcatggcccttcttttctttggtgtcactagttttctttaggttgtttcttttccgtttcattttttgcaacacatgttcaattttttcatacacattttatattttcgtatacgccaggaatcttttattatacatgtttaatattttaaaaataaatgataaagatttgtatatacatccaaaacatttttgtataaaagtttaatatttttcaaatacatgattgacattctttctaacacatgcattgaactgtattttaatatgtgttaaagatttgtttttcaaacagagattgaaacatttctttaaactacatgtacagtattttatcttatataagaatttatgtaaaatgtctttttttaaactgtacattttttaaatgtaatgtacaatttgtttgaatggtcaattgattttgaattatacgtcattctttacatttataaatattttctgaaaatgctacgtatattatttaaagcagtgaacatgttttaaatgtcaccaccatattttttaaagctatcaacattttgaaaaaatgacgcaaagaaaaaaaaactatgttaacattttcatattcggggttttcaattttttgaaaatgaattcagttttgaatgtatgtatttagatttttttaaaaatataaaaaaatataaaatacaaaaaagaaatgaatgaataaaaataaaagggaaatgaactaactaggaagttcggtcgacataatactagcgagtgtgtgggcgctccttcagactagaccttgttcggcgaaatcactaattgaggagtactccttgcaaagatcactcccgccttttcaggttgtgacaagtgttgcgctgcatgtgcgccacttgttgcaacctgggagtttttccttttttctagatccgttaattcaaaatgtttttatctcttaaaccgtgcgttcaaatctcgaactgttttcaccgctggattcctcgcgtcgagattagggatggcacccgcagggtatgggtacgggtggagccaccccatatccttacccgtccaccctaaacttacctatcacccatncgcagggtaggggtgcgggtggagccaccccatatccttacccgtccaccctaaacttacctatcacccatacccgtacccgtcaaggggacaattttttcccatacccgtcacccgacagggtatatgggtacccgcgggtaaaaatactcgcgtttacaacacatcaattgtgtaaaaatagttgtaaaatgcaacatataatcataaattattaatagcaacacattgtaaacaacaatgtacaacaacatattgtaacaacaacacattctcataaacaaaaatacttgcctataaagcgacacataataatgttaaacaacaactcataatcataaataacaagacataggcatacattttaagttcacaaaatcatgataaattacagagataatttggctacacattagagtttttacctagcgcgattaggaggggaaaattaataagttaggatattaaagaaaacgcacttgccaacattttagatgggcacatgagtacgcgctcatgggttatacaatcccatacccctaccctctctacccaatgggtatgatattttcgcatctaagtacccatgggtaaatttttgtctcatacccttaccttaatagggtttttacccgcagggtacgcggataatgggtacccattgccatccatagtcgagatcttcaaaactagttcccatgttgataggttttgagaaactttttttcacgaaaaaaccggacgaaaaaactgaacgaaaaaaccgaaaagggagcacaatttttccctttccgaaagaggcacagctgtgcctctcgcgaaagcaaagcagtgcctcttgaggaagcaaaatcgtgcctctcgcggaaggaaaaaaactaagaatgcatttttttcgtttccgagaggtacggccgtgcctctcgcggaaggttaaaaaaacgaaaaacacgttttcttttcatttccgagaggaacggccgtgcctctcnnnnnnnnnnnnnnnnnnnnnnnnnnnnnnnnnnnncgaaagcaaaaccgtgcctctcgtggaagcaaaaccctgcctctcggaaaaaaaatagaaacatattttttccgttttcgagaggcatggatgtgcctctcgcaaaagcaaaaccgtgcctctcgcggaagcgaaagaaaagcacacaattttttttcgaattacttttggtccaaaagctaaggaagacaggtggaaaaccataaaataaaaaaaaaacgtttaaaaagccgaaaacgcatacggaaaaataaaataaaaaacaaaattcggagggagcgcgcagagcacgacacgtggcgacagctgaaagcgcgctcttagcccacccgcgagtgatcgttgcgaggctcccgaaatggttgtttgggtagcgacgggattatagaactgggttttagccgatttttagtaaaatcggttttatcaattgttatgtgcgtaaccagtttatgaaaaagatgtttggactagaaaaacatagtaccgatttgtcacgtttcttgtttggtttggatagacttttggcccccatgcatcaatcgaagagtttgttgaggaaaagacgggcgaagagaaaaggttaacgatcgagtatatgcagatcgcgttcgtagaaaaacaactaatcctcgtttttccatatacctaaaatcttgggttttgtactcactaattttttttatttctagctttgtcgaccattcacacagggagctactgttttttttacttttttgtttcttcttctcttttttctctgaaaatatcttgaaaaatagaaatatttatttttctagaaaactatttttagaaaaaatgagtttttaaaagggtcacatagttccaaaaaaataaaaataaaaattcatgttcagattaaaaaatccataattatttcaaaagtcgcgttccttaaaaatgtttttaattagcttttaaaaacaaaaaaaattagaaaaccaaacaaaaacacaactaagaacccgctataaacagtaataagaaaaatatcgttccctttcgtctgagttgctacagtcccaacgcgaaagggtcggcctagtcacggcgcccctgtgcaattggccatgacacacggtcttgtgcgtttgctccgtatcccgacgcagagagctttgacgccttaaggagacctcctatttgccgctcactacaacgtagtacaagaccagacattcacaaatatgtgcatacactcacccctatgaatacacgcacgcactctaattctatgagcatcttcgagaaattgagccggccggtcttgagattgagaagtcaccacatatccctcgctatttgcacgaatgtcaatgtcgcctctcagttctcactgaaagaatattctgcctttatgagacctaaacgtcaaacctgaagtttgatccttgatgggttgggaatagcaccgcttctctaaccatccaacaaaagattagttacttggttctcctcatgggccagcctattactttgacaggaatggcatgatcattgatcgggaagcaacagcaaaccactaggacatgcggctgttatgagaatcgcagatgcggcagccaatatgatctgtgaaagcatcatgtcatgcttcactgtcttctccgttgctcttgttcgctctgcgcctcggatgccatacgcatctgttcaagtggcaaactcacgagctagttataattatcctcaagaaggcttacttctactcccttcgtttcataattttttttttaaaaaaggttatccccggcctctgcatcagaacgatgcatacggccatattattgataagcaaaaggttcaaacaaagtcttcaggtctcaaacaaggaaaaaggctcacacagagcataaacaaaatagcgggataaccacaaccggcagggataaaagaagataggaaactaaacacttatcctattacatgaccgtcatccaaaccggttgaagatagcccgtgctaccgtctcccacccgatagacccagtaaccatacgctccctggcatccgtcggagtgagtagcgatcacacaggatcagtgcagtgacgcgaaaaataacctgcaagaaatgaatatttgttattctgttaaaaaccaaaacgtttctgcagttccaaactgcccataataaagtacatactcctatacggatatgtctcgccgtttttgaatctatgccatcaagccaagtcccaaataacatgttgataatattcggaggagtaatattaaaagctgtggacagatcgccataatttttttgccaatgggcagtcaagaaagaggtgtttgatagattcatcatgatcacagaagctacatcttttagatcctgtcgagttacgttttgccaaaatgttcttagttagaatgacttgtttatgtacaaaccacatgaacactttaatttttaaaagcactatgactttccagacatgtttcaatcgaggaatagagcccgtgttgataatatccaaatacatgaatttaactgaaaactcaccattcttaattagtttcataatgtaaatattttttgacactagtctccgtcccactagactagtgtcaaaataacgttttacattatatgggacggagggagtagtagttagcatgggtcttgaatcaaaagtcaaaaccgattagatcagtcattagacttctcggaatcaaggcccatcgcgcccagttttcatctccgaaaactgataaaacaacagacaagccggcctcacgttcaggtcctgtcacgaacccacaagaaaaccagtctcgaggacagccactcggggggtaatcgtaatcggaacataacgcagcagcaagctccagctaccaaagccagcaaaaaacagcagccagagcttattactaccagacagtagcagtaactcacaaacatcaaaacaaggctaatcggaaaactttcatcttggcgtgagctcctccttgttcttttttccatcatcctttttgctggcgtgtcttgtcggtccttctacatccgcacccaacctctacgtctgctaaaaaaaacccagctgccgcttgcaaccaagcaaacgcatttatcctcctcttcccccagctcaaaatctacaaagaaattatgtaattcctgccagataacagctagctcccccaccaagttgcggtctgaagtttaaacattcgcagcccacctcaaaaacagttgaaactgacattcttgctgaaggtcagattatagaggcgattgaattttttacaaagcacgttacctccaaaccatcctcccggaaacgtgttctttgaccattaccaactttgatttctatcattgcataggttcactctccttgggaaacaaattacgataattagaaactcaaagaactagatggtgctggtcaataatcattttatttcaaaaaggttgaaaaatggaatcgagagtttatttccccctatagtatgcaaaactgaaactatacattcacttatgttatacacacaagctttacaattcaggaggaaaaaagactgtctataaatcatggttgcaaagagtatggaattagcagtacagagaaacacacacgaggtactttcatccggtactgctatcatgtcaatcttggtgtttgaagccattctgtggtggcaatggtggttgttcctgcaagtcctgctgctcctgtggttgtaaaggtgggtgcaactgatcctgtccctcttgctgttgcaatggtggttgcaactcatcatgtggctgctgtgttggcgacggtgagtgatgctgctccccttgcagaatcggctctgaccgctgctgctgatcaccttgccatacccatacaatatccttgagtgctggccttacttcctctttcaccagcttttgatactccaaagtgtctccattagtctttttcaactcaactaggagaaaagaaggggctacctcaaaaatctcggcatcaagctcaagaaaccccttctttccttcctttggagcagccaacttcaggactccattttctttcttcttaatttttagcttcaagcgtttggccagttccttcaactttgcaagtactattgttacaggctgcctagtggtaaatcgttcttccctgcgagcatactcctcctcaaaaaacccagatagattgaaccctgttgacagagagatgatgtcaaatgcattcaggttcgggaggcttaacgacccttgatttccctcagaagtgctgcatcctgttgagcctgatgacgcagcttcagctgaaaaggtgttgtgcgttgcttcaggctcagttttctttgcatttacctcaactggtcttctgtaccaagcacttctctttatcctagagattggaatcctagtactgggatctggatcaaggattttatgtaatagatccttcaactcagcggaaaaataccgagggcatttaaagtccgctttggagatccttctatacatctctatgagatttgcctcatgaaagggaaggtaaccagccacaagcacaaacagaattactccactggaccatacatctgcttttgcaccatcataacctttcctgctaagcacttcaggggcaacataagctggagttccacatgtggtgtggaggagaccatcctgccttgcgcaatcagccagggcactcaaaccaaaatccgagacttttaggttttcattctcatccaggagtagattttcaggcttcagatcacggtgataaacgcccctgctgtggcaatagtcaatagcactgatcaattggtggaaatacctcctggcggcatcctcattaagcttcccctcctttactatcttgttgaaaagctcaccacctttagcatactccaatacaaaataaatcttgctcttggtagccataacctcaaaaagctgcaggacatttgggtgcttcaccaatctcattactgaaatctccctctttatctgctccataagtccaaccttcgtaaccttatctttgttgatcatctttatagcaacagcttggccagttgcaagacttcgggcataatagaccttcgcaaaagttccttgtcccaattgcttcccgatttcataacggcccatcaagatagtcctcctctcatccatcatatcaagctatctgaatggagtaaaacaaacgaacaccacacaccgcttatatagcagtatctagactgtatacctcatgagtggagcagatgttaactgtcttcacaaatgtgattatcaacaaatactgaccccttcggtatactgtgcttcacaccacggagaggaggaacagtaccagtatgcagcacattatcatgagagccccacagcatgagcttgaacgaagctctgatcttgtttgcgcaaagtaagtgtttgctgttacgcaagctcatctttctgcattcccaagttttcttccaatcaacaactgccagcaagaagatcagattaaattataaagtatacatgtaatgcagggtgggattacgagctctaatgaaaaagacgaattcagtggcctttgaagacattaaggcaaatcttgggtaacaccccaatgttgtctatcaaacttagatggcacagaacaaagaagagcatctacagcaatcagagccaccatagttggataattgggtgaagcaaagatgccatctcttgagttctacaaacgatgatttcgaatattctaactcaaaaaccgaccattcctgcaacaggcatccaaaatcggcaaaattcatgcccaggacgcggcgaaacagggcgctgccagcaagtagatctcttcctgtcagaggaacagtacctgatcaagaacgggagaaggaagcgccgcgaatcccgcggagcgccatagaaatcgatcgggatcgatcccgtgaagcgagcgggcgccgatttggtgcgcgtctggccgcctt

>TaCIPK12_1BL_3828145

agggatttgatctatcagggctgttcgaggagcgagggagcgaagtgagattcatctcggcacaacccatggaaaccattgttacaaaattggaggagattgccaagatgaagagcttctccattcgccgcaaggactggcgcgtaagcatagaaggcaccagggaaggggagaaggggccattgacgattggggctgagatatttgagcttacaccaagcctcttggtgttggaggtgaagaagaaggcaggggataaggcagagtatgatgacttctgcaacaaagagttgaaacctgggatggagcctctcgtgcaccaccaatctggttnnnnnnnnnnnnnnnnnnnnnnagttctaaaggtagctctcttgcttgaaaggaatataaggaaattttggattgaaaggatgcgtcttttatatgtttattaagcatgggacctgagcagaaaaacgctattcatattccttagtcccttttgtgttagtattattcatttttgcaatccagaatttttcatgctagcggttactgtgtttgttgtagttgttacatacatacagtctgaagggagttgcggatttcgctttcgccacattatcaatgtttatcctagctcaagtgctcacgttaaattatgctttattcataataatgtaccctctgtaattaattcatctcttaaacataaatgtaccattggaactttcgtgagtgctagagacttacgagtattatagctgttgtgtaacttgtggattattttctttgctttcatcaaaaaagtatcacatcataagcagaatggccattggctcgctgatgtccagcttccgtgatatactaattcagaccatgctgttattaggtcaaagcaaaataagatgtgatgaagtgatgctctactgatgatggtttagttgaacaaaactttgaaatttgaatctactctatagatcaactcggctttgttatcttctgaattagtacaaaatcgtgttatcctctctctttcttctgagtcaagttggtcttgacagaaaccgaccagaagcgaagggctgcaaacaattcaattcctgttcctgcaaagtcacaaacatattgctattcagaaagaggtgcggtaagttgcttgtatattctaataatcgtctattagttccaaatctgttggtccagaaaaaggaaagacaaactgaagcttagtgcctattgttttttgcttgcaagcattgcaaatgaagaggaccaatggttctgtgctcctgtatagtcgtgtatgcgagaatgggtaagacacttaccatgtaccatgattcaggagttgggatgggacgatatgacaggaggggcagggaggattgtgtgtaggttggaggatggtcaatgtaggccactggggcttaagattggacactgctatatgaataaagttagatcaggcatcacactgaataaggtgaagtttaggggtcaggatataagcatttactaaatattttatattgcatatgcctattcctaatctattccatgtacatcagaggttgatatgttatttcagaggaggtacaattaagtacagtagcttccaagcaactggacacttgataagttttcttatgtgatcatatgaaatttgacaacataacttaattgttattataatataactaatataagaacagtatggcgtgacaaattacaaaggggtaatagctcaatcaggccaagaattgtgacaaaagatctatatttagctgaatgcatacagttgtcacactgaataaggtaaagttggccaggttaggggtcaggatataaacatttgcagatttattgaattatttgtgttacacatgtctattccaatctacttcatgtacatcaaaggttgatatgttatttcagaggaggtgcgacaagcaccttccaacactggagtaataggagctcaatcatgccagtaactgtgatgatgaatcatagctgaataaaaaaaaggtgcagcccggtgcatgtagctcccgcttgcgcagggtccgaggaagggtccgaccactttgggtctattttatgcaacatttccatacattttctgtgagaggctgttgctagggaatggtagctgaaatgcagtgaaagaaaaaagaatggcatgcttaatttatgatcttctattatggatgtgttcttatccagttttgaagatgtacataccggcaaactgatcgcgtggatacctactataccgccggttcttgtttattaattcactccgtcaaataaatttacaagtattcccgcaaaaaaaatatttacaagtagattgattgggaaattgcaatgtggcgtgaaggaggggaaatgcttcaaaaataccaatgttgtgcataacagaatgcgcataaggttgtcatgctgactaccttttcatgtttattaaataacaaaaatgttgatttttttttctctctctgctccttaaggctggctccacatcaacaaggcatgccatctgttgccgctctttgctgaacaaggatctaggctgcagttgccagttagaaaggctaagcgctggcaatcacataaagacataggcgtgagtgaaatcagtggccttggcagtacatccttggaagtcgctgcttcgctgaatcgtttgacagttgtgtttggtgactattatcctattaatcttggattgtgtttatacttagtttacatgaggctcatgtttcctgtaaattcttatcccattctatgttcatccataggagaagtgctatacacacgacgatttctcgcccggtacatgcacaacactgagccgtgtcctttgatcgaacatgagaagcgtcgggttggtatcgggcgtggaacgtcgtgtgtgaagcaatttcgtatccatattcagttattatttcgatggtgatcgagggatgcggattgatttgaaatgtctggcagactcttaatctctgttatctaaaaatgtgaaaaatgtgatgacaagaagataagatgttgatttggtctttcatatcatacataaaacgggtctgacatcttgttattggttgcaggttggccggtttcagtcacacagagaagtttctctatctagttgcgctaccactggcgttgtctctagagtttggttgcctagaatggtacccgccaacgctattggtaggaaggattttgacttggaacaccagactataaatagtcagcttgaatttggttgcatagaaatgtcgttctgaagcaagaaagggttcgattcaaaccggagaaaaagtcagattcttgctgtacaacatacatggtggtctagtgactcttaaatcaccctcaaacgtccgtgcgaccgggcgcactttgtcatgcaacgcggtacctcctctattccgtggacgtgtctggttgtccgaaatcacaaaaactggacgcaaactaggggggttttgcaggcatccagaccgccttcacgtacatgtcggacaccctggtaactagtgatgctcgatggcgatagggtgtttgttcgaccaattcgtccttctgcacaaggactacgtctggttggaggggttgtgatttaacacagaagataaactctcttcgcccaattctcctcgacaattggttcgtcaaccaatgccgattactcgtggtagatacttgaggtgatcttcgaacctttacagacttccttcttcgaaaaccacaatcactcttggtctctgaaaaaaattgacacctaaccgtctagagagtttgcaactctcaagagtaataggtggagcgtactggacttagattcgactgatgctgaaccactatctaatttttcggcctaggggttttctctcggtggattttaaactcaaatcactcggagagggggttgctcaataatcttctcataannnnnncaagccgacgcggggtggctatttatagccgcagccttcctggataggaaatgaccattttggacactctgtccagccaatggccaaccgacacgttctcaacggtcggattttggagcaacgataacattacttatggagcaagtaatgctaactcctcggtctgagacaaatctctcgcagcgagtaatgctaactcctcggtctgagacaaatctctcgcagcgaagaagacctcagtctcttgctggcaagtattttctcggaacacaataggatttctctcgcaactttcataggatttggtttagcattagagaatcaaagttccgaatgttatacccctcttaatagtacggtg

>TaCIPK12_1DL_2112699

cgggcgtctcacacggccagcaagcctcaacgcttttgatatcatatccttctccaagggatttgatctatcagggctgttcgaggagcgagggagcgaagtgagattcatctcagcacagcccatggaaaccatcgttacaaaattggaggagattgccaagatgaagagcttctccattcggcgcaaggactggcgtgtaagcatagaaggcaccagggaaggggagaaggggccattgacaattggggctgagatatttgagcttacaccaagcctcttggtgttggaggtgaagaagaaggcaggggataaggcagagtatgatgacttctgcaacaaagagttgaaacctgggatggagcctctcgtgcaccaccagccgggctcggctcgaaatgtaccttctgatactgagtagttctaaaggtagctctcttgcttgaaaggaatataaagaagttttgggttaaaaggatgtgtcttttatatgtttaagcatgggacctgagcagaaaaatgccattcatattccttagtccctttgtgttagtattattcatttctgcaatccagaattttttatgccagcgtctacagtgtttgttgtagttgtttgtacatacatacagtctaaagggagttgcggatttcgctttcgccacattatcaatgtttatcctatttaagtgttcacgctaaattatgtttattcataatgtacaaatttcatgtgtagttaagtcatctcttaaaaataaatgtaccattggaactttcgtgagtgttgacttacgagtattatagctatgtgtaacttgggttatctcagctttcatcaaataaccatcacatcataagcagaatggccattggctcgccaatgtccagcttcagcgatatactgccattgtgtttgatcaaaacaaaataagatctgtggaagtgatgctctactgatgatggtttcagttgaacaaagctctgaaatttgaatctactctatagatcagcttggctttcttatctttgaaattagtacaaaattgtgttattccctctaattcttctgaatcaagttggtcttgacagaaaccgagcagaagcaaagggttgcaaacaattcaattcctgttcctgcaatgtcacagacatatgttacatattagtgttattcagaaagaggtgcggtaagttgcttgtatattctaataattgtatattagttccaatcttttggtccagaaaaaggaaagacaaactgaagcttagtgcccatcctttttcgctgcaaacattgcgaatgaagaggaccaatggttctgtgctcctgtatagtcgtgtatgtgagaatgggtaaggcacttaccatgtactccatgattcagaagttaggatgggtagggaaacgataggacaggaggggcagggaggaatgcttctagttggaggatggtcaatgcatgccactggggcttaagattgggcactgctatatgaattaaagttagagcaggcatcacactgaataaggtgaagtttaggggtcaggatataagcatttactaaatattttatgttgcatatgcctattcctaacctattccatgtacatcagaggttgatatgttatttcaaaggaggtacaattaagtacagtagcttccaagcaactggacacttgataagttttcttatgtaatcatatgaaatttgacaacttaacttaattgttataataatataactaatataataacagtatggcgtgacaaattagaaacgggtaatagctcaatcaggccaagaattgtgacaaaagatatatttttagctgaattcatacagttgtcacactgaataaggtaaagttggctaggttaggggtcaggaactcaggatataaacatttacatatttactgaattatttgtgttacatatgtctattccaatctactccatgtacatcaaacgttgatatgctatttcagaggaggtgcaataagcaccttccaacactggggcaataggagctcaatcatgctagtaactgtgatggtgaatggtagctaaatgcagtgagaaaaaaggatggcatgcttaatttatgatcttcgattatggatgttttttgatcatgtgaatacctactataccgccgattcttgtttattactccctccgtcccataatgtaagacgttttttgatactagtgtggtgttaaaaaatgtcctacattatgggacagagtagtaattcagcccatcaaataatttacaagtagattgattgggaaatcgcaatgtggcgtgaaggaagggaaatgcttcagaaataccaatgttgtgcataacagaatgagcataaggttgtcatgctggctatgttttcatgtttattaagtaacagaaatgttgatttttttctctctactccgtaaggccagctgcacaccaacgaggcatgccatctgttgttgctctttgctgaacaaggatcttggctgcagttgccagttagaaaggctaagcgctggcaatcacataaagacattggcgtgagtgaactcaatagcctgtggcagtgcatccttggaagtcactgcttcgctgaatcgtttgacagttgtgtttggtgactattatcctattattaatcttggattgtgtttatacttggtttacatgaggctcatgagaagtgctatacacacgacgatttctcgcccgatacatgcacggcgacaccgagcgtgtcctttgatcgaacgtgagaagcaaatctgacgatacatgacaccgtcgggttggtatcgggcgtggaacgtcgtgtgtgaagcaatctcgtatccatattcagttattatttcgatggtgatcgaggggtgcgaactgatttgaaatgtctgggagactcttaatctttgttatctaaaaatgtgaaaagctgtgatgacaggagataagatgttgatttggtcttcatatatcatacataaaacgggtctgacatcttgttattggttgcaggttggccagtttcagtcacacggagaagtttctctatctagttgcgctaccacctggcgttgtctctagaatttggttgcctagaatggtacccgccaacgctattggtaggaaggattttgacttgggacaccagactaaatagtcagcttgaacttggttgcatagaaatgtcgttctgaagcaagacggggttcgattcaaaccggagaaaaatacaacatacatggtggtccaataagagcatctccaacgccgactcttgaatcacccgcaaatgttcggaccaccgggcacactttgtcatccaacgcgatacctcatttgtccgtggacgtgtccggttgttcgaaatcacacaaactgaacgcaaaatgtgggggacatgtccaacaccctggtcccaaccaaaaacatctcccgcgtgaacaaacctccgctctccgcgcatgcgactgcttggattgaagcggtgtccgaccgagagtgccgctcatgctgcatcccgatcgatctctgcgcacattcaatgccagagagatgtgactggacgggacggctatctatcgcatttgaaccgggtgcccattcgtcagcctgctgacattaaacatgcgcggcgactgagaaaccaactccgacacctgcattacaaatctggtcgcatggactggctggcgctcgcaattttataagtagctacacccgacatgatcagcacaactccacgcctgcttcctttcctcctcc

>TaCIPK13_3B_10753103

ccccttgtgtcgaatcaataaatttgggttgaatactctatcctcgaaaactgttgcaatcccctatacttgcgggttatcagtccacgcggcgtcataacagtcaatggaaacacagaacgctcactctgcatggaggagcacatcgcggctctcgcagccgaagtatagaacacccccctcaagccgcacacctcatcggccatcaagccgctagactttgttaaaagagtccggtcagtctcgcaagctaactgctcggtacttccaaagctagactagtagtttcgcctccgtcgatcgccgcgcacacccacaaaatttgtaccacgcatgcatcactacacactcaaaataccttgggcatcaactgaggcacaatacagacaggtctatagtatggttcgaccatgtatgacctcccacctttctttgattacttttctctcctaccacaggtgcattcgagcccatatatcctatggacttgcaaggactctcttcgagcccggtttcgtgcgggtaaccaaagaccgttcccttcaaggaacccctctctacaaggcgaaagcggcgcagacgtgcggtaggatatccaaaggactcttcggaccaaaccttatttaaaggacctatattgagcttttctccttattattagacccctggcatgtaaaatgactttggtgcttatggtaccctctgtaagatcacgttttgacgtatcaatcagatcctagtagggacaattttctggtatcaactttattcataaaactgtactccgtctccccagttttctcacatacctcgacacccattgccaggggctctatacggccacatatgtgccgctaaacaaaagcccgaacaactttgtagcacactttggcgtcccgaacttggcattatatgcatgggctccgaatcatgtctttggtcaatagttgggttgccctgctcctgtgtttgctaccttaccttccgtttgttcggctaggttagtaaagggagaactactgcgattgtgtttctggttcgtccggtcaagcacctcagtagagaaagccgaaaactgactgtcatgatgcggtgagagttggtcagccactcggtgacttactaaatctttaacgatttttccgtattaaatgaaggatcgtttttctagtgatatacgtaatgcatccacattcggatggctgcatacgtaccaagggctattttatagccccatcgtcaaactcctatggctaagtgagagtggtaaagccgtatagtccaatttcctggttcgccgcactaacacctctgtcacggaccaagacgttgagtcaagagtgctcatgtgctatttcgaacacccccgtagcatctacgtgggggccgaagccgacgaatggaaaactctcaggagtacaaaaatggatgcacaggaagaaagaactttcagataatggcacaaatatattacaaagccttaatgtatataataaacacgcccgggtcgttcggatacattcattcgaatataatgtcctttgagcattgaccctctatcaagcgagcaccctctaggacgtcgtctaaataacgctccagcgtgcgctggtccttgccttcgggaggaccctccactgcgataacagcggccttcatctttgcccaatgcaccttgacgcaggcgaaggccatctgcgcaccttctatgcacactgaccatttgattgcatcaattcggggcacgacatcgatgagtcgccgcactaggccaaaatagctgctcagaacgggttcggccggccacggctagagtatgatctccttcatgtccgctccgtacatcttatgcaactcggcccactgtgccatttggtcgttcaacagcgtcgagcgctcctgcatcgcgaactgtgaccagaatagcttctccgtcgcatgcccctcctgggctcggtagaattacgcagcatcgacggtgctcttcggaagatccacaaaggtgtctggagaactccatagtcgattaagtaaggcatacctctgatcaccaaacttagactataaaagaaagggcttaccggccgttatctgcccggcctgtcgaatctcctcgcgagccgctcgtgactcggacctcgcctcttgggcctcctgaagggccttggcgagttcggccaccttagctttattctcctcctcgaggagctcgcacttgcgagtggcatccttgagctcttgctcaactttggacacccgctcctcgaactggcgctgggagacctattcgaccttcaaatcagcaaccgctttctcagcggccgcatcactcacccttgcctgctccttcgcacgggcaaggtcactcctgagggtctcgacctcaacaacgccatctgcaattatgcatatagcagcacgtaagcttcaacttagcatttgcaaactaattcaattgtatatggagtacttgaaacataccctacgcctcgtcgagccgcttattaatccagatattgtcctcgtcgaccagctgcagcttctgcttcagtttggaaacttcggcggcctgcgcggtcgccgctaacagtgaagcatgttttatcaaaaagataagtatgttattccctgcggatatctatagaccctctgctcgccctccttcattggccaaacagagtctcaggggatactatctatatacatgcagatcttttatatggaaagcggctaaaacattacatcacataccttgaagcttgttagcaggactgtgaaggcttcattcagtccgctcttagcggactgaaccttctcaaccaccatacccatcaaggtacgacgctcctccaagacggacgcactttgtagagcatccatcaacatgttcatcgcctctgcattaatagaggtcgctgccggaatagccgtctccccctcccgcggagggggctgctgattcggttccggagctgtttgtgtctctggaagagtatccggccgggggtcggataatccaggacccccgtcgccggtctccatgagggttgcgcccctcatgttctcggcagccaaggcgttaccctctggcactgtcttggtcccctcctacacttctccggagcctggagaaatcctttgggacgacacctcggtgtcgtccgccctaggaggcggggaagcttgtgggggtgtcttgctctccatcttctccggcgaaagatcccccgatgacgatgattctcgagggatgtcacgggccggactggaacgcgaaatttgatattattcctataatctataaaaagattagatgtatctaaggcatcttaaggtacttacgattcggcaaggggcttggctcggggggcggtgcttggggacagctttggtgcccggatttgaatcatccgagagggatatatttcccctcttggatgccccctcctccagatctatggaggccgccctctttttccctttgggaggagggttctcttcttcctcctcctcctcctcctcctccttgtcctcgtcttcgtctccctcatgagaggaaggagcctcggttcctccggacacggcacccgaagtttcttcgtgagggaggccaccttcggcctactttgctcttcttcttcttgtccttctttgccgacgcctggtagggcgccaggaccaacatcttcattaacaaaggctcggctgggttttcgaggagcgggaacggacatttaatccgctcggccttctttatccagtcctatttaggagatggaattttcaggacccctccctaagattaacaagctaagtaatgttcggaaatatgatacttactggggataccggattgttgcagtcgaggccggtatcgttggacgtctttggccacatcttctgcttcttgaagagcagctttcagatccctttgtgcgtcatgccaaagaatcgttgcagagtctgtggcccctccggattgaactcccacattttgagaggccatcgttggcaagggagagctcggcggatgagcatcacctgaatcacatcagcaagggtgatgtccttttttttatcatactcttgatacgcttctgcagtgttgacacttcatcaggggatccccaatctagacccttgttggtccaggatgcgagccacaacggaggtccagatctgaaagtaggagcagcagcccacttggtgccgcggggttcagtgatgtagaaccactcctgctgccacaccttgatagtatccacaaatctccctttgggccaggtaacgtggttgagcttgctcaccatggcgccaccgcactcggcgtgttggccttcaaccaccttcggcttcacactaaaaaccttgagccataagccaaagtgtggggggacgcggaggaatgcctcacacacgacgataaacgccgagatatggaggaaggaattcagggccagatcatggaaatccagcccgtagtagtacatgagacctccgacgaagggatggagggcgaacccaagtcctcggaggaagtggggaataaaaaccaccctctcactgggctctagggtggggatgatctgccacggggttggaagcctatgttttatctctgctgtcaggtaccttgcctcacgtagatctttgatgtccttctccgttacggaggaggccatccacttgccctgggaaccggatccggccattgctggagaagttggggcaaagggaaagtttagaacttgggccctggagctcggggacggaaaggcagaggaagaagaaggcgtgaggtgaacagatgggtccttgtcctcttatagaggtggtgaatatcaagtgccccctcataagccttaaacctcgcctattcccaaggatacgtgcgaacggcacggttggattacccaaacccgcattgatgaggaccccgtgataagggggcacgatctctgctttgacaagacgtgcctatgatcgtgcctcgaaacacgaagcgggggctgtaaaatggttcagaatactaacaagaccaggacataacgcctcgccagaaaaatcgcctatggatgtgacttactttgttttgagtatttttgcccttacagcttaagattgtaattattaaacagagttggatacaactatcatgtgcggaggactgctttggagtattcggagaaggaacctgccttgcaatgccgaagacaatctgcgcgccggatacatcgtcattgaagctcggttcaggggctattgagggagtcctggtctaaggggtcctcgggcgtccgggctatgtgatatggaccggactaatgggccatgaagatgtgagattaaagacctttccccatgttcggacgggactctcctttgcgtggatggcaagcttggcgtccggatgtgtagtttccttcatctataaccgactttgtacgaccttagtcccctctggtgtctatataaaccggagggttcagtccgtagaggcaaggacaatcatacaggctagacatctagggtttagccattacgatctcgaggtagatcaactcttgtaacccctatactcatcaaagtcaatcaagcaggaagtagggtattacctccataaagagggcccgaacctggttaaacatcgtgtcccctgcctcctgttaccttcgatccttagatgcacagttcgggaccccctacccgagatctgccgtttttgacaccgacagtggtggcttcacaagatctttgaatatgtcattctttcttcggcatggctggttctcacaatgatatcaacgtacatcagcgttctccaatgttcgcaaggtctgcagaaggccaatccccagaggtcaactttgagatcagtgaccaccattacaacaagggatactacatagctgatggtatctatccttagtggtcaactcttgtgaagacaatacccaacccacaaggagagaatagatagaagtttgcccaaatgtaagagtgctaggaaggatgtggagggtgcttttggtgtgcctcaatctcgatggggtgtcgtttgacaccctgcactgacatggagcaccgagaagctttgggaggtaatgactgcttgtctgatcatgcacaacatgatcgtggagtatgagcgtgatgacaacatttacgaccaagggttggattttcagggtgcaaaatgttagagcctgggcgccaagaaccggaaacgtttcaacagttcacccaattctatcatgaaatatcatatgcaacttcaagttgacttggttgagctcatgtggaatcacatcggcaaccagtaaatgtatcgattcatttttttcatctattgaagataattttgatttgtttgtaaaactatttgcaatgtttaatgtatttggttgtaaaagctatttacaatgtgtatagttcgtaaaatgtatacaatacatgggatccagcagttgtcgcggaccagatgcatccacccacggcaagcacattaaaaaaaaacagccggacgccgttctattgccctatccaaacaaaacaaaaattcagaccaaactagtgtctgtttgggtcgcgtcggagctcggctactaatttagtgatgccctgaacgcgctagtacatatcactaccctcccagtccatggccccacccaccattgccacatgccacatgctgccgatgcgaagcgggtgcggacggcggaggcgctgcggcttatcgcatttcaccggaaatgattcgcgcacaccaagggagaggcgagaggaggaaaattgcccgtgggctcgtggctcacgccgaggtatccagccacgatactcccacacgtccccgtcgcctcctctgtttcccaccacctctgcttctccggaagcccagacagcagcgcgggagctcctcctagctcaggatcggtgggagttgatacgaggcggaggagatggcggatgcgaagcagcagcagccnnnnnnnnnnnnnnnnnnnnnnnnnnnnnnnnnnnnnnnnnnnnnnnnnnnnnngcggcgcctccggcatgctcgccacctgcgtcatccagcccatcgacatggtcaaggtatgcatgcacggtggccggttagtggagctgcgcgctctgcttgagagttgggcagttctagctctacgttttcatttgcggaatgccgacggaacagtgggtttgaggcttgggccggggttgtgtctttgtaggcgagctgtttgatattttgcgtccaaggagagtgttgctccatttggttggaaatgtcgcatcgcagcttatgtatactggaattttgagaaactggagtcaggtctaggtttgtttcaaaactatggagtcaggtctggccgtctgggagtttggttgtagttgactatccttagaaagaaaaatgttgcctactggatttgaggctttgttgatttattcctgatgtgttgcatcagaatatgagctcacaagattctaattggtttttgtattgatgagtacattaatcaggtctagtttttttttttttttttgaagaacctcattgcgagggcaggagttcctgcagttcattaagaagaatagagatggtccagtttataaggaaaacctgaaccaaaagtctaacatatcccagttaattaggggaaccgagataaaacccaaaaacagaaaaaagacagtagagcttgggggaaccggttaatcaggtctagttcatattgcacattcttgtatttagtttacttccaagttccgagaagatgctgctttctgttcatggcttaggatcataggatgtagcccatctgtactataccttaaggggatttttcttgtaatattgttgttaaagccttggtttccatgagacctgcctgctcttaggtctttttctttcatggagtgttttttggtctaggtatctaactctaggatccaggcacttagttggtggttgctattttgtttcaggtgaagatccagttgggcgagggctctgcaacttctgtcaccaagaagatgcttgctaatgagggagttggttccttttacaaggtgtggcctcttaggtcttctgccatacagattctggtcatcgtgctgaggattgaattattgatcttgtgaatgtggcatgtactgcaatttggtgacaaacctgtgttactccatttgctagagaattagtctattacggttatgactggcatttcatttgtgcagggattatctgctggcttgctaaggcaagcgacatacacaaccgctcgacttggatccttcaggtttgtatgtaaaccattaactgaattcatcagtaactttcatttttttaagatctaataatttgcacatgaaggtttgtgaatttgacgttgatagtatgtctgatttttaagccggtgaatggaaaatgcataaacggcgtgaaataccattttgtatcaaatgattatgtctcatgcataaacagtagtttagatatttttaataacattttatggtaatgcttgctacctatcgttggtcgttgtacagataagcattttttaccatctaagtgcgcactttggcatgacccatcaattgtaggataacttctgaatcaacaatggtttattcatgatttagagttctattgaaaagatttccaagaggaaatactgatactaaggtaaggttgaagctgcaagactcatcactcctggagatggaataaaatatttaagttgaagccatcatacaacatcaaccattgtgttttttacatcactctgtactaactgcaaggcagatgatatgttatctgtcgtgttattactataaagagtattcaggaaaatcacttacacataatctgtcagctattttgattctgttttatcttagaagttagatcctaactacactagctgctgttcaatgtttgaatcaattttatgtcttcacgctgtattttctcagggttctcacaaataaagcagttgaagcaaacgaaggaaaaccactgcccttagtccagaaggctgctattggtctcactgctggagcgattggagcgtgttttggtagtcctgcggatttggcactcattaggatgcaggctgattcaaccttgccagcagcccagcgccgccactataagaatgcttttcatgcactttatcgtattattgctgatgaaggtgttctggcactttggaagggcgcaggtccaactgtagctagagcgatgtcactaaacatgggcatgcttgcctcctatgatcagagtgttgaggtacttagagacaaacttggtgctggtgaactttctacaatgcttggtaaggttattttagtttcattgaatttgatgcaatatttatgttttatcatttctcttctgagaattagctgtgcattttgagacgtgaatcagagtggtatgctggtcatgctatctataggccttggactctggcatcttcagtgcattgtctggaggagaataaagaataaaatgttagaaccgagaaatgattcagtgtagaagtaccatggacacctcaactatcacgccatgttctaaacttgagttaagatctctggattgctagcagggttctttgctttgtttgttagccgacctcatgtcgcaaggtgcatcttcccatctgtatgcgtaactcactcttccttaatagatgaggcagcatttcagtcaattttatttcaaaagaaaacctataggtttcaatgctccgctgacctgttgaagctagctgcatgatgataggcatgctgaaactagccttccatttgcttagtttgtgctccggttgacagatttgcatggaccttacttgccatttttccataaaaatataaacagatttgtatcacatgtgtcaaataagcagtgcactgctaccgaaataactgttcattcacatgcctttcattacacactttgatgtcagtcttttttgttgtagtttttcaccgtgttatgtttgtcttttcgttctgttattaaaactacatggtcctgatactcccattatcctcatgtttgctctactcattccttatatgatatattgaatttctcttgtaggggccagtgctgtttcaggattcttcgcgtctgcttgcagtttgcccttcgactatgttaagacacaggttcagaagatgcaacctgatgctactggaaagtatccatacactgggtctcttgactgtgcaatgaagaccttgaagagtggtggtccatttaagttctacactggctttccggtctactgtgtcaggattgctccacatgtcatggtaatgcaactaatacacggtgtttctctacttaatagtcaactcttcaacttcatgtctatcaaacttacttaatgacatcttgtatgcagatgacttggatattcttgaatcagatccagaaggtcgagaagcgtgtcggcctttaaagtgccgtgctatgctatccttgccgacaaattataatagtgtagctgctttgtacctacagtataaatcagcagttggttctgatagacaaaatctgtacttatctccttggcctatatgctaccattgttctctgtgtctttcagaagaaataatcatatcatttctgggtaacttttggctgaaagtagagttaggagcagatatgccaccaccgggttgaacccatctcatcagtggagccagacatcaaacaaactttttcagtactccctccgtccgaagaaacttgccccaagcttgttcctcaaatagatgtatctaacactaacttggtgctagatacatccatttgaaggacaagttttttcggacggagggactatatatttgtttaggccttatgtttgacaagtgtcaacatgtatctttttttggcagggtaatatgtgtagttaattctcgttgttgttgtatttttcttggtatatcacgcacaaacatttttgttggttctgttgtgtgccactgctgcatccggtgaaatgtgatagtggatgtttgtgtcagctgttgatcatgctaggtattgtatggtttctggtttgctgctgtcattctgtatgaaatgcacgatgtcggactgccacatttacagatctaaaatgtaaattacctacgataaatgttgaaaacattgcctcgtattttctttatcttgatgtactgatggaaatccaaaatgccgctgttacgtcttacattgttcatcatgtgcttcttttgatgatgcaggtccactggccttgccgcactaacagttcaacaggacatttcagttactactggtaccccttcttaatgctactatgacaggctatatactttgccagcattcagatccaagtactctcaacttccatcagagcatactaggtaggtagtagtgcccagcctaaaagggctatgcacaaaatagtaatagtactactagggcactagcatttggagaaaagaataagcggataacagaaaggctacggggcatggacaaatggactgtccctgcgccctgaccctgtggctgaatttcagtaatctagctcctcacgcacgcttgtagccagaacagaagcaccgacaacatacctgcaaattgcatttgaccagaacagaagcacccagttttgatatatggcagaaaagccagacaggaaaatttctgtagcctccttccgtgttgttgggctggcagctattagtggtggtcacatcattttcttgcccgtggccgtgtgttggatctgacgtgtaatgccaggcccgggcgccccatccatagttatatatccatattattgcgtttttaaaatattttccctaaatgtttatccattcttgaagttttctctcggcattttctatgctccaagaggtagccataatgatttttcttccgtttgttaaagcccgtgaaagtttcatcgtgtcgaccatgcagtgcgcccttgtgacttgtgaaaagaaacgcaaagtcaaataatgaacatggacaagggcactgtggaaaagtactagtttcttgtgttgttttgagcgaaggagagaggtaaggaacagactcaaccgtaaactgtcccctggtaccttgggaggctgctacgactagacgacaatctgggacattccttctgcttgtcttcttgagaccaactgaccttggagcaacaaaaaggccattattctctatctgaaaagatcttgtagagagtgtagtgttatctgatattaacctttttaaaggaatttttcaaacaacctctgaggctccgaagaatcctttcccatgtacattgccccttttcctccaagtgcaacgtgtgagaaatgtatgaactccgagttaagactgctcatattcatcaaaataatactaacgcatttttggtttctatttcctttccagatctcggaagagttaagtagttgccaggacaaacaggtggcatctaaggacgctatgccaaagtttcttgactaacacaagaaattgttgttgtttaagtggtgacccaccaaccttcctcctacgtggaggatagcaacaatgtgcctgtcaaatgttgaaatgtcatactgcaccgaccatctggtccaatcagaattggactcatattagtttccatccaattgtcaaatgaggatttgacatctaactattttccgactaaataacagtaccatcatagcagaaaagagaactctgaactgatcatctgcagatcaagatccttactcggaaccgacggccaatatagcatcataatccacaaatgaacagttggatcggcctctcatcatgtatgcccaaattagctctaggctgtcacatattcagatactctaactgtgcaagaaggcaaattctacaatgcaaaaaggaaaatcaattgacttgagaacacgaaaacttatttgcgcgttaattcactatacaatggagtaaagaaatgcaaaatctcaccaataagaattgttcatggagactaatgaaaaaagagaacattaccaaaaaagtcaatcttttctttaagagtttttttctttttgaaactctttgcctttagagtccagcgcggagcaccacactcctcggggcatctatttaaacggcatcccatgtctccattctattctctcctctccccccattcagttctccgcattcttttgtcgaagggaaattttcaaatttttttgaaggaaattctctcctctcgctccattctttgcagttagctagctagtgaagttctttcatttgattcttgctcgtggatcaatggcgcggatggcaagcaaggggagcagcggcnnccggcggggccagcgggcgcgagcgcgaggggaagaaggcgctgctgctggggcgcttcgaggtggggaagatgctggggcagggcaacttcgccaaggtgtaccaggcgcgcaacgtggccaccggcgaggaggtggccatcaaggtgatcgagaaggagaaggtcttcaagtccggcctcacggcgcacatcaagcgcgagatcgccgcgctccgccgcgtgcgccacccgcacatcgtgcagctctacgaggtcatggccaccaagctccgcatctacttcgtcatggagtacgtgcgcggcggcgagctgttcgcgaaggtcgccaagggccccctgccggagggcgaggcccggcgctacttccagcagctggtctccgccgtcgccttctgccacgcgcgcggggtgtaccaccgggacatcaagcctgagaacctcctcgttgacgacgccggcgacctcaaggtctccgacttcggcctctccgccgtcgccgagcagatgcgccacgacgggctcttccacaccttctgcggcaccccggcgtacgttgcccccgaggtgctctcccgccgcggctacgacgccgccaaggccgacctctggtcatgcggcgtcgtgctcttcgtcctaggcgccggttacctaccgttccaggaccgaaacctcgtcggcatgtaccgcaagatccacaggggcgacttccgctgccccaagtggttctccccggagctcctccgcctcatgcactgcgtgctcgacaccaagccgctgcgccgcgccaccatcgacgagatcatggacaacgaatggttcaaggtcggattccgccgcttctccttccgcatcgaggacgaccgctccttcacctgcttcgacctcgacgacggcgatgcctatgcgcccacctcgccgcccgacaccccgcggacggcggacggcagcgactacggtgacgcgaccgatcagcaacaaaaaatctcgggtgggatgacgtcgtgcggatcggcgccgtcgctgctggaagggaggttcgggcagctgggcgggagctcgcggcggcggtcgagcctgaacgcgttcgacctcatctccttctccccggggttc

>TaCIPK14_2AS_5117725

cggtactccagcggatcaccacttgtttgtttcatctcaatgaggtggtgggaaggtgtgatctcgaagatctccatgtctaactgaacagcaccattccttccctccttccttgcttgcatcttcaccacaccattgtccttcttccttaccctgagattcagcgcctttgcaacgtattccagcttcgagatgatggctgaagcaggcttgtctgaagtgaa

>TaCIPK14_4AL_7147326

atgtattatgtttccggttaatacaattctagcatgaataataaacatttatcatgatataaggaaataactaataactttattattgcctctagggcatatttccttcacttcgtctttgtggtctttgattaactctaacactagctcatttacttgtgttattacattggtcccttgaccttgcaaacttcatatatgttgtttatctcaccttagtaaaatttgtagttgaaatattcacccctctaggtccgtttcgatcctttcaggcgggggcgcgtgcttgatctgttctgatggtcataatagcatcataggcaggaatcaatgacctagcatgtgatcgcccgcatgtggggaaaacctacacacaagatgaggctgccggtaacaagctgacaaccaaacctgccagcgctccagagcacatgcccgacgatgacgctcacccactgccttctcaacaccgttgacgccatcgctcaaatgtggttgccaacatgaaatcaaaacctaaccttacctaagtgacagacatgcaaccccccacacacacaccacatgagcatcatacataggcgaggataaccaccaaccttatcggtggaacgacatgaggatctagagagagaaggacgacaataatgttggccaacctttaaacaagcaaaacatcgaccaacatttgcatttttacaccctccattcttttatgtaaggtgtattattcttggcacgatgaccaaggtacataattatagacaatttaggatgaaattaccctttccaattcatttattaccggcaagtaaatcatttcagctaggaaagaaacaaatacatgcaatcgggagagatatagtttccttttttctctacaaggagagatacatgcaatcaggtgagagatactttccttttttggagggtcaataacggaattacgaggaattagaacaaatgcaccttatattgtgaaattttatcaaaaaacaaatgcacctaatataaaggaatggagggagtaatagggaaagcttgagcacaccgaaaatctaaattagaccgttccctccgattccgtcttggtccttcggctccagttctgatcgatgaaggttagtcagctttgtcctcgtcggagagcgtcggagagttgtgtttcccgactccgtctgaccagacctacattgttatacaagccctatattacattttcttctcctggccaacgatttgctacgctgattgtggtcaccagcatcttcaggtctacattgatgattcctcaaccattgcttccacaagcttctatgtttaaacaagttttctcccttgagacagagacccagaaacaacaacgaactttgctcacggtgcgccaccaatggatgcaagaggaagaaggcttcggtaccccaaagatttgaatgtgcttttcaatttctataaggacgttcttataaatacgtgctacctaatatgacatcacgggtcgtacgagcacaattctcctctctcctaatcatgaagacacaatgatacgttttgtaccactctaccacgaatatgcccctctgcgatgcggtgcccaggaaacgcaccgggggcatcttggtcaaatccactagcttcttctcctcactcccccttccctccggcggctcctcccctgctctggctctgctccgctttatataagaagagcggcctcctcctccacaagcacacacaccaaaacaaaactccagcagctcacacagaggcacagacacagagagagaactgcctcgaggtcccggcttggatccggagcctcccctcttggcggcgccggaaggtacgcaaaatcccctccccgttctttgattccgttcagcctcaatgcgagccgtcgatgtaggcatccttgttggctagtttgtgcgtcttcttcagttttgccaagggcgaatcactggagaagcaattctttcccgtccatcagagtttgggcatatatgagccaaatttacggccttttaattgtcggcgaggctgcgttcagccgagtacggcggtgcattcctgcggttggatggatctgggtatttgcaagttgttatgctcttagttttaggattggaggaatggcccggttctctgcaaaatcaaacatgtttggccacttttcttcatgaatttgttgacggtgttatgtttctacgatgtaccaccgagttgctcctcaaccctgacttttctgcatacctctgtttacattctcaactggatgcacaagtatgcagccttgttgattctaaagtaaaattaataagaattacagatacgttttctgctgtttctagtttgccgtctgcacagaacaaatgttttttccttcatataaaccaccgaccaccatgtgcctgatcccgtcagatttgtttcctctaaagattatgcaataatcaaatttgtttcaccattcttctgtatgctccagttgttaagccaaccaataaatgttttgttatttcttcagtaatccatacaagggtatattttgaatttccaattgcagaatctgaagatttcttttcttatttgcagagaactccaacacagaacaagcactcaaagcctcggggaatgctgaaagatgtgcttgcttagaagaaatttcacctgcgcaataaaggagattgcctcttctccacaggagctgagttggtcgactctttatgtgagcaaccatcttgtttgcatcagctctgcaccatctagactccgtcgagagaaagacatgagagtgttgttcatcgtagtggatcaagactaccttggagaggatggttgaagtgctgcagtccatttgaattatagccgcaatataggctgtggttttatgcaacttgaagtcatggcaaacagagggaagattctaatggagcggtacgagctgggaagattgttggggaaaggaacatttggcaaggtacactatgcaaggagcctagagtcgaaccgaagcgtcgccataaagatgctggacaaggagaaggtgctcaaggttgggctctcggagcaaatcaggcgtgaggtcacaaccatgcggttggtggcacacaagaacattgttcagcttcatgaggtcatggcgacacgaaacaaaatatactttgtcatggagtatgtgaaaggcggtgagctctttgacaaggttgcaaagagtggcaagctcacagagggtgctgcacataagtatttccagcagctcatcagtgcagtggattactgccacagccaaggcgtgtatcaccgggatctcaagctggagaacctgctcctggatgagaatgagaaccttaaggtctcggattttggattgagcgcactttcagagtcaaagaggcaagatggcttgctgcacaccacctgcggaacacccgcatatgtagctccggaggtcatcagcaagacaggttatgatggtgcgaaatcagatatctggtcttgtggtgttatcctttttgttcttgttgctggttatctccctttccatggttccaacttgatggacatgtaccggaagattgagcaaggagatttcaggtgccccagctggttctcacacaaactccagaagctcttgttcaagattctggaccccaatccaagcaccagggcatctatccagaagataaaagagtctacctggttccggaaaggtccaaggggaacccttgcagtgaaggagagaactcccagtgagaacgtcaccacaaatgctcctcctacagctggtgtgaggccaaggaagaacactcatgaagatgtgaagcccctgatggtgacaaacttaaatgcctttgagatcatctccttctccacggggtttgacctgtctggcctattcatccgagaggagtgcagaaaggagacaaggttcacttcagacaagcctgcttcagccatcatctcgaagctggaatatgttgcgaaagcgctgaatctcagggtaaggaagaaggacaatggcgtggtgaagatgcaagcgaggaaggaaggaaggaatggtgctgttcagttagacatggagatcttcgagatcacaccttcccaccacctcattgagatgaaacaaacaagtggtgatccactggagtaccgggagctattggaggacatccggccagcgctgaaggacatagtctgggcctggcacggagatgaccaccaccagcagctagagtaggttttggaatcttgagtatttagttttggttctgcgaattcgtccagttttcagctggaacgatcctattttacatgcgtgtgttctttgtacaaaatatattttttcctacatgaggtgaggcattctgttcgaggcattgttgagaagttgaaatgcataataaaggttgttcctgtttagttgctgtcaacttgtcactgtcctgtcataatgctcattttgggtgaaattaaatgctatgccaagcatttacagtagaaagctcccactacacctccataaacacatttctcaaaacatacgacgtattagccagcacttgaacattttgtgatttgccatctctggaagaaacctacaactgttcagtttttttcatgatgtgagttgtaatttgggtaagataatcttcagaaagtcctctggagagatggctctcccatcatctagtttaatgtttattacttatcgggagctgaatatgtcccatggaaatttctttccagattttggaagaattttcatattaacagaggttgctcagcggtcatactcaataaccaatgcacagactaaggaagtagaaaatcaacaagtggtgcctattcagtctgatccgaaaagtagaaaatcaacaagtggtgccaacaatttgcagagagctgcagcttcaccgagaaagcgaaataatagaattttactaaagaggggtatcctcttattaaacaacagaaagtgaagttatcggtaaagaaaataaataccgagcgtataaattcacttgctggcaggttcgatctagaaaatatctgaactccactgtgagtaaattaaagaaattgtcactatcacaggggataggtccaacatccaagtgtttcttttcactttcctaatcaaccatccaaaaaattcaacagtaaaattactgatttgattgagctaaccaatcatagatttctcaaagtcgttgaaggataaactctttatgtcctaggtatccaacatatatgcttctgtccttgatgcctgatgccctgatccatgattgatgcctgctgcgatgcattgccgcagatccgaaagaaaatcttgctacggattgaaattgtaatctgacgacatggatatttactttatgaaaatcttgctattacaacttgctaatttgaagatttacatatttgtatcggttttgttagttggaactttgtaggatatttggtaaatcgatggcaggttaatttgcaataattgaactttatctctgtatgagtatatttgtacctaccaaatacaatgcttagactgatttcatttttatgagcagataaacaagaaaaacagaatagttcattttctacggacacgatgtacgcgaatgcggataaatagtttgcatggctgacatgctataactttatttttgctttagttgattatttatttatttttttgctatgcacctgaagatgtataatctggtgtgatgcttgattgaagattagtcagcaacgtgatgatagtgagatatcttatgatgacgacaggttggtttggaacatctctaccggtaaggatttttcagtacattcattatcttggaagatactgagaaatctcatgatggtgtaaggtttcggcgaagttctagatggaagtttaggtgttatcatctcacgaaagatataggcctcctttgatttggaggaatttcataggaattttagaggataggaattttataggattttttcctttagagccctttggttcataggaatagattcctattcctacataggattggttcctatcctccacattttacagaaaaataaaaatgagcctagactcaatgaaaaaattcctttggtgtcaaccaaatgacatctcgtttcctattcctactcataggatttgagatacatg

>TaCIPK14_4BS_4951408

agccagacgccaccgtcgacgacgacccctactacaccggaggtgcctactactacgtgcagcccgctgacaacgcccaggagtagtttaggaggatcccaggcaggaggcctgcgcctcttttgatctgtatcccagtttgtgctagccttcttaaggcaaacttgtttaacttatgtctgtactcagatattgttgcttccgctgactcgtctttgatcgagcacttgtattcgagccctcgaggcccctggcttgtattatgatgcttgtatgacttatttatgttttagagttgtgttgtgatatcttcccgtgagtccctgatcttgatcgtacacatttgcgtgcataattagtgtacgattgaatcgggggcgtcacagacacattgcgcaaggaagacattgcctcacatcaaggaagcccccgattggcacaaaaataggtagcaactttgtagcgaaggataagatggttgcaagcactagcaagaaaaatacaagacatggaagagatgacaacgtaaggacttcttcaataattgacatcaatgatacttctacattagatgatgtggatgaaagagagaacactgtcaatgacgaggaagaattaggcagtgaagatgctgatggcgatcaaccacaaataagtgaatcaagtgccaaagggaaaaaaactaagaaacaaaaaggtgccacatctatacagcggcttgaagattccattatggcttatgtgaatttcaagaaagagcaagcttccaagaaggaaaaagtgtcacagcaaggaaaacaaccctcaattacagaatgcttgcaggtcttgaatgacatggatgatgttcccgacgaggtcaaaatctttgcctctgatgttttcaaggatgcagcaaatcgtgagattttcctgggttacaactcaagattgcggggtatgtggctaaagaaggaagtcgacaagattagtcctcagcctcctccttgtaagtaccattatcttggttttctgcatttcttctccccactaaagataagtttgcaaggcttagtcgagcgagtattcctttgcagctcggttttcatctggcggtattccttcaatcaatctggtgctctgatcaaaggtattgtctgtgttccttttatttccattaggtgaagcataccttgcttgctcctagagtccctttggcctcaacgaggctccgctagtgggagggagtaacataatattctggacatgtttgttattatccacaatagacctgtgttgagaacttgcaattcttggagctatcatttttgcattcaaaatcatatagacctgctgctgtctagagcatcttttcttatttaagatgttgtttatattgctgccatgttgtttcttcatatcggtgttttttgaaaaataatatggtttctttaaatttagtgacatgctatggctaccttgtaccatctggctccgcttggtaattcaggattttgtgctaatctttttgacatacatggttgttcttgacatgtgtggctggtatccttcatagagcacaaagtgttcttacatagctaatgtttttcaattttttttcttttcaggatgtcctttcacatgattctatgattacccgagtccatgaagcaatcaataatcttcctgaaatattttatgtctaagcgcacaatggttgaacaactgcctcaataaatttggttatgtagcatgcggacaagacgagacaagtatgcctcgataaatttgtaatttaaactactgactttgagcactatgagttatcggaactatgttcaaatggatgattgcaaaaatattatcctttttttgatgtggttgtactgatatataaatcaaaattttagctcattttatacctgtgtaatttgtgtggaagtgcagtctgtgctgttgaaaattctgtacgtgtgtattagaaagtctgtgctgttgaaaagctatacatttgggtgccatgcatgtttgaacatatatgtagtgctgcaacttgggcgccatgtatattttacaaccgaattgaaactggagccaatttacaggggtggcagaaaaacaacattccaggcggaggttagaagcggaatggaaatacaaccctgtggcatgtattcggttcgagactagtataatacaggtgtaaaatgttacaggggtggcagaaaaacaacattccaagcggggccttagccttttttttcgtgggactctggcgatgcatgtaggctgtagcgttcattttttttatctgttttctgatttattagcgtcggattagaaattgctaggctgatttttttctatttccaaatgataaatgacacacatgtgactcggagcactccagtcctgacttttttacaactaaaataaagttttcattaaaaaaacggaaacttgtcatccgaaaagaaagtcgtcatgcttgaaaactaaaattgccatcgaaaacgttcaatgttgtcatgtattcatgtcacacgtatataacttatcaggatcctttttattgtattgtatttgttcttttctttgtaagcgcctacacaagcgctttgcagtggagatactcagtgtcgtacgatttttgttcggcagtccgtcgtttacacagtaattctggtattgggtttaatgtggccctgagctgcttcatgattgaacctgaaataggctgagagaccacgtaccagccaggtggtgatgtgtgtatttctcgcaagaaaaagctggtgatgtatgaagccacgaatcctagccagattaagttaccgacgacgatgtacactgtgatctggccgtgtatcttgcgcacatgcaacaagccaatgaatatagccagaattttggccgagattcaatgatgtacggtcgaaatcaattttggctaagtagaacgaaatcatagctaagcagaaagaacaacttctcaagcagacaagtacctatagatcttactcatttatgtatgcatttatcagtaaatgtggagtttttaacataaaaggatatgacatcttggtactccccaagtatagtagaaagtctaagaatattggggcatgttttttacgaaaatacaaatgtggacatggtttcacgcgcacctatatgaatagtaatttttaaagaaaatactagaaacataataaaaatctgaaattttggggtaccaaacttgatcgaccattctcttatgtgtcatgtttcgtggagaaatgacgcccatggtattcttatgaagaaaatactgttttgaccttactattcatcacacaatgcttttcatatagccttgattttgtcatttttgcctagaataccatgagtgtcatttcttcgtgaaacttcatgcacgagtataccggttgactatgtatgttcaaaaagctagaaacttttaattttttatagtatatttttttgaatttataatttatagggggtccacgtagaaccgtgtgttcaccaaatctgtggccgtatgcatctcccagacacagaggccgggggtcatcctcctttaaaaaaaacaaatttgtgtcccatttttaatatactactccctctggctactccagcgacaaatattacggtaagagggagtcatatattggctattgtaaggagttttgtagagttttcttaacacatgtataagtaatagagttttcagacttgatcttttcttgggtatgtactcctctcttaggtgaaagatcactggtcggtatcaaaatcatatgtacagctagccatgggaccacacgtgttttctgcaggcttgatgagacccaataatgcattatcccgctgtattttggatgtcactaattttttcttcaaacgaacagcgatgatacgttggtgctggatgtggcgccacgacgctgtcgacgctgcagccatgcgttacgtatacgtatccatccaaatcccttgcatgtcgctattactactctcgcgtgccttcttttgctctcttctcgcagtcaataatacttgcagcagtatgagttttcttttgtaggtagaggaacaatgtaggccgacttgcatgtgccgaaacacgtaggtgtttctacaattttgatacgacatagcaacaccgttgagcagcacagattcacggttcattgtattgaaatatattgccccctcttttgatcagttcggacttctgaggcaagtagctggagtatgaattcaatacactgcaccaccgtaacaaaattgcaattccgttctcattatgtagtcgatacaacagtgcatgttgacagatatatctggaaatgagggccatgtatgttgttttcaattggacataatagcggtacacaatatttatatgttgcgtctgctaacgcctcatagtagtagtctcccaaaatcagagtatgtgtgtataggcagtatggccaaggcaccacgtgtttctcgctccttgccacatcacgagcagactatgctaggtgagaacgaaaaatgatggcagttttcttgcgactaatctctgaaataaatggggtttgttggttggtgttgcatcttgcatgtggcgccacgactctgttgacggagcaccgcgtatcttccagatcccgtgtctcagttgcctgttgaaaccgaacacaatatttctctattatcaagtgccaagttttacttagccgtgtcttctgtcggtctgtcctcttgcaacgcagtaatgtgtcctggatacacgaacgtcggagtgaaccttgtatgtgtcgaaacgtaggcagagggtcatcgacggacaaacatctggctgatttcgcataatatataagatgacaagcgataaaatcagcaagcacgtaacaggatttcttttgaaatactccctctgtaaagaaatataaaatcgtctagatcattaatatttcttaaagagagagtactacctacatcctttttgcgataaagattaaaacatttatctttgttctttttgtggcaacattcgtcttttcttgcgaaaagcatcaaatttattgtaaaaactcatcggaagcacaaagcatttcaaatataataaaaattatatcgagattacgagaccaccagacaaccactactaccgacagaatgagccgccgacattgatccttattatttttttctcgaatatgcacgagtgtgcgtattattcattaaagaagagggggggggagagtccccaaaatccacgcgggctacaatagttacaaagttcccgagtccaccgggcaccaacttgtattacaaactactcataagctgtccgctcgctacctagctcttcaaagcctttaatgtcatctttaaacatttccgctttcgcccacagccttccttcctcaggtagccgctctttgactcgtgacagagaaactggagccccattgaacacgacgtcgtttctatgcttccaaattgtccacaaggctaggatgcatttagccctcgttgatctcctacctctcggttcccgatcctaccttaggcaccatgcactcaacgattcatcctgtaatggctccaactcccgaagccctgttgcccggctcatctcatgccagacctgtcgcgcaaacacacagccaagcagaaggtgttgcaaagtttcttcctgttggtcgcagagggggcacgcatcctgatggggaagacctcgactggctaagcgatccgatgtccagcatctgttttggatcgctagccagaggaaaaaacggcaatgcagcggcgcccttgactttcaagtaaacttagcagttggtgattcttcaaggcccatgaaaccagccgcgtaagccgatcgcgcagagaaactcccattcggctcccaggcccaagaagctgtgtcaggagtgttagttttattttaagctaatgtttacacactaatatttagattatattaattaagaaaagaatgtaaaaagaaatatctagatgtagggtagttgtccccaaattttacaggaaagaccacgtggaaacatacaaaaacatgtgagccctcaagaaaatccttctgaattgaatgcaccgccgctcgctccgtcattgcatcactagaggagagaaggaagatgagaagagcaacaactgtgtagtccacatagacccgtcgtgtaagacatgagttgggggcggaagaggccgaattagcaatgaaaaaaacatttgttcctctaagtggatttagtgtaaatgtcataccaattaaggagtaatattgtttcctaagtgtatagaaggtgtgttagtccataagtttgcgagcaaagatgattgatgaaccccatagctagagagagaagaaagatgacatatttgaaaatgtatctttgtttctctcttgatcttgagtcgtaggatagccacactattaagagtggtcatatgatgaacttaaagcgatgatttttctgttgaggatttaaagttataattatgttgcctacatatagccaaatacatagactaagttccatacataatttcaccaaatagcctcggtgagatttccacttccaaatattccagaggagagtttttgtgtggtccgaaacatcgtgtcagatctttcgagacgatgtttcgtggtgtccagaatccgggttactttccatattatgttctgaaagtggcataacaaaagtattgggttgttccaaaacccaactccgaaaccaactctgaaatcagaactagtgaaaggtcaaagtagaacgactgggaagggaagaggtgaataggcgtttatcaaaactctttgaaataaagaaacccattgcagaaaacatagataacaaactataatagtgtgcagtgccaaataattctaggaagataactagtgaaacatacaagcatgtatggaaacaaccaagtaaaaaaacactgcaatggccgaggtaagctaggaaagcattaaacaatgcagagagatatgctgaggaacttaactagcaacaggaagaaaggcttcaaagaaaacaacttgagaaacacttaacaaaattaaacagcggaaagttaagtgctcagaaagacttcacaacagtcacaaagagtgagagagagagagaaactcctcaaaacatgtcaaagtattgaaagtaaaggtttcaaagaattgaacccataaggttggtgaagacacgatgatttctttaacaaagttcaaactattggcaactattgtacatcttggttggagaggttgagcacactagtccgaggacacacaagtcctcgcctttttctccttgagttaagttccacgaaactcgtccaatactcaaggtagattttgcggtgatctttggtccggtgcaaacttgtccttcagtgaattcgaactttcgattcttacataactatgcctaaccgtctagaggacgccaagttctcaagagtaatagatcacgagctaggatcgagtcgattctttagtgatgctcagtcactttctgttcttcgccttgggtgattttcctcacttaggattggttcacatctctgcaaggaaggacgctctttgcagacgcagcaaaatatcactcgagcaaccacccgttatctaaggggcaggggctatatatagctggcacactgcccctgagtaccgatatgactattggatgagacacactcaccagcacgctactcatcaatgaggaaaataactcgggtgacttgaagaattaacttcatcacgggacctagtaggctcgcttggaaggagatttccaattaagtcttcactcacaagtgtagataggttcagactgagcacacacatgaagacttttagtcttgcttcactctgatcccccttaatagtacagtttgtcctacgactcagtgaagaattaaataaactagtaagtagcaatccactcttcggtcttcaattttcaacggcattcccgtagacttcaatgatccgtcaactttttctaatggtcgtaatccttcagttaaactaaatcatcagggattttcacctgtgcatatttaacatacatattagtcccttaaattttttttctttaatcatcaaaatctactaggggtagtagatgcacttacaactagctttcgggatgtgttaagaaattggcctataacctccaagagcttagaaagttgcggggccatatctagaacctatcccccaaatccggcagtccaaaccctatagtggatgttccgggatatatagcttctgggtggtcgaagttaaacctaatggacatatttcaaagtgggctatataaaggtccactcattcttcaccaagtgttttcttctttttctactcgagttgaacatatttgtctctcatactccatttcacaggctctgagccttgaatcctttcacatccattcttcaaggaaaaaatagagaggagccccgatctacgttctcaccaaaggaaatcgagttccctttgatttcatcatttggctttactcttgaaggttggagactcctaggcggtaggagtctccggtgaaggaatcaaattgtgtgtgacggtttggatctctcctcaaggtctacccactagtggttaagaatctcctctatggtgatgtttcaaggaaaaggtgagcctttgtggcgtttcttgttcctttatggaactcccgcctctccatttgtgactagctcccttccaagagagggaacactaggatacatcttcgtctctatggtctttggttaaccctaacactagcctgtttacttgtgttattacattggtcccttgaccttgcaaacttcatatatgttatttatctcaccttagtaaaatttgtagttgaactattcatccctctaggtctgtctcgatcctttcaggcgggggcacatgctcgatctgtttcgatggtcagactagcatcataggtgggaaccaatgacctggcatgtgatcgcccgcatgtgggaaaaacctacacacaagatgaggccgctggtaacaagccaacaaccaaacctgtcagcgctccaaagcacatgcctgacgatgacgctcacccactgccttctccacactgttgacgccatcgctcaaatgtggttgccaacatgaaatcaaaacctaaccttacctaagagacggacacgcaaaacctcccctccccccccacacaccacatgaggatcatacataggcgagaataaccaccgaccttactggtggaacaacatgaggatctagagagagaaggacgacaactaatgttggccaacctttaaacaagcaaatcatcgaccaacattttcattttttactccttccattcctttatgtaaggtgtattattcttggcacaatgaccaagatacataattatagacaatttaggatgaaattacccttgtccaattcatttattagcggcaactaaatcatttgggctaggaaagaaacaaatacatgcaattacccttgtccaattcatttattagcggcaagtaaatcatttgggctaggaaagaaacaagtacatgcaatcgggagagatatagtttcctttttttctgtacaaggagagatacatgcaatcaggtgggagatactttcctttttttggagggtcaataacggaattacgaggaattagaacaaatacaccttatattatgaaattttaccaaaaaacaaatacacctaatataaaggaatggagggagtaatagggaaagcttgagcacagcgaaaatctaaattaggccgccccctccgattccatcttggtccttcggcttcagttctgatcaatgaaggttagtcagttttgtcctcgtcggagagcgtcgaagagttgtgttccccgactccgtctgacaagacctacattgttatacatgccctatatgatgttttcttcccctagctaacgatttgctacgctaattgtggtcatcagcatctttacgtttgcatcgacgatttccagaccattgtttccacaagcttctactccctccattccaaaatagatgacccaactttgtactaaagttagtacaaagttggatcatctatttgagaacggagggagtaggtttaaacaagttttcttcaccgagacagagacctagaaaacggcaacgagttttgctcacggggcgccgccaatggatgcaagaggaagaaggcttcggtactccaaagatttgaatgtacttttcaatttctataaggatgttcttataaatactccgtgctacctaatatgacatcacgggtcgtacgagcacaattctcctctctcctaatcatgaagacacaatgatacgatttgtaccactctaccaggaatatgcccctctgcgatgcggtgcccaggaaacgcaccgggggcatcttggtcaaatccaccacaaactagtttcttctcctccctcccccttccctccggcggctcctcccctgccctgctctgctctgctttatataagaagagcggcctcctccttcacaagcagtcaagcacacacaccaaaaccaaaactccagcacctcacacagaggcacagacacagagagagaactgcctcgaggtcccagcttggatccggagcctcccctcttggcggcgccggaaggtacgcccgcaaaatcccctccccgttccttgatgttttttgattccgttcagcctcaatgcgagccgtcgatccttcttggctagtttgtgcgtcttcttcatttttgccaagggagaatcattggggaagtaattctttcccgtctatcagagtttgggtgtgtatgggccaaatttatggccttctaaatatcgtcgaggctgctttcagccgagtgcggcggtgcattcatgcggttggattggatctgggaatttgcaagttgttatgctcttagttttagcattggttggaggaatagcgcaattctctgcaaaattaaacgtatttggcctttttcttcatgatgaatctgtcgaccgtgttatgtttctacaatgtctatagtttgttagaaatactcctgcatacgatttttttccccaagaggtaaagatctagaagaccagcgagttgcttctgaactctgactcttctgtatacctatgtttacattctcaactggatgcagaagtatgcagccttggttctaaagtaaaataagtagaattacagatacgttttctgttgtttctaggtttttttttttgactaattctggtgtttgccgtgtgcacagaacaaatgttttgtttcatataaaccaccgaccgccatgtccctgatcctgtcagattgtttcctctaaagattatgcaagaatcaaatttgtttcaccattcttctgtatgccccagttgttaaaccaaccaataaatgttttgttatttcttcagtaatccatacaagggtatgttttgaattttcaattgcagaatctgaatacttcttttcttatttgcagagaactccaatacagaataagcactcaaagcctcggggaatgctgaaagatgtgcttgcttagaagaaatttcacctgcgcgataaaggagattgcctcttctccacaggagctgagttggtcaactctttatgcgagcaaccatcttgtttgcatcagctctacaccatctagactccgtcgagacaaagacatgagagtgttgttcattgtagtggatcaagactaccttggagaggatggttgaagtgccgcagtccatttgaattatagccacaatataggctgtggttttacgcagcttgaagtcatggcaaacagagggaagattctaatggagcggtacgagctgggaagattgttggggaaaggaacattcggcaaggtgcactatgcaaggagcctagagtcgaaccaaagcgtcgccataaagatgctggacaaggagaaggtgctcaaggttgggctctcggagcaaatcaggcgtgaggtcacaaccatgcggctggtggcacacaagaacattgttcagcttcatgaggtcatggcgacacgaaacaaaatatactttgtcatggagtatgtgaaaggcggtgagctctttgacaaggttgcaaagagtggcaagctcacagagggtgctgcacataagtatttccagcagctcatcagtgcagtggattactgccacagccaaggcgtgtatcaccgggatctcaagctggagaacctgctcctggatgagaatgagaaccttaaggtctcagattttggactgagcgcactttcagagtcaaagaggcaagatggcttgctccacaccacctgcggaacacctgcatatgtagctccggaggtcatcagcaagacaggttacgatggtgcgaaatcagatatctggtcttgtggtgttatcctttttgttcttgttgctggttatctccctttccatggttccaacttgatggacatgtaccggaagattgaacaaggagatttcaggtgccccagctggttctcacacaaactccagaagctcttgtgcaagatcctggaccccaatccaagcaccagggcatctatccagaagataaaagagtctacctggtttcggaaaggtccaaggggcacccttgcagtgaaggagagaactcccagtgagaatgtcaccacaaatgctcctcctacagctggtgtgaggccaaggaagaacactcatgaagatgtgcagcccctgacggtgacaaacttaaatgcctttgagatcatctccttctccacggggtttgacctgtccggcctattcatccaagaggactgcagaaaggagacaaggttcacttcagacaagcctgcttcagccatcatctcgaagctggaatacgttgcaaaggcgctgaatctcagggtaaggaagaaggacaatggtgtggtgaagatgcaagcaaggaaggagggaaggaatggtgctgttcagttagacatggagatcttcgagatcacaccttcccaccacctcattgagatgaaacaaacaagtggtgatccgctggagtaccgggagctattggaggacatccggccagcgctgaaggacatagtctgggcctggcacggagatgaccaccagcagcagctagagtaggttttggaatcttgagtatttagttttggttctgcgaattcgtccggttttcagctggaacgatcctattttaaatgcttgtgttctttgtacaaaatatattttttcctacatgaggtgaggcattctgttgaggcattgttgagaagttgagatgcataataaaggttgttcctgtttactactcctgtcataatgctcatttcgggtgaaattaaatgctatgccaagcatttacagtagaaagctcccactacacctccataaagacatttctcaaaacatacaacttactagctatgccagcacttgaactttttgtgatttgccatctctggaagaaagctacaactgttccgtttttcttttcatgatgtgagttataatttgggtaagataatcttcagaaaggcctctggagagatggctctcccttcatctagtttattgtttattacttatcgggagctgaatatgtcccaaggaaatttcttgccagattttggaagaattttcatattaacagaggttgctcagctgtcatactcaataaccaatgcacagactgtacgaaggaagtagaaaattgttagagtacgtaatgggcctaatgggcccattagtgttagggttaattagagataagggtcgcttgcttagaggtcaagtaagccttgcttgtgagtcaagtaaacctctctatataaagagaggagatgtatcaatctaatcaaacaagaattaagaaggccccctcttgcccggccgtgggcaaaaaggcccccggccagccctctcgcgccctccttctagcagcggcataacaaaaatcaacaagtggtgcctattcagtctgatccgataagtagtaataaaactggtaatagatagccaacgatttgcagagagctgcagcttcaccgagaaagcgaaataatagatttttatccaaagaggggtatcctcttattgaacaacagaaagcaaagttatcggtaaagaaaataaatatcaagcgtataaattcacttgctggcaggttcgatctacaaaatatctgaactccacttgtgagtaaattaaagaaattgtcactaccacaggggataggtccaacatccaagtgtttctttttcactttcctaatcgaacatccaaaaatatcaacagtaaaattactgattcgattgagctaaccaatcacaactatgtcaagtcgttggaggataaactctttatgtcctaggtatccaacatatatgcttctgcccttgatgcctgatgccctgatccatgattgatgcctgctgcgatgcattgccgcagatccgaaagaatatcttgctacggattgaaattgtaatctgacgacatggatatttactttatgaaaaccttgctcttacaacttgctaatttgaagatttacatctttgtatcggttttgttagttggaactttgtgggatatttggtaaatcgacggcaggttaatttacattaattgaagtttatttctgtatgagtatgtttgtacctatcaaatacaatgcttagactgatttcatttttatgagcagatagacaagaaaaacatgatagttcattttctatggacatgatgtacgcgaatgcagataaagagtttcgatggctgacacgttataactttacttttgctttagttgatgttttttttttgctctgcatccgaagatgtataatctggcgtgatgcctgatgaggattattcagcaacgtgaagatagtgagatatcttatggtgacgacaggttggtttggaacatctctaccagtaaggatttttcagtacattcattgtcttggaagatactgagaaatctcatgatggtgtcaggtttcggcgaagttctagatggaagtttaggtgttgtcatcttacgaaagattatcttttaagaaagaagtgttctctttgtcgcaaccccgagagaattaagcatcaatgttttagatgcccagttgatgctgattaactgtgcttgatgccacattggttcccctgtccctgaaacatatgcttgatcaaatttttaaggaacaggtgaatcaagaacaaagcggaaacaaggcgctgctactcgtccgccgtgagtgatcaaggcctgtctctgaaggaagaatgaagttcagagacagggaaggatagggcacagaggatgtaatccaacatgtcaaagatagacttcaaagtaatatcaaagctcagctcataagcgttggaacgagtagcactacaggtacagagcatgcaacatggtggtacaaaatagtacggaataagtttttcttttggaattatagaaaatagtaaccgataggtaacaaaatacagaaatggtgggtaacaaaatacagaaatggtaggtaacaaaatacagaaaatccctacatttctgtaagaggctgttgtcaaggcttaaacccgtggccttatggtcacaaggcagcagctttaccactgcaccaaggctccccttcgcaaaaaaaaaactatggatatccacacaaaaaaactacggaaagactatccttttattaaaccgctgaaagcagagttagcgattaacccgagcacttgcagacaagtttgatccggagtatctaaactccacttgtcagagttttttaaagaaatcgtccctatcactagggaggatagatccaacacccaaccccttcactttcctaacaagccgtccaaacaatttcagaggttaaataacaataatggttcagcaaacaaatcacagcaatgccgagttgttagtaaaagatactatataaactcttgatgtcccaggtttccaacatagatgctttgtccttaatgcctgcctgatgccatcaaccatatgatagatagatgccagatgcgacgaatcgccgcaaacaaaaagaaattaaatgaatcaattttgtcatctgacgacaggtagatgtgtacctttacggctaagctcgccttttctggaaacatgataaccgcgcaagtggttggttggtgcatagacggttttccactgtcggtccttttagatttacctattttccccttcttttattctctctctctctctctctctcacgaaacagtcttttgtccgctttataaatataaagccacactaccgaatgatacatagtgctaaggaaaccctcaaacatgctgataaaagcactactactcgtctccaacaaaccgcctagaataagcacaagagaggccagacaatgcaagacgccatcaaggccaccgacaagcgaacacatgacacctagaagaaataactggatttcgtcgcaccaccccaccaaactgttgtcggagaggaccctccatcctctcgctccagacctaggaacgttgaccctgccgtcaagcagaaacgaccgagagaagattccatcaagagtaaaagataggtgcaaacattagttgtcagaactgaggcgttgctggacggaactctaaatcatctcgaacatgccgcaacgaccgaaagacacatgccctcgctaccgatgccctcaagagggtcatgaaacaaagtgtcgccgccactatgtctgccaaagtgaacatggattttcaccctggacattggaaggaggagaaaagccaaggaggcattcccaagaggatacttctttcttgataggccgccctagattggtgctagtttttgtatcctaattgtcaggaccccgactcgatgccacatcgatctagcatgtaacacctcatatcgctttgcggcctcacgcacggtatccccacgggtgtcgccttacctttgcccgagaccgtttgcgcattttggcacacgtatataatggtgtcgctagcatccatatgataaagagcccgggctgacatggctagtcgtaaacccaaagtggcactaacttacagggacaggcattcatgacccaacatcgaacgtgtcggtcatcagcgagtgaatccaggctgtagcactgggctaacaggactccggtgaaccgggctgtagcgggctagcaggactccggtattcatcgcgtgacatttccccgaaggaacagacacaggatcgaagaaggacacatgccggccagcctaagtgttccggagcagtagcaagctaccagg

>TaCIPK14_4DS_2283949

atttatcatgatataaggaaatataaataacaactttattattgcctctagggcatatttccttcaagaggacggagggtcctctccgacaacagtttggtggggtggtgcgatgaaatccagtgatttcttctaggtgtcacgtgttcgcttgttgtcggcggccttgatggtgtcttgcggtggtctggcctctcttatgcttattctaggcggtttgttggagacgaatagtagtgcttttatcagcatgtttgaggtttccttagcaacatgtattattcagtagtgtggctttatatttataaagcggacaaaagattgttttgtgagagagagaataaaagaagggtaaaataggtaaatctaaaaggaccaacagtggaaaaccgtctatgcaccaaccaaccacttgcgcggttatcatgtttccagaaaaggcgagtgtagccgtaaaggtatacatctacctgtcatcagatgacaaaattaattcatttaatttcttttggtatgcggcgattcgtcgcatcaggcatctatctatcatatggttgatggcatcaggcaggcattaaggacaaagcatctagagtatgttggaaacctgggacatcaagagtttatatagtatcttttactaacaacttggcatagctgtgatttgtttgctgaaccaaattattgttatttaacctctcaaattgtttggatggcttattaggaaagtgaagggttgggtgttggatctatcctccccagtgatagggacgatttcttttaaaaactctgacaagtggagtttagatactctggatcaaccttgtctgcaagtgctcgggttaatcgataactctgttttcaacggtttaataaaaggatagtctttccatagtaaatatattaatctctttccggtgaatttgcagttttctgcattttgttacctaccagttactattttctataattctaaaaagaaacttattccgtactattttgtaccaccatgttgcatgctgtgtacctgtagtgctacttgttccaacgcttatgagctgagctatgatattactttgaagtctatctttgacatgttggattacacctgctgaagaatgtacagaaaacagtatacatcctatgtgccccatccttccctgtctctgaacttcattcttccttcagagacaggccttgatcgctcaggactccccagagcatcgcgtagcagcgccttgtttccgctttgttcttgattcacctgttccctacaaatttgatcaagcatatgtttcagggacaggggaaccaatgtggcatcaagcacagttaatcagcatcaactgggcatctaaaacattgatgcttaatgctctcggggttgcgacaaagagaacacttctttcttaaaagattatctttcgtgagatgataacacctaaacttccatctagaacttcgccgaaacctgacaccatcattagatttctcagtatcttccaagacaatgaatgtactgaaaaatccttaccagtagagatgtcccaaaccaacctttcgtcatcataagatatttcactatcttcacgttgccgactaatcctcaatcaggcatcacaccagattatacatcctcgggtgcagagcaaaacaaaaatttactaaagcaaaaataaagttactctataacatgtcagccatgcaaactctttatctgcgtccgcgtacatcgtgtccatagaaaatgaattatcatgtttttcttgtttatctgctcataaaaatgaaatcagtctaagcattgtatttgataggtacaaacatactcatacagaaatagacttcaattcttgtaaattaacctgccgtcgatttaccaaatatcctacaaagttccaactaacaaaaccgatacaaagatgtaaatcttcaaatcagcaagttgtaagagcaaggttttcataaagtaaatatccatgtcgtaagattacaatttcaatctgtagaaagattttctgctggatgcctaggcaatgcatcgcagcaggcatcaatcatggatcagggcatcaggcatcaaggacataagcatatatgttggatacctaggacataaagagtttatccttcaacgacttgacatagctgtgattggttagctcaatcaaatcagtaattttactgttgaattttttggatggttgatcaggaaagtgaataagaaacacttggatgttggacctaccccctgtggtagtgacaatttctttagtttattcacaagtggagttcagatattttgtagatcgaacctgccagcaagtgaatttatacgcttgatatttactccctctgtaaactaatataagagcgtttagatcactaaagtagtgatctaaacactcttatattactttacggagggagtattttctttaccgataacttcactttctgttgtttgataagaggatacccctctttagtaaaaatctattatttcactttctcggtgaagctgcagctctctgcagagtgttggctatctattaccagttttattactactttccggatcagactgaataggcaccacttgttgattttctacttccttagtacagtctgtgcattggttattgagtatgacagctgagcagcccctgttaatattcagctcccgataagtaatcaacattaaactagctgatgggagagccatctctccagaggactttctgaagattatcttacccaaattacaactcacatcatgaaaaaaactgaacagttgtagctttcttccagagatggcaaatcacaaaaagttcaagtgctggcatagctaataagttgtatgttttgagaaatatgtttatggaggtgtagtgggagctttctactgtgaatgcttggcagagcattatgacaggacaatgacaagttgacagcaactaaacaggaacaacctttattatgcatctcaacttctcaacaatgccccaacagaatgcctcacctcatgtaggaaaaaatatattttgtacaaagaacacgcgcatgtaaaataggatcgttccagctgaaaaccggacgaattcgcagaaccaaaactaaatactcaagattccaaaacctactctagctgctgctggtggtcatctccgtgccaggcccagactatgtccttcagcgctggccggatgtcctccaatagctcccggtactccagcggatcaccacttgtttgtttcatctcaatgaggtggtgggaaggtgtgatctcgaagatctccatgtctaactgtacagcaccattccttccctccttcctcgcttgcatcttcaccacgccattgtccttcttccttaccctgagattcagcgccttcgcaacatattccagcttcgagatgatggttgaagcaggcttgtctgaagtgaaccttgtctcctttctgcagtcctcttggatgaataggccggacaggtcaaaccccgtggagaaggagatgatctcaaaggcatttaagtttgtcaccattaggggcttcacatcttcatgagtgttcttccttggcctcacaccagctgtaggaggagcatttgtgatgacattctcactgggagttctctccttcactgcaagggtgccccttggacctttccggaaccaggtagactcttttatcttctggatagatgccctggtgcttggattggggtccaggatcttgagcaagagcttctggagtttgtgtgagaaccagccggggcacctgaaatctccttgctcaatcttccggtacatgtccatcaagttggaaccatggaaagggagataaccagcaacaagaacaaaaaggataacaccacaagaccagatatctgattttgcaccatcgtaacctgtcttgctgatgacctccggagctacatatgcgggtgttccgcaggtggtgtggagcaagccatcttgcctctttgactctgaaagggcgctcaatccaaaatccgagaccttaaggttctcattctcatccaggagcaggttctccagcttgagatcccggtgatacacgccttggctgtggcagtaatccactgcactgatgagctgctggaaatacttatgtgcagcaccctctgtgagcttgccactctttgcaaccttgtcaaagagctcaccgcctttcacatactccatgacaaagtatattttgtttcgtgtcgccatgacctcatgaagctgaacaatgttcttgtgtgccaccaaccgcatggttgtgacctcacgcctgatttgctccgagagcccaaccttgagcaccttctccttgtccagcatctttatggcgacgcttcggttcgactctaggctccttgcatagtgcaccttgccgaatgttcctttccccaacaatcttcccagctcgtaccgctccattagaatcttccctctgtttgccatggcttcaagctgcataaaaccacagcctatattgcggctataattcaaatggactgcagcacttcaaccatcctctccaaggtagtcttgatccaccacagtgaacaacactctcatgtctttgtctcgacggagtctagatggtgcagagatgatgcaaacaagatggttgctcgcataaagagtcgaccaactcagctcctgtggagaagcggcaatctcctttatcacgcaggtgaagtttcttctaagcaagcacatctttcagcattccccgaggctttgagtgcttgttctgtattggagttctctgcaaataagaaaataaatcttcagattctgcaattgaaaattcaaaatatgcctttgtatggattactgaagaaataacaaaacatttattggttggttttacaactagaggatacagaagaatggtgaatcaaactaaattctcgcataatctttagaggaaacaatctggcaggatcagggacatggtggtttatatgaaacaaaacatttgttctgtgcacacggcaaactagaaccaacagaaaacatatctgtaattcttcttattttattattactttagaatcaaggctgcatacttgtgcagaaaagtgtaaacataggtatgaagaaaagtcagagttcagaagcaactcgctggttctctggatctttaccgcttggggaaaaaaaatcgtatgtatttctaacaaactatagacattgtagaaacataacacggtcaacaaattcatcatggagaaaaaggccaaataagtttaattttgcagagaattgcgctattcctccatccaatcctaaaactgagagcataacaacttgcaaattcctagatccatccaaccgcaggaatgcaccgccgtactcggctgaaagcggcctcgccgacaattaaaaggccgtaaatttggctcatatgtgcccaaactctggtagacgggaaagaatttcttctccagtgattcgcccttggcaaaactgaagaagacgcacaaactagccaacaaggatgcctacatcgacggctcgcattgaggctgaacggaatcaaagaacatcaaagaacggggaggggttgcgtaccttccggcggcgccaagaggggaggctccggatccaagccgggacctcgaggcagttctctctgtgtctgtgcctctgtgtgagttgctggagttttgttttggggtgtgtgcttgtggaggaggaggccgctcttcttatataaagcggagcagagcagagcagggcaggggaggagccgccggagggaaggggcgagggaggagaagaagctagttttggtggatttgaccaagatgcccccggtgcgtttcctgggcaccgcatcgcagaggggcatattcctggtagagtggtacaaatcgtatcattgtgtcttcatgattaggagagaggagaattatgctcgcacgacccgtgatgtcatattaggtagcacggagtatttataagaacatccttatagaaattgaaaagtacattcaaatctttggggtaccgaagccttcttcctcttgcatccattggcggcgccccgtgagcaaagttcgttgccgttttctgggtctctgtctcggtgaagaaaacttgtttaaacctaggagcttgtggaaacaatggtctggaaatcgtcgatgcaaacctaaagatgctgatgaccacaattagcgtagcaaatcgttagccaggggaagaaaacgtcatatagggcctgtataacgatgtaggtcttgtcacaactcttcgacgctctccgacgaggacaaaaccgactaacctttatcgatcagaactgaagccgaaggaccaagacggaatcggaggggacggcctaatttagattttcggtgtgctcaagctttccctattactccctccattcctttatattaggtgtatttgttttttggtaaaattccacaatataaggtgcatttgttctaattcctcgtgattccgttattgaccctccagaaaaaggaaagtatctcccacctaattgcatgtatctctccttgtacagaaaaaaggaaactatatctctcccgattgcatgtacttgtttctttcctagcccaaatgatttacttgccgctaataaatgaattggacaagggtaattgcatgtatttgtttctttcctagcccaaatgatttacttggcgctaataaatgaattggacaagggtaatttcatcctaaattgtctataattatgtaccttggtcatcgtgccaaaaatatgaaggaaatatgccctagaggcaataataaagttactatttatttcctcatatcatgataaatgtttattattcatgctagaattgtattaaccggaaacataatacatggtgaatacatagacaaacatagtgtcactagtatgcctctacttgactagctcgttgatcaaagatggttgagtttcttaaccatagacatgagttgtcatttaattaacgggatcacatcattaggagaatgatgtgattgacttaacccattccgttagcttagcacttgatcgtttagtttactgttattgctttcttcatgacttatacatgttcctgtgactatgagattatgcaactcccgattaccggaggaacactttgtgtgctaccaaacgtcacaacgtaa

>TaCIPK15_5AL_2741722

gaagggattggtaggtgtaggattttgagttgagcatcacatggaaatttttccttagtatttcctcgaccccctttaacagtacagtgtttcctatgactcaaaaaaaagaaaatgaaactatgaaaacaaaagtcttctcgcttcatgttcctcgaaagaataccaagtcttcaaggtcacaccaatttcttcactttcaaagtcttcagaaagtcttcagaataccaaaatcttcagtcgaagatattcatttttaggggtcgatttttcctgtaaatatcaaactcctcatagacttatagacctgtgtacactcacacacgcattagtcccttaacctataagtcttcaatacaccaaaatcactaaggggcactagatgcacttacacgcttaattcccgttccttcccaaaagaagcgggagcggggcatgtccaatttagcgtgaaccccatccgctaacaagaacattcccatggtgaacattggaagagaggataggctagagttagtaaggataagtcgccccgcggaagacataaacctcccatgccacgggctcactctacccgcaacctttccatataagggctcccactccttaatggaaagccggctatgagaaaccggtaagccaaggtatttgattggcaagctccctagcttgcagtttagaagattttccactctcgtactctcttggtcatccatccccatggttatgacctcgctcttaaagaaattgattttgagcccagataagggttgaaggcaagcagcagcagcttgatcgaggctatgctattctcgtcaggctcaaaaagtagcatcgtgtcgtcagcgtaatgaaggtgcataacccctcccgggatcaggtgagacaccaaccctttgatgtgccctgccgagcgagccccatgcaccatagcagataaagcgcccgcaacaaaattaaaaacgattggagacagggggtctccttgtcttaggccttttttgtcgtggaagaatatgcccacctccccattaatagagaccgccgtttgtccacaattaatcaggttcatggtacggtggacaaaaccagcgtcaaaacctttttttaggaagaccttccgtacaaactcccagtttacacgatcgtatgctttctcgaagtcgagcttgagcagcaccccttgctgccttttcattttaagctcatggacgatctcgtggagggcgatagggccctccaaaatattccgtcctttgagaaaagccgtttgacttctaagaatgacctgctgcgcaacgagggccagacgagtcacgtaggccttggcgcagatcttgaaaggcacgctgatcagcgtgatgggtcggaaaagtttgatgcaatcagcccctttaacctttggaataagtctaatgaccccaaaattgaggcgtgagacgcctaccgttcatagcgcaaacccattcacaatgtcaagaaaaatgggccgaagcataggccaaaatctgcagaagaattcgacaggccagccgtcagggccggnnnnnnnnnnnnnnncttccgggagaaatgagagcgccaggctctcattctcctgcgatgacaccctatcctgaggatcccacaaatcctcacggatacagagttttttctcgtctgtcgtacccaacagaccaacaaaatactcgtaaatgtggcgggcaatttctccctgggagaggaggaggccctggtccaactagacgaaggatcgtgctcttacgctccctaccgttagcatatgcatgaaaataagttgtgttagcgtcacccttagtcacccacttaatgccgccctacgacgccagtattcttcttccgcaagcaacagttattacttgtcgaatacaaaagaaggaacaatttatttgtgacaaagagcacactcatgtagagtatataacacaattactcgagctgaaaaaaaaagcagaactaaacataataagtgagacatctaaatctacccttgaggctcctgctgaccatcagctccatgccatgcccagacaaatgtccttcagtgctggccggatgctctcctccaatagcttctggtactccagtgaatcgccgcttgtttgtttcatctcaatgagatggtacgaggggctgatctcaaagatctccgagtcaaactgaagaacaccattcctgccttccttcctcacttgcatcttgacaacaccattatccttcttcctcaccctgagattcagcgtcttcgcgacggcttccagcttcgagacaatggctgaggcaggctgttctgaagtgaaccttgcttcctttttgctctccttctcgacgaataggccagagaggtcaaaccctgtggagaaagagatgatttcaaaggcatttaaatttgtcactgccaggggcttcccgtcttcatggctgttcttcctgcgcctcacaccaagtgttgggacagcatctgtggtggcattctcacttggcagtctctccttcgctgtaaggttctccccgggacctttccggaaccaggtggactcctttatcttctggattgatatcctcttgttcggatcagggtccatgatcttgtacaacagcttctgaagtttttgggaaacccagccagggcacttgaaatcgcctttctcaatcttccggtacatctctatcaagttctgaccttggaaagggaggtaaccagcaacaagaacaaacaggatgacaccgcaagaccagatatctgattttgcaccatcatagcctcccttgctgatcacctctggagccacataagcaggtgatccacaggtggtgtggagcaagccatcttgtctcttcgattctgaaagtgcactcagcccaaaatcagaaaccttcagattttcattctcatcaagcagtaggttctcaggcttcaagtcccggtgatacacgccacggctgtggcagtaatccacnnnntcggtgagcttgccacccttggagaccttgtcaaagagctcaccgcctttcatgtactccatgacgaagtagatcttgcttcgtgtcgccatgacctcatggagctgaacaatgcttttgtgcactaccaaccgcatggttgtgatctcacgcctgacctgctccgcgagcccgaccttgagcaccttctccttgtccaaaatctttatggcgacacctcggtttgactcaaggttcttggcgtagtgcaccttgccaaatgctcctttccctaacaatctccccagctcatacctccccatgacgatcttcccactgttctccatgccttcaagagtataagtcacggccactaatattcttgtggctagcacttaaatatgctacgacacttcagccagcctccc

>TaCIPK15_5BL_10796477

cccccctagggcgcgcctcccctcttggccggccccctcctccctccctcctttatatacgggggcggggggcaccctatagacacacaagttgatcatcgtgatcgttccttagccgtgtgcggtgcccccctccacgacattacacctcggtcatattgtagcggtgcttaggcgaagccctgcgacagttgaacatcaagatcgtcaccacgccgtcgtgctgacggaactcctccctgtacctctgctggatcggagttcggggtgcgtcatcgagatgtacgtgtgtcaagaactcggaggtgccggagtaacggtgcttggatcggttggaccgggaagacgtacgactacttcctctangttgcgtcaacgcttccgcttcggtctacgagggtacgtagacaacactctcccctctcgttgctatgcatcaccatgatcttgcgtgtgcgtaggaaaactgttgaaattactacgttccccaacacttgctccactgagcaaggcggagcgccctttttttgagcttatggtggaggacctggtacggttcagaatgactcacccgctccgcccaagcatcaagaacgacctcgtgaaatccgggcaaagataccaagaaattctcaaatctgaatgtacgcgggcgcctagggcccttgtcatcggcgagcaacagagggcaatggtcagagagggaagatgacagggcatgtagcaagtgattgttgtgagtggtgtcccactcggcattatagaagaaggaatcaagcttgcacaaggtgggattggcgcgttcgttgctccaagtgaagcgtctgttctggaggtggatttccttgagttcacaggaatgaagcgtcgagcggaagcgtttaattctactcgtgttgacgttcctcttgttcttatccctggctcggtgaatttggttaaagtccccggcaaccaaccaagccacatccggaggtggtttgtggttgagcaactccgcaaaaaaagcgtctttgcacgaggaatcagtagggccatagaccgaagtgagcttgaagcaggagccagaagcacgtatacagaacgtggcagaaatacagtatgcagatgtgacaaagtcagaagcatcaagtagattctcatcccatagcaataggataccgcccctagttccgttcgccggacgctgggcaaagccacccagtctgtttcccccaagtgacgcaacgatgaatttgtcgacaacattcaacttagtctcctgaagacagacaagctggcaggaggagtcggcaagcgttgctttaacagtagctcttcggtctgggcaattaagcccccgcacattccagctaattgtgtttatcggttgtactgtcattggaaagtaaggtggccctggaggtttggatgaagcccatgatgggggcgcacacaagcatgaacaagatggaagcatggactagcacaagaccaaatggacgaagggccgccactaaaaaccacgtacagagttgcactaacatgagctattgcccttgaactagtacaaaccacgtacagagttgtagttcttccacaaatagagctattgcccttgaactggaacatacccttacaaaacaacagatgttgcgctaacatgaggaacaacgcacccagcagcagctactacatgatctaaacctcaggcattcacatccagcgagtccgcggcaacatctgctcccacagcgtcgaggacccccgctccaccatgctcgatcagagcatcttccaccgctgtaacagccccattgtctagctcaaagaactctctcatggccacaattacatcaggaggaagctgctctttgaactttacagcgaaggcatccagggtggcagtggtgacgtcctgcccgttcttgatgatgcccatcaggaggaagttttttcaatattgaaacttcagagcatcatatcctgtattactcccaccctgctttgctcttcagaaagttgcttggctggctattaccagtgtgaaaatgggctgttggatagggacaatctctttaacttgcagctggagctacaatattttctagataagacttagccaatgagctgttggatagggacaacctctttaacttgcagctggagctacaatattttcttgattaggcttagtcaacacgtgaagtacattgatcctgcttagtatgttattttatcatatatgtgatttgtttgtcaatttacttccagtgaacttgcaagttgcatgtttatacgctgcattcctaatctactagtactctactactgattgtgcaagtgtttctgcctgatggaagtccgcttattgggacagtctggtcatcgagaagatgcatacggcagcaaagcaccaaatgctagtgtcctgtttccagggggggaaacttttagtagtatgatgatttcttcttaagtggtcatcacctgcatactgaattcagtatccagtgatataatgacaccgtcagcatatgaccttatctgtttatgctcggcacagtatttgaccaaaaaaggcattagtagttcagtacataaacaagatttacaaagcatatcaatatgtatttcattatgatatatgttacagcaaagaaacagcttaatccatggcctcagcttagcaacagttattacttgtcaaatacagaagaaggaacaatttatttgtgacaaagagcacactcatgtagagtatataacacaattacttgagctgaaaagcaaaaaaaaatgcggaactaaacataataagtgagacatctaaagctacccttgatgttcctgctgaccatcagccccatgccatgcccagacaaatgtccttcagtgctggccggatgccctcctccaataacttctggtactccagtgaatcaccgcttgtttgtttcatctcaatgagatggtacgaggggctgatctcaaagatctccgagtcaaactgaagaacaccattcctgccttccttcctcacttgcatcttgacgacaccattatccttcttcctcaccctgagattcagggtctttgcgacggcttccagctttgagacaatggctgaggcaggctgttctgaagcgaaccttgcttcctttttgctctccttctcgacgaataggccagagaggtcaaaccctgtggagaaagagatgatttcaaaggcatttaaatttgtcaccgccaagggcttcgcatcttcatggctgttcttcctgcgcttcacaccaagtgttggagcagcatcacttggcagtctctccttcgctgtaaggttctccccgggacctttccggaaccaggttgactcctttatcttctggatcgatatcctcttgttcggatcagggtccatgatcttgtacaacagcttctgaagtttttgggaaacccaaccggggcacctgaaatcgcctttctcaatcttccggtacatctctatcaagttctgaccttggaaagggaggtaaccagcaacaagaacaaacaggatgacaccgcaagaccagatatctgattttgcaccatcatagcctcccttgctgatcacctctggagccacatatgcaggcgatccacaggtggtgtggagcaagccatcttgcctcttcgattctgaaagtgcactcagcccaaaatcagaaaccttcaggttttcattcccatcaaggagtaggttctcaggcttcaagtcccggtgatacacgcctcggctgtggcagtaatccactgcactgatgagctgctggaaatacttgtgtgcagcaccctcggtgagcttgccagccttggaaaccttgtcaaagagctcaccgcctttcatgtactccatgacgaagtagatcttgcttcgtgtcgccatgacctcatggagctgaacaatgcttttgtgcgttaccaaccgcatggttgtgatctcacgcctgacctgctccgcgagcccaaccttgagcaccttctccttgtccaaaatctttatggcgacacctcggtttgactccaggttcttggcgtagtgcaccttgccaaatgctcctttccctaacaatctccccagctcatacctccccatgacgatcttcccactgttctccatgccttcaagagtataaatcagtattcttgtggctaccacttaaatatgctacgacacttcagccagcctccccgaagtcatgatccagcactctggtggattcgtcatagcggagtctgaatggcgtggagttgatgcaggcaaggtggctgctcacatcaagaggtgaccaaatgggcccacgcagagtggtgatgttcttggtctcgcagaagaaatctctcctatgcaagcacatctttcagcattccttgagactttgatttcttgtttttaatgaattccctgaaaaacaagtaaccttcagatttggcagacacagatttaataatacgtagtaataaggataagtactactttcgatccggttttccttataaactggatcaaacacttggcattttggccactttgagcaatatattca

>TaCIPK15_5DL_4490016

gggatagccattaaagataagatccgttggagtataacagaataaaaaaatggacttaatggaagatgacgtcacggcgggttaccaggactccagaagatgacgtcacggtgggttataaccttcgcatgaccataagccggaagattttttctctcataagaattgaagattttttctctcaaaagaattgaagattgacatgaactggttcaaatcaatctggggcctaatgttgaggatataaccattagagtcacccgcccaggagggaccgggttactcataatggtcatcacgcgaagcccagtaccgagtttgaagacggcgggtcaataatggacttaagacccggagatggctaaaggcccgtagtgataaccgccattatggtggaacttgtagtgtaaggcaagactagttaagagtccgagccggacactcttatgagccggccgggactctgagagccgctgggcgtcaacctctctatataaaagggcgacccggcggcggtttagggacaagtaagatctcgtcgagagccaggcatagcgattaagctctctggtcatcgaaaccatagtcaataccacctcaactgaacgtaggcttttaccttcaccgtaaggggccgaaccagtataacccccgtgttccttgacccgtttaacccctttaagcttcctagctgcgatggctgcacgactaagtcctaactcgaggacatctgccgtgacaattccacgacaagttcagtgcataaacaagatttacaaagcatatcaatatgtatttcattatgatatatgttacatcaaagaaacagcttaatccatgtcctcaacttagcaacagttattacttgtcaaatacagaagaaggaacaatttatttgtgacaaagagcacacatgcaaagtatatatataacacaattactcgagctgaaaagaaaaaagttgcggaactaaacataataagtgagatatctaaagctacccttgaggctcctgctgaccatcagctccatgccatgcccagacaaatgtccttcagtgctggccggatgctctcctccaatagcttctggtactccagtgaatcaccgcttgtttgtttcatctcaatgagatggtacgaggggctgatctcaaagatctccgagtcaaactgaagaacaccattcctgccttccttcctcacttgcatcttgacaacaccattatccttcttcctcaccctgagattcagcgtcttcgcaacagcttccagcttcgagacaatggctgaggcaggctgttctgaagtgaaccttgcttcctttttgctctccttctcgacgaataggccagagaggtcaaaccctgtggagaaagagatgatttcaaaggcatttaaatttgtcaccgccaggggcttcgcgtcttcgtgactgttcttcctgcgcctcacaccaagtgttggaacagcatccgtgttggcattctcacttggcagtctctccttcactgtaaggttctccccgggacctttccggaaccaggtggactcctttatcttctggattgatatcctcttgttcggatcagggtccatgatcttgtacaacagcttctgaagtttttgggaaacccagccagggcacctgaaatcgcctttctcaatcttccggtacatctctatcaagttctgaccttggaaagggaggtaaccagcaacaagaacgaagaggatgacaccgcaagaccagatatctgattttgcaccatcatagcctcccttgctgatcacctctggagccacatatgcaggcgatccacaggtggtgtggagcatgccatcttggctcttcgattctgaaagtgcactcagcccaaaatcagaaaccttcaggttttcattctcatcaaggagtaggttctcaggcttcaagtcccggtgatacacgcctcggctgtggcagtaatccactgcactgatgagctgctggaaatacttgcgtgcagcatcctcggtgagcttgccagccttggaaaccttgtcaaagagctcaccgcctttcatgtactccatgacgaagtagatcttgcttcgtgtcgccatgacctcatggagctgaacaatgcttttgtgcgctaccaaccgcatggtggtgatctcacgcctgacctgctccgcgagcccgaccttcagcaccttctccttgtccaacatctttatggcgacgcctcggtttgactcaaggttcttggcgtagtgcaccttgccaaatgctcctttccctaacaatctccccacctcatacctacccattacgatcttcccactgttctccatgccttcaagagtataagtcacggccactaatattcttgtggctaccacttaaatatgctacgacacttcagccagcctccccgaagtcatgatccagcactctggtggattcgtcatagcggagtctgaatggcgtggcgttgatgcaggcaaggtggttgctcacatcaagaggtgaccaaatgggcccacgcagagtggtgatgttcttggtctcacagaagagatctcttctatgcaagcacatctttcagcattccttgagactttgatttcttgtttttgacgagttccctgaaaaagaagtaacattcagatttggcagtcacagatttaataatatgtacataaagataagtactagtatgaatgattgaagagaaaagtagagatggatggaaagaaaatcgaattcaggaataagttgctcaggagcaaacaaaaaaatattactactaggactgcatggatttcagagttcaaagatgaaaaatatcaagttattccttcatcagcagcgctgaattgctatgacttctgaaaccctgattacctctaacaaactaggtatcgggagaagacggcgcctaccaacgatccggcaagagggacagatcacatgatgagattaggatcccacacgaaagcaacagagaaaaactgcgctacctttgtgcatctaatgattcgacacctagcaggagtcttccagataaacttggtagaatttcgaaatctgaccgcatggggcgcagatgattggaagattgtactacctcccggctgaaccgagagaaaaccagacaacgtagaactccaaaaatcaaaatctgggcgagctagcgcggaacagaaggccccgggtttccccccttctccaatcaatgtgcccctgcacccaccggcacgccagagattttcttggctctcaagaagatccatcgccatcacatcacaacgcacaacagcgatcgagaaaagccccctccacggacggtcacggaccgacggcgaaacccaatgcaggcgcgcgaaagtaaaggaagcggaggcgacagggaagagagctagccgccgtaccttccggcaccgccgcggagacgaggagacgggagccgagagacttgcgccggagcgccacgcggggttctggttctctccttttcttggggcttgcggggctgagtcgggttcccagtgtgttggggttgtgaggtgagcggcctccggcgaccccgcgttttatatatcgaggctcgaggggcggaggaggttaggaaaggaaaggagatgatgagatgaggacaccacgtggacgccgacgcggtatgaccagactgcccctcccggcacggcctgcggttccagggcaccgcatcgcaccaaaggcatattccggttttcttttctctcgctggtcgttttaatccaggaatccatcctcgcccatcgatttaaatttaaataaacacaaaaacgaacaaatcttgtgctccatcaaggccacaaatactacaagaaataggcacaaatcttttgcagttactactccaaaggaaatctcgtaccccatcaaggctgagtattctcttttgcaatttattttacaagaaatatgcacagatacaccacctggagccgtgtcctttttggtaattcgtagccaggatagatggcgtctcagctgacagcagggcgcagtaagtaaccagctaggacgcagccaacgctcttttgtttttctggcgatccactcgtggcaatcgaggccgccgctcaattagttaccttcccatgcatgatgcatgtggccgtgtgatttttctacgtgattgtgatcgcccttatctttatcctatgcattgctgagtcaagggcagcctgttccttggaccagtcatgatgccgtgttacatgtgatggagattgaagaaccaaccatatagatggatacagtcccatcaggaaaaccatggatgccccagcccatagcataggtccaattggacatacccatcaaccagatgcacatggacaggcaggtcgtgaattattgagtgaattctagtttctaccacctactttgatcttttatccctaaaaaaacctactttgattttttacgctacttacctcgtttaatagcatttcattcgaactgcccacctaaggaaagttttgccggtttttaccatggtttctctcgtggttcttttgccgtacaaaaagcgttgatagggctatgtctggtatgcgccaatgaggtggaaactatgaacaagaagttgtgtggcgtgaagaagtaactcatcaaggcaaagcggtgcgtacatacacatgatgaccgtgagaaaaaaaagtgtgtttgactaagccatttggccagttcgggcatggggaaaaatgcctgaaggaaaattgcaacgtggcacgacacgtgtacctgatgggcggggctcatctaacagatcgctaaataggattaaaaatgtggcaaaactttcgctaaatgtggttaaaatataaaaataacctaaatggggtaatccataaggaaaaatactaaatggggtaatatgtgtcaaaagtatcgacgtatgaggtaaaagattgaaattcacacttaattagtactccaccttatgttaccttgagcagagtttttacaatataatgtataagggaaaccatatatgtgaaatttgactaaaggttgttttactatgctgaaaattgtagggattttaccaagagccaagactacggaaaaatatattctacgaaacagtttgcaaatgcactcttatggaacttcctacatttatggtcattcaaaaatattttttgaattaaagacgtctgagcataattcaactccatggaaaatgaatgtataaccatcatgaaggaagtccctcttttaggaaaaaaatggaaaagtgattaggatcgaaaggaactgaatggcattttttaaatggacaaaatatgacacacaataatggatgggtgacaataaagtcatggcgccacatgcagccatcatcaaccatgaacaacaaaccatcttattagtacatctttttgaataaattgcaaggaaagacgccagccacacttaaattctgtctactatattgatgcggaggaaagtggtccatattatccccttgatgattgcaaaagaattgttgatgacattgtgtagataggctactgatgtcgcaaaataaagagtcaggaacatttggtcccttggctatgatgtgattttgatttatatctaccaccacaatggatagaagtatatacgaaaacatcgaaaaaattaatacaaattcgtgaacttcaaatttggataatgcttaacactgcacgtgaactgcaacatgtatcgatttagatattggcaaggttttcgaagtaacatttttaatggaagtagactaagtaggttgctaaaactgaggctaattatgtacgcaatctgaaaaataatactagtagtatttcataatagatcttgtaacatcgatttgatattgcagatgttttcgtttatcatggtcaagccccaaaacatgttttactagcacacattctaaatcaacgtacactgactcaggggagtatataaaaccagaatgtactacagaaaccgaacaaaaaaaaggagagaaattattgctgcatgacgaacaggaaatcaatcagttacctagtactccctctgtaaacaaatataagagcgtttagatcattactttagtgatctaaatgctcttataaaagtttacagagggagtaatttagtaccattatcatctaggatttgtcttcagtcaagttcaaggatttgatgaaaactacattcctgtagctaaacctgattaaaaggacttgagcgagtgcgccaccttatcttgtggtagttgtgatgttcctctagcttcttacgtactgcttctgaagttgtaattctaattttactctgctaaatcttgttttcaggagggcactgaatctagttcagaatcatgtctccatccattcgctgcaggttccatatgaattttggggcatgtacagagagctcctaacctggtccaaaattatgtctacattaattcatctctccaccggctggaattctggccacattcatctttgctctaggcgtccaaatgaacaaacttagagtatcttctacaaacaatttcttaactccttattataaatataagcaactttagctatccagtaagtggcaatacaaattgcttaatttgtctgggaatttgcagtatatacatgcccttttacacttgttttagtgatctttgatattacctccatcccaaattacttgtcttatatttgtctagatacggatgtgcctagcactaaaacgtgtatctagagttctagacaaatataagacaagtaattcaggacggagcgagtatatgtgaagggtcctgttttttcagggtaagaaggggtagagaaaatgcctcgagccgaggtatacacttataaaccgaaccaaagggagtaagaatttgcattattttagtgttctctgatatctccatacccctttccacttgttttagtggtctctaatatctgtgcaagcttacactcccgg

>TaCIPK16_5BL_10860020

aattttttaaatttttctagtatttttttttgaatttttttctaagcgtgggtgcagatgagcccgggcaccgttttgagttttccctgatttaggtacatctctctctccgtctgaaaagagcccacacgcagtttctctcctgcaagaaatgcccacatcctgaacctgctcggccggacaaaagctcccggagcagcgcccattccatgcccaaactgcatcgtccttatccgcggaagatgcctgccagtgccagtggtgcatcactttgctcaaatacaatgcaccctactgcaattcattaggaagatgtcaaaatcgacgacgagctgcacatgcatttcgacctttccccgctagatacgacgaacaggattaggattaggattaggcggcggaagtgtttggccaatctcggatgcaaatcgggattaacgcttatccggaacatagcaaccggagacgacccggtgacactgacaaagcaagttaaccccgagatctatcgatcgggagcgtcgctggaaactgggagtgcagagtcagcagcaccgccagaaccacgtaggtgaaaatgacaagacagaagcttcaagagcccgaacagcacacatagcagcagtcaaggggagtccaataaaactaaaacacgtccgttcacctttttcttggcacattttctcccctcctgcgtggagctttgacgacgttggcgccgttcaggtacgtgttggtgcagcatttggtgtcacggtcgctgggagttcaccagtggcacgcaacgcatggtggtgacatgggagcgccaaagaggtgaatgaacagcttgcaacagtggagcaatgaaatttaatcgtcatttcattgattgcattttttgttggtagcttaccaagctggccgcttggtaatttcattgagtataattttcgttggtagcttatcaagctgaccgcttggtaattttgatcgtcatttcgtggagtgtactcgggcgtctgagctctcagagatccttttttctgtgattaacgtgcttgaaaggaaaactcaaacactaataaggtccaaatgcttcccgggacacctattctaaaacgagtgttctaaaactaagcgagctcgactcgactggaatcgaattgatggctaatcaagagagttggctcatattctacccacagcatttgagctaatcgccaaacatacaaaaataaataagtggttagtgagaaaggtgtgctgctcccgccggtcttcggcccttgtcatcatgtctggcttaataatgtaatattttatctgataatttattttggcatagttcaatagtactccataattctaacataaatataagtatctattctgctgataccgaccgcgtacaagcctatgttttccttaggcaaaaatacacaatgagtacaattattagggcccctttaaaaagtagcaaaatgattgcaattagaaatacaactattagggtccctttgcccttaattaaaaggagtatggaggccaggcactttgaaacgacaaaagggaagacttcatatcagttgttgggcatttttttccgcccgtgtttgttgggcactttcttttagtcatggtttgttgggcatcttcaccaaatcaaaaacaagccttggggcctagcaaggaggcaaagaaatctattgcagaaggattggctttcaagatcgaggaccacaaaaaatggttctatatttaaaattagggttttaatgcttatttgttggacattttggtgtgagtgaaatgttagggtgctccaacacaagtccacgctactgtctatcattcactccaacatcaagaatgaggcatcgctttgggtcaccgtcgtcgcgaagcacttgggaaacatcatgccacgatagtagatgcattgtgatttcatttcacttttgtttgtgaaagcaactccaaactcattaattaatggatataacactagtctatattactactaacttcgtagtgcaaagtgttactatcatctattagcatgtagactcaattttttcaggaagtcccatgtgatgtaacataatatgttatcacaaacatagctttgttaactagctgccacataaacaaatttatcttggagtaaagttatttacttgctataagttactcccattatgaatagcctaatcctcttgacccatacaaaatggcttaggcaagagcatctacagccggaagccttaaaccatctcccttatgcccgcggacgcgcccggtcagtcaccggacaggagagagaagaaaaacatggcccaactaggcccctcatatgttccctatgggcgtgtttggatgactgcctcgcacttggctcgcatccgcgatgcaaatttcagcgtgattggttacctgtgcggcctcctcagcctggcctgatacatgcaaaatgagccctctcagccaggctgaggggaacgcaagaatccagcatattcgggggccaggcctgtgcctgcatccatgcctcaaccgcaacctctcattctctttcctttttctttctctcacgcatcgactcgagaaaccaaccaccgccgacgtcgtctaagccccgcatcgtagctcggagctcccttgcccgccgccggtccaatccctgtcgccggcgctcctccacgtccaaagcgggcgctcgcctccccgcctcgacatcgttggtttccgtagtcgcctcaacaccgccgaactccgccatagccgtgcgcgcctagccctgcggacgagctcgacggagctcgtcccggcaccggcccaatctctgccgtcggcgctcctccacacccatgccgggggctcgccttcctgcctcgacatcatcggtttccgccgttgaactcctcaatagccgcgtgcggcctgccctgtggatgagctcgctggagctcgtcagcctaccctccaccgatcgagaggaaataggggtgaggggagcatctagcgccgtgagccgcaactaccatcgagttcccatcgggcgccgcttttctccgccgtttgagctttgttggttcagatttcagtttaggctttgattggcttcagaaatttgtgataaataaaagggagtgcctagaactcatctagatgagatataatttggtctcattcactttgaaaacaagaacaaatacaacccacatcagcatacacgcatcttatagcatcacatccaatggctataaaaggtgaatgagaccaaactatatctcatctactcccttctttccgatttatagggcttatctcaaaattttagtttttccattttataaggctcaatttggttgtttctcatcacatgttcagattccaaggtgcattaaatcattgcatgcaagtattaagaaaaaattgatcaatgcatgtaatttatgcatgcatgcattgcaattaatgcattggtaaacatacttttttgaggataacaagagcattaattgggtgcttttgcaaactacaaaaagtatttcaccactcaccatctaccttggttggtgagatttttgaattgagccttataaaccggaaaggagggagtagatgagttctagcaaaactgtaaataaaattacaacttgagctctgcttcagatttaagtccgggcttattttacttgttgttttttgtccatggaaccatccagattcaattggaatgcttgaaattgtaagagaaaagttcatatataattaagctcaaatgcagcttaccaaacaacatctgttgtatatggcatctctcatgcatacatatcatgcagcttaccaaacacacatctcaactcgtctttgcatcggctcaaacatgccacgctcacccaggatcccccatgccatgcagactagagccatccaggtaaccaaacacgtcctatatgtccgggttgtccgcgaaccctaatatccatctcaaatatggaaaggatatgagggctcgcggacacgcccggaagcgcccggacaatccgccacataggacggggccccatcccagaccacattttctctttcgttattcattcttttatttctctctttatctctatcaatcgtgtgcaagtgaccggatatatgagaaagaaatgaggattgtggctgcgcggacgaataaacagggactcaaaatggacatgccccatcactaaccagacgtgtccgcgatgtttggagggtcgaattagaggcacgtggctgaagatgctctaagctgagtaatcatagatgcactccacttatagcagaacaaaaaggcagtacgcagcacgattcctagcagttcattttcgtcacatcaactcaactgataagaatctgaaatatagagaggaccatcgaccatgtgatcatcgaatcgaatccccgggaacaaaagctgaattgatcaggaacaaaactttgcatatatgtacacatcgatcgccggacgtatacatcacgcacaaatcctcgcaagtatcgtcatctacaaacacctctctcttcaagttgctctgcacgttacaccgagccttagcactctagtcagagttggctgccccgaagaaactagtagcactaaaatttacagcccaagctggtgcaccgatcgggcaagtcctacacagcatcactaatttgccccatttgtatgcacagacaaaacgaacgccggaacggcctgggatcacgcgacggcgcccggcaaggcgggcacgtcgccctgccacgcccagacgatgtccgcgagcccagggcgcacgtcctcggcgcagaacttgttgaaatcgagcgcgtcgccgccgtcgtgcgcgaactcgaccacggtcacgtcggcggccacctcgaacacctccgcggtgaccgcgagccgcccgttcgcgccgtcggccttggcctccattctgatcctccatcctttccctctggtcgtgttgtacctcagcgccttgcccgcggactccagcttgtggaacacggtggccgccggcgcgcgggacgtgaacaccgtcgcggccttctgctcgctctcgaacagccccgacaggtcgaacccggaggacatggaggatatgagctggaacgcattgcacgtccgcggcgagatgttgccggagctgtcctcgctgccgtcgatcagggcggcggcgttgtcgtcgtcccacttcctgggcgtgacgggggaggacgggacgggcggcacgaaccccctcttgaaccacggcgtgagcatgatctccgggatggagatgcgcttggcggggtcgacggcgagcaggcggccgatgaggcggcgtgcctcgccggagacccagggcggcatctggtactcgcccttgaagatcttctggtacatcttgacgtagttgtcgtgctggaaggggaggaagccgcagagcaggacgtagagcacgacgccgcaggaccacatgtcggcgcgcgcgccgtcgtagccgcgcttccggagcacctccggcgcgacgtaggccggggtgccgcactgcgtgtggagcaggccgtcgtggcgcagctgctcgggcagcgcggcgaggccgaagtcggtgaccttgagccggccctcctcgtccagcaggaggttctcgggcttgaggtcccggtgcgcgacgccgcggctgtggcagaaggcgacggcggcgacgagctgctggaagtacttgcgggcggcgtcctccgtgaggcggccgcgggcgaccttggcgaagagctccccgccgcgcgcgtactccatgacgacgaagacgcgctggcggctggcgagcacctcccggatgccgacgacgttggggtggcgcaccatgcgcatgatggagatctcccggcgcagctgctccaccatgccgtccgtctggcggaggcgggccttgtcgatgaccttgatggccacgctctggccgtcgcgcaggtcgcggccgtagtagaccttggcgaatgagccctgccccagcatccgccccagctcgtacttgcccagcaccaggttcctcacctcgccctcccgccctctcgccattgccggctgctctgccacgaggattgctcggaagttgttaacttgttttggaagttcactaatcactaccactgctgctgctgctgctactcgtctgggatttaactaggcggggttggcacgcgtcatggtggctagtggcgctcggcacgcagagatgtggcgcgtagatgggagcgggcacatgggtttgacgcgggcgttgctggtttgaggagggcggcacactggttcggcaccgtggggtacgccgcagccggtcgcttatttgtactggcgtgatggccgagggcggtgtggtgcggggcggtggagaaggagaggtgtgcaggtggctagcaggctagctgggtcaggggcgtgtggggtcgggtggacgacgcgccgggcggtgcaggcgagaggggacaaatgtgaaggcgtcgaggtgcgaggttgggagtggtggaggaagagtagcccggtgacggcccatcagtggtggaggggggtttgttgcttcaggttgcagcagcacggaaggtggtgaagggggcgtgtctgccggctaattgggccgattactatacccgttcaacgtttaacgaatgcggccagacatgtgaacttggttggaccgggcatttgctttgcacgaaacacccggcagccttccgcggcgtcaacggtccaacgtccaatgaatcttcatcttcgcgaacctcttaaccactcttttcctagttctcaggaaaaaaacacaatgttttccttgccaattctattgaagccactgttgaccctcctcccctaactgccctttgaggatcccttgaccgaggaggcacatcgttgacctcctatggccccctctatggttgctaggctatggtcaaactatttttcaaacggatgctttcatttccgcttctctctccaaccctttttcaagagtcaccttgagtccaatgatccctctttactgaggtgttcgactcgttaaggctcaactctttaagctcgcgattgtcaaggctcggtttgtaaagctcggtacttataacaagataaaattcatgatcaactcgattattttaatgctcgttaagcacgtgagcactcgttatagaagtgcacgactcaacaatattacacacaatttataatgggctactcattcgacttcgactaggcaagccaagtgtaaaagagggataaggtggccgatgggcaacatgaaattggggttactaggctaagcggctgagccttggcaattaatagcaataatggcttaatgggtcccccccccccggnggtgaattttaacaagcaaaacgagttgctcatgaaacttgcgagctcgctcgttacgtttgttaatcttagcaagttacatttgttgttcggcttggttcattagcaaacgaacacaagatcaaatgagtcgagcttgagattgataggtaagctctcaagcaacgagtttctcgtccaaacctatccactcctccctcgacgatggtcatggtgggaatgggagaggtggttgggacatcattgtggttagtagaggtagtttaggtctaggttttcatttgactccatgatgtttggcgacatgtcatgtcaacacttcatcttagcatttgtgagctacacttgatcgacgtcgggttgtttattttggttgtcgtccatggttgggattggcatgggctgtagatccgacacgagtttattcagtaagctcactatgccaccagtgtagctaaacccagagtcataatcgctcctcccaccgtcaaggtggcgatgggtgtgggttctattatcatcgatcaagtgttgtttgaggtagctttccgagtgccttgttcaaccctgcggggaggcctttgttcccttactcgtcgccatcaaggtttgcatcagagtcggtggccttagtgtctctaatgaggtcattgtatgtgttactacccaattgtggtcttggtgatatattcggggccctatctgcaaaaaggagggacctagctacaatttctgtttttagggtattgtatgcaattttgtcaaaatttggctatgaaatgccatatgttttagcaaaaggttatcgcccggccttaaactaaggcccttacaaggttcaacaggttttaagggaagagacggtggcctaagtgtctccaatgaggtcattgtatgtgttattaccaattgtgttcttggtgttatatccggggccctatctgccactaggagaaaaagggctattcgaactggtttgtgagggccatttgtcctggttcacaaactgggactaaagggtcgtatctaaagccctccacctttagtcctggttcgatcgggaaccgggacagatagggctccacgtggccgtagcggcttgcccaggcagggggaccttttgtcccggttggtgccaccaaccgggaccaaaaggcctccacgcgtcagcagttcacggtctgggtttgttttt

>TaCIPK17_1AS_2298229

cttatatttgtgaacagtgggagtggttttttttagggatagcaatagtttattttgaatttttgcttccctagtttttttgatttttgctagtacctcacggtcgtatatttattttagaatagggatccttctcatactcttgatgtattggttagaacaagtggagatgaatggcgcagataataagaagtaacaaaaccaggtcaacaattcatctcttatatgtcaatgttcctttcttcagcttttttttcatttacatcgccgacagtcagttttcaccaatcggttatctgtacatctatacactatatgatcctaatccttatccctcaccgtcatttccaatccctgaagagctatgatcctaggtcctacaacatcttg

>TaCIPK17_1AS_2417639

cctaatccttatccctcaccgtcatttccaatccctgaagagctatgatcctaggtcctacaacatcttgtcagtgtgggggtacaacacaacactcgtcacaaggcaaccagtggcgtcgcgcctctgctgtcgaagctggggagatcgtcgtcaaagagctgctccatcctgaatatcttgtcaatccccaggtcacccgagatcctatcgcagagctgcaaaaataaatttcaagaaagcagcatcattaagtcattcactcaataaccaccagcactcaagatgt

>TaCIPK17_1AS_3259469

ggggacatgcttgggaaggcgcttgcgcgaggaagaagaagtacagcagaaaaaaaaatgttcgcttgaggcccacctagcgtgcgtggaaaaagagcgagcgagcgaggggaaccggccgagcgttcggccggtgtgccgattccaaacgattaccttgttaaaattgannnnnnnnnnnnnnngaatacttagaaagacctgatcgagctgggttacaaaacattgaccggataagagttcattctcctttctgcgggaggataggggttcatatctaaacatgatgacctggaacaatcaatatatgctacaatcatcgaaaaatgacagcttattaaggagagtgatgtgtccgccccagagcaagggaggctcaaactcgggcaggtcacaactcgggtgggtgacaacttattgtcaagtcctaaccatccggggacctgggcatgcgaccggtctgcatcacaatacaattatcaaccgcagattccttaaaaaaaatggtactcctgcttggcgatggagattgcaagttttcaagaatcatcataatgtttatctatatatgtttgcaaagatccataggaacacttctataattgtgggggttctcttttgaatattatagtagggtgggctaatagcctgcagatcattgcacgtcacaggtagagatcattagcgcgtagcacgtggagttggtccagttggctggatctagttctagttgcataatctttgctccttggtcaaccctgaagacatgggctagtggtcccacagaggcagattcagctccataccacgcacttctccatgctaagattaatccttgatttggaatgcatgtgacgtgtcgccgtcaagtagctgctgccagccccgtccttatctttttattattttctactactatgcaatgatctttgtcttctgtgagatataattgaaatagaattgttttaggtctaactcatggtgcacataagcatatgaattcgttcacgtgggccgtttaaataacatcattgtcgtattattaatcctgcgattgttttaactcttggtttcaggttgctgctagcaaaacaaagatctatatggtgcttgagtttgtcaatggaggcgaactttttgacaggattgtaagcccgtataacttttatttttgaattgtctgaaaccttgctttgcactataaaattctctatcaggtacatttcttagttctaacacttaagttgattcctttcctacaaataaaaggcaataaagagaaagctatctgaacgagaaggaaggaggcttttccagcagctaattgatggtgtgagctattgccatggaaagggtgtctaccacagggacctcaaggtactgacgactgaagtatttctttatttggagcatctgatcaagcaattatcaatcattcttattgttcttgtattatataatccgcgagatggccaatatggcttgactgttctgtgttctcctcatttatcccactgttacatgttacagcctgaaaacgttcttattgaccggaaaggcaacatcaagatctctgattttggtctcagtgctttaccccaacatctcggagtatgacagaaaaacttatcacacggttttcttgtattgatcattacatttttattgatatattttccgagaatatttccgtttcactaaatttaagatctgcaacctttcagaatgatggattgctgcatacaacctgtggtagccccaactatattgctcctgaggtaaaagactggccaccttcagaccaattgtctattttacagtatgcttgctaattcttgtgccaaatatatccaggttctgcagaacagaggttacgacggatcattgtcggatatctggtcttgtggagtaattctttacataatgctcgtaggacacctaccatttgatgaccgaaatatggttgttctttatcagaaggtagatattcataaaaaaggaaaataagtgatatttagtgttctttctgttatgatagttctctcaagccattccaatgatactactattatcttgttgcgtccagattttcaagggtgacgctcagatcccggagtggctttctcccagtgcacaaaaccttcttcggaggattcttgaaccaaatccgaggaagaggattaacatggcagagatcaaaatgcacgaatggtttcagaaggactatattcctgttgctccatatgatgatgatgatgaagatgtacggcttggtgcaattctacctatcaaacaggtatggcaagatgacacaaactatatgatgctgaagaccaatttatgtcctaatataggcatcatgtgttccatatttgtagccgctgatcggtgaatttttgtttctgtttttctatcctgaagcaaattagtgaagcacccggcgacaagagtactcatcagatgaacgcttttcagctgatcggaatggcatcttcccttgatctttcaggtttatttgaggaagaggtatgcacaaacattttccagtgcctcccttactgtcttcagagtgatttggtctgcaaagctgggaagaaaaatcatatgcatggcttctgaagataaacactacatattctttgatcaaaataatcactgaatgctgccactacggtcttagacaacaactaatctgatccagctgaatttgttgcagggagtgtcccagagaaagatcaggttcacatcagcacatccaccgaaggatttgttcgacaagattgaagtgtccgcgacgcagtcagggttccatgttcagagagcacatagcaaggtgagcagaagcttctcaaaaggtttttcagcatcgcatgattgagtacaagttttgaaggaactgaaaatgatgtgtcgtgttatatttcagctcaaaataacgggcaattgcaatggaccgaacaaccccacaccattcttagtctgtgccgaggtataaattcggcgagctgaacctttcttctgtgaaacgcgtgggaagggtaaccaatggagatcaactgttaacatctgtggtgctgtttctttttcttgtcccaggtttttgtgcttggcccctctctgcatgttgtggagcttaggaagtcccatggtgacactgcagtgtacaaaaaggtagacaagagcgctaacaacaactggatttgccacagccataacttcaccgtacacatattcagtactggtgcttagtgagtgaatgacttaattgtgttgctttcttggaatgtgttttgcagctctgcgataggatctcgagtgatctggggattgacaagactttcgggatgggctcgctcttcgacgacaacctccctggcttcgacagcagagcaacgacaccactggttgccttgtgatgagtgttgtgctgtaggtaccccaactgacatgatgttgtaggacgctaggatcatagctgtttattgattggaaatgacggtgatggtgtaaggattaggatcgtacggtgtacagaactatatacatatagtcaagtcgtgaaattgactatcagtgatgtagttttttgcacgaaaaggccaaaggccttttatatgaaagccgaccaacctttacaaggatttctttacataaatgaaaaactacacacagtcttcctcctgcacttgccatgagcgaagctcgataccccctctggacattccaaacaccatgagagttaggaaaacggtgttgacgcagacgggaagttgcctgtcggagccagatgatgccaacgactacaatctaggaaatagacaatcgtcccggagaagaaggcgatgaacatggttggatgaccaccatagatctagccaaacagatacagggagacataacctcgtaagcacattgacgatgctgaaactggccgaatgttgccagttggaggaggagctaggggccaagacacgatgcgtccgtccactcagtcgaaagatacattcgaagaccacctacataacacgaagagatgactagacccaccactgtagaagagtgctgaccaggcgaccccttcacccgccgccgccgccctcctctactccaccgacaagatcgtcgctgccgaggtggaccaagattcgtggaaacctttttcttgatctgcacggtgccaccatcaccatcctagtgccatcgtgtgtgtgtgngggggggacttctaattctaagaggcctaagtacaatgtcacgatgtagattcggggggtgaccctcccgtcggtagagcaacatctggaggatgggactcgtggcctcgttgacggggagcaatcgcctcaacggtgtatgaagagggatgcctcttggaaaaggtacgacttgttttcagcgacggagttcaaaaataagagatgaattatctacatgccttgttagggcatgcagtgcggtgtttagccaacataagttaggtatgcccagaaccctgtccactttggccggcataccatgtcctaggccacaacgcagcttcttccattggcatcatccttgccgcaccacaagaatccacaacatattttggccatcgccgttagcgttttgggtggcagttccattgccaacattgcctgaattgggatggcgcagagactggcgtaatccggagtaaccttccactcttcggtaacatgaccgccttgcaattgggcatgcatcatgtaagttgatccacagtagcttcaagctgggttggcctttgcttgcggatcgaaagtggcattcccaagtacttgcaagggaaagaggcgttcgcgcatccgagcagtgtgcacactgaggccatctcctcctgggaacacctgacgggcaaggtagcgctcttcgacaggttgacagttaggtcggaggcatgcccaaacaaccccgatatgagccacatgccctagtctcatgttgaatgggcttgaagaaaatcatgatgtcgtccatgaacattgataatcgctggtgcatgcccgatctggctagcggtgcgaacaatccaccccgatctggctagcggtgcgaacaatccacgggaagtaccagcttaaacagctacacgaggataattggatcagatcggtcgcccgccttcagttccccatgcacctgcaggtctcgaccgcatcactgacctctcaggttacactgtctacagacagggagaactccatctcctggcgttggactcccaatgaaatctactccgcagcttcgacatatacaacccaattctccggctccttccccaggttctcgtcgacgaagatttgggaagcacacgcggagcccaaatgcaagctcttttcctggctggtgctccatggcaagatcctcatggccgacatgctcgctattcgcggatggccgcatgatcctacatgcccgctatgcctcggagcaccggagacgacaactcacctttgcaaggactgtccatttgcgattgcggtttggtcgcatgtccatgcatggaccggcgaggcgccgatgaacacgatgacacttccgcctcccatgggagtctccgactggtgggatctgctcatctctcccctatccaggtatgaaagacgccgacgtagcggtcacttcctctagaccatttggaatatctggaaggagcgcaaccgccggatattcaacggaacccgtctcactcacctggaggtggcggccgtcgtgctcgatgacataagactgcgggcttgcgccttcggcggggcacaagtggcgaccgatattagttgatttggctcacttagcgtaacatgccggtccatatgggggttttgtctggtttccctggaaaaacctgtcatctctgtacataaaccctctttttttgtctgaaatgaaaaggcaatgctcctgccggttcctcaaaaaaaacaaacagctgcaatggtttcatgcatagaatgaagagcatgggagaccacggatatagcacgagtcctcaatattgtaaggatatatttgcagaaataacattgccatgtgtcgacaaaacaaatgagttggaccagggaaagtatctggtgctcccagccttggggtacttccggtactccaatgtcaataaacatttttttaatgtttcaaataattccaaaaacaattgtgaatgttcacaatgaaggtgactaacaagtcctaaaaatttcagatccaaactcgaatgacacattgggaaacaaaaatgtgaaatttaggctgaatagtgtaaagaagagataaaagcaacattgacactattcaagtatgtatttctcttttttttctaaatgtacatttcaatgttggacctaaaaattttaggggatgtagtgacagtccttgtgaacattctctctttttttctaaattttttcaaaacatttaaaaatactttttgacattgaagcatcgggagtaccccaaggctggaagcactggatactttcccgaccaagtatgaacccaccggtagagtttcttttaccgtgggtgaaccatattcaaattttagtatggatctgttcccatctgccaagtttatgtgcctgggccgagtgcaaccaccacacacacattttttttgcgggataaactttcgatctattcatttttaattatgacagtacaacgaacactagaaataatctgtcttagtttcgtcgccttacctagtgatagtacaccgcgggtgcgatttgttaatccgatatagtagatcatagacctgtcgacatgtaactgatggatggagaccagccgacccacaacacatacagactgtatactacactcattcgaggtgttgtgttttttttccggctagttgtctagcaatactccgtgtttaaacaagcacagtactagcccgcgccgcgcgcgtgcgatttgttaatccgatactgaagaatccaaaactgccgagacgcgactgatggaggccggccgtcgacccgaagctatcaaggagcagacgatcgatccgatcgaatcagtcaagatgtgtacgtccggccctggctcaagatatccattcgttcaccggctcccggaccggtaggtgcaccggatcacccacgtgcactgggtcgtgcagcacaccggatagccacagggaatgtgacgggtgtccggttgaattcaccgccgcatggtgaccggcgagtgtgtgggctagcgcgccgctgcggtgcaacgcaacgagcggccatcgcggttctgacggcaacgtaccgggcatagctacatcactaattaactactggccacttgctgcatcgcggtcttctgtttctgaaagcaaattttgaaaatgcagaggcgtagagctctccggtttcatcgttgacggagacgcggtgcagcgtgttatgtctcgttgcatgcgccaagagctgatttttgcgtgcaccctaaactcctgccatgcacgccgactcctcgacctgtcctagaggtaccgatctacgtatcatgtatcgtatgcatataccccatggccacgtatatacgggagtgacgggaatcagaatatatagtcatctccgtcgaagaagaaccgtatacaaaaaaggcccgagactacagagggctcgggaagaagaagatgcactggaggcttgcatcaaccaaaagggacagtttgtcccttctgaaaaaaaaaatgaaactccatgggcactagcacccacggtttattgatatagaagaagcatccctcatgagaattccagaaattcgtccccgacgtggatcgaactctggtcgttgggtttacaatcatgcgcccccaaccactgggctatgccctggaatcctagtgaccttagtcacacggaagaagatagaagtctctagaagatagatagggctgagggcgtcgaggagcaattggaagcatctacgtttggcgtaggggatcactgagagtgagaggccttgtttacaccggagtgtgtaggatatgcatcttctcagaacaacacgataggatttagtgcatatttattttgagaggaactgatccgtacggaagtacgtacacaagatcagatcatctgaataagaatatgttagggaggtcactggcaagtcttgtactatacaccaaagtggtcagggacatgcatatcaacatgcgaataatgaaaccaacatgattttgttgattcggtaatgactaatgaaagaacaaaaatatcaaaataagcaaaattaataggtacgtattaaagatttggaatagactcgaaagctacatcaagattcttttaagtctacatctgtagttccattattcacatcatgatatttctctatgcaataataggtttttatggcctagtttattaccatcatagtcagactcaaaaaagtttgactactccatctttagaatatcatagtagcttttgccttaattaatccatgaggcaactttttggcctccaattcagaaaagaaaaatgttagcaagccaattcgtcggctgtcaaccaagcatatatgttggtaccgcatgtatgtgtgcttggttcttcatgggtattgaggcgtgtaatgcaataactatcaacttttatcacctttcagtcaatcaagtgtcgactcgaaacgaatactaccaaaattgttgtgtatattgctagtccgctaaaaggaatacccagattgttgtatatattccttccgtatttgcacgcatcactgaactaactagatgataacccaaacattgctacggaaagttgtgaattggatgcaactgtttcagtgcgatttggtttatgaaacattactatttaaactgacaatatgtgtactgaaacaaaaatacatatagtgtgttccaatttatcattctatagttgtataatattttcgaatatgagtagaatgcatggtgcattttaatgagggtatctttccatgcatgatgatatggtgaggtgatatagttgcatgttaagataattgaagttggtgttgaatatgattggcaagaacttgcattttttaatgcgggtgtttttcatgcacgatgacatagttgcatgccgtgataattggagttaggaaatatccaacggcgacccgcaaattttctcccgtgtggctgttattcaaagctgcgcgcatacatccgacaaacaattcaaactaaccgaacaaatttcgttcaaacacgcccgtattcatataaacatggtcagatttcatataaacatgacatacttcattacatttacaccaactaagcggtgctagtccagctctaaaggtataattaatacccaatctaaatggtcgctggcgcccgttccccgtgtctggccatgagcccgagaactgaagcttcgccttctccagcttcgcctcggcgctatccagttctgcctccgtaacccctgcctccggaggcccgggaagcgcagttgtctgcctccacgtcgctgccaagctgctaatcggccgacgatgttcaacccaccaaaagcttggcttcaacaggaggcgaggggcggtggtctttttgggacccgagcggcagcgacctactgtgcactactcttcctcgctgcgagcggcgcctgcggacgtgccggcttcgtggtcgtggcggcggggcgcggcctcggcgaagtcctccaccccgtcgccggtctcgccgaataccacctcgtcgcattccttgtaggcggccccacctccaccacctgctagcggtaccgccaacgggcgaggcggatatcgtgggcagagcggtaggactcaagcagcgcctcctgctccgccagttccgcgtcctgcgcgaacgccctactggcggcgagttgctcctccgccttccggtgctcctggaggaggtggtggttgtaggaggcgtcggctgcgccttccggaactgctcctcccgcaccatgtccatgtaatgggcatgggcctcgccgatggtcatggtgcagtgcaccgacgagggtggcgtagaggaggaggaggagggtggcgcagaggacgaggagggtggcgtcgactcgtcgccggtcgcgtcctccggctacctgttggccaggcaaccggcaacaatggcggccatctccttcttctggtccggcgtcagcccatcccagagagctttggcgggggaggaatccatggtgggggtgttgcggagagggatcggaggttgatgactgcggctatggccggcttcggtttatatagcaatggtggacggtaaagggacggacgtgtggcgccgaaggagagccccggtaactgcgtgccttcaatgtgggcggcagacagatggacgagcgtccgtcgtgtcgtttgaacgcgcaacagtcacctacacaaggaagaggcgggggcggcgctctctcggccggcgcgtagcttcaatgccgacgccagtgagcggccgtgtccgctctggccggacatgaatgcaggcactggctctctgaagcggcattgaccatttcgggcgggaagcgtgcgggagggaggagggttttttggtagccagagcggtcaaaagcgggcgtgtcagcggtacggactctcgcaaagcccccccccctcccaacacacacgtttgttttccgtttgcgggagaaaatacgtctgaattgcgtcgcggaccggtacagaaacggtttaggtagtttttgcggtcagaacaatacggtccggatggatgcatgatggtgaggtgacatagttgcatgctaagataattgaagttggtgttgattgttgaatatgattggcatgcatgtgcattctaatgtgggtgtttttcatgcatgaacataatgagatgacatggttgcttggcctgagaactgaagttagtgggatcaacagttaattgttttcttgcacagagtcacacagacatgacgtttgtattcggagcagtgtgtgcatgccgacttctagtatatgctgcatgcatgcaacatgcatgtaagcgcacgatcgagacaatacacggcaacagcatggtgtccatgatcatacttgacacgcactgctacgtctcaagtgaaaatgaaaagataaggtggaatagaacaaagtgtcacccactcttggagaggggtagactaagaaaattagacagcgcgatgcgtcttgctggggtcgtcacgtacctaactaatccagagctggtgactcatgagtaggcacatgcagcgagcacagtcttatcacttgtctttttactgagaggagtcgtatcacttgttgacggccgtctgattgcacgtgagcgacaaagcaatggcatctccgaatctgcacttatatttctccgaagagaaacgaagtgcgggctacgcagcatccggacgcttcaaacccgcttcatacattcgggcgaaccgtccagtcactatctgatcatatttttctattcagacgatccttaaatgttgggattaatcgacacccctcatatccagtctaaacatggggcggatacggggcttccgggagcactcgggcatgcgctctacgtcggaccaggcccacgctggtccacccgatcccacataaattcctctcatccgctcgccggtccgaaccctagccacttcactcctctcccctccactcccctccgccacccaacctcgtctccggttccttctggccatttccggcatggcgggcagcggatccaagtccttcacctcccgatctttcgacctcgaactcatcccacgcggccccgaggaggagatggccgtccggcttgcgctccgccgcgcccgggaggaggcccatgcatggctgcgctcgaactccattcatcgggaatccattacttccgtccaaatggcgcatggatccggcgctaggtcagccgtagctgcctcaccggaggccgtgcggtccgtgtggcgtccgaacacgctagcggacgcgcatcccctccgttggcgcactgagcatgaggacacaccatgcgcctcgtccgacgaggccatggcacgacgtgcccgccgtgcaaggcaccgggcacgggaggcggcggcgaacctcgcggcagtggacgtcggcgagacggagtcgcattctccagtgccccgtatggcgcatcagttcgggcaacgcaaccacatcgtggtggacatcggcggctcgtccccggacagatccgtcatcgatatgatgtctaccggcacccaacgggttccgggctccgacgatgaagagtagggcatgggagacggcggcgccttgagtcccgtgagctggtttgtgttccatgccctactctaccttgccggcgaccgggcgaacactctgggaagcgcagctggccgccggacatggccacggcgattggacgagctccagttaaagatcgctagattgcatgtaataatatggatttaaattttttaatttaaggcatccagatatggaatgtaatttttgagatgtaatcggtcactatccgcggatacacccagacgcatccacggacgtttgaagcatcggatttgccaaatccgacttagatgctctaatgctatgcttctattacggtcgacgccgtactgcaccgaaaccaattatttgatcacggcattagcttcagctagatgacaggctgatgagagattacgtaagaatacagattattgctaggcccgccaggcgagatacggtctcgtcaggccaagctaccgcacgcacacccccacttatgaaatgaacatatatttaggtaatacaacgaccaagaagacggccccctggattaatcgagtctggaagcaacaggtggggcgtctaaggaccagccccggatcattttgtaaagagacttgccggtaacgagactgaaccaggtatgatgataccgtcgatcaaatcttggaaaagtaacataccgatgacaaagggaacaacgtatgttgttatgcggtttgaccgataaaaatctcgtagaatatgtaggaatcaatatgagtatccaggtttcgctattggttattgaccgaagatgtgtctcggtcatgtctacatagttctcgaacccataggatacgcacgcttaacgttcaatgatgatttgtattatgagttatgtgatttgatgtaccgaagtttgtttggagtcccggatgagataacggacataacgaggagtcttgaaatggtcgagacataaaaattgatatattggaaggctatattcggacatcggaatggtttcagagaagtttgggtatttttcggagtaacgaaaggttaccggaacctcccggggagttgatgggccttagtgggccttagtgggaaggagaggcagcagccaagaggtggcgccccccaagcccagtccgagttggacaaggggttgggggcggggccccctctcccttcatcccccttctcctttaggtggaaacctactaggacatggagtcctagcaggattccccttctcccggcacgcctagcttggccggccggcctccccctccctcctttatatacgggggtagggggcatccaagaacacacaagttgatcttttagctgtgtgcggtgcccccctccacaaatacacacctcggtcatatcgtcgtagtggttaggcgaagccctgcgctggtaacttcatcatcaccgtcgccacgacatcgtgctgacggaactctccctcggcctcaactggatcaagagttcgagggacgtcattgagctgaacgtgtgctgatcacggaggtgccgtacgttcggtacttggatcggttggatcacgaagacgttcgactacatcaaccgcgttactaaacgcttccgctttcagtctacgagggtacgtagacacactctcccctctcgttgctatgcatctcctagatagatcttgcgtgatcgtaggattttttttgaaatattgcgttccccaacagtggcatccgagccaggactatgcgtagatgttatatgcacgagtagagcacaaagagttgtgggtgataatagtcatactgcttaccaacaacgtcttactttgattcgacggtattgttggatgaagcggcccggaccgacattacatgaccgcgttcatgagactggttctaccgacatgcttcgcacacatgtggctgacgggtgtgtgtttctccaactatagttgaatcgagtttgactacggccgatccttgttgaaggttaaaacaacacacatgacgaaaaatcgttatggttttgatgcgtagttaagaacgattcttgctagaagcccttagcagccacgtaaaacttgcaacaataaagtagatgtcgtctaacttgtttttgcagggcttgttgtgatgtgatatggtcaagatgtgatgatatataaattgttgtatgagatgatcatgttttgtaaaagttatcagcaactggcaggagccttatggttgtcgctttattgtatgaaatacaatcgccatgtaattgctttactttatcactaagcggcaacgatgctacgatggagatcaaggtgtcaagccggtgatgacggtgatgatgacggtgctttggagatggagatcaaaggcacaagatgatgatggccatatcgtatcacttatttgattgcatgtgatgtttatcctttatgcatcttattttgcttagtacggcggtagcattataagatgacatctcactaaatttcaaggtataagtgttctccgtgagtatgcaccgttgcttcagttcgtcatgccgagacaccacgtgatgatcgggtgtgataagctctacgttcacatacaatgggtgcaagccaattttcgcacacgcagaatactcaggttaaacttgacgagcctagcatatgcagatatggcctcggaacactaagtccgaaaggtcgagcatgaatcatatagtagatatgatcaacatactgatgttcaccattgaaaactactccat

>TaCIPK17_1AS_3274044

caataaaaggaatcgattgatttgcataatttaaggaagttagattaaaatgtattatttgtttcctgaaaatctgttatttgtttcctaagacaaattaaaccaataaaagaaatcgattaatttgcataatttaaggaagttagattaaaatgtattatttgtttcctgaaaatctgttatttgttttctaagacaaattgaaccaataaaaggaatcggttgatttgcataatttaaaaaagttagatccgtgatttgttatacgttaggaaagtcctggttgtaacaaagagtggagagaaaaataaaccgatggaccggggtgggagggggtggtgggaggagagacgaagaaaaccagccaaaataaaccacggaccagggtgggagggggtggtgggaggagagacgaaaataaaccgcgcagacgattcaccaactcgtccattaaaagtagagattttcgacagctttgggatcactcgcaccaccgaagcttccttttttacgtgaaaaggtttgatctatacatcttcaattaggttagtacaagcactgaaccgagcatcggaaataataaaaattacatccatatctgtaaaccatccaacgacgactacaaacactgaactgagtcaaagctgtaccgcagtcatcgccacttcctcgacagagctggacaaaacttgttgtagtagacagttagaaagtcatcgtgctaaagccccataggaccagcataccagaatagcaaccgtcgccgataaagagtaacgtagattgtaaggacgcaacgcgtagatgaacgatgaccagatccgagcagatccaccgaagacatacatccacatgcgcatcgacgatgctaatgctagacgcacgatcgaaacttctttctcacaaggatgaacatgggagtgctcgcaacgccgactgtttttctttcccctctcgcaaggcgactagggaaagccttccttcccttcttcacctgcgagcccgtggattcgcctctctttcgcctgccgctccggcgactggtgatcatgggaaggggatttctggtgcctcagatccggttagtagataggtaggattttagtcctcgcagaggcgacgtttggacgaatggtggcgcttcttctttgagtcagtcttccaggcttcgatcctccccaagttcgttcgttgggactggttagacggagctccaacgtagattccggccaactcctcagggcggcgaggttagggtttcttgtcgtgcgtaggcaacggcgatatttggtgtcaagttcttcagatctattcaagaattcaacggtgacgactgcggctccagagtgctggtccttaggggcacgtgcatgaagactttccagctgtcatcgacaagggcaagccggctctggtatgggagcggcgacaacggcccgtcggcggctcgttctggccttttggtgaatttttataatagctctaattttctcaagaaaaaaaaagaacatgggagtgctctcatgcgacggtcaaaaccaggcagccacaccgtttttttttactttttatatcttccttctccgctcgccacaccttcacaatgaataacgttattttattctgttggattggcttcaggattaaggtaatccaaacaccgagcaaaccccacaccacacacaaccacacgcaagttctcagaggattcagagctataaatactcccccctccacattccaattcgcctcgtcagcacgcgaagcacgccaccgacccaaatcaattcaccacgaacgacggaacgagtcgctctccgggcagcagatggtggcgacgggggacgcggaagaggcggcggcgggggccgcgcgcgggcggcgctgctgggcgcctacgagctcggccgcacgctcggggaaggcagcttcggcaaggtgaagcacgcgcggcaccgtgccacgggggaccacttcgcggtgaagatcctggaccgcggcagggtgctctccctccgcggcgccgacgaccaggtccgcagggagatcgccacgctcaccatgctcgcgcacccaaacgtcgtccgcctccacgaggtaattagtactattcactagtagtaatcgcgatctcggctcctgttcttctctcggtgctgcgctgctgcctctgctagatgcgtgtatagaatccatagattgagtacccttggtggagaaactgagaaaatattttaaaattttaaataccttgtcgagcttgagagcaagggtgcttgaactacgggcacgccacggttggaatgacgctacaattatcaaccagagattccttaaggaaagggatagttcgtggtggagattgcaagcatcatcatcatcatcatcatgtttatccacatatgtttttcctacattcatagcaacacaaacctataatttggggattctactactttgtatagtattaagagcaactccaacggccgacccaaacggacacggacggcgtttttgtccgctttttgtccgtttggatcggccgcccgcccgccgtccgctcttttttagttttgggtcggcagtgcacccaacgggccgatccattttcatgaccgcgcgtgtttaacatcgtaccgtcgtcctggttttggcgctccagcgcgcgggaaaggttcgcgcggggaaagcggtctagcgcgcgctgattttggcgctccagcgtgcgggaaaggttcgcgcgcgcgccgcggccggcgctcgctataaagaaggcgctctctccacactttgtcggtcgcccactctccctgcctctgcgccaccatgccgatcggccgcctgggcgcttcggacttttgcggagtccgcgagcgccgctccggtgccttcttcgccgagatctgatttcgcgagaaacgtctcgtcctcggcaccttcgacaccgcagaggaggcggcccgcgcgcacgacgcggcggcgtggcgcctcctgaggcctcgtcgggatatga

>TaCIPK17_1AS_3313233

ttttaaatgaatgtctaccttctgataaagaacaaccatatttcggtcatcaaacggaagctggcctatgagcattatgtaaagaattactccacaagaccagatatccgacagtgatccgtcataacctctgttctgcagaacctggatatatttggcacgagaattagcaaatataccgtaaaacagataactcgtcccaaggtggctagtcttttacctcaggtgcaatatagttggggctaccacaggttgtatgcagtaatccatccttctgaaaagttgcagactttaaattcaacgaaatgaaaatatcctcgcaacagaaaaatcaacgaaaatataatgatcaatacaaggaaatttttctgccataccccgagatgttgaggtaaagcacatagaccaaaatcagagatcttgatgttgccttttcggtcaatcagaacgttttcaggctgtaacatgtagcagtgggataagtcaggagaacacagagcaatcaaccataattccatcatacatgagaaataataatgttagataattgtttgccgcagattcttcaaaagaaaagaaaagaaaacttcagtcgaaagtaccttaaggtctctgtggtagacaccctttccatggcaatagctcacgccatcaattagctgctgaaaaagtcttcttccttctcgttcggatagttttcccttcattgccttttatttgcaggaaaaaaatcaactcaaagtgttagaactgaccaatgtacctggcagagaattttataatgcaaagcaagctttcagatagttgaaagtaaaagttataggggcttacaatcctgtcaaacagttctcctccattgacaaactcaagcaccatgtagatctttgttttgctagcagcaacctgaatcaagagttaaaagaattacaggattaatattacgacactgatattatttaaacgggccacgtcgatgaattcatatgctcatgtgcaccacaagagacccaaagaaattctgtctctcagaaaataaatagtaagatcatggcatagtcataaagatagggacggggcagcagctggttggcattattggatatacttgacggctacacatgcgatccaaatcaagcattaatcctaaaatagagaagtgaatggcctcgagctgaatctgcctccaactccacgtgctgctacaggtctagtggtctacgcgcaacgcgctacgtatagacaaatgctaatatccaatgcaatccctacaggctacaattattgataagtgctatgaatgtacgaaaacatatatagacaaatacattatgatggtgcttgcaatcccgagtacaagctgtggcgtgctgtggcgtgcccggatgtactgatacgttatcgccgtcccgagtacaagcctcccgcaaccgtcgacttgacagaacattctccttatactaccccatagaagaccaacgggctctttcccctccggcgtcaaatttttaaataattgcagcacgattcttcccggtcatcaagctggtcaatgttttgtaacccagttcgacatcctccatgtgtaacgcggaatgaagttgtatatatagactattaatatacaaagtagaatcccgaaattataggtttgtgttgctatgaatgtaggaaaacatatgtagataaataaacattatgatgatgatgcttgtaatctccaccacgaactatccctttccttaaggaatctctggttgataattgtagcgtcattccaaccgtggcgtgcccgtagttcaagcacccttgctctcaagctcgacaaggtatttaaaattttaaaatattttctcagtttctccaccaagggtactcaatctatggattctatacacgcatctagcagaggcagcagcgcagcaccgagagaagaacaggagccgagatcgcgattactactagtgaatagtactaattacctcgtggaggcggacgacgtttgggtgcgcgagcatggtgagcgtggcgatctccctgcggacctggtcgtcggcgccgcggagggagagcaccctgccgcggtccaggatcttcacggcgaagtggccccccgtggcgcggtgccgcgcgtgcttcaccttgccgaagctgccttccccgagcgtgcggcccagctcgtacccgcccagcagcgccgcccgcgcgcggcaccccgccgccgcctcttccgcgtcccccgtcgccaccatctgctgcccgctgctgcctggagagcgactcgttccgtcgttcgtggtgaattgggatttgggccggtgaattggcgtatttcgcgtgctgacgaggcgaattggaatgtggagggggcgtatttatagccctgaatcctctgagaagttgctgtgtgcgtgcgtgtggttgtgtgtgatgtggggttagctcgctgtttggattaccttaatcctgaagccaatccaacagaataaattaacgttattcattgtgaaggtttggcgagcggagaaggaagataaaaaggtaaaaagaaaaaacaggtgtggctgtctggttttgaccgtcgcatgagatgagagcactctcatattcatccttgtgaaaaagaagcttcagttgcacaggtgcgaatggtccattgctaattggacaaaactgccgaaaatgacaataacgcttctgtggtgtggctatattcaagctttttttttgcaggtagctatatttaggcttactggggcggtgttaatatgcttaccgtccggtgaggagaataaatcattttgtcgccacgattcatggtgtatttggattttagtaaatcaaaatttatattttattgatccaacatgcttcaatgcatctaattttgtcatttggattttagaattttaaatttgtaattttattgtgccttttgtttttctttggctactccatctttctagagggacgccaggttatgaaatatcggtcataaattgaatcttaaacatgtttgcacattttgggcaacaaacgtctacattgactcggtcctaccatccatcattacggcttgtttggttggaagagagtttgtgaattttattccttcggatattttccccagggccggccctgagggggtggcaggggggcggccgcccagggcccccgaaatctaggggccccctccagggttcgcaaggagcccatggcccagaccataaggaggcagcgacctagacgcacgtagtccaggagtattcatgttttccttctttccatcacgagttcgcagcttcgcgacgatactattccccgagggccccgatcgtgagacgcgccgccgatgtagatcgctctccgccatgcggccggctggtcttcctccaggtccccgggcccgttgtgttatcccgcgttcgcgagatgcctctcattgcaagacgaagccaccccacttgtaaggtcatccctaacctcctttttctagttggtcacgttcatatggtgcagccgcagtcagatcttagaaggtctcgcatatgatgcagtacaaaattttgatttcgtgtgcccgttgctctatttcttacttcaatctagactttgtcaacacaacaccaatcgccttatctattgaaaaaattgcctactatgtgttcgattgtcggtctttgcattctctctccaaatcatcatgactgcaaatttaacttaaaaattggaagaaggtctcaactattcttacgtgcataatttcaatccaagcatggttatataacaaagccactcagactcccatagccatcgggtaggtaaatgatcttttattgttgcaaatgaagctttaaaactttataaacctttcatcttgatgtttgccctctatca

>TaCIPK17_1AS_449966

gaaagaaattactgaaaatatttgtatacctcttcctcaaaaaaccctgaaagatcgagggaagatgccattccgatcagctgaaaagcattcatctgatgagtactcttgtctccgggtgcttcactaatttgcttgagaatagaaaatggaaacaaataatcaccgatcagcgacgtcaaatacgcaacacacgatgcctatattaggacataaattggtcttcagcattatacagtttgtgtcatcttgccatacctgtttgacaggcagaattgcaccaagccgtacatcttcatcatcgtcatcatatggaccaacaggagtatagtctttctgaaaccattcgtgtagtttgatctctgccatattgatcctcttcatcggatttggttcaagaatcctcttaagaaggttttgtgcaccaggcgaaagccactccgggaccttagtgtcacccttgaaaatctacacgcaaccagataacagtagtattattggaatggaatcacagaactatcagaacaaagcaccagatatcacttaggtttttagttgttttaaatgaatgtctaccttctgataaagaacaaccatatttcggtcatcaaacggaagctggcctatg

>TaCIPK17_1AS_461783

tgcaaaaataaatttcaagaaagcagcatcattaagtcattcactcaataaccaccagcactcaagatgtgtagagtgaagttatggcagtggcaaatctagttgctgttagcactcttgcctacctgtctgtacactgcagggtcaccgtgggacttcctaagctcaacaacatgcagagaggggccgagctcaaacacctgggatagaaaagcagcaccctagatgttagcagctgatctccatcggttatcattcccatgcatttcactgaagaaaagtgcagctcaccgaattatacctcgacacagactaagaatggcaaagggttgttcgctccattgcaattgcccgttattttgagctgaaatataacacggcacatcattttcagttccttcaaaagttgtactcaataatgtgatgacgaaaaatctctacaatgtgatgatgagaagcttgtgctcaccttgctatgcactctttgaacgtggagccccgattgcgttgccgttacttcaatcttgttgaacaaatccttcggtggaagtgctgatgtgaacctgatctttctctgggacactccctgcaataagtccaactggatgagatgagttgttctatgaggccgcagtggcagcattcagtgatcatcttgatctaaaacatcttgagaaactatgcatatgatttttcttcccaattttgcagattaaatcattctgattatagcaagggaggcacaagaaagaaattactgaaaatatttttatacctcttcctcaaaaaacccagaaagatcgagggaagatgccattccgatcagctgaaaagcgttcatctgacgagtactcttgtccccaggtgcttcactaatttgcttctggatagaaaaaggaaacaaaaaatcacctatcagcggctacaaatacgcaacacagatgcctatattaggtcataaattggtcttcagcattaaacagtttgtgtcatcttgccatacctgtttcacaggcagaattgcaccaagccgtacatcttcatcatcgtcatcatatggaccaacatgaatatagtctttctgaaaccattcgtgttgtttgatctctgccatgttgattctcttcatcggatttggttcaagaatcctcttaataatattttgtgcaccaggcgaaagccactccgggatcttagcgtcacccttgaaaatctacacgcaaccacataacagtagtattattggaatggaatcatagaactatcagaacaaagcaccagatatcacttaagtttttagttttttttaaatgaatgtctaccttctgataaagaacaaccatatttcggtcatcaaacggaagctggcctatg

>TaCIPK17_1BS_1265003
[truncated: 881,266 more chars]
