# Supplementary material for: Identification and comprehensive analyses of the CBL and CIPK gene families in wheat (Triticum aestivum L.)
Source: BMC Plant Biol. 2015 Nov 4;15:269. doi: 10.1186/s12870-015-0657-4 (PMC4634908; doi:10.1186/s12870-015-0657-4)
Supplement: Additional file 4: — Exon-intron analysis of TaCBLs and TaCIPKs in wheat. (PDF 1435 kb) [file 12870_2015_657_MOESM4_ESM.pdf]

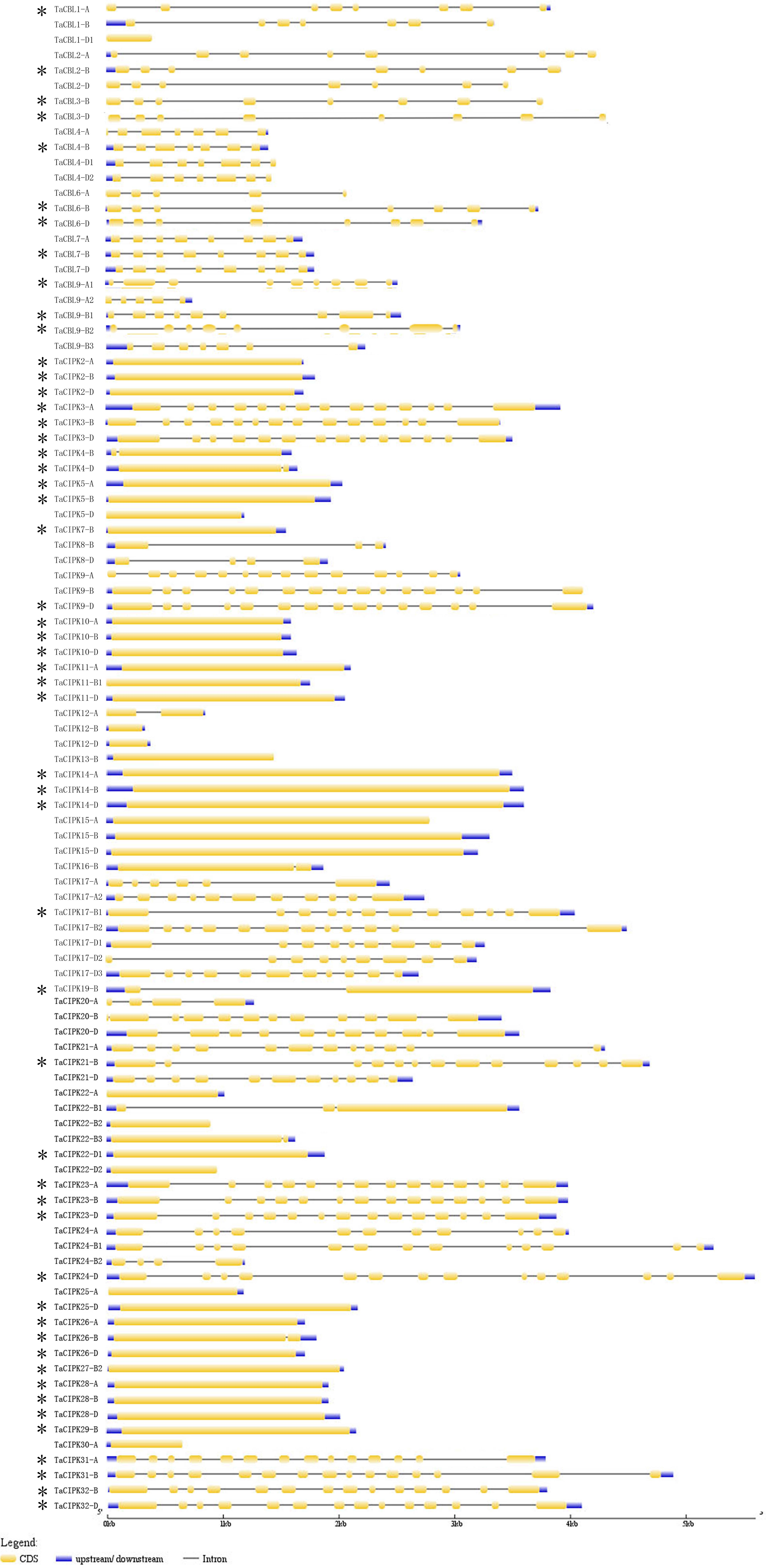
* TaCBL1-A
TaCBL1-B
TaCBL1-D1
TaCBL2-A
* TaCBL2-B
TaCBL2-D
* TaCBL3-B
* TaCBL3-D
TaCBL4-A
* TaCBL4-B
TaCBL4-D1
TaCBL4-D2
TaCBL6-A
* TaCBL6-B
* TaCBL6-D
TaCBL7-A
* TaCBL7-B
TaCBL7-D
* TaCBL9-A1
TaCBL9-A2
* TaCBL9-B1
* TaCBL9-B2
TaCBL9-B3
* TaCIPK2-A
* TaCIPK2-B
* TaCIPK2-D
* TaCIPK3-A
* TaCIPK3-B
* TaCIPK3-D
* TaCIPK4-B
* TaCIPK4-D
* TaCIPK5-A
* TaCIPK5-B
TaCIPK5-D
* TaCIPK7-B
TaCIPK8-B
TaCIPK8-D
TaCIPK9-A
TaCIPK9-B
* TaCIPK9-D
* TaCIPK10-A
* TaCIPK10-B
* TaCIPK10-D
* TaCIPK11-A
* TaCIPK11-B1
* TaCIPK11-D
TaCIPK12-A
TaCIPK12-B
TaCIPK12-D
TaCIPK13-B
* TaCIPK14-A
* TaCIPK14-B
* TaCIPK14-D
TaCIPK15-A
TaCIPK15-B
TaCIPK15-D
TaCIPK16-B
TaCIPK17-A
TaCIPK17-A2
* TaCIPK17-B1
TaCIPK17-B2
TaCIPK17-D1
TaCIPK17-D2
TaCIPK17-D3
* TaCIPK19-B
TaCIPK20-A
TaCIPK20-B
TaCIPK20-D
TaCIPK21-A
* TaCIPK21-B
TaCIPK21-D
TaCIPK22-A
TaCIPK22-B1
TaCIPK22-B2
TaCIPK22-B3
* TaCIPK22-D1
TaCIPK22-D2
* TaCIPK23-A
* TaCIPK23-B
* TaCIPK23-D
TaCIPK24-A
TaCIPK24-B1
TaCIPK24-B2
* TaCIPK24-D
TaCIPK25-A
* TaCIPK25-D
* TaCIPK26-A
* TaCIPK26-B
* TaCIPK26-D
* TaCIPK27-B2
* TaCIPK28-A
* TaCIPK28-B
* TaCIPK28-D
* TaCIPK29-B
TaCIPK30-A
* TaCIPK31-A
* TaCIPK31-B
* TaCIPK32-B
* TaCIPK32-D
5'
3'
0kb
1kb
2kb
3kb
4kb
5kb
Legend:
CDS
upstream/ downstream
Intron

Additional files 4. Exon-intron analysis of *TaCBLs* and *TaCIPKs* in wheat. The boxes and solid lines represent “exon” and intron, respectively. The “upstream/downstream” indicts the flank sequence of “intron/exon” structure in genome sequence. The complete structures covering the full length ORF of genes were marked with "*".
