# Supplementary material for: Identification and comprehensive analyses of the CBL and CIPK gene families in wheat (Triticum aestivum L.)
Source: BMC Plant Biol. 2015 Nov 4;15:269. doi: 10.1186/s12870-015-0657-4 (PMC4634908; doi:10.1186/s12870-015-0657-4)
Supplement: Additional file 5: — Analysis of EF-hand motifs in calcium binding proteins of representative species. (PDF 147 kb) [file 12870_2015_657_MOESM5_ESM.pdf]

```
                          (1)  1        10        20        30             46
AtCAM1 NP_001154755       (1)  ----------------------------------------------
OsCaM LOC_Os05g41210      (1)  ----------------------------------------------
AtCAM2 NP_001189724       (1)  ----------------------------------------------
OsCaM3 LOC_Os01g17190     (1)  ----------------------------------------------
CeCaM NP_503386           (1)  ----------------------------------------------
DrCaM BC164893            (1)  ----------------------------------------------
HsCML3 NP_005176          (1)  ----------------------------------------------
ScCaM NP_009667           (1)  ----------------------------------------------
MmCam4 NP_064420          (1)  ----------------------------------------------
AtCBL4 AF192886           (1)  ----------------------------------------------
OsCBL4 ABA54179           (1)  ----------------------------------------------
TaCBL4                    (1)  ----------------------------------------------
OsCBL7 ABA54182           (1)  ----------------------------------------------
TaCBL7                    (1)  ----------------------------------------------
OsCBL9 ABA54184           (1)  MESGYGFRFSDDDVESASSLTVGERLCAAFLPFVAIAEAVFFALTD
TaCBL9                    (1)  MASRFNSRISSGDLRSGSSLTVGERLCAVVLPFVAIAEFVFFALTD
AtCBL1 AAC26008           (1)  ----------------------------------------------
AtCBL9 AF411958_1         (1)  ----------------------------------------------
OsCBL1 ABA54176           (1)  ----------------------------------------------
TaCBL1                    (1)  ----------------------------------------------
AtCBL7 AF290434_1         (1)  ----------------------------------------------
AtCBL6 AF192884_1         (1)  ------------------------------------------MMMQ
AtCBL2 AAC26009           (1)  -------------------------------------------MSQ
AtCBL3 AAC26010           (1)  -------------------------------------------MSQ
OsCBL2 ABA54177           (1)  -------------------------------------------MVQ
OsCBL3 ABA99199           (1)  -------------------------------------------MVQ
TaCBL2                    (1)  -------------------------------------------MVQ
TaCBL3                    (1)  -------------------------------------------MLQ
OsCBL6 ABA54181           (1)  -------------------------------------------MVD
TaCBL6                    (1)  -------------------------------------------MVD
Consensus                 (1)

                         (47)  47           60        70        80         92
AtCAM1 NP_001154755       (1)  ----------------------------------------------
OsCaM LOC_Os05g41210      (1)  ----------------------------------------------
AtCAM2 NP_001189724       (1)  ----------------------------------------------
OsCaM3 LOC_Os01g17190     (1)  ----------------------------------------------
CeCaM NP_503386           (1)  ----------------------------------------------
DrCaM BC164893            (1)  ----------------------------------------------
HsCML3 NP_005176          (1)  ----------------------------------------------
ScCaM NP_009667           (1)  ----------------------------------------------
MmCam4 NP_064420          (1)  ----------------------------------------------
AtCBL4 AF192886           (1)  -------MGCSVSK-------KKKKNAMR-PPG-------------
OsCBL4 ABA54179           (1)  -------MGCASS-----------KQFKR-PPG-------------
TaCBL4                    (1)  -------MGCVLSS-------P--RRSRR-TPG-------------
OsCBL7 ABA54182           (1)  -------MGCISS-----------KQFKR-AAE-------------
TaCBL7                    (1)  -------MGCVSS-----------KQFRR-APP-------------
OsCBL9 ABA54184          (47)  CLADLLPPSAAAS----RHRRSAASSYLAAVAR--KWNHQQRGRVG
TaCBL9                   (47)  CLAGICSPPSASSSSHPRRDPSAPSFFLTAKKGSHHHHHLLRRRVA
AtCBL1 AAC26008           (1)  -------MGCFHSK-------AAKEF-----RG-------------
AtCBL9 AF411958_1         (1)  -------MGCFHST-------AAREF-----PD-------------
OsCBL1 ABA54176           (1)  -------MGCFQST-------ARRP--R---PG-------------
TaCBL1                    (1)  -------MGCIQST-------PKRRQHP---AG-------------
AtCBL7 AF290434_1         (1)  -MDSTRNSASSNST-------GCFTDQK------------------
AtCBL6 AF192884_1         (5)  CLDGLKHLALILLT-------CCDADPP---KV-------------
AtCBL2 AAC26009           (4)  CVDGIKHLCTSVLG-------CFDLDLYKQSGG-------------
AtCBL3 AAC26010           (4)  CIDGFKHVCSSFFR-------CFDIDIYKQSGG-------------
OsCBL2 ABA54177           (4)  CLDGVRQLLAVVFK-------CCDLELK-QPRG-------------
OsCBL3 ABA99199           (4)  CLDGVRQLLAVVFK-------CCDLELK-QPRG-------------
TaCBL2                    (4)  CLDGVKHLLAVLFK-------CCDLELK-QPRG-------------
TaCBL3                    (4)  CLEGVRHLGGVLLK-------CCDIDLK-QPKG-------------
OsCBL6 ABA54181           (4)  SSEGLRRLAALLFK-------CCSLDSSNRPNG-------------
TaCBL6                    (4)  FPEGLRRLAALMLK-------CCDLDIPNRPKG-------------
Consensus                (47)         M                        G
```

```
                          (93) 93     100       110       120            138
      AtCAM1 NP_001154755   (1) -------------MADQLTDEQISEFKEAFSLFDKDGDVFVLSDLG
     OsCaM LOC_Os05g41210   (1) -------------MADQLTDEQIAEFKEAFSLFDKDG---------
      AtCAM2 NP_001189724   (1) -------------MADQLTDDQISEFKEAFSLFDKDGDG-------
    OsCaM3 LOC_Os01g17190   (1) -------------MADQLTDDQIAEFKEAFSLFDKDG---------
          CeCaM NP_503386   (1) -------------MADQLTEEQIAEFKEAFSLFDKDG---------
           DrCaM BC164893   (1) -------------MADQLTEEQIAEFKEAFSLFDKDG---------
        HsCML3 NP_005176   (1) -------------MADQLTEEQVTEFKEAFSLFDKDG---------
          ScCaM NP_009667   (1) -------------MSSNLTEEQIAEFKEAFALFDKDN---------
        MmCam4 NP_064420   (1) -------------MSHGFTKEEVAEFQAAFNRFDKNK---------
        AtCBL4 AF192886  (19) --------YEDPELLASVTPFTVEEVEALYELFKKLSSSII-----
       OsCBL4 ABA54179  (15) --------YEEPAVLAAQTTFTVNEVEALRELYNKMSYSII-----
                 TaCBL4  (17) --------YEEPTVLASQTSFTVNEVEALYELYKKLSYSIF-----
       OsCBL7 ABA54182  (15) --------HEDPAILAKETTFSVSEVEALYELFKKISHSIF-----
                 TaCBL7  (15) --------HEDAALLAKETTFSLNEVEALYELFKKISYSIF-----
       OsCBL9 ABA54184  (87) IGCTSLTLRQLARLADESRCFSVNEVEALFELYEKTSCSII-----
                 TaCBL9  (93) PGCSSLSFRDLARLADESRCFSVNEVEALFELYKKISCSII-----
        AtCBL1 AAC26008  (15) --------HEDPVKLASETAFSVSEVEALFELFKSISSSVV-----
      AtCBL9 AF411958_1  (15) --------HENPVKLASETAFSVSEVEALYELFKSISSSVV-----
       OsCBL1 ABA54176  (15) --------YEDPVGLASETAFSVSEVEALFELFKSISGSVI-----
                 TaCBL1  (17) --------YEDPVHLASQTAFSVSEVEALFELFKSISGSVI-----
      AtCBL7 AF290434_1  (21) --------KRK---ALYEVFKKLSGVDCQRNEGNVVEGVTC-----
      AtCBL6 AF192884_1  (28) --------RQNPKDVARGTVFTVNEIEALYELFKSIS---------
        AtCBL2 AAC26009  (30) --------LGDPELLARDTVFSVSEIEALYELFKKISSAVI-----
        AtCBL3 AAC26010  (30) --------LGDPELLARETVFSVSEIEALYELFKKISSAVI-----
       OsCBL2 ABA54177  (29) --------LEDPQVLARETVFSVSEVEALYELFKKISSAVI-----
       OsCBL3 ABA99199  (29) --------LEDPQVLARETVFSVSEVEALYELFKKISSAVI-----
                 TaCBL2  (29) --------LEDPQVLARETVFSVSEVEALYELFKKISSAVI-----
                 TaCBL3  (29) --------LEDPEVLARETVFSVSEVEALYELFKKISSAVI-----
       OsCBL6 ABA54181  (30) --------LQDPERLARETVFNVNEIEALYELFKKISSAVV-----
                 TaCBL6  (30) --------LEDPERLARETVFSVNEIEALYELFKKISSAVV-----
              Consensus  (93)          EDP MLA  T FSVSEVEALFELFKKIS SVI
                                                  EF hand 1
```

```
                         (139) 139        150       160       170         184
      AtCAM1 NP_001154755  (34) FDFKRLSNCLETTPELSHGCITTKELGTVMRSLGQNPTEAELQDMI
     OsCaM LOC_Os05g41210  (25) -----------------DGCITTKELGTVMRSLGQNPTEAELQDMI
      AtCAM2 NP_001189724  (27) -------MLHPPFPSIIVGCITTKELGTVMRSLGQNPTEAELQDMI
    OsCaM3 LOC_Os01g17190  (25) -----------------DGCITTKELGTVMRSLGQNPTEAELQDMI
          CeCaM NP_503386  (25) -----------------DGTITTKELGTVMRSLGQNPTEAELQDMI
           DrCaM BC164893  (25) -----------------DGTITTKELGTVMRSLGQNPTEAELQDMI
        HsCML3 NP_005176  (25) -----------------DGCITTRELGTVMRSLGQNPTEAELRDMM
          ScCaM NP_009667  (25) -----------------NGSISSSELATVMRSLGLSPSEAEVNDLM
        MmCam4 NP_064420  (25) -----------------DGHISVEELGDVMKQLGKNLPEKDLKALI
        AtCBL4 AF192886  (52) ----------------DDGLIHKEEFQLALFRNRNR-RNLFADRIF
       OsCBL4 ABA54179  (48) ----------------KDGLIHKEEFQLALFRNSRK-ANLFADRVF
                 TaCBL4  (50) ----------------KDGLIHKEEFRLALFRTSKG-ANLFADRVF
       OsCBL7 ABA54182  (48) ----------------KDGLIHKEEFQLALFRNSNK-KNLFADRIF
                 TaCBL7  (48) ----------------KDGLIHKEEFQLALFRNSNR-KNLFADRIF
       OsCBL9 ABA54184 (128) ----------------DDGLTHKEEPQPALFRTPSG-RNLFLDWVF
                 TaCBL9 (134) ----------------NDGLIHKEELQLALFKTPSG-QNLFLDRVF
        AtCBL1 AAC26008  (48) ----------------DDGLINKEEFQLALFKSRKR-ENIFANRIF
      AtCBL9 AF411958_1  (48) ----------------DDGLINKEEFQLALFKNRKK-ENLFANRIF
       OsCBL1 ABA54176  (48) ----------------DDGLINKEEFRLALFKNKRK-ENLFANRIF
                 TaCBL1  (50) ----------------DDGLINKEEFQLALFKNTRK-ENLFANRIF
      AtCBL7 AF290434_1  (51) ----------------YYGEMNKEQFHVAIFQTDKN-ESLFSERVF
      AtCBL6 AF192884_1  (57) ----------------KNGLIDKEQFQLVLFKMNTT-RSLFADRVF
        AtCBL2 AAC26009  (63) ----------------DDGLINKEEFQLALFKTNKK-ESLFADRVF
        AtCBL3 AAC26010  (63) ----------------DDGLINKEEFQLALFKTNKK-ESLFADRVF
       OsCBL2 ABA54177  (62) ----------------DDGLINKEEFQLALFKTSKK-ESLFADRVF
       OsCBL3 ABA99199  (62) ----------------DDGLINKEEFQLALFKTSKK-ESLFADRVF
                 TaCBL2  (62) ----------------DDGLINKEEFQLALFKTSKK-ESLFADRVF
                 TaCBL3  (62) ----------------DDGLINKEEFQLALFKTSKK-ESLFADRVF
       OsCBL6 ABA54181  (63) ----------------DDGLINKEEFQLALFKTNRK-DSMFADRVF
                 TaCBL6  (63) ----------------DDGVINKEEFQLALFKTNRK-DSMFADRVF
              Consensus (139)                  DGLI KEEFQLALFK  KK   LFADRVF
                                         EF hand 1                   EF hand 2
```

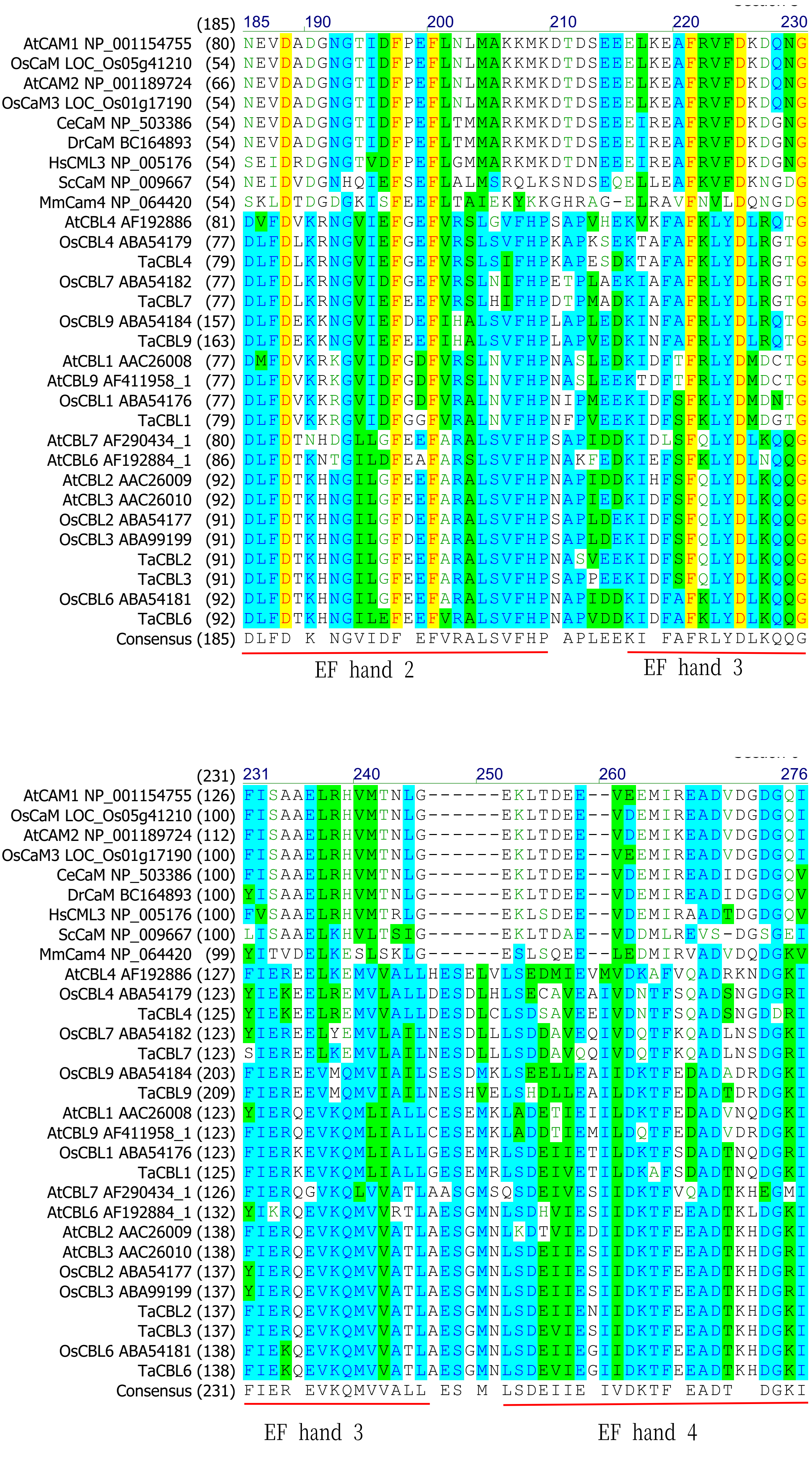
(185) 185 190 200 210 220 230
AtCAM1 NP_001154755 (80)
OsCaM LOC_Os05g41210 (54)
AtCAM2 NP_001189724 (66)
OsCaM3 LOC_Os01g17190 (54)
CeCaM NP_503386 (54)
DrCaM BC164893 (54)
HsCML3 NP_005176 (54)
ScCaM NP_009667 (54)
MmCam4 NP_064420 (54)
AtCBL4 AF192886 (81)
OsCBL4 ABA54179 (77)
TaCBL4 (79)
OsCBL7 ABA54182 (77)
TaCBL7 (77)
OsCBL9 ABA54184 (157)
TaCBL9 (163)
AtCBL1 AAC26008 (77)
AtCBL9 AF411958_1 (77)
OsCBL1 ABA54176 (77)
TaCBL1 (79)
AtCBL7 AF290434_1 (80)
AtCBL6 AF192884_1 (86)
AtCBL2 AAC26009 (92)
AtCBL3 AAC26010 (92)
OsCBL2 ABA54177 (91)
OsCBL3 ABA99199 (91)
TaCBL2 (91)
TaCBL3 (91)
OsCBL6 ABA54181 (92)
TaCBL6 (92)
Consensus (185) DLFD K NGVIDF EFVRALSVFHP APLEEKI FAFRLYDLKQQG
EF hand 2
EF hand 3
(231) 231 240 250 260 276
AtCAM1 NP_001154755 (126)
OsCaM LOC_Os05g41210 (100)
AtCAM2 NP_001189724 (112)
OsCaM3 LOC_Os01g17190 (100)
CeCaM NP_503386 (100)
DrCaM BC164893 (100)
HsCML3 NP_005176 (100)
ScCaM NP_009667 (100)
MmCam4 NP_064420 (99)
AtCBL4 AF192886 (127)
OsCBL4 ABA54179 (123)
TaCBL4 (125)
OsCBL7 ABA54182 (123)
TaCBL7 (123)
OsCBL9 ABA54184 (203)
TaCBL9 (209)
AtCBL1 AAC26008 (123)
AtCBL9 AF411958_1 (123)
OsCBL1 ABA54176 (123)
TaCBL1 (125)
AtCBL7 AF290434_1 (126)
AtCBL6 AF192884_1 (132)
AtCBL2 AAC26009 (138)
AtCBL3 AAC26010 (138)
OsCBL2 ABA54177 (137)
OsCBL3 ABA99199 (137)
TaCBL2 (137)
TaCBL3 (137)
OsCBL6 ABA54181 (138)
TaCBL6 (138)
Consensus (231) FIER EVKQMVVALL ES M LSDEIIE IVDKTF EADT DGKI
EF hand 3
EF hand 4

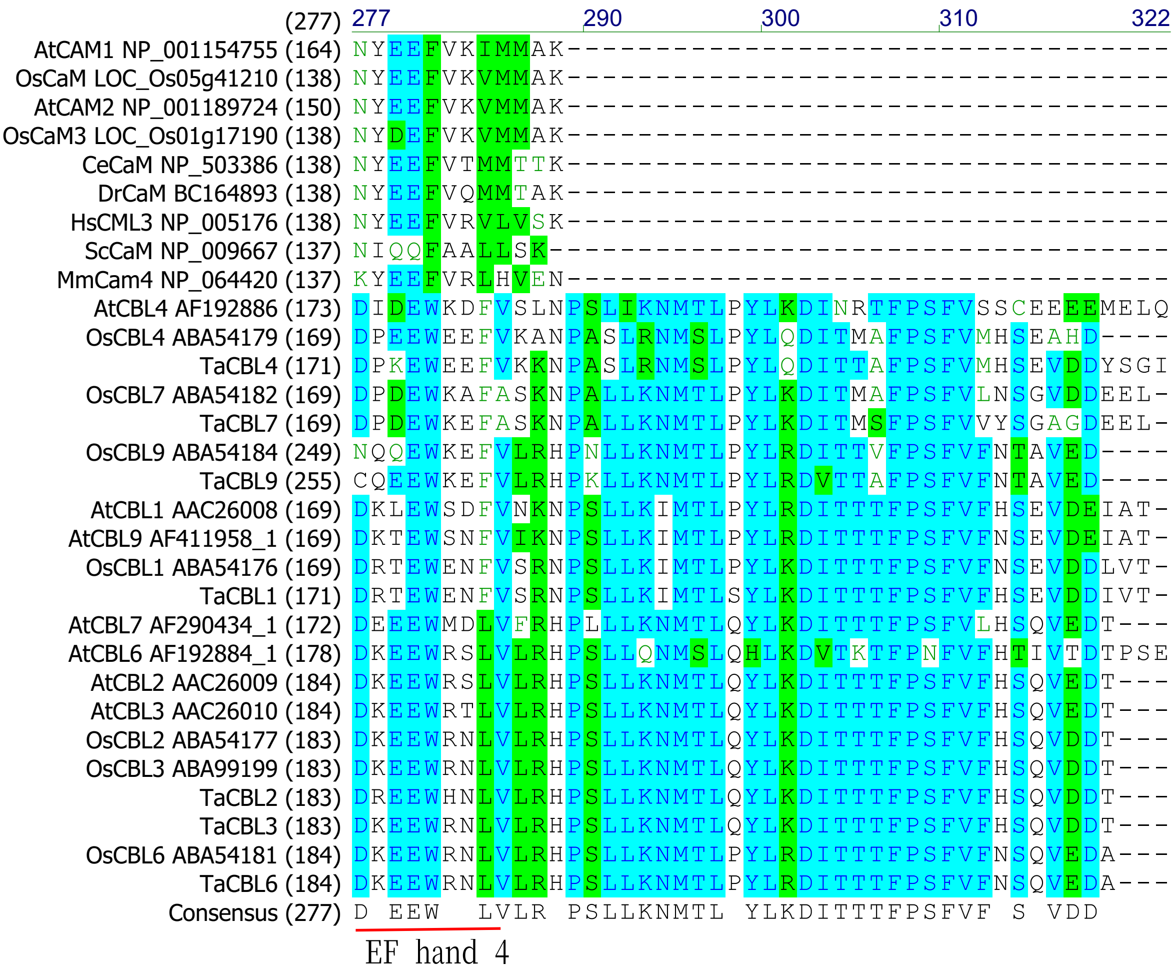
(277) 277 290 300 310 322
AtCAM1 NP_001154755 (164) NYEEFVKIMMAK----------------------------------
OsCaM LOC_Os05g41210 (138) NYEEFVKVMMAK----------------------------------
AtCAM2 NP_001189724 (150) NYEEFVKVMMAK----------------------------------
OsCaM3 LOC_Os01g17190 (138) NYDEFVKVMMAK----------------------------------
CeCaM NP_503386 (138) NYEEFVTMMTTK----------------------------------
DrCaM BC164893 (138) NYEEFVQMMTAK----------------------------------
HsCML3 NP_005176 (138) NYEEFVRVLVSK----------------------------------
ScCaM NP_009667 (137) NIQQFAALLSK-----------------------------------
MmCam4 NP_064420 (137) KYEEFVRLHVEN----------------------------------
AtCBL4 AF192886 (173) DIDEWKDFVSLNPSLIKNMTLPYLKDINRTFPSFVSSCEEEEMELQ
OsCBL4 ABA54179 (169) DPEEWEEFVKANPASLRNMSLPYLQDITMAFPSFVMHSEAHD----
TaCBL4 (171) DPKEWEEFVKKNPASLRNMSLPYLQDITTAFPSFVMHSEVDDYSGI
OsCBL7 ABA54182 (169) DPDEWKAFASKNPALLKNMTLPYLKDITMAFPSFVLNSGVDDEEL-
TaCBL7 (169) DPDEWKEFASKNPALLKNMTLPYLKDITMSFPSFVVYSGAGDEEL-
OsCBL9 ABA54184 (249) NQQEWKEFVLRHPNLLKNMTLPYLRDITTVFPSFVFNTAVED----
TaCBL9 (255) CQEEWKEFVLRHPKLLKNMTLPYLRDVTTAFPSFVFNTAVED----
AtCBL1 AAC26008 (169) DKLEWSDFVNKNPSLLKIMTLPYLRDITTTFPSFVFHSEVDEIAT-
AtCBL9 AF411958_1 (169) DKTEWSNFVIKNPSLLKIMTLPYLRDITTTFPSFVFNSEVDEIAT-
OsCBL1 ABA54176 (169) DRTEWENFVSRNPSLLKIMTLPYLKDITTTFPSFVFNSEVDDLVT-
TaCBL1 (171) DRTEWENFVSRNPSLLKIMTLSYLKDITTTFPSFVFHSEVDDIVT-
AtCBL7 AF290434_1 (172) DEEEWMDLVFRHPLLLKNMTLQYLKDITTTFPSFVLHSQVEDT---
AtCBL6 AF192884_1 (178) DKEEWRSLVLRHPSLLQNMSLQHLKDVTKTFPNFVFHTIVTDTPSE
AtCBL2 AAC26009 (184) DKEEWRSLVLRHPSLLKNMTLQYLKDITTTFPSFVFHSQVEDT---
AtCBL3 AAC26010 (184) DKEEWRTLVLRHPSLLKNMTLQYLKDITTTFPSFVFHSQVEDT---
OsCBL2 ABA54177 (183) DKEEWRNLVLRHPSLLKNMTLQYLKDITTTFPSFVFHSQVDDT---
OsCBL3 ABA99199 (183) DKEEWRNLVLRHPSLLKNMTLQYLKDITTTFPSFVFHSQVDDT---
TaCBL2 (183) DREEWHNLVLRHPSLLKNMTLQYLKDITTTFPSFVFHSQVDDT---
TaCBL3 (183) DKEEWRNLVLRHPSLLKNMTLQYLKDITTTFPSFVFHSQVDDT---
OsCBL6 ABA54181 (184) DKEEWRNLVLRHPSLLKNMTLPYLRDITTTFPSFVFNSQVEDA---
TaCBL6 (184) DKEEWRNLVLRHPSLLKNMTLPYLRDITTTFPSFVFNSQVEDA---
Consensus (277) D EEW LVLR PSLLKNMTL YLKDITTTFPSFVF S VDD
EF hand 4

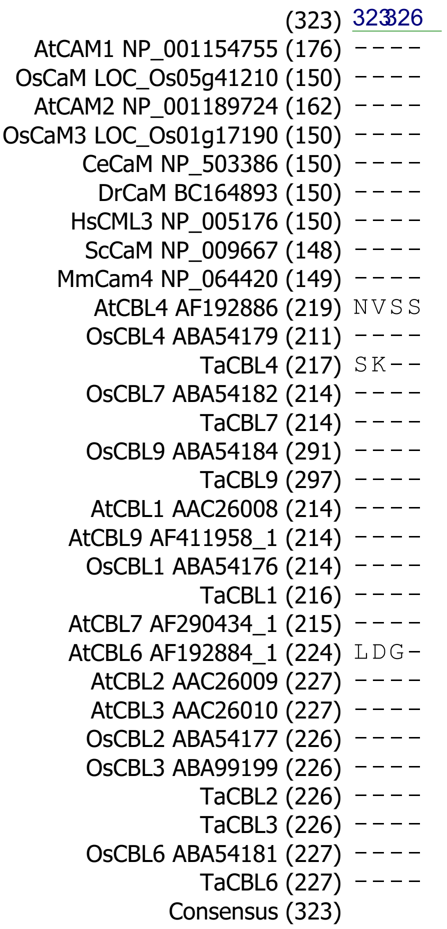
(323) 323 326
AtCAM1 NP_001154755 (176) ----
OsCaM LOC_Os05g41210 (150) ----
AtCAM2 NP_001189724 (162) ----
OsCaM3 LOC_Os01g17190 (150) ----
CeCaM NP_503386 (150) ----
DrCaM BC164893 (150) ----
HsCML3 NP_005176 (150) ----
ScCaM NP_009667 (148) ----
MmCam4 NP_064420 (149) ----
AtCBL4 AF192886 (219) NVSS
OsCBL4 ABA54179 (211) ----
TaCBL4 (217) SK--
OsCBL7 ABA54182 (214) ----
TaCBL7 (214) ----
OsCBL9 ABA54184 (291) ----
TaCBL9 (297) ----
AtCBL1 AAC26008 (214) ----
AtCBL9 AF411958_1 (214) ----
OsCBL1 ABA54176 (214) ----
TaCBL1 (216) ----
AtCBL7 AF290434_1 (215) ----
AtCBL6 AF192884_1 (224) LDG-
AtCBL2 AAC26009 (227) ----
AtCBL3 AAC26010 (227) ----
OsCBL2 ABA54177 (226) ----
OsCBL3 ABA99199 (226) ----
TaCBL2 (226) ----
TaCBL3 (226) ----
OsCBL6 ABA54181 (227) ----
TaCBL6 (227) ----
Consensus (323)

Additional file 5: Analysis of EF-hand motifs in calcium binding proteins of representative species. Multiple sequence alignment was performed using the ClustalX1.83. Identical amino acids are shaded in yellow, conservative amino acids are shaded in light blue and similar amino acids are shaded in green. The four EF-hand motifs are indicated in the figure.
