# Supplementary material for: Identification and comprehensive analyses of the CBL and CIPK gene families in wheat (Triticum aestivum L.)
Source: BMC Plant Biol. 2015 Nov 4;15:269. doi: 10.1186/s12870-015-0657-4 (PMC4634908; doi:10.1186/s12870-015-0657-4)
Supplement: Additional file 6: — Analysis of SnRK proteins of representative species. (PDF 420 kb) [file 12870_2015_657_MOESM6_ESM.pdf]

```
                           (1) 1       10        20        30             46
AtSnRK2.4 At1g10940        (1) ----------------------------------------------
AtSnRK2.9 AT2G23030        (1) ----------------------------------------------
HsSnRK NP_001094064        (1) -----------------------------------MAGFKRGYDGK
MmSnRK NP_001158044.1      (1) -----------------------------------MAGFKRGYDGK
OsSnRK 1a Os05g0530500     (1) ----------------------------------MEGAGRDG---N
OsSnRk1b Os08g0484600      (1) ----------------------------------MEGNARGGGHSE
ZmSnRK NP_001105673.1      (1) ----------------------------------MDGSSKGSGHSE
TaCIPK20                   (1) MYAVDERETHRASPFYRAVVHAVDGRADGQTATGSCARKSRCHGYF
TaCIPK17                   (1) ---------------------------MVATGDAEEAAAGCRARAA
TaCIPK21                   (1) --------------------------------------MAAAEGAM
AtSOS2 AAF62923.1          (1) ----------------------------------------MTKKMR
TaCIPK24                   (1) --------------------------------------MAGAARKK
TaCIPK8                    (1) -----------------------------------MVGGGGGGALR
TaCIPK23                   (1) --------------------------MVDSSAGGKMSAHGGGGGRT
TaCIPK3                    (1) -------------------------------MYKARRQAS-LKVRR
TaCIPK31                   (1) -------------------------------MYRAKRAALSPKVKR
TaCIPK32                   (1) --------------------------------------MSTTKVKR
TaCIPK9                    (1) ----------------------------------MAAAGGGPRRTT
TaCIPK22                   (1) ------------------------------MGPEDSPAAGESYSKV
TaCIPK29                   (1) -------------------MPSASSAVPSAVAPGDESQS-LAAPKV
TaCIPK4                    (1) ----------------------------------MNGRRKKSRGTP
TaCIPK7                    (1) --------------------------------MAVAKSKAGKHAAP
TaCIPK12                   (1) -----MAATPPSSRDPSPQPRRPAAAAGRPAASGTGTIGNGKRGGL
TaCIPK19                   (1) -----MSAIKPPPPDRPPQAARLPSPSSSSSAVAAAKRGATGSRGL
TaCIPK25                   (1) ----------------------------------------MGDRPK
TaCIPK13                   (1) --------------MARMAGKGSSGRALAGGASGASGREREGKKAL
TaCIPK10                   (1) --------------------------------------MVEKKGNI
TaCIPK2                    (1) --------------------------------------MGEQKGNI
TaCIPK11                   (1) --------------------------------------MMDERRTI
TaCIPK26                   (1) ---------------------------------------MEDRRTI
TaCIPK28                   (1) ----------------------------------------MEERSV
TaCIPK14                   (1) -----------------------------MGCGFMQLEVMANRGKI
TaCIPK15                   (1) ---------------------------------------MENSGKI
TaCIPK5                    (1) ---------------------------------------MERKSAI
TaCIPK27                   (1) -----------------------------------MEDAAEGKTNV
TaCIPK6                    (1) -----------------------------------MEDAAEGKTNV
TaCIPK30                   (1) -----------------------------------MAMEKNQDSKV
TaCIPK16                   (1) ----------------------------------------------
Consensus                  (1)
```

Catalytic domain of the Serine/Threonine Kinases

```
                          (47) 47          60        70        80           92
AtSnRK2.4 At1g10940        (1) -MDKYELVKDIGAGNFGVARLMKVKNSKELVAMKYIERG-------
AtSnRK2.9 AT2G23030        (1) -MEKYEMVKDLGFGNFGLARLMRNKQTNELVAVKFIDRG-------
HsSnRK NP_001094064       (12) IAGLYDLDKTLGRGHFAVVKLARHVFTGEKVAVKVIDKT---KLD-
MmSnRK NP_001158044.1     (12) IAGLYDLDKTLGRGHFAVVKLARHVFTGEKVAVKVIDKT---KLD-
OsSnRK 1a Os05g0530500    (10) PLGGYRIGKTLGIGSFGKVKIAEHILTGHKVAIKILNRR----KS-
OsSnRk1b Os08g0484600     (13) ALKNYNLGRTLGIGSFGKVKIAEHKLTGHRVAIKILNRRQ--MRN-
ZmSnRK NP_001105673.1     (13) ALRNYNLGRTLGIGTFGKVKIAEHKLTGHRVAIKIINCRQ--MRN-
TaCIPK20                  (47) LPKPPTTFPLSSHLRFTVPSVVHYHHRQAHIPVQPLLDQGLLLGEE
TaCIPK17                  (20) LLGAYELGRTLGEGSFGKVKHARHRATGDHFAVKILDRGRVLSLRG
TaCIPK21                   (9) RMGKYEMGRTLGEGHFGKVRLARHADTGRAFAIKILDRQRILAMK-
AtSOS2 AAF62923.1          (7) RVGKYEVGRTIGEGTFAKVKFARNTDTGDNVAIKIMAKSTILKNR-
TaCIPK24                   (9) LVGRYEVGRTIGQGSFAKVKFAVDADTGAPVAMKVLDKATILNHR-
TaCIPK8                   (12) RVGKYEVGRTIGEGTFAKVKFAQNTETGESVAMKVLDRSSILKHK-
TaCIPK23                  (21) RVGRYELGRTLGEGTFAKVKFARNVETGENVAIKILDKDKVLKHK-
TaCIPK3                   (15) RVGKYELGRTIGEGTFAKVRFAKDTESGDPVAIKILDKAKVHKHG-
TaCIPK31                  (16) RVGKYELGRTIGEGTFAKVRFAKNTETMEPVAIKILDKEKVQKLR-
TaCIPK32                   (9) RVGKYELGRTIGEGTFAKVRFARNTETGDPVAIKILDKEKLLKHK-
TaCIPK9                   (13) RVGPYELGKTVGEGSFAKVKIAKDTRNAATCAIKVLDRNHVLRHK-
TaCIPK22                  (17) LQGRYELGRVLGRGGSSKVYRARDIRTGVSVAVKAVRKPHHPCSPE
TaCIPK29                  (27) LLGRYELGGLLGRGASAKVYRARDLLTGRDVAIKSFPNPRSGARDG
TaCIPK4                   (13) LLGKYELGRLLGRGTFAKVYLAHTVTGGEPVAVKVIDKAEVMGTEG
TaCIPK7                   (15) LLGKYELGRLLGRGTFAKVYHARSLVGGEAVAIKVLDKPELAATAG
TaCIPK12                  (42) LLGRYELGRVLGHGTFAKVYHARHADTGETVAIKVLDKEKALRTG-
TaCIPK19                  (42) LMGRYELGRVLGKGTFAKVYHARHVQTGESVAIKVLDREKAVRSG-
TaCIPK25                   (7) LPARYEQVKLLGEGNFAKVYLARHMDTKEEVAIKVMDKEKLIKLG-
TaCIPK13                  (33) LLGRFEVGRLLGQGNFAKVYQARNVATGEEVAIKVIEKEKVFKSG-
TaCIPK10                   (9) LMERYEMGRLLGQGSFAKVYYGRSLKTSQSVAIKVIDKEKIFKCG-
TaCIPK2                    (9) LMGKYEMGKMLGQGTFAKVYHARNIETSQSVAIKVTDKEKVLKGG-
TaCIPK11                   (9) LMGRYEIGKQLGQGNFAKVYYARNLATGQAVAIKMINKDKVTKVG-
TaCIPK26                   (8) LMDRYEIGRQLGQGNFAKVYYGRNLAGGQAVAIKMIDKEKVSRVG-
TaCIPK28                   (7) LTQRYEIGRQLGQGTFAKVYYARNLANGQSVAIKILDKDKILKVG-
TaCIPK14                  (18) LMERYELGRLLGKGTFGKVHYARSLESNRSVAIKMLDKEKVLKVG-
TaCIPK15                   (8) VMGRYELGRLLGKGAFGKVHYAKNLESNRGVAIKILDKEKVLKVG-
TaCIPK5                    (8) LMNRYELGRMLGQGTFAKVYHARSLATNQSVAIKVIDKEKVLRVG-
TaCIPK27                  (12) LQGRYELGRVLGHGNFGRVHAARDLHTGRGVAVKVVAKDKVERAG-
TaCIPK6                   (12) LQGRYELGRVLGHGNFGRVHAARDLHTGRGVAVKVVAKDKVERAG-
TaCIPK30                  (12) IMGRYKLGRLLGRGSFAKVYKAHNISTGEVVAIKVFDKEAVRQSG-
TaCIPK16                   (1) ----------------------------------------------
Consensus                 (47) LMGRYELGR LG GTFAKVY AR L TG  VAIKVLDK KV K
```

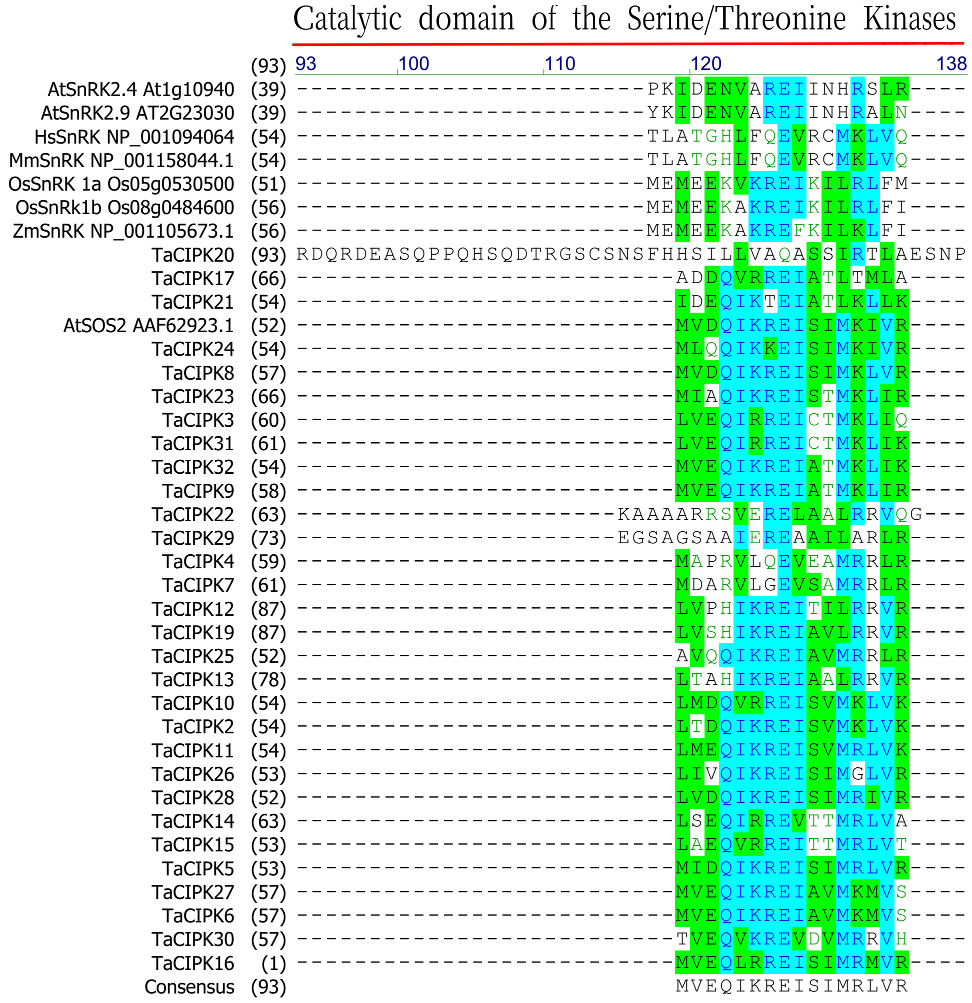
Catalytic domain of the Serine/Threonine Kinases
(93) 93 100 110 120 138
AtSnRK2.4 At1g10940 (39) ------------------------PKIDENVAREIINHRSLR----
AtSnRK2.9 AT2G23030 (39) ------------------------YKIDENVAREIINHRALN----
HsSnRK NP_001094064 (54) ------------------------TLATGHLFQEVRCMKLVQ----
MmSnRK NP_001158044.1 (54) ------------------------TLATGHLFQEVRCMKLVQ----
OsSnRK 1a Os05g0530500 (51) ------------------------MEMEEKVKREIKILRLFM----
OsSnRk1b Os08g0484600 (56) ------------------------MEMEEKAKREIKILRLFI----
ZmSnRK NP_001105673.1 (56) ------------------------MEMEEKAKREFKILKLFI----
TaCIPK20 (93) RDQRDEASQPPQHSQDTRGSCSNSFHHSILLVAQASSIRTLAESNP
TaCIPK17 (66) --------------------------ADDQVRREIATLTMLA----
TaCIPK21 (54) --------------------------IDEQIKTEIATLKLLK----
AtSOS2 AAF62923.1 (52) --------------------------MVDQIKREISIMKIVR----
TaCIPK24 (54) --------------------------MLQQIKKEISIMKIVR----
TaCIPK8 (57) --------------------------MVDQIKREISIMKLVR----
TaCIPK23 (66) --------------------------MIAQIKREISTMKLIR----
TaCIPK3 (60) --------------------------LVEQIRREICTMKLIQ----
TaCIPK31 (61) --------------------------LVEQIRREICTMKLIK----
TaCIPK32 (54) --------------------------MVEQIKREIATMKLIK----
TaCIPK9 (58) --------------------------MVEQIKREIATMKLIR----
TaCIPK22 (63) ----------------------KAAAARRSVERELAALRRVQG---
TaCIPK29 (73) ----------------------EGSAGSAAIEREAAILARLR----
TaCIPK4 (59) --------------------------MAPRVLQEVEAMRRLR----
TaCIPK7 (61) --------------------------MDARVLGEVSAMRRLR----
TaCIPK12 (87) --------------------------LVPHIKREITILRRVR----
TaCIPK19 (87) --------------------------LVSHIKREIAVLRRVR----
TaCIPK25 (52) --------------------------AVQQIKREIAVMRRLR----
TaCIPK13 (78) --------------------------LTAHIKREIAALRRVR----
TaCIPK10 (54) --------------------------LMDQVRREISVMKLVK----
TaCIPK2 (54) --------------------------LTDQIKREISVMKLVK----
TaCIPK11 (54) --------------------------LMEQIKREISVMRLVK----
TaCIPK26 (53) --------------------------LIVQIKREISIMGLVR----
TaCIPK28 (52) --------------------------LVDQIKREISIMRIVR----
TaCIPK14 (63) --------------------------LSEQIRREVTTMRLVA----
TaCIPK15 (53) --------------------------LAEQVRREITTMRLVT----
TaCIPK5 (53) --------------------------MIDQIKREISIMRLVR----
TaCIPK27 (57) --------------------------MVEQIKREIAVMKMVS----
TaCIPK6 (57) --------------------------MVEQIKREIAVMKMVS----
TaCIPK30 (57) --------------------------TVEQVKREVDVMRRVH----
TaCIPK16 (1) --------------------------MVEQLRREISIMRMVR----
Consensus (93) MVEQIKREISIMRLVR

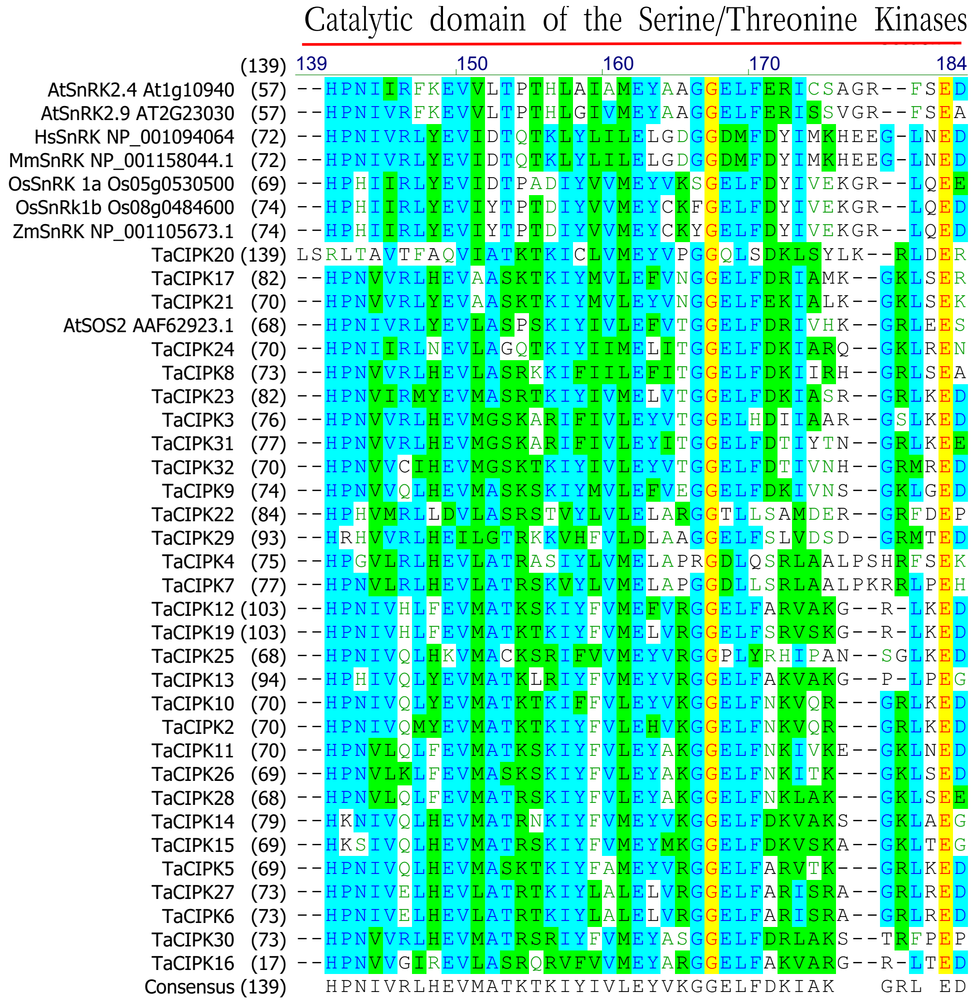
Catalytic domain of the Serine/Threonine Kinases
(139) 139 150 160 170 184
AtSnRK2.4 At1g10940 (57) --HPNIIRFKEVVLTPTHLAIAMEYAAGGELFERICSAGR--FSED
AtSnRK2.9 AT2G23030 (57) --HPNIVRFKEVVLTPTHLGIVMEYAAGGELFERISSVGR--FSEA
HsSnRK NP_001094064 (72) --HPNIVRLYEVIDTQTKLYLILELGDGGDMFDYIMKHEEG-LNED
MmSnRK NP_001158044.1 (72) --HPNIVRLYEVIDTQTKLYLILELGDGGDMFDYIMKHEEG-LNED
OsSnRK 1a Os05g0530500 (69) --HPHIIRLYEVIDTPADIYVVMEYVKSGELFDYIVEKGR--LQEE
OsSnRk1b Os08g0484600 (74) --HPHIIRLYEVIYTPTDIYVVMEYCKFGELFDYIVEKGR--LQED
ZmSnRK NP_001105673.1 (74) --HPHIIRLYEVIYTPTDIYVVMEYCKYGELFDYIVEKGR--LQED
TaCIPK20 (139) LSRLTAVTFAQVIATKTKICLVMEYVPGGQLSDKLSYLK--RLDER
TaCIPK17 (82) --HPNVVRLHEVAASKTKIYMVLEFVNGGELFDRIAMK--GKLSER
TaCIPK21 (70) --HPNVVRLYEVAASKTKIYMVLEYVNGGELFEKIALK--GKLSEK
AtSOS2 AAF62923.1 (68) --HPNIVRLYEVLASPSKIYIVLEFVTGGELFDRIVHK--GRLEES
TaCIPK24 (70) --HPNIIRLNEVLAGQTKIYIIMELITGGELFDKIARQ--GKLREN
TaCIPK8 (73) --HPNVVRLHEVLASRKKIFIILEFITGGELFDKIIRH--GRLSEA
TaCIPK23 (82) --HPNVIRMYEVMASRTKIYIVMELVTGGELFDKIASR--GRLKED
TaCIPK3 (76) --HPNVVRLHEVMGSKARIFIVLEYVTGGELHDIIAAR--GSLKED
TaCIPK31 (77) --HPNVVRLHEVMGSKARIFIVLEYITGGELFDTIYTN--GRLKEE
TaCIPK32 (70) --HPNVVCIHEVMGSKTKIYIVLEYVTGGELFDTIVNH--GRMRED
TaCIPK9 (74) --HPNVVQLHEVMASKSKIYMVLEFVEGGELFDKIVNS--GKLGED
TaCIPK22 (84) --HPHVMRLLDVLASRSTVYLVLELARGGTLLSAMDER--GRFDEP
TaCIPK29 (93) --HRHVVRLHEILGTRKKVHFVLDLAAGGELFSLVDSD--GRMTED
TaCIPK4 (75) --HPGVLRLHEVLATRASIYLVMELAPRGDLQSRLAALPSHRFSEK
TaCIPK7 (77) --HPNVLRLHEVLATRSKVYLVMELAPGGDLLSRLAALPKRRLPEH
TaCIPK12 (103) --HPNIVHLFEVMATKSKIYFVMEFVRGGELFARVAKG--R-LKED
TaCIPK19 (103) --HPNIVHLFEVMATKTKIYFVMELVRGGELFSRVSKG--R-LKED
TaCIPK25 (68) --HPNIVQLHKVMACKSRIFVVMEYVRGGPLYRHIPAN--SGLKED
TaCIPK13 (94) --HPHIVQLYEVMATKLRIYFVMEYVRGGELFAKVAKG--P-LPEG
TaCIPK10 (70) --HPNIVQLYEVMATKTKIFFVLEYVKGGELFNKVQR---GRLKED
TaCIPK2 (70) --HPNIVQMYEVMATKTKIYFVLEHVKGGELFNKVQR---GRLKED
TaCIPK11 (70) --HPNVLQLFEVMATKSKIYFVLEYAKGGELFNKIVKE--GKLNED
TaCIPK26 (69) --HPNVLKLFEVMASKSKIYFVLEYAKGGELFNKITK---GKLSED
TaCIPK28 (68) --HPNVLQLFEVMATRSKIYFVLEYAKGGELFNKLAK---GKLSEE
TaCIPK14 (79) --HKNIVQLHEVMATRNKIYFVMEYVKGGELFDKVAKS--GKLAEG
TaCIPK15 (69) --HKSIVQLHEVMATRSKIYFVMEYMKGGELFDKVSKA--GKLTEG
TaCIPK5 (69) --HPNIVQLHEVMASKSKIYFAMEYVRGGELFARVTK---GRLKED
TaCIPK27 (73) --HPNIVELHEVLATRTKIYLALELVRGGELFARISRA--GRLRED
TaCIPK6 (73) --HPNIVELHEVLATRTKIYLALELVRGGELFARISRA--GRLRED
TaCIPK30 (73) --HPNVVRLHEVMATRSRIYFVMEYASGGELFDRLAKS--TRFPEP
TaCIPK16 (17) --HPNVVGIREVLASRQRVFVVMEYARGGELFAKVARG--R-LTED
Consensus (139) HPNIVRLHEVMATKTKIYIVLEYVKGGELFDKIAK GRL ED

Catalytic domain of the Serine/Threonine Kinases

```
                        (185) 185     190            200            210            220            230
AtSnRK2.4 At1g10940      (99) EARYFFQQLISGVSYCHAMQICHRDLKL---ENTLLDGSPAPRLKI
AtSnRK2.9 AT2G23030      (99) EARYFFQQLICGVHYLHALQICHRDLKL---ENTLLDGSPAPRLKI
HsSnRK NP_001094064     (115) LAKKYFAQIVHAISYCHKLHVVHRDLKP---ENVVFFEKQG-LVKL
MmSnRK NP_001158044.1   (115) LAKKYFAQIVHAISYCHKLHVVHRDLKP---ENVVFFEKQG-LVKL
OsSnRK 1a Os05g0530500  (111) EARRFFQQIISGVEYCHRNMVVHRDLKP---ENLLLDSKCN--VKI
OsSnRk1b Os08g0484600   (116) EARRIFQQIISGVEYCHRNMVVHRDLKP---ENLLLDSKYN--VKL
ZmSnRK NP_001105673.1   (116) EARRIFQQIISGVEYCHRNMVVHRDLKP---ENLLLDSKYN--VKL
TaCIPK20                (183) EAKKYFYQLIDAVDYCHRRGVFHRDLKCQQPENLLLDNQGN--LKV
TaCIPK17                (124) EGRRLFQQLIDGVSYCHGKGVYHRDLKP---ENVLIDRKGN--IKI
TaCIPK21                (112) EGRKLFQQLMDAISYCHERGVYHRDLKP---ENVLVDAKGN--IKV
AtSOS2 AAF62923.1       (110) ESRKYFQQLVDAVAHCHCKGVYHRDLKP---ENLLLDTNGN--LKV
TaCIPK24                (112) EARKYFQQLIDAINYCHSKGVYHRDLKP---ENLLLDSRGN--LKV
TaCIPK8                 (115) DARKYFQQLIDGVDFCHSKGVYHRDLKP---ENLLLDSQGN--LKI
TaCIPK23                (124) DARKYFQQLINAVDYCHSRGVYHRDLKP---ENLLLDANGT--LKV
TaCIPK3                 (118) EARRYFQQLINAVDYCHSRGVYHRDLKL---ENLLLDTAGN--LKV
TaCIPK31                (119) EARKYFQQLINAVDYCHSRGVYHRDLKL---ENLLLDAAGN--LKV
TaCIPK32                (112) EARRYFQQLINAVDYCHSRGVYHRDLKP---ENLLLDSCGN--LKV
TaCIPK9                 (116) EARRYFHQLINAVDYCHSRGVYHRDLKP---ENLLLDSYGA--LKV
TaCIPK22                (126) TSRRLFVQLVSALAHVHSRGVFHRDVKP---ENLLLDEHGD--LKL
TaCIPK29                (135) LARHYFRQLVSAVRYCHSRGVYHRDIKP---ENLLLDGDGD--LKV
TaCIPK4                 (119) AARRVFVQLTVALAHCHARGVTHRDLKP---QNLLLDGAGN--LKV
TaCIPK7                 (121) AARRVFLQLVSALIYCHARGVSHRDVKP---QNVLIDADGN--LKV
TaCIPK12                (144) TARRYFQQLISAVGFCHARGVFHRDLKP---ENLLVDERGD--LKV
TaCIPK19                (144) IARRYFQHLISAVGFCHTRGVFHRDLKP---ENLLVDEAGN--LKV
TaCIPK25                (110) ETRRIFQQLVSALTFCHAQGVYHRDIKP---DNLLVDEHGN--LKV
TaCIPK13                (135) EARRYFQQLVSAVAFCHARGVYHRDIKP---ENLLVDDAGD--LKV
TaCIPK10                (111) VARKYFQQLNSAVDFCHSRGVYHRDLKP---ENLLLDENRN--LKI
TaCIPK2                 (111) AARKYFQQLICAVDFCHSRGVYHRDLKP---ENLLLDENSN--LKV
TaCIPK11                (112) AARRYFHQLISAIDYCHSRGVYHRDLKP---ENLLLDENEN--LKV
TaCIPK26                (110) AARKYFHQLISAVDYCHSRGVYHRDLKP---ENLLLDENEN--LKV
TaCIPK28                (109) GARTYFHQLISAIDYCHSRGVYHRDLKP---ENLLVDEYGT--LRV
TaCIPK14                (121) AAHKYFQQLISAVDYCHSQGVYHRDLKL---ENLLLDENEN--LKV
TaCIPK15                (111) AAHKYFQQLISAVDYCHSRGVYHRDLKP---ENLLLDGNEN--LKV
TaCIPK5                 (110) AARKYFQQLIGAVDFCHSRDVYHRDLKP---ENLLVDEHGN--LKV
TaCIPK27                (115) VARRYFRQLISAVDFCHGRGVYHRDLKP---ENLLLDEAGN--LKV
TaCIPK6                 (115) VARRYFRQLISAVDFCHGRGVYHRDLKP---ENLLLDEAGN--LKV
TaCIPK30                (115) VARRYFQQLVTAVEFCHSRGVYHRDLKP---ENLLLDAHGN--LKV
TaCIPK16                 (58) AARKYFQQLVAAVAFCHSRGVAHRDLKP---ENLLLDEEGR--LKV
Consensus               (185)  ARRYFQQLISAVDYCHSRGVYHRDLKP   ENLLLD  GN  LKV
```

Activiation loop

Catalytic domain of the Serine/Threonine Kinases

```
                        (231) 231      240            250            260            276
AtSnRK2.4 At1g10940     (142) CDFGYSKSSLLHSR------PKSTVGT-PAYIAPEVLSRREYDGKM
AtSnRK2.9 AT2G23030     (142) CDFGYSKSSVLHSN------PKSTVGT-PAYIAPEVFCRSEYDGKS
HsSnRK NP_001094064     (157) TDFGFSNKFQPGKK------LTTSCGS-LAYSAPEILLGDEYDAPA
MmSnRK NP_001158044.1   (157) TDFGFSNKFQPGKK------LTTSCGS-LAYSAPEILLGDEYDAPA
OsSnRK 1a Os05g0530500  (152) ADFGLSNVMRDGHF------LKTSCGS-PNYAAPEVISGKLYAGPE
OsSnRk1b Os08g0484600   (157) ADFGLSNVMHDGHF------LKTSCGS-PNYAAPEVISGKLYAGPE
ZmSnRK NP_001105673.1   (157) ADFGLSNVMHDGHF------LKTSCGS-PNYAAPEVISGKLYAGPE
TaCIPK20                (227) SDFGLSVLRK---------------VV---------IQHKTYDGAA
TaCIPK17                (165) SDFGLCALPQHLGK----DGLLHTTCGSPNYIAPEVLQNRGYDGSL
TaCIPK21                (153) SDFGLSALPQHQRK----DGLLHTTCGSPNYIAPEVLNKGYDGSM
AtSOS2 AAF62923.1       (151) SDFGLSALPQEG------VELLRTTCGTPNYVAPEVLSGQGYDGSA
TaCIPK24                (153) SDFGLSSLSQN--------GFLHTTCGTPNYVAPEVLSDGGYDGSA
TaCIPK8                 (156) SDFGLSAWPAQG------AALLRTTCGTPNYVAPEVLSHKGYDGAL
TaCIPK23                (165) SDFGLSALSQQVRE----DGLLHTTCGTPNYVAPEVINNKGYDGAK
TaCIPK3                 (159) SDFGLSAISEQAKA----DGLLHTTCGTPNYVAPEVIQDKGYNGAL
TaCIPK31                (160) SDFGLSALTEQVKA----DGLLHTTCGTPNYVAPEVIEDRGYDGAA
TaCIPK32                (153) SDFGLSALSQQLKD----DGLLHTTCGTPNYVAPEVLEDQGYDGAM
TaCIPK9                 (157) SDFGLSAFSPQTKE----DGLLHTACGTPNYVAPEVLADKGYDGMA
TaCIPK22                (167) TDFGLCALADRHLG---ADGLAATRCGSPAYVAPEILYKKRYDAGK
TaCIPK29                (176) ADFGLGAVAD--------ESLHHTLCGTPAYVAPEILSKQGYHPAK
TaCIPK4                 (160) SDFGLSALPDSFRE----DGRLHTACGTAAYAAPEVLRNKAYDGAK
TaCIPK7                 (162) CDFGLAALPESHRD----DGRLHTACGTPAFAAPEVLRRKAYDGVK
TaCIPK12                (185) SDFGLSAVADQFHP----DGLLHTFCGTPSYVAPEMLARRGYDGAK
TaCIPK19                (185) SDFGLSAVAEPFQP----EGLLHTFCGTPAYVAPEVLARRGYEGAK
TaCIPK25                (151) ADFGLSAHADTARR----EALLHTVCGTPLYVPPEVFARRGYDGAK
TaCIPK13                (176) SDFGLSAVAEQMRH----DGLFHTFCGTPAYVAPEVLSRRGYDAAK
TaCIPK10                (152) SDFGLSALAECKRQ----DGLLHTTCGTPAYVAPELISKKGYDGAK
TaCIPK2                 (152) SDFGLSTISECRRL----DGLLHTSCGTPAYVAPEVINRKGYDGAK
TaCIPK11                (153) SDFGLSALADCTRQ----DGLLHTTCGTPAYVAPEVLSRKGYDGAK
TaCIPK26                (151) SDFGLSALAESTRQ----DGLLHTTCGTPAYVAPEVLSRRGYDGAK
TaCIPK28                (150) SDFGLSALTKSKWR----DGLLHTACGTPAYVAPEVLSRKGYNGAK
TaCIPK14                (162) SDFGLSALSESKRQ----DGLLHTTCGTPAYVAPEVISKTGYDGAK
TaCIPK15                (152) SDFGLSALSESKSQ----DGMLHTTCGSPAYVAPEVISKGGYDGAK
TaCIPK5                 (151) SDFGLSALKESQKQ----DGLLHTTCGTPAYVAPEIINKKGYDGEK
TaCIPK27                (156) ADFGLSALAGHARP----DGLLHTACGTPAYVAPEVLGGNGYDGAK
TaCIPK6                 (156) ADFGLSALAGHARP----DGLLHTACGTPAYVAPEVLGGNGYDGAK
TaCIPK30                (156) SDFGLSALADAAASRHGGDALLHTTCGTPSYVAPEVILKRGYDGAK
TaCIPK16                 (99) TDFGLAALPEQLRH----DGLLHTQCGTPAYVAPEVLRKRGYDGAR
Consensus               (231) SDFGLSALAE   R    DGLLHT CGTPAYVAPEVLSRKGYDGAK
```

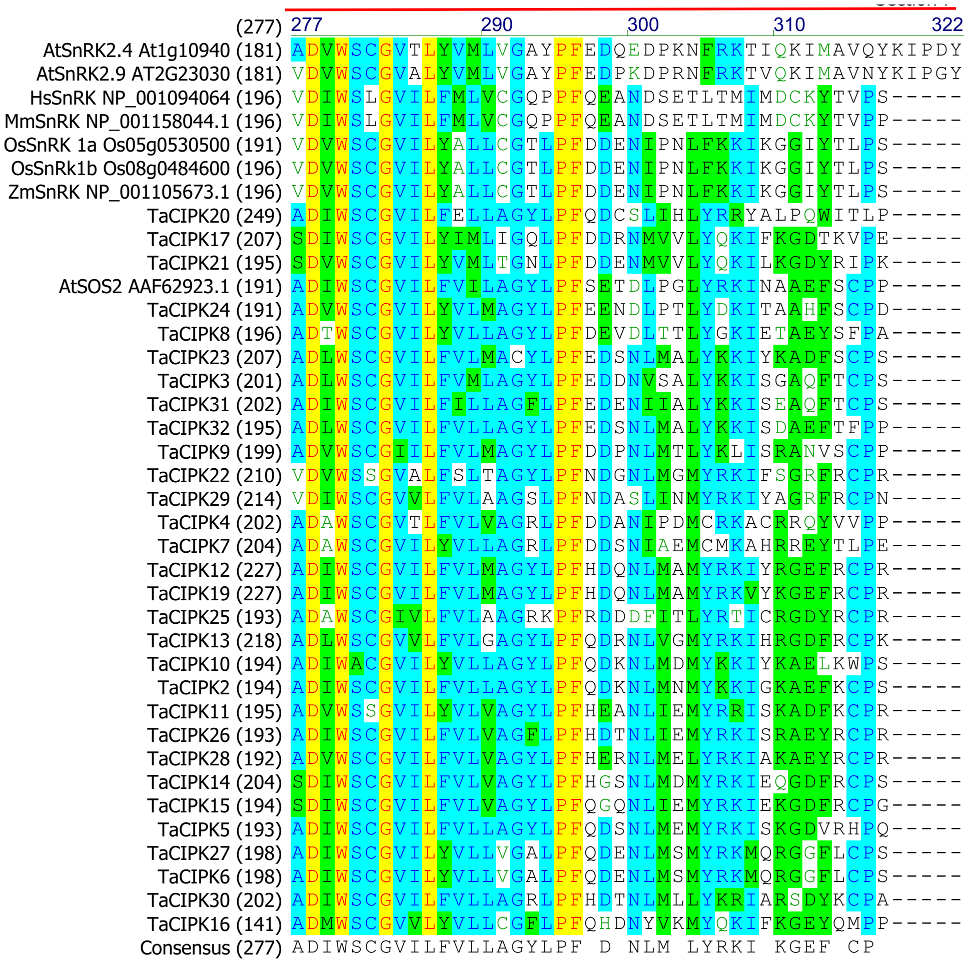

Catalytic domain of the Serine/Threonine Kinases

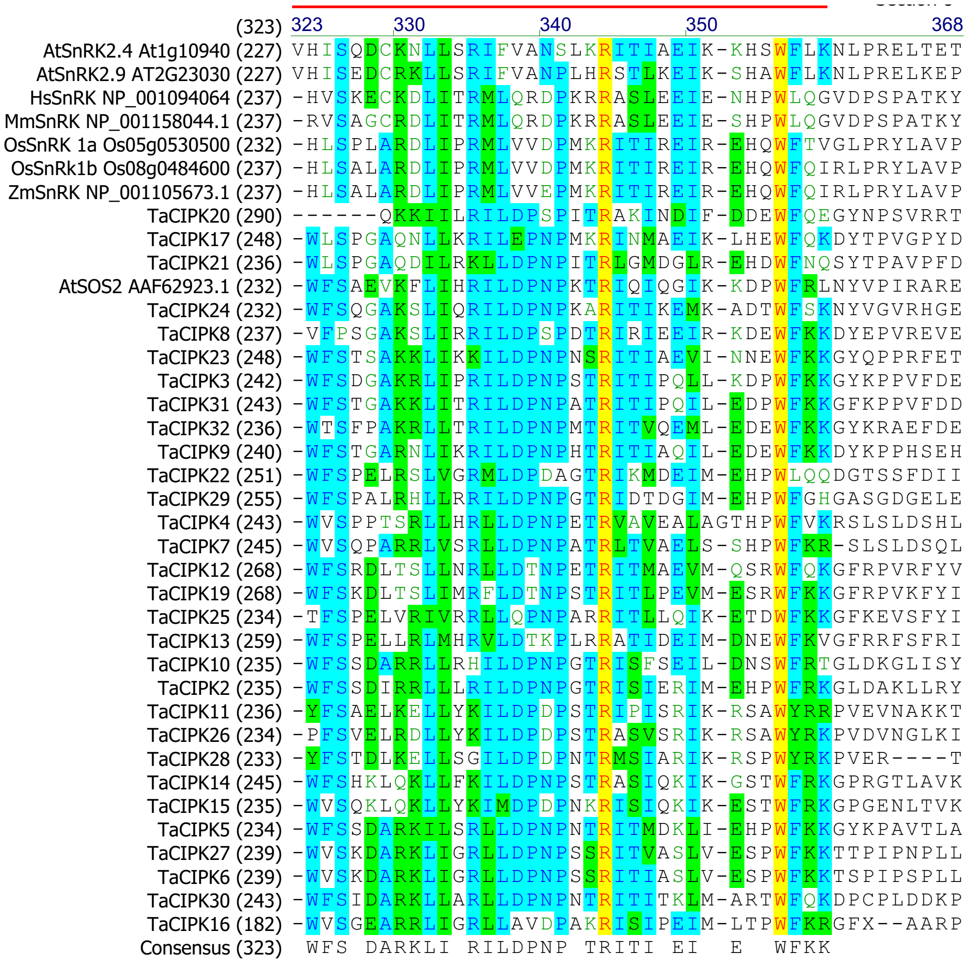

```
                              (369) 369        380       390       400          414
          AtSnRK2.4 At1g10940 (272) AQAAYFKKEN-------------PTFSLQTVEEIMK----------
          AtSnRK2.9 AT2G23030 (272) AQAIYYQRNVNL-----------INFSPQRVEEIMK----------
          HsSnRK NP_001094064 (281) NIPLVSYKNLSEEEHN-------SIIQRMVLGDIAD----------
        MmSnRK NP_001158044.1 (281) NIPLVSYKNLSEEEHN-------SIIQRMVLGDIAD----------
        OsSnRK 1a Os05g0530500 (276) PPDTAQQVKKLD-----------DETLNDVINMGFD----------
         OsSnRk1b Os08g0484600 (281) PPDTAQQAKMID-----------EDTLQDVVNLGYE----------
        ZmSnRK NP_001105673.1 (281) PPDTTQQAKMID-----------EDTLRDVVNMGFN----------
                     TaCIPK20 (329) GSDDGDDCVDLDEAGTDSDGSHSTEVREAGGANPEP----------
                     TaCIPK17 (292) DDDEDVRLGAILPVKQQ--------IS-EAPGDK------------
                     TaCIPK21 (280) DDDDNY-VGDDNSHMTK--------NN-GIQDNP------------
             AtSOS2 AAF62923.1 (276) EEEVNLDDIRAVFDGIEGSY----VAENVERNDE------------
                     TaCIPK24 (276) DENVSLDDVQAAFDNIEDKY----VSEQVTRNDG------------
                      TaCIPK8 (281) NEEVNLDDVNAAFDDPEED------NQHTFDDEA------------
                     TaCIPK23 (292) -AEVNLDDINSIFNESGDP-----AQLVVERREE------------
                      TaCIPK3 (286) KHQTSLDDVDAALGDS--E-----EKQVEEEMEG------------
                     TaCIPK31 (287) KYETSFDDVYAAFGDS--E-----DQHVKEETEH------------
                     TaCIPK32 (280) KYDTTLDDVDAVFNDS--E-----EHHVTEKKEE------------
                      TaCIPK9 (284) NEDVSLEDVDAAFDSS--E-----EHLVAERRE-------------
                     TaCIPK22 (295) RAPSSDPRPE-------------VMKWEAEMEQVR-----------
                     TaCIPK29 (299) KLMRGHEEEA-------------WFETEFKEDMAR-----------
                      TaCIPK4 (288) DGLLDSQPER---------------AVAFRAP--------------
                      TaCIPK7 (288) GSLLGGAAER---------------DLLFQAPP-------------
                     TaCIPK12 (312) EDDQLHSLG-------------------------------------
                     TaCIPK19 (312) EDDQLYNVIDA--ENDMLD-----LGLPDPLPQPLLPPPSSPSPQE
                     TaCIPK25 (278) DNKDCLRSLDGSEEPDLCD-----SDSEDETAMSSSSSGSSSPVAH
                     TaCIPK13 (303) EDDRSFTCFDLDDGDAYAP-----TSPPDTPRTADGSDYGDATDQQ
                     TaCIPK10 (279) NTPT-EGIVAVDMDP---------TCDPFSSCTTET---IQEA---
                      TaCIPK2 (279) NLQAKDAVPAADMTA---------TSDSLSCSNSATEGKEQEA---
                     TaCIPK11 (280) EPEATHNTFSAEAAS---------SGSTGCSTS-GG---NQGS---
                     TaCIPK26 (278) KQETRDKVQKGEPTT---------SESTEGSNSEVN---QEAS---
                     TaCIPK28 (273) P-LRKDKTYASEAAA---------SGLPCRKSS-------------
                     TaCIPK14 (289) ERTPSENVTTNAPPT---------AGVRSRKNT-HE---DVKP---
                     TaCIPK15 (279) ERLPSENANTDAVPT---------LGVRRRKNS-HE---DAKP---
                      TaCIPK5 (278) TPRASKDLNDVQAAF---------STDQKDGKGNRAE-QPNSP---
                     TaCIPK27 (283) EPVPSAAARG------N--------GEDKDEP--------------
                      TaCIPK6 (283) EAVPSAAARG------N--------GEDKDEP--------------
                     TaCIPK30 (287) LDTSETAVFLGKEARGHHDDNQPEGATRKRKRSKVTASSPTIN---
                     TaCIPK16 (224) SSPVTPRKWDDDNAAALID-----GSEDSSGN--------------
                    Consensus (369)
```

NAF/FISL

```
                              (415) 415  420       430       440       450       460
          AtSnRK2.4 At1g10940 (295) -----------------------------------IVADAKTPPPV
          AtSnRK2.9 AT2G23030 (297) -----------------------------------IVGEARTIPNL
          HsSnRK NP_001094064 (310) -----------------------------------RDAIVEALETN
        MmSnRK NP_001158044.1 (310) -----------------------------------RDAIVEALETN
        OsSnRK 1a Os05g0530500 (301) -----------------------------------KNQLIESLHKR
         OsSnRk1b Os08g0484600 (306) -----------------------------------KDHVCESLRNR
        ZmSnRK NP_001105673.1 (306) -----------------------------------KNHVCESLCSR
                     TaCIPK20 (365) ------------------------------------EQFINAFRLI
                     TaCIPK17 (317) -----------------------------------STHQMNAFQLI
                     TaCIPK21 (304) -----------------------------------AINQMNAFQLI
             AtSOS2 AAF62923.1 (306) -----------------------------------GPLMMNAFEMI
                     TaCIPK24 (306) -----------------------------------GPLMMNAFEMI
                      TaCIPK8 (309) -----------------------------------GPLTLNAFDLI
                     TaCIPK23 (320) -----------------------------------RPAVMNAFELI
                      TaCIPK3 (313) -----------------------------------QPASMNAFELI
                     TaCIPK31 (314) -----------------------------------KPTSMNAFELI
                     TaCIPK32 (307) -----------------------------------EPVALNAFELI
                      TaCIPK9 (310) -----------------------------------KPESMNAFALI
                     TaCIPK22 (317) --------------------------------------ELNAFDII
                     TaCIPK29 (321) --------------------------------------DMTAFDIL
                      TaCIPK4 (305) --------------------------------------SVNAFDII
                      TaCIPK7 (306) --------------------------------------TLNAFDII
                     TaCIPK12 (321) ----------------------------------------------
                     TaCIPK19 (351) V-DGDDSGSESDASVVSCPATSSFEERHRLRGPLPRPASLNAFDII
                     TaCIPK25 (319) G-DGGGMHTSVSAPSLVNLEKMHIAAARAPEPRIRRIKSMNAFDII
                     TaCIPK13 (344) QKISGGMTSCGSAPSLLEGRFGQLGGSS------RRRSSLNAFDLI
                     TaCIPK10 (309) ----------------------------------TELTNLNAFDII
                      TaCIPK2 (313) ----------------------------------KKLSNLNAFDII
                     TaCIPK11 (310) ----------------------------------LSLPNLNAFDII
                     TaCIPK26 (309) ----------------------------------SSLTNLNAFDII
                     TaCIPK28 (296) ----------------------------------DGPASMNAFNII
                     TaCIPK14 (319) ----------------------------------LMVTNLNAFEII
                     TaCIPK15 (309) ----------------------------------LAVTNLNAFEII
                      TaCIPK5 (311) ----------------------------------LKPASLNAFDII
                     TaCIPK27 (301) ------------------------------------PEALNAFHLI
                      TaCIPK6 (301) ------------------------------------PEALNAFHLI
                     TaCIPK30 (330) ----------------------------------VRPSSMNAFDII
                     TaCIPK16 (251) ----------------------------------ISPRTCNAFQLI
                    Consensus (415)                                        LNAFDII
```

```
                               NAF/FISL
                         (461) 461           470               480               490                506
AtSnRK2.4 At1g10940 (306) SRSIGGFGWGGNGDADGKEED----------------AEDVEEEEEE
AtSnRK2.9 AT2G23030 (308) SRPVESLGS----DKK-------------------------DDD-EE
HsSnRK NP_001094064 (321) RYNHITATYFLLAERILREKQEKEIQTRSASPSNIKAQFRQSWPTK
MmSnRK NP_001158044.1 (321) RYNHITATYFLLAERILREKQEKEIQTRSASPSNIKAQFRQSWPTK
OsSnRK 1a Os05g0530500 (312) LQNEATVAYYLLLDNRLRTTSGY------------LGAEFHESMESS
OsSnRk1b Os08g0484600 (317) LQNEATVAYYLLLDNRFRATSGY------------LGADYQESLERN
ZmSnRK NP_001105673.1 (317) LQNEATVAYYLLLDNRFRATSGY------------LGADYQESMDRN
TaCIPK20 (375) A-TCRDLDLSGLFQEQKTKLGSPHSVQETLGIITAAARDVSLSARR
TaCIPK17 (328) G-MASSLDLSGFFEEEGVS----------------QRKIRFTSTLTP
TaCIPK21 (315) G-MSSCLDLSGFFEKEDVS----------------ERKTRFASNYPP
AtSOS2 AAF62923.1 (317) T-LSQGLNLSALFDRRQD-F--------------VKRQTRFVSRREP
TaCIPK24 (317) T-LSQGLNLSSLFDRQQE-Y--------------VKRQTRFVSRKPA
TaCIPK8 (320) I-LSQGLNLSALFDRRQD-Y--------------GKLQNRFLSRKPA
TaCIPK23 (331) S-TSQGLNLGTLFEKQTDS---------------VKRETRFASRLPA
TaCIPK3 (324) S-LNKGLNLRNFFEADKK----------------YRRETRFTSQCPP
TaCIPK31 (325) S-LNQGLNLDNLFEAKEE----------------HKRETRFTSQCPP
TaCIPK32 (318) S-MSAGLNLGNLFDSEQE----------------FKRETRFTSKSSP
TaCIPK9 (321) S-RSEGFNLGNLFEKEMMGM--------------VKRETSFASQRTP
TaCIPK22 (325) AFAS-GCDLSGLFGPLPDR-----------------VRFAVVGVVI
TaCIPK29 (329) AFSP-GSDLSGLFGAGPG------------------TERVFVGEPA
TaCIPK4 (313) S-TSQWLDLSGMFGESRRSK----------------EKRFVTTASP
TaCIPK7 (314) S-MSPGLDLSGLFGENRRSR----------------EKRFMTTASP
TaCIPK12 (321) ---------DGLFEERGSE------------------VRFISAQPM
TaCIPK19 (396) S-FSRGFNLSGLFEEKGDE------------------VRFISSEPM
TaCIPK25 (364) A-SSPSFDLSGLFEERGEQ------------------LRFVSSAPV
TaCIPK13 (384) S-FSPGFDLSGLFEDGGGEGSSGEGEQ------QQNAARFVSAAPA
TaCIPK10 (321) S-LSSGFDLSGMFEDKSNK-----------------ESKFTSTNTA
TaCIPK2 (325) S-LSTGLDLSGMFEDNDKKR----------------ESKFTSTNSA
TaCIPK11 (322) S-LSTGFNLSGFFEDEYGRR----------------EERFTTRQPV
TaCIPK26 (321) S-LSTGFDLSNLFEEKYGRR----------------EDRFTTRQPA
TaCIPK28 (308) S-LTPGFDLSGLFDERYSQR----------------EARFASNEPP
TaCIPK14 (331) S-FSTGFDLSGLFIREECRK----------------ETRFTSDKPA
TaCIPK15 (321) S-FSTGFDLSGLFVEKESKK----------------EARFTSEQPA
TaCIPK5 (323) S-LSKGFDLSGLFEKDQEQKS---------------NSRFMTQKPA
TaCIPK27 (311) S-LSAGFDLSPLFDQEPPTGRGTRG-----------GVMRFATREPA
TaCIPK6 (311) S-LSAGFDLSPLFDQEPPTGRGTRG-----------GVMRFATREPA
TaCIPK30 (342) S-RSSVLDLAKMFDAEHKTS----------------EARFSTKETT
TaCIPK16 (263) SSMSSGFDLSGLFESEQKA-----------------ATVFTSRAPA
Consensus (461) S  S G DLSGLFE                         RF S  P
```

```
                         (507) 507            520               530               540               552
AtSnRK2.4 At1g10940 (337) VEEEEDDEDEYDKTVKEVHASGEVRIS-------------------
AtSnRK2.9 AT2G23030 (325) EYLDANDEEWYDDYA-------------------------------
HsSnRK NP_001094064 (367) IDVPQDLEDDLTATPLSHATVPQSPARAADSVLNGHR--SKGLCDS
MmSnRK NP_001158044.1 (367) IDVPQDLEDDLTATPLSHATVPQSPARAGDNVLNGHR--SKGLCDP
OsSnRK 1a Os05g0530500 (347) LAQVTPAETPNSATDHRQHGHMESPGFGLRHHFAADRKWALGLQSR
OsSnRk1b Os08g0484600 (352) LNRFASSESASSNTRHYLPGSSDPHASGLRPHYPVERKWALGLQSR
ZmSnRK NP_001105673.1 (352) LNQLASSESSSSGTRNYVPGSSDPHSSGLRPYYPVERKWALGLQSR
TaCIPK20 (420) MGSSMVISQSLAIFEFSILNDLEKSQVKLQDIRLLSRSMLD---LT
TaCIPK17 (358) KDLFDKIDVSATQSGFHVQR--AHSKLKITGNCNGPNNP---SPFL
TaCIPK21 (345) TYLFEKIESNVINMGFQVQK--NNGKLKVIQERKGPTNPRGHGSLL
AtSOS2 AAF62923.1 (348) SEIIANIEAVANSMGFKSHT--RNFKTRLEGLSSIKAGQ-----LA
TaCIPK24 (348) KTIAATIEVVADSMGLKVHS--QNYKLRLEGVSSNKMSP-----FA
TaCIPK8 (351) NVILSSMEVVAQSMGFKTHI--RNYKMRVEGPNANKTSH-----LT
TaCIPK23 (362) NEILSKIEAAAGPMGFNVQK--RNYKLKLQGENPGRKGQ-----LA
TaCIPK3 (354) EEIISRIEEAAKPLGFDIQK--KNYKMRMKNLEAGRKGN-----LN
TaCIPK31 (355) KEIISKIAEAARPLGFDIQK--KNYKMRMENPKAGRKGN-----LN
TaCIPK32 (348) KEILRKIEEAAKPLGFDIHK--KNYKLRLEKVKAGRKGK-----LN
TaCIPK9 (353) QEIMSKIEEACGPLGFNVRK--QNYKMKLKGDKTGRKGH-----LS
TaCIPK22 (353) ASVLDKAEEIGREEGFVMRR---KEEVGCGGIMFEAIQR----EII
TaCIPK29 (356) AAVLARVEDAGKKQGHRVRR---EGKSGAGPVYLEAEAG----DIV
TaCIPK4 (342) EQTLEQLGRAGRKLGYVVVVG-KKGRECQRCPLGGLT---------
TaCIPK7 (343) EQMVEQLGHSGAKLGYFMVG--KKG--VERLPLGGLSGL-----VA
TaCIPK12 (340) ETIVTKLEEIAKMKSFSIRR--KDWRVSIEGTREGEKGP-----LT
TaCIPK19 (423) PDIITKLEEIANVKSFAVRK--KDWRVSLEGTREGVKGP-----LT
TaCIPK25 (391) NTIISKLEEIAGQVSFTART--KDCQVSFEATRNGHKGA-----LA
TaCIPK13 (423) EQILAALERTAAAAGMAVWA-REDGSVIMEGTREGANGA-----LA
TaCIPK10 (349) ATIITKLEDIAKRLRLRLMK-RDGGLLKMQSLQPGRKGV-----MS
TaCIPK2 (354) STIVSKIEDIAKCMRLKLVK-KDGGMMRMESFKPGRKGV-----MS
TaCIPK11 (351) TIVLTKLKELAKRLKLKIKK-KENGVLKLAAPKEGKKGF-----LE
TaCIPK26 (350) EAIFAKLNEVANKLKLKIKK-KENGVLKLAAPKEGMKGI-----LE
TaCIPK28 (337) AAVFVKLEELAQRMKLKVTK-KDNGAMKLAAPREGKKGT-----LE
TaCIPK14 (360) SAIISKLEYVAKALNLRVRK-KDNGVVKMQARKEGRNGA-----VQ
TaCIPK15 (350) SAIVSKLEAVAKTLNLRVRK-KDNGVVKMQVRKEGRNGV-----LQ
TaCIPK5 (353) SAIVSKLEQIAETESFKVKK-QDG-LVTLQGSKEGRKGQ-----LA
TaCIPK27 (346) SGVISRLEGLATGGAMRVTK-SGARGVRLEGAERGRKGR-----LG
TaCIPK6 (346) SGVISRLEGLATGGAMRVTK-SGARGVRLEGAERGRKGR-----LG
TaCIPK30 (371) TAIVSKLGKIAEAGRFSFKLNKEKGRVQLEGSQDGRKRA-----LA
TaCIPK16 (292) ATVFHKLESVGKALRYNTTR-GKGWRIRMEAKADGANGR-----LA
Consensus (507)    IISKLE IA  L F V K        VKL G   G KG     L
```

```
                          (553) 553    560       570       580              598
AtSnRK2.4 At1g10940       (364) ----------------------------------------------
AtSnRK2.9 AT2G23030       (340) ----------------------------------------------
HsSnRK NP_001094064       (411) AKKDDLPELAGPALSTVPPASLKPTASGRKCLFRVEEDEEEDEEDK
MmSnRK NP_001158044.1     (411) AKKDELPELAGPALSTVPPASMKPAASGRKCLFRVEEDEEEDEEDK
OsSnRK 1a Os05g0530500    (393) AHPREIITEVLKALQELNVCWKKIGHYNMKCRWSPSFPSHESMMHN
OsSnRk1b Os08g0484600     (398) AQPREIMIEVLKALEDLNVCWKKNGQYNMKCRWSVGYPQATDMLDV
ZmSnRK NP_001105673.1     (398) AHPREIMVEVLKALQELNVRWKKNGHYNVKCRWCPGFPEVNDTLDA
TaCIPK20                  (463) LSAEVIQVTPAHCVVEVSKSSGDLRAYKEVCQYGNMNAVCSFQSDL
TaCIPK17                  (399) VCAEVFVLGPSLHVVELRKSQGDTAVYKKLCDR-ISSDLGIDN--I
TaCIPK21                  (389) ISAEVFEINESLYVVELKRSCGDCSLYRQLCAT-LSDDLGICKSQQ
AtSOS2 AAF62923.1         (387) VVIEIYEVAPSLFMVDVRKAAGETLEYHKFYKK-LCSKLENIIWRA
TaCIPK24                  (387) VVLEIFEVAPALFMVDVRKVAGDSLEYHRFYKS-LCSKLESIIWRP
TaCIPK8                   (390) IMLQIFEVAPSIFMVELERSAGDTSEYNKFVNN-YCSKLDDIIWKV
TaCIPK23                  (401) IATEVFEVTPSLYMVELRKSNGDTLEFHKFYHS-ISNGLKDVMWKP
TaCIPK3                   (393) VATEVFQVAPSLFVVELKKAKGDTLEFQKFYKT-LSAQIKDVVWVC
TaCIPK31                  (394) VATEVFQVAPSLHVVELKKAKGDTLEFQMFYRS-LSTQLKDVVWKC
TaCIPK32                  (387) VATEILRVGPSLHMVEVRKAKGDTLEFHKFYKN-LSNTLKDVVWKS
TaCIPK9                   (392) VATEVFEVAPTLHMVELRKTGGDTLEFHSFYKN-FSSELKDIVWKT
TaCIPK22                  (392) AMVRVSRLLEEMLMVEVERAS--SSEAPNLWERLQQGLKFSND---
TaCIPK29                  (395) AKVTVFRIADTVSVVEVVKGH--GPEAAAFWNDWLEPAVKPQAV--
TaCIPK4                   (378) ISVEISELELPLLLVEMRLEM-DDGEVRVFSWDQLRVELGDGVVRA
TaCIPK7                   (380) MSMEMSEVAPPLMLVELRLEAGDDEEVQAFGWDELRTELG-EVVMA
TaCIPK12                  (379) IGAEIFELTPSLLVLEVKKKAGDKAEYDDFCNKELKPGMEPLVHHQ
TaCIPK19                  (462) ICAEIFELTPSLVVVEVKKKAGDKEEYDDFCNKELKPGMQHLVHQM
TaCIPK25                  (430) ISTKIFQLTPELVMVQVCKKAGDTAEYRQFCGSELKPGLRGIVDGL
TaCIPK13                  (463) VAAEIYELTPELVVVEVRRKSGGAAEYEEFYRARLKPSLRELMTEE
TaCIPK10                  (389) IDTEIFRIAPNFHLVEIRKTNGDTLEYQKVK-HDMRPALKDIVWAW
TaCIPK2                   (394) IDAEIFEVTPDFHLVELKKTNGDTIEYQKVLNQEMRPALKDIVWAW
TaCIPK11                  (391) LDAEIFDVAPSFLLVELKKTNGDTFEYQKLVKEEVRPALKDIVWVW
TaCIPK26                  (390) FDAEVFEFAPSLHLVELKKTNGDTIEYKQLMKDEIRPALKDVVWAW
TaCIPK28                  (377) FEAEIYELASSFLLVELKKTNGDTMEYQKLLKEDIRPSLKDIVWSW
TaCIPK14                  (400) LDMEIFEITPSHHLIEMKQTSGDPLEYRELLED-IRPALKDIVWAW
TaCIPK15                  (390) FDSEIFEISPSYHLIEMKQTSGDSLEYQKLLEESIRPALKNICLGM
TaCIPK5                   (392) IDAEIFEVTPSFYVVEVKKSAGDTLEYERFCKMGLRPSLKDICWNG
TaCIPK27                  (386) VAAEFFSVAPSVLVVDVKKDGGDTMEYRSFCSDELRPALKDIVWAA
TaCIPK6                   (386) VAAEFFSVAPSVLVVDVKKDGGDTMEYRSFCSDELRPALKDIVWAA
TaCIPK30                  (412) LEAEIFEVAPSVHVVEMRKTGGDSLEFHDFYKQELKPSLGDIVWAW
TaCIPK16                  (332) VTAEVFEVAADVTVVEFAHDGGDALDFNKFCAEDVRPGLADIVWAW
Consensus                 (553) V  EIFEV PSL LVELKK  GDT EY  F    L   L DIVW

                          (599) 599        610       620       630        644
AtSnRK2.4 At1g10940       (364) ----------------------------------------------
AtSnRK2.9 AT2G23030       (340) ----------------------------------------------
HsSnRK NP_001094064       (457) KPMSLSTQVVLRRKPSVTNRLTSRKSAPVLNQIFEEGESDDEFDMD
MmSnRK NP_001158044.1     (457) KPVSLSTQVVLRRKPSVTNRLTSRKSAPVLNQIFEEGESDDEFDMD
OsSnRK 1a Os05g0530500    (439) NHGFGAESAIIETDDSE------KSTHTVKFEIQLYKTRDEKYLLD
OsSnRk1b Os08g0484600     (444) NHSFVDDSIIMDNGDVNG-----RLPAVIKFEIQLYKSRDEKYLLD
ZmSnRK NP_001105673.1     (444) SNSFLGDSTIMDNDDANG-----RLPTVIKFEFQLYKTKDDKYLLD
TaCIPK20                  (509) ISRKHDAQFTDEQCNCIVNTCSSAQACPDC----------------
TaCIPK17                  (442) FGMGSLFDENLPNFDSRAATPLVAL---------------------
TaCIPK21                  (434) LLKNDSIRQELYRFNSSF----------------------------
AtSOS2 AAF62923.1         (432) TEGIPKSEILRTITF-------------------------------
TaCIPK24                  (432) IEVSAKSALLRTTTC-------------------------------
TaCIPK8                   (435) SAEKGKSRTSRLSKR-------------------------------
TaCIPK23                  (446) EGSIAEGDETRHRRSP------------------------------
TaCIPK3                   (438) ESEAEERGST------------------------------------
TaCIPK31                  (439) GGEVEDNSTVA-----------------------------------
TaCIPK32                  (432) DDLQIQPS--------------------------------------
TaCIPK9                   (437) ESNTIAK---------------------------------------
TaCIPK22                  (433) ----------------------------------------------
TaCIPK29                  (437) ----------------------------------------------
TaCIPK4                   (423) WDSREDLQQV------------------------------------
TaCIPK7                   (425) WHGCEEL---------------------------------------
TaCIPK12                  (425) QGSARNVPSDTE----------------------------------
TaCIPK19                  (508) VP-VPNTPTISELAERPSR---------------------------
TaCIPK25                  (476) PEDGLPPTLNVA----------------------------------
TaCIPK13                  (509) PAPRVGSKELTRSV--------------------------------
TaCIPK10                  (434) QGEQP-----------------------------------------
TaCIPK2                   (440) QGEQQPQPQPQPQQQPC-----------------------------
TaCIPK11                  (437) QGDQQQRSEPILQGEQHHSPSPTQQPHDELQPPLQQQEGQDQLHPP
TaCIPK26                  (436) QGESHPLPEKFIRGEQQQSPLPSQQQQQQQSPLPSQQQQQQSPLPS
TaCIPK28                  (423) HGDVQQQAQ-LPQDPQWPPPQQAQQPQDRQWPPPLPPQRLEHLAAN
TaCIPK14                  (445) HGDDHHQQLE------------------------------------
TaCIPK15                  (436) AWG-------------------------------------------
TaCIPK5                   (438) PSEEKLPSVSESVPPTPSSKSTKRNVI-------------------
TaCIPK27                  (432) AAAAAAASPGDPPAPT------------------------------
TaCIPK6                   (432) AAAAAAASPGDPPAPT------------------------------
TaCIPK30                  (458) QGGDSPPPTLTPAVPRPNTNS-------------------------
TaCIPK16                  (378) QGDVPALPGAVV----------------------------------
Consensus                 (599)
```

```
                                                                                  Section [illegible]
                      (645) 645  650       660       670       680       690
    AtSnRK2.4 At1g10940 (364) ----------------------------------------------
    AtSnRK2.9 AT2G23030 (340) ----------------------------------------------
   HsSnRK NP_001094064 (503) ENLPPKLSRLKMNIASPGTVHKRYHRRKSQGRGSSCSSSETSDDDS
 MmSnRK NP_001158044.1 (503) ENLPPKLSRLKMNIASPGTVHKRYHRRKSQGRGSSCSSSETSDDDS
  OsSnRK 1a Os05g0530500 (479) LQRVSGPQLLFLDLCSAFLTQLRVL---------------------
   OsSnRk1b Os08g0484600 (485) MQRVTGPQLLFLDFCAAFLTKLRVL---------------------
  ZmSnRK NP_001105673.1 (485) MQRVTGPQLLFLDFCAAFLTKLRVL---------------------
              TaCIPK20 (539) ----------------------------------------------
              TaCIPK17 (467) ----------------------------------------------
              TaCIPK21 (452) ----------------------------------------------
     AtSOS2 AAF62923.1 (447) ----------------------------------------------
              TaCIPK24 (447) ----------------------------------------------
               TaCIPK8 (450) ----------------------------------------------
              TaCIPK23 (462) ----------------------------------------------
               TaCIPK3 (448) ----------------------------------------------
              TaCIPK31 (450) ----------------------------------------------
              TaCIPK32 (440) ----------------------------------------------
               TaCIPK9 (444) ----------------------------------------------
              TaCIPK22 (433) ----------------------------------------------
              TaCIPK29 (437) ----------------------------------------------
               TaCIPK4 (433) ----------------------------------------------
               TaCIPK7 (432) ----------------------------------------------
              TaCIPK12 (437) ----------------------------------------------
              TaCIPK19 (526) ----------------------------------------------
              TaCIPK25 (488) ----------------------------------------------
              TaCIPK13 (523) ----------------------------------------------
              TaCIPK10 (439) ----------------------------------------------
               TaCIPK2 (457) ----------------------------------------------
              TaCIPK11 (483) LQPQEQQNLQEQPPLPPQNGFKHQD---------------------
              TaCIPK26 (482) QQPQE-----------------------------------------
              TaCIPK28 (468) APPPQ-----------------------------------------
              TaCIPK14 (455) ----------------------------------------------
              TaCIPK15 (439) ----------------------------------------------
               TaCIPK5 (465) ----------------------------------------------
              TaCIPK27 (448) ----------------------------------------------
               TaCIPK6 (448) ----------------------------------------------
              TaCIPK30 (479) ----------------------------------------------
              TaCIPK16 (390) ----------------------------------------------
             Consensus (645)

                                                                                  Section [illegible]
                      (691) 691      700       710       720            736
    AtSnRK2.4 At1g10940 (364) ----------------------------------------------
    AtSnRK2.9 AT2G23030 (340) ----------------------------------------------
   HsSnRK NP_001094064 (549) ESRRRLDKDSGFTYSWHRRDSSEGPPGSEGDGGGQSKPSNASGGVD
 MmSnRK NP_001158044.1 (549) ESRRRLDKDSGFAYSWHRRDSSEGPPGSEGDGGGQSKPS-SGGGVD
  OsSnRK 1a Os05g0530500 (504) ----------------------------------------------
   OsSnRk1b Os08g0484600 (510) ----------------------------------------------
  ZmSnRK NP_001105673.1 (510) ----------------------------------------------
              TaCIPK20 (539) ----------------------------------------------
              TaCIPK17 (467) ----------------------------------------------
              TaCIPK21 (452) ----------------------------------------------
     AtSOS2 AAF62923.1 (447) ----------------------------------------------
              TaCIPK24 (447) ----------------------------------------------
               TaCIPK8 (450) ----------------------------------------------
              TaCIPK23 (462) ----------------------------------------------
               TaCIPK3 (448) ----------------------------------------------
              TaCIPK31 (450) ----------------------------------------------
              TaCIPK32 (440) ----------------------------------------------
               TaCIPK9 (444) ----------------------------------------------
              TaCIPK22 (433) ----------------------------------------------
              TaCIPK29 (437) ----------------------------------------------
               TaCIPK4 (433) ----------------------------------------------
               TaCIPK7 (432) ----------------------------------------------
              TaCIPK12 (437) ----------------------------------------------
              TaCIPK19 (526) ----------------------------------------------
              TaCIPK25 (488) ----------------------------------------------
              TaCIPK13 (523) ----------------------------------------------
              TaCIPK10 (439) ----------------------------------------------
               TaCIPK2 (457) ----------------------------------------------
              TaCIPK11 (508) ----------------------------------------------
              TaCIPK26 (487) ----------------------------------------------
              TaCIPK28 (473) ----------------------------------------------
              TaCIPK14 (455) ----------------------------------------------
              TaCIPK15 (439) ----------------------------------------------
               TaCIPK5 (465) ----------------------------------------------
              TaCIPK27 (448) ----------------------------------------------
               TaCIPK6 (448) ----------------------------------------------
              TaCIPK30 (479) ----------------------------------------------
              TaCIPK16 (390) ----------------------------------------------
             Consensus (691)
```

```
                            (737) 737          750       760       770         782
        AtSnRK2.4 At1g10940 (364) ----------------------------------------------
        AtSnRK2.9 AT2G23030 (340) ----------------------------------------------
       HsSnRK NP_001094064 (595) KASPSENNAGGGSPSSGSGGNPTNTSGTTRRCAGPSNS-----MQL
     MmSnRK NP_001158044.1 (594) KASPGEQGTGGGS-QGGSGGTPSGTAGSSRRCAGPDSSSPSPASAS
     OsSnRK 1a Os05g0530500 (504) ----------------------------------------------
      OsSnRk1b Os08g0484600 (510) ----------------------------------------------
     ZmSnRK NP_001105673.1 (510) ----------------------------------------------
                  TaCIPK20 (539) ----------------------------------------------
                  TaCIPK17 (467) ----------------------------------------------
                  TaCIPK21 (452) ----------------------------------------------
         AtSOS2 AAF62923.1 (447) ----------------------------------------------
                  TaCIPK24 (447) ----------------------------------------------
                   TaCIPK8 (450) ----------------------------------------------
                  TaCIPK23 (462) ----------------------------------------------
                   TaCIPK3 (448) ----------------------------------------------
                  TaCIPK31 (450) ----------------------------------------------
                  TaCIPK32 (440) ----------------------------------------------
                   TaCIPK9 (444) ----------------------------------------------
                  TaCIPK22 (433) ----------------------------------------------
                  TaCIPK29 (437) ----------------------------------------------
                   TaCIPK4 (433) ----------------------------------------------
                   TaCIPK7 (432) ----------------------------------------------
                  TaCIPK12 (437) ----------------------------------------------
                  TaCIPK19 (526) ----------------------------------------------
                  TaCIPK25 (488) ----------------------------------------------
                  TaCIPK13 (523) ----------------------------------------------
                  TaCIPK10 (439) ----------------------------------------------
                   TaCIPK2 (457) ----------------------------------------------
                  TaCIPK11 (508) ----------------------------------------------
                  TaCIPK26 (487) ----------------------------------------------
                  TaCIPK28 (473) ----------------------------------------------
                  TaCIPK14 (455) ----------------------------------------------
                  TaCIPK15 (439) ----------------------------------------------
                   TaCIPK5 (465) ----------------------------------------------
                  TaCIPK27 (448) ----------------------------------------------
                   TaCIPK6 (448) ----------------------------------------------
                  TaCIPK30 (479) ----------------------------------------------
                  TaCIPK16 (390) ----------------------------------------------
                 Consensus (737)
```

```
                            (783) 783    790       800       810             828
        AtSnRK2.4 At1g10940 (364) ----------------------------------------------
        AtSnRK2.9 AT2G23030 (340) ----------------------------------------------
       HsSnRK NP_001094064 (636) ASRSAGELVESLKLMSLCLGSQLHGSTKYIIDPQNGLSFSSVKVQE
     MmSnRK NP_001158044.1 (639) AAPRGAELVQSLKLVSLCLGSQLHG-AKYILDPQK-ALFSSVKVQE
     OsSnRK 1a Os05g0530500 (504) ----------------------------------------------
      OsSnRk1b Os08g0484600 (510) ----------------------------------------------
     ZmSnRK NP_001105673.1 (510) ----------------------------------------------
                  TaCIPK20 (539) ----------------------------------------------
                  TaCIPK17 (467) ----------------------------------------------
                  TaCIPK21 (452) ----------------------------------------------
         AtSOS2 AAF62923.1 (447) ----------------------------------------------
                  TaCIPK24 (447) ----------------------------------------------
                   TaCIPK8 (450) ----------------------------------------------
                  TaCIPK23 (462) ----------------------------------------------
                   TaCIPK3 (448) ----------------------------------------------
                  TaCIPK31 (450) ----------------------------------------------
                  TaCIPK32 (440) ----------------------------------------------
                   TaCIPK9 (444) ----------------------------------------------
                  TaCIPK22 (433) ----------------------------------------------
                  TaCIPK29 (437) ----------------------------------------------
                   TaCIPK4 (433) ----------------------------------------------
                   TaCIPK7 (432) ----------------------------------------------
                  TaCIPK12 (437) ----------------------------------------------
                  TaCIPK19 (526) ----------------------------------------------
                  TaCIPK25 (488) ----------------------------------------------
                  TaCIPK13 (523) ----------------------------------------------
                  TaCIPK10 (439) ----------------------------------------------
                   TaCIPK2 (457) ----------------------------------------------
                  TaCIPK11 (508) ----------------------------------------------
                  TaCIPK26 (487) ----------------------------------------------
                  TaCIPK28 (473) ----------------------------------------------
                  TaCIPK14 (455) ----------------------------------------------
                  TaCIPK15 (439) ----------------------------------------------
                   TaCIPK5 (465) ----------------------------------------------
                  TaCIPK27 (448) ----------------------------------------------
                   TaCIPK6 (448) ----------------------------------------------
                  TaCIPK30 (479) ----------------------------------------------
                  TaCIPK16 (390) ----------------------------------------------
                 Consensus (783)
```

```
                          (829) 829        840       850       860          874
       AtSnRK2.4 At1g10940 (364) ----------------------------------------------
       AtSnRK2.9 AT2G23030 (340) ----------------------------------------------
       HsSnRK NP_001094064 (682) KSTWKMCISSTGNAGQVPAVGGIKFFSDHMADTTTELERIKSKNLK
     MmSnRK NP_001158044.1 (683) KSTWKMCISAPG-----PS------------P-SADLDPVRTKKLR
     OsSnRK 1a Os05g0530500 (504) ----------------------------------------------
      OsSnRk1b Os08g0484600 (510) ----------------------------------------------
      ZmSnRK NP_001105673.1 (510) ----------------------------------------------
                  TaCIPK20 (539) ----------------------------------------------
                  TaCIPK17 (467) ----------------------------------------------
                  TaCIPK21 (452) ----------------------------------------------
        AtSOS2 AAF62923.1 (447) ----------------------------------------------
                  TaCIPK24 (447) ----------------------------------------------
                   TaCIPK8 (450) ----------------------------------------------
                  TaCIPK23 (462) ----------------------------------------------
                   TaCIPK3 (448) ----------------------------------------------
                  TaCIPK31 (450) ----------------------------------------------
                  TaCIPK32 (440) ----------------------------------------------
                   TaCIPK9 (444) ----------------------------------------------
                  TaCIPK22 (433) ----------------------------------------------
                  TaCIPK29 (437) ----------------------------------------------
                   TaCIPK4 (433) ----------------------------------------------
                   TaCIPK7 (432) ----------------------------------------------
                  TaCIPK12 (437) ----------------------------------------------
                  TaCIPK19 (526) ----------------------------------------------
                  TaCIPK25 (488) ----------------------------------------------
                  TaCIPK13 (523) ----------------------------------------------
                  TaCIPK10 (439) ----------------------------------------------
                   TaCIPK2 (457) ----------------------------------------------
                  TaCIPK11 (508) ----------------------------------------------
                  TaCIPK26 (487) ----------------------------------------------
                  TaCIPK28 (473) ----------------------------------------------
                  TaCIPK14 (455) ----------------------------------------------
                  TaCIPK15 (439) ----------------------------------------------
                   TaCIPK5 (465) ----------------------------------------------
                  TaCIPK27 (448) ----------------------------------------------
                   TaCIPK6 (448) ----------------------------------------------
                  TaCIPK30 (479) ----------------------------------------------
                  TaCIPK16 (390) ----------------------------------------------
                 Consensus (829)
```

```
                          (875) 875  880       890       900        912
       AtSnRK2.4 At1g10940 (364) --------------------------------------
       AtSnRK2.9 AT2G23030 (340) --------------------------------------
       HsSnRK NP_001094064 (728) NNVLQLPLCEKTISVNIQRNPKEGLLCASSPASCCHVI
     MmSnRK NP_001158044.1 (711) NNALQLPLCEKTISVNIQRSRKEGLLCASSPASCCHVI
     OsSnRK 1a Os05g0530500 (504) --------------------------------------
      OsSnRk1b Os08g0484600 (510) --------------------------------------
      ZmSnRK NP_001105673.1 (510) --------------------------------------
                  TaCIPK20 (539) --------------------------------------
                  TaCIPK17 (467) --------------------------------------
                  TaCIPK21 (452) --------------------------------------
        AtSOS2 AAF62923.1 (447) --------------------------------------
                  TaCIPK24 (447) --------------------------------------
                   TaCIPK8 (450) --------------------------------------
                  TaCIPK23 (462) --------------------------------------
                   TaCIPK3 (448) --------------------------------------
                  TaCIPK31 (450) --------------------------------------
                  TaCIPK32 (440) --------------------------------------
                   TaCIPK9 (444) --------------------------------------
                  TaCIPK22 (433) --------------------------------------
                  TaCIPK29 (437) --------------------------------------
                   TaCIPK4 (433) --------------------------------------
                   TaCIPK7 (432) --------------------------------------
                  TaCIPK12 (437) --------------------------------------
                  TaCIPK19 (526) --------------------------------------
                  TaCIPK25 (488) --------------------------------------
                  TaCIPK13 (523) --------------------------------------
                  TaCIPK10 (439) --------------------------------------
                   TaCIPK2 (457) --------------------------------------
                  TaCIPK11 (508) --------------------------------------
                  TaCIPK26 (487) --------------------------------------
                  TaCIPK28 (473) --------------------------------------
                  TaCIPK14 (455) --------------------------------------
                  TaCIPK15 (439) --------------------------------------
                   TaCIPK5 (465) --------------------------------------
                  TaCIPK27 (448) --------------------------------------
                   TaCIPK6 (448) --------------------------------------
                  TaCIPK30 (479) --------------------------------------
                  TaCIPK16 (390) --------------------------------------
                 Consensus (875)
```

Additional file 6: Analysis of SnRK proteins of representative species. Multiple sequence alignment was performed using the ClustalX1.83. Identical amino acids are shaded in yellow, conservative amino acids are shaded in wathet blue and similar amino acids are shaded in green. The PKC domain and NAD/FISL motifs are indicated in the figure.
