# Supplementary material for: Identification and comprehensive analyses of the CBL and CIPK gene families in wheat (Triticum aestivum L.)
Source: BMC Plant Biol. 2015 Nov 4;15:269. doi: 10.1186/s12870-015-0657-4 (PMC4634908; doi:10.1186/s12870-015-0657-4)
Supplement: Additional file 7: — The interaction analysis of wheat TaCBL and TaCIPK proteins were performed by Y2H method. (PDF 191 kb) [file 12870_2015_657_MOESM7_ESM.pdf]

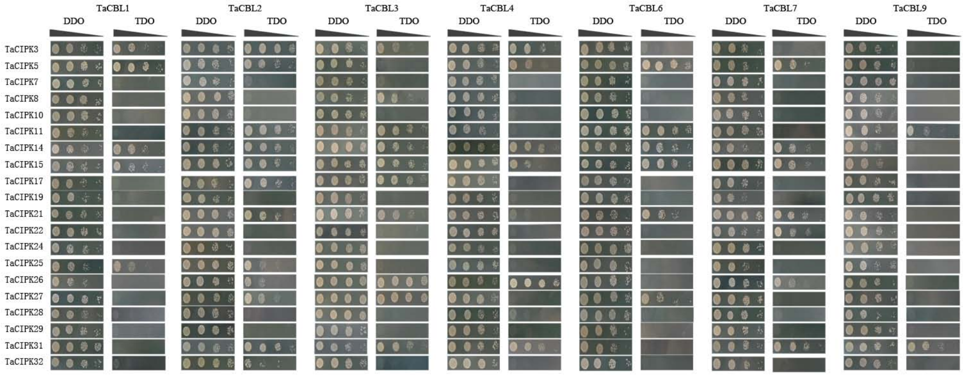
TaCBL1
TaCBL2
TaCBL3
TaCBL4
TaCBL6
TaCBL7
TaCBL9
DDO
TDO
TaCIPK3
TaCIPK5
TaCIPK7
TaCIPK8
TaCIPK10
TaCIPK11
TaCIPK14
TaCIPK15
TaCIPK17
TaCIPK19
TaCIPK21
TaCIPK22
TaCIPK24
TaCIPK25
TaCIPK26
TaCIPK27
TaCIPK28
TaCIPK29
TaCIPK31
TaCIPK32

Additional files 7. The interaction analysis of wheat TaCBL and TaCIPK proteins were performed by Y2H method. TaCIPKs and TaCBLs were respectively cloned to PGAD and PGBK vectors, and then co-transformed into Y187 strains. The transformants containing the target plasmid combinations were grown on either the double dropout medium (DDO: SD/–Trp/–Leu) or triple dropout medium (TDO: SD/–Trp/–Leu/–His/+10mM 3AT)
