# Supplementary material for: Identification and comprehensive analyses of the CBL and CIPK gene families in wheat (Triticum aestivum L.)
Source: BMC Plant Biol. 2015 Nov 4;15:269. doi: 10.1186/s12870-015-0657-4 (PMC4634908; doi:10.1186/s12870-015-0657-4)
Supplement: Additional file 8: — The bimolecular fluorescence complementation experiments. (PDF 310 kb) [file 12870_2015_657_MOESM8_ESM.pdf]

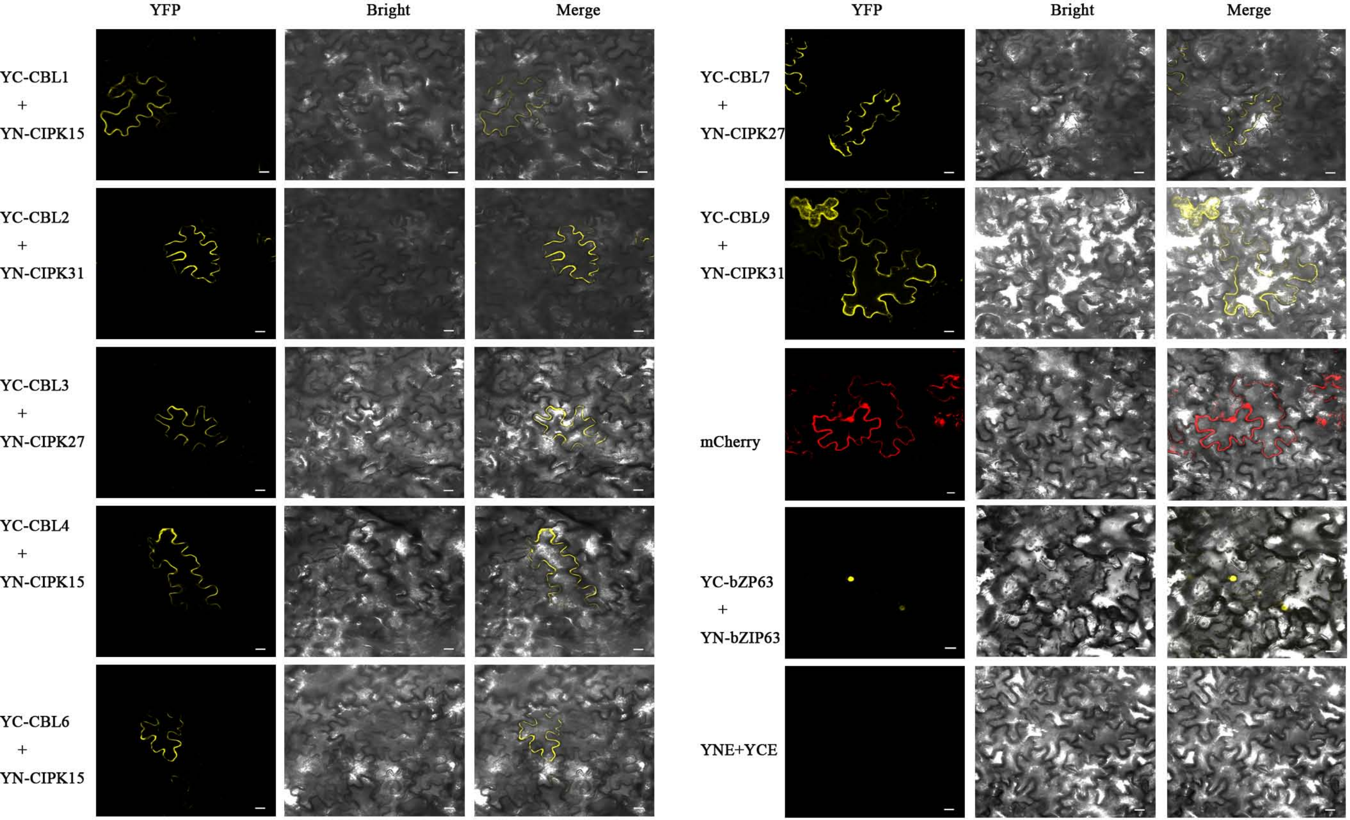

YFP
Bright
Merge
YC-CBL1
+
YN-CIPK15
YC-CBL2
+
YN-CIPK31
YC-CBL3
+
YN-CIPK27
YC-CBL4
+
YN-CIPK15
YC-CBL6
+
YN-CIPK15
YFP
Bright
Merge
YC-CBL7
+
YN-CIPK27
YC-CBL9
+
YN-CIPK31
mCherry
YC-bZP63
+
YN-bZIP63
YNE+YCE

Additional files 8. The bimolecular fluorescence complementation experiments in tobacco leaf cells. The coding region of CIPKs and CBLs were inserted into 35S-SPYNE (YN) and 35S-SPYNE (YC), respectively. The bZIP63 and mCherry proteins were used as positive controls, and YNE/YCE were used as negative control. (Scale bar: 20 μm).
