# Supplementary material for: Identification and comprehensive analyses of the CBL and CIPK gene families in wheat (Triticum aestivum L.)
Source: BMC Plant Biol. 2015 Nov 4;15:269. doi: 10.1186/s12870-015-0657-4 (PMC4634908; doi:10.1186/s12870-015-0657-4)
Supplement: Additional file 9: — Putative ABRE, DRE and LTRE core sequences in the 1-kb promoter regions of the stress-inducible genes. (PDF 114 kb) [file 12870_2015_657_MOESM9_ESM.pdf]

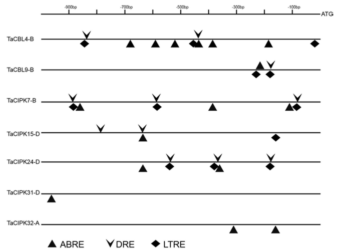

-900bp
-700bp
-500bp
-300bp
-100bp
ATG
TaCBL4-B
TaCBL9-B
TaCIPK7-B
TaCIPK15-D
TaCIPK24-D
TaCIPK31-D
TaCIPK32-A
ABRE
DRE
LTRE

Additional files 9. Putative ABA responsive element (ABRE), dehydration-responsive element (DRE) and low-temperature-responsive element (LTRE) core sequences in the 1-kb promoter regions of the stress-inducible genes identified by qRT-PCR analyses. The lines represent promoter sequences. The elements located in the promoter regions were marked beside the lines, respectively.
